# Household concepts of wellbeing and the contribution of palliative care in the context of advanced cancer: A Photovoice study from Blantyre, Malawi

**DOI:** 10.1371/journal.pone.0202490

**Published:** 2018-08-22

**Authors:** M. Jane Bates, Treza Mphwatiwa, Jane Ardrey, Nicola Desmond, Louis W. Niessen, S. Bertel Squire

**Affiliations:** 1 Department of Clinical Sciences, Liverpool School of Tropical Medicine, Liverpool, United Kingdom; 2 Department of Family Medicine, College of Medicine, Blantyre, Malawi; 3 African Network for the Care of Children Affected by HIV and AIDS, c/o College of Medicine, Lilongwe, Malawi; 4 Malawi Liverpool Wellcome Trust, Blantyre, Malawi; National Institutes of Health, UNITED STATES

## Abstract

**Introduction:**

Cancer and other life-limiting non-communicable diseases are on the increase in Africa affecting younger populations frequently diagnosed at an advanced stage of disease. The United Nations Sustainable Development Goal 3 aims for ‘healthy life and wellbeing for all at all ages’, though there is a limited understanding of wellbeing particularly from patients’ and families’ perspectives in these populations. Palliative care is an approach which aims to improve the quality of life for patients and families affected by life-limiting disease, though access to palliative care has been described as an issue which is ‘largely ignored’ on the global health agenda. The aim of this Photovoice study was to explore patient and family perspectives of wellbeing and the contribution of palliative care following a diagnosis of advanced cancer in Blantyre, Malawi.

**Methods:**

Between November 2016 and February 2017, 13 co-researchers (6 patients receiving palliative care for advanced cancer and 7 un-paid family caregivers) gathered photographs to depict aspects of their daily lives. Participatory analysis was conducted and an advocacy event (including photographic exhibits) held.

**Results:**

Wellbeing was described as seeing improvements in the patients’ function facilitating inclusion in activities of daily living (including income generation) that had not previously been possible due to their illness. Family caregivers, neighbours and community members play a key role as ‘courage givers’ supported by health workers and religious groups, though discrimination in the form of social exclusion was also reported to be significant with patients expressing that they may be considered ‘prematurely dead’ in their community. Palliative care improves wellbeing by providing pain and symptom management enabling patients and / or family caregivers to return to household and income generating tasks. Through close interaction with households and ongoing counselling palliative care services assist to reduce fear and discrimination.

**Conclusions:**

To achieve Sustainable Development Goal 3 for patients and families affected by life limiting illnesses in low resource settings, further understanding of the frequency and impact of discrimination is required as well as improved access to palliative care.

## Introduction

The number of cancer cases and cancer deaths in sub-Saharan Africa is set to increase by 70–85% by 2030[1] forming a major part of the growing challenge of life-limiting non-communicable disease on the continent. Few countries have national cancer plans, and data on cancer type, incidence and outcomes is still severely limited in scope and quality[2, 3]. For a variety of reasons over 80% of cancer in the region is diagnosed at an advanced stage requiring a palliative approach from the time of diagnosis[4]. Using the principles outlined in the WHO Public Health Strategy for Palliative Care [5] there have been a number of successes in scaling up drug availability, policy, education and service provision across Africa[6–8]. Despite this, the Lancet Commission on palliative care and pain relief reported in October 2017 that ‘access (to palliative care and pain relief) is a health equity and human rights imperative which has been largely ignored’ [9].

Because of global advocacy, the World Health Assembly passed a resolution in 2014 calling for the ‘strengthening of palliative care as a component of comprehensive care throughout the life course’. Figures from the World Health Organisation estimate that currently only 14% of the 40 million people requiring palliative care globally receive it[10] with services clustered in high income countries of the global north. The World Health Organisation’s definition of palliative care focuses on the importance of wellbeing through achieving ‘quality of life for patients and families affected by life limiting illnesses’[11]. Though lacking a consensus definition[12], wellbeing is of growing importance in wider global conversations about health following its inclusion in the United Nations Sustainable Development Goal 3 (for health) which aims to ‘ensure healthy life and wellbeing for all at all ages’[13].

Qualitative studies exploring quality of life and/or wellbeing of patients and families receiving palliative care are limited. A recent systematic review revealed only one paper (out of a total of twenty four) from the African continent[14]. Early work during the development of palliative care services in Africa used standardised questionnaires to assess the needs of patients and caregivers receiving palliative care in five countries in Africa (Zimbabwe, Uganda, Ethiopia, Botswana and Tanzania)[15]. Financial problems and pain were the most commonly expressed needs. More recently data on wellbeing has been measured in palliative care patients in South Africa and Uganda using a quality of life tool (the Missoula Vitas Quality of Life index) which was evaluated as being suitable for the setting [16, 17]. Spiritual wellbeing (being at peace and having meaning in life) was found to correlate most highly with quality of life scores.

This study used Photovoice to explore wellbeing for patients and family caregivers. Photovoice is a participatory action research method in which photographic material is gathered and used to discuss strengths and concerns of a particular group with the potential to catalyse social change[18]. Wang and Burris were the first to describe the approach in the late 1990s [19], detailing its conceptual roots in the work of South American educationalist Paolo Friere and feminist theory. In common with other participatory action research approaches it supports Robert Chambers’ notion that ‘poor and exploited people can and should be enabled to analyse their own realities’[20]. Photovoice is increasingly used both within and beyond health-related research[21, 22]. Photovoice was considered appropriate to explore this topic, as an engaging and empowering method for a traditionally ‘hard to reach’ group incorporating a strong advocacy focus. A small handful of Photovoice studies have been reported from Malawi [23–25], but to our knowledge, this was the first time it has been used for palliative care research anywhere in Africa.

Malawi is a densely populated peaceful democratic country listed 170 out of 188 on the Human Development Index [26]. Over 80% of the population live in rural areas as subsistence farmers. Life expectancy is 64 years[27]. Cancer prevention and treatment services are slowly improving but access to specialist surgical and oncology services remain extremely limited[28, 29]. Many patients are diagnosed once their disease is at an advanced stage requiring a palliative approach [30, 31]. Persistent and severe shortage of health workers necessitate greater involvement from family caregivers who are referred to as ‘guardians’ in the local setting[32].

Palliative care services are provided to a relatively young population facing irreversible progressive illness during what should be their most economically productive years[33]. Nationally coordinated efforts in Malawi have resulted in increased morphine usage[34] (a commonly used proxy for palliative care development), integration of training modules, and service delivery expansion in public sector hospitals [35]. Despite these successes, centres of excellence for palliative care in Malawi remain heavily reliant on external donor funding, challenging their sustainability. The Tiyanjane palliative care team has provided palliative care for adults through hospital and community teams based in government facilities in Blantyre district in the Southern Region of Malawi since 2003. Blantyre is the second city of Malawi and is sometimes referred to as its commercial capital. Ndirande is a township area, four kilometres from Blantyre city centre with a population of around 250,000 people. Community-based palliative care services have operated from Ndirande Health Centre since 2005.

To explore concepts of wellbeing and the contribution of palliative care to wellbeing from the perspective of patients and families affected by advanced cancer we undertook a Photovoice study amongst households receiving services at Tiyanjane palliative care clinic in Ndirande.

## Methods

Informed and voluntary written consent was gained from all study participants. All households in which a patient was receiving palliative care from Tiyanjane clinic for a diagnosis of advanced cancer within the catchment of Ndirande health centre were considered eligible. At the start of the study this comprised sixteen households, all of whom were approached in advance of the study by the community palliative care nurse. The nurse provided the patient information sheet (S2 File) and discussed any questions about the study with eligible households. Co-researchers (i.e. patients and family caregivers) identified themselves through choosing to attend the first group meeting. The community palliative care nurse followed up non-attenders by phone to check they did not want to take part in the study. The research team comprised six patients and seven household members (self-identified as main un-paid family caregivers) from eight households, seven field workers (home based care volunteers from the community), two field work coordinators (palliative care nurses), two qualitative researchers (one junior and one senior), one photographer and the research lead (a palliative care physician). All team members were Malawian except for the research lead who is a British family physician (JB) who has been instrumental in establishing palliative care services over the last fourteen years within the community where the study took place.

The study was carried out in Ndirande township (four kilometres from Blantyre city centre) where the Tiyanjane community palliative care team is based at the non-fee-paying government health centre. Community sites away from the health centre were used for the group meetings and advocacy event. A two-stage consent process was used for co-researchers: at the start of the study (for participation) and towards the end of the study (relating to disclosure and sharing of photographic material).

Ethical approval for the study was obtained from the College of Medicine Research Ethics committee (P.07/16/1999) and the Research Ethics Committee of Liverpool School of Tropical Medicine (16–045). All individuals in this manuscript have given written informed consent (as outlined in PLOS consent form) to publish these images.

The Consolidated criteria for Reporting Qualitative Research checklist (COREQ) was used for reporting[36] (S3 File).

At the first group meeting co-researchers were informed about the study, then they were invited to ask any outstanding questions before being given the opportunity to consent to participate. They were given digital cameras and received basic training in their use. Over the period of one month (November 2016) they gathered images on the subject: ‘the story of my illness’. Practical issues with the cameras were discussed and images selected during one to one sessions immediately before the start of each group session. Field notes were taken by the qualitative researchers (CK, TA) during these sessions. All co-researcher images were transferred to password protected files on the laptop used by the Principal Investigator and backed up securely on a password protected hard drive which was kept in a lockable cabinet. Selected images were printed into hard copies for use in participatory analysis (S4 File). Further details of the conduct of the study have been published separately[37].

Participatory analysis took place during seven (out of a total of nine) group sessions led by the two qualitative researchers: CK (junior qualitative researcher) who had previously conducted a number of focus group discussions (including one linked to a recent photovoice study in rural Malawi) and TA (senior qualitative researcher, MPH) who has many years of qualitative research experience in the health sector. Both were Malawian females, and fluent in Chichewa—the local language of co-researchers. In the first phase (during five group sessions) co-researchers sorted through hard copies of their selected images together, grouping them together into categories with a similar meaning or ‘message’. After each grouping exercise these categories were further explored through a process of reflection and critical dialogue using locally adapted prompts based on the SHOWeD prompt described and used by Caroline Wang[18] in earlier Photovoice projects. During the process of dialogue co-researchers agreed on and named these categories. Towards the end of the first month through reviewing images and reading and re-reading categories aloud as a co-researcher group, categories were brought together into named themes.

As a separate exercise (across two group sessions), co-researchers were invited to individually select photographs for which they composed a short-written piece, conveying what they wanted to tell others through that image (‘captioning’). Wherever images were chosen which contained identifiable images of people not directly involved in the study, co-researchers were given consent forms in local language and asked to obtain consent. Where consent was not gained, photographs were excluded from further use. Twenty-seven captioned photographs were displayed in a photographic exhibition at an advocacy event which was staged by the research team at the end of the data collection period. Local health and community leaders and the media were invited to attend, and a speech was delivered by the local district health officer.

Group discussions were audio recorded then transcribed verbatim in local language (Chichewa) and translated into English (S1 File). A proportion of translated scripts were subjected to quality control by a local third party with expertise in translation and transcription. Secondary deductive analysis was based on the themes and categories identified by the co-researchers, and is reported here, in an attempt to stay as close as possible to a community based participatory research approach. Deductive coding was conducted manually by the Principal Investigator (JB, partially fluent in local language). Analyst triangulation was undertaken by the senior qualitative researcher (TA, fluent in local language) coding all the transcripts and a further selection of transcripts was coded by a third (external) qualitative researcher (JA with experience using Photovoice in rural Malawi). This team met to discuss and reflect on their findings, allowing the opportunity to return to the data to clarify inconsistencies or differences in understanding between the three researchers. Provided that there was appropriate consent, images and quotations illustrative of the co-researchers’ themes and categories were identified by the Principal Investigator for inclusion in this report.

Following secondary analysis, a final group meeting was held. Co-researchers reviewed all their selected images and revisited the categories and themes which they had identified. They were then asked whether they wanted to identify any new categories or themes.

## Results

A summary of co-researcher demographics is shown in Table 1.

**Table 1:** Summary of co-researcher demographics.

Variable	values	Frequency
**Age**	<40	6
	>40	7
	Median	44.5
	Range	33–66
**Sex**	Male	6
	Female	7
**Role**	Patient	6
	Family caregiver	7
**Diagnosis [cancer type in the household]**	Cancer (Kaposi’s Sarcoma)	5: all HIV +
	Cancer (other)	3 (brain, cervix, thyroid): one HIV +
**Religious affiliation**	Islam	1
	Christian (various)	10
	Other (Jehovah’s Witness)	2
**Length of time receiving palliative care**	Months (median)	42 (range 9–120)

Co-researchers generated five themes incorporating ten categories as shown in Table 2.

**Table 2:** Co-researcher themes and categories.

	Themes	Categories
1	**Things that make us happy**	1.1What is a good day
		1.2 After being sick then being happy
		1.3 Working
2	**Courage givers**	2.1 Role of guardian
		2.2 prayers
		2.3 the cancer patient receiving medicine from the guardian
3	**Discrimination**	
4	**Cancer as an illness**	4.1 Cancer as a disease
		4.2 Patients with illness
5	**The help you get from the hospital**	5.1 Help from Tiyanjane
		5.2 Transport

### Theme 1: Things that make us happy

The ability to work or perform daily chores (such as walking, cooking and cleaning) was considered important for what was ‘a good day’ (1.1):

“We should be able to walk like that granny when we have woken up better, walking so that the legs should be strong”.

**Fig 1:**
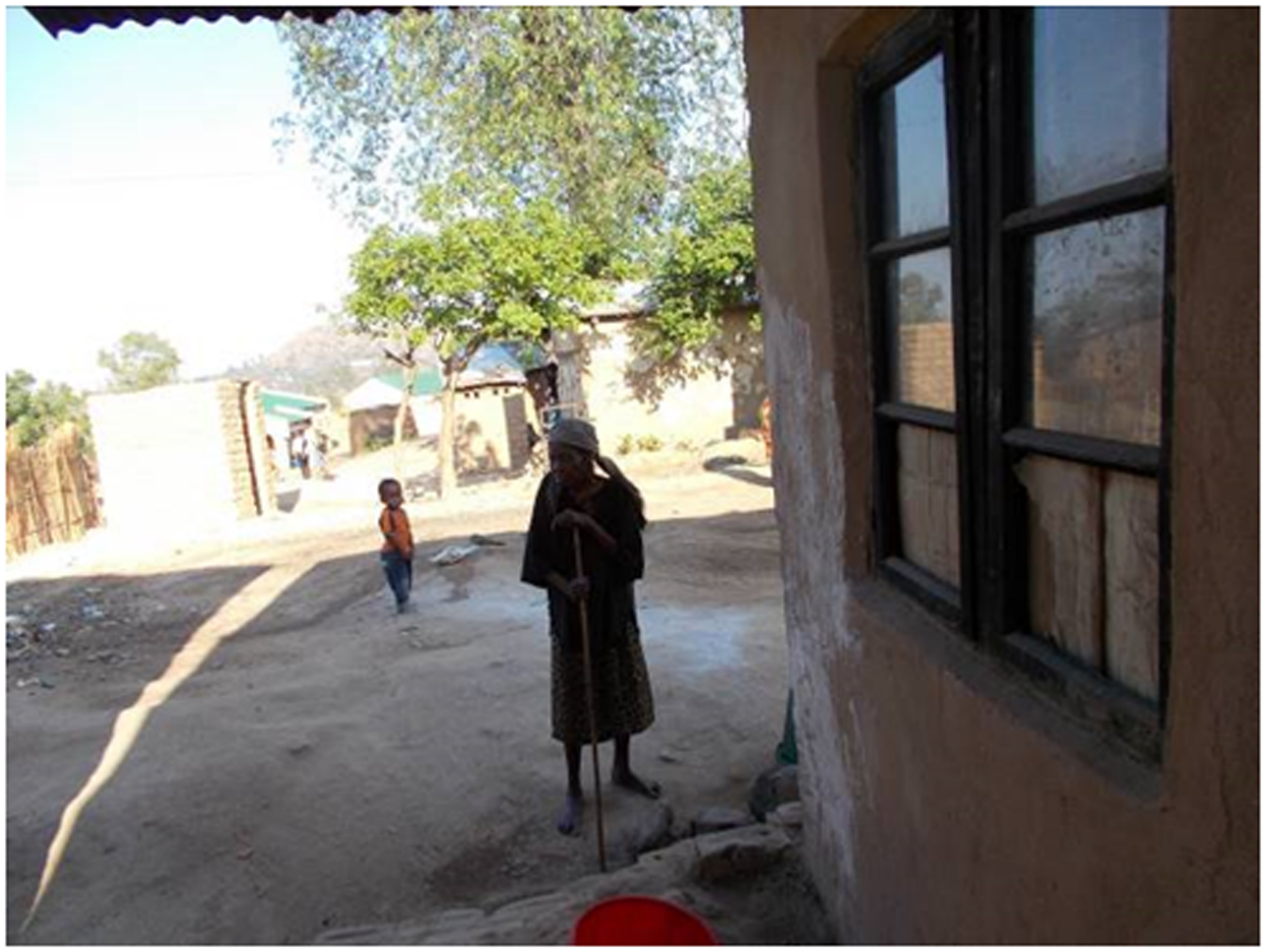
What is a good day?

PT patient 34 years male (Fig 1)

“Here this lady is washing her clothes. She woke up strong this day that she could wash on her own”.

**Fig 2:**
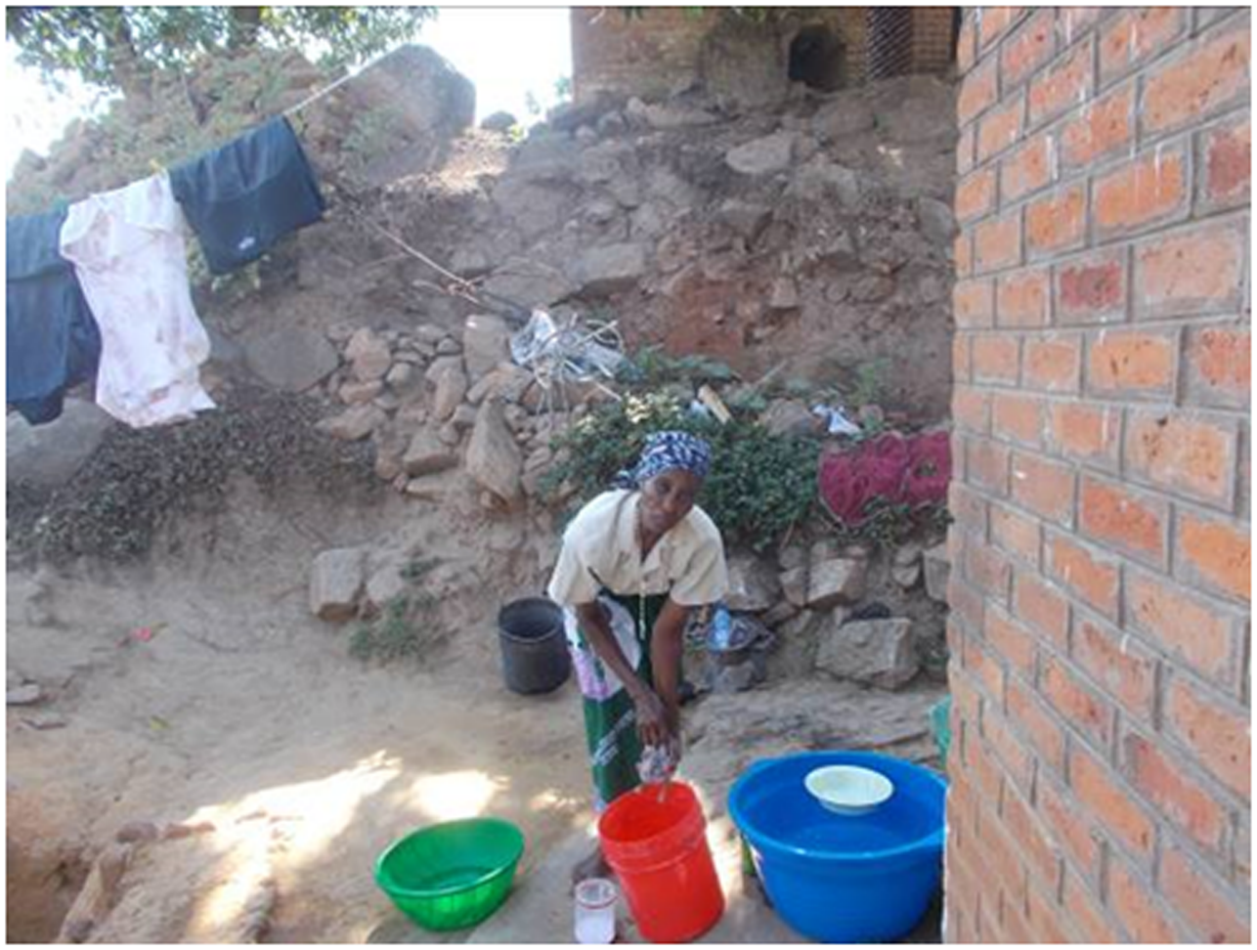
After being sick then being happy.

MM family caregiver 36 years male (Fig 2)

Seeing improvement in the patient brought happiness (1.2 ‘after being sick then being happy’, 1.3 ‘working’).

“…even though some of us are sick from this disease we are working because of the medicine that we are receiving from Tiyanjane, they give us medicine but seeing that we are able to walk and we are working knowing that our family should be ok because of the medicine that we are given”.

**Fig 3:**
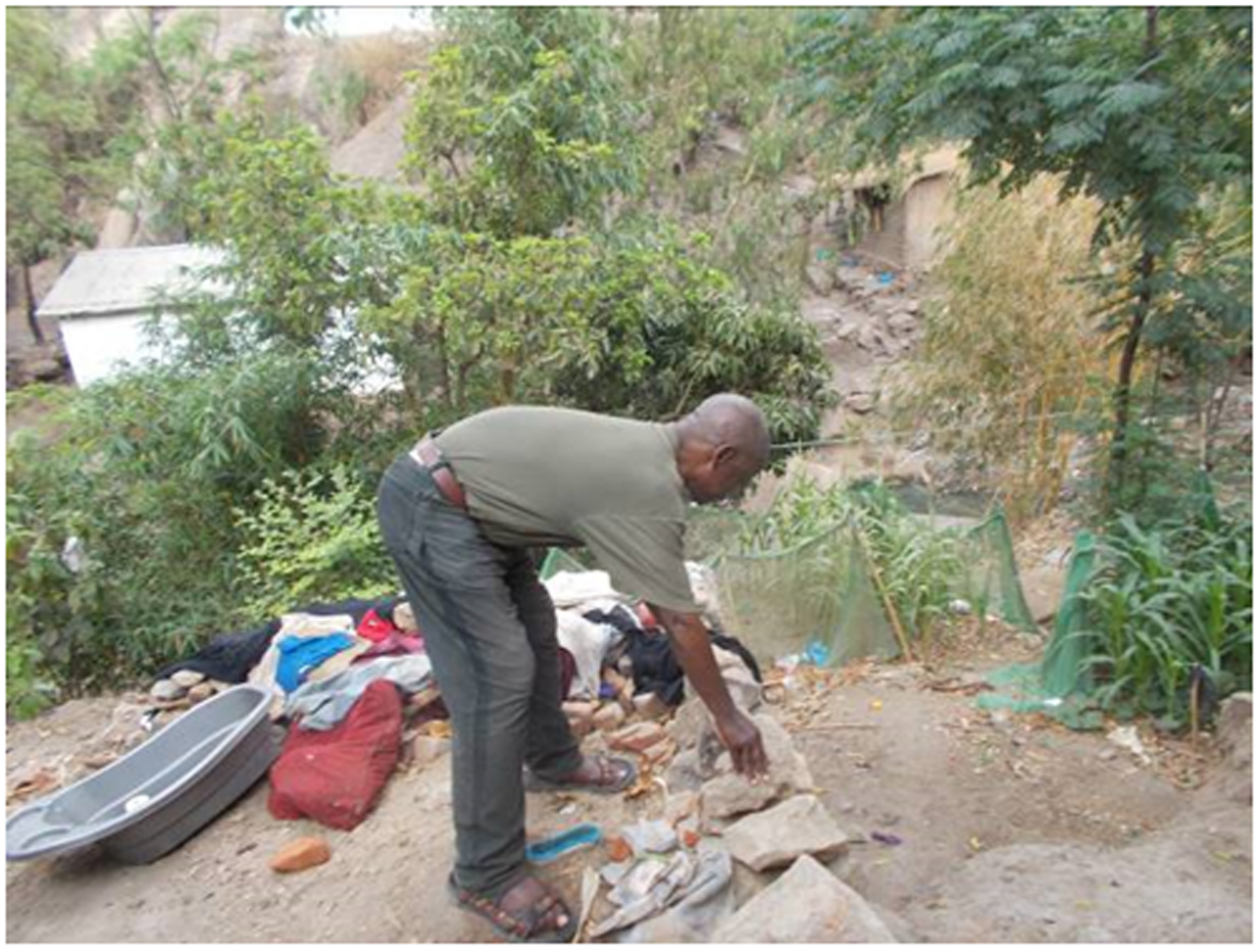
Working.

GM patient 62 years male (Fig 3)

### Theme 2: Courage givers

Courage was provided from a variety of sources: children, older family caregivers (2.1 ‘role of the guardian’), neighbours, religious organisations (2.2 ‘prayers’). Health services worked together with community-based support (2.3 ‘the cancer patient receiving medicine from the guardian’) though ‘health worker’ was not identified as a separate category within the theme.

“The people who happen to encourage us on some other things for example maybe at home…being free with you by talking to you that you are not supposed to be worried this is not the end but the beginning …those are the category of people assisting in your everyday life like the everyday food and activities.”

**Fig 4:**
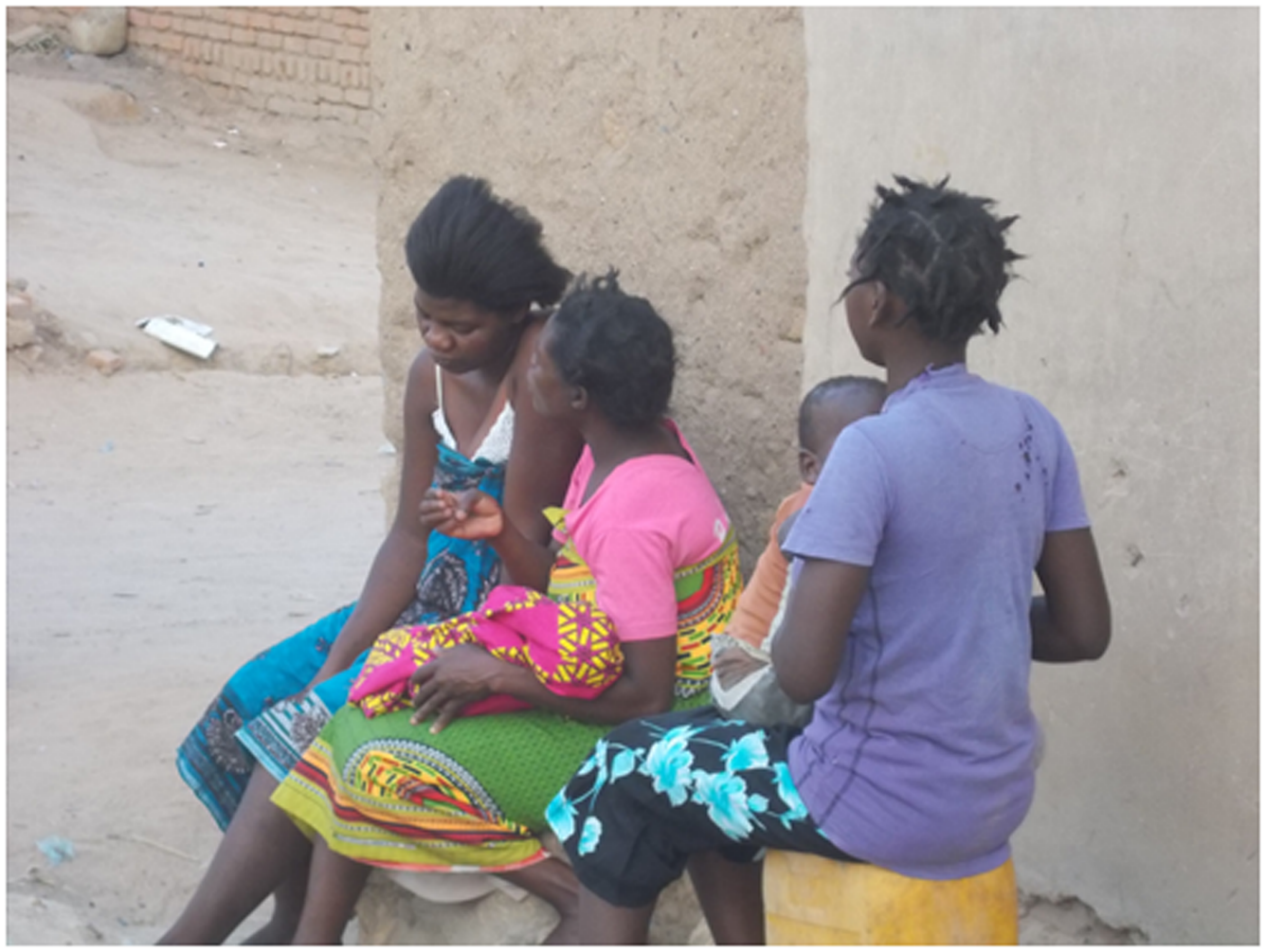
The role of the guardian.

PJ 37 year old female patient (Fig 4)

“I can see some carrying bibles…It’s not that your life has come to an end just because you have been diagnosed with cancer so those people comes and happen to give you courage with the words from the bible”.

EK 49 year old female patient

Children were courage givers:

“I took this picture so that she should be eating the food, the children were coming around so that she should be eating”.

**Fig 5:**
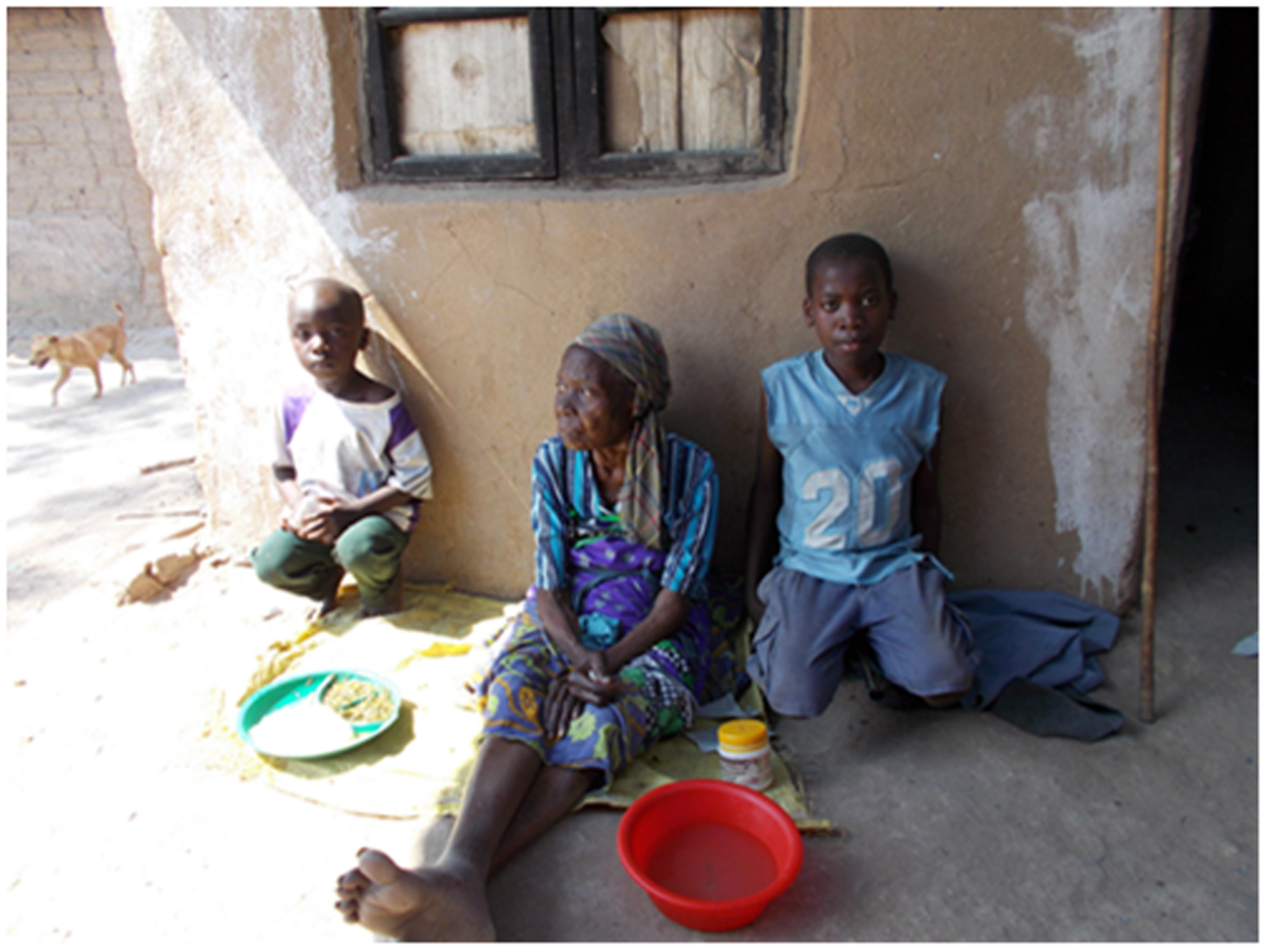
Children as courage givers.

EM 56 year old female family caregiver (Fig 5)

and health workers, “when you go there they encourage you and gives you enough courage that …although I have been diagnosed with cancer it doesn’t mean that I will die today, no I will be alive as long as I follow what the Tiyanjane people are saying”. PJ 37 year old female patient.

### Theme 3: Discrimination

All agreed on this thematic area, although they commented that it was a difficult area to illustrate using photography. Co-researchers also used this theme for the advocacy event. Discrimination was described as being due to cancer patients being thought of as being infectious and/or ‘prematurely dead’.

“My wife left me when I first got sick of cancer, but my child is happy now”

**Fig 6:**
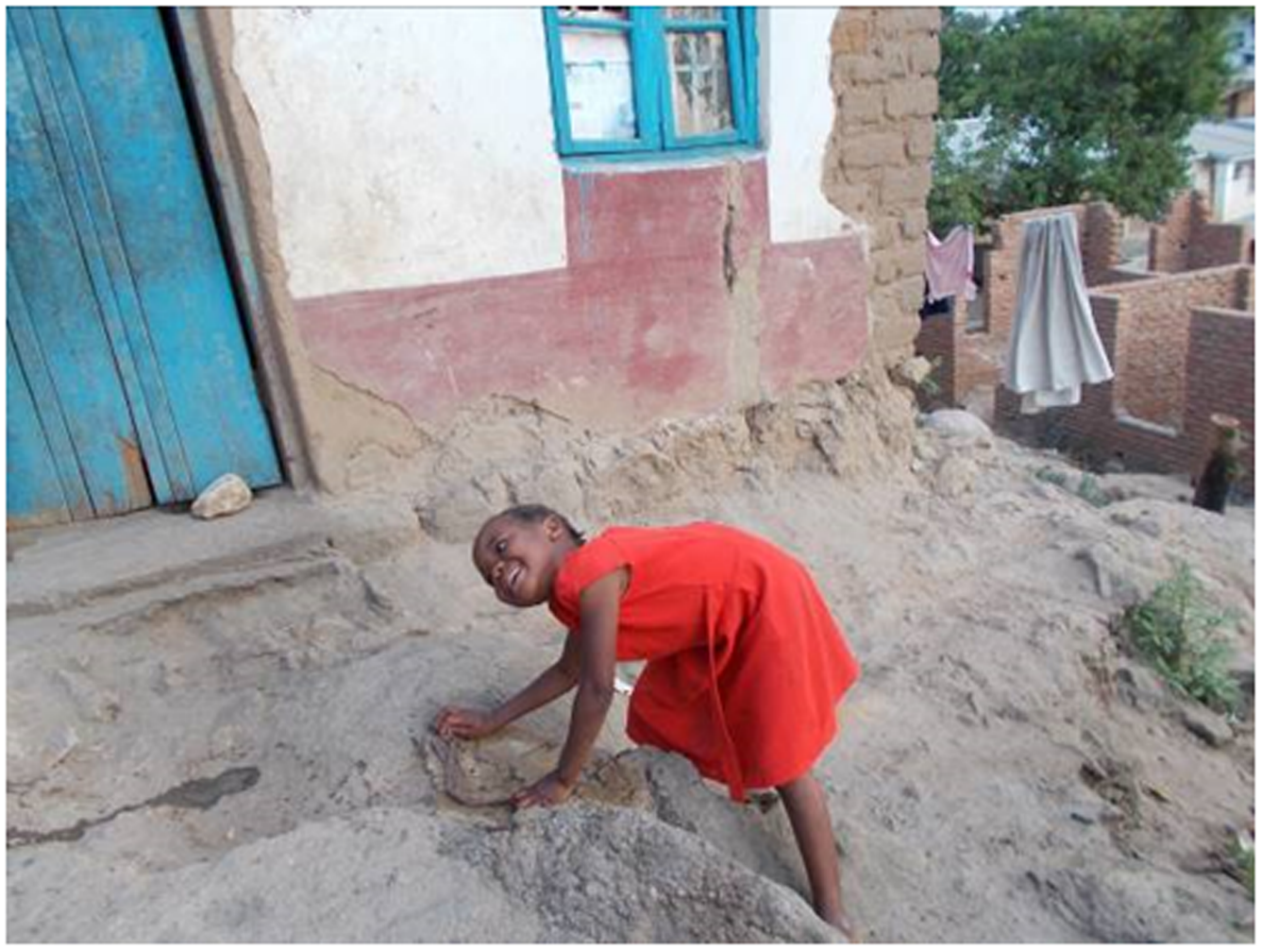
Discrimination.

PT, 34-years male patient (Fig 6)

He also reported from his experiences:

“there are some neighbours who insult the cancer patient, for example I was carried on the back, from bus stage to (hospital) and to home. They could insult by spitting and saying that, ‘that one will die and in 3 weeks, there will be a funeral at that house, he should just die’. And some spit when they see me failing to walk, so (I) am asking that these neighbours should not speak these things.”

Another co-researcher added, “There are some people who said that if a person suffers from cancer (they) will automatically die…these rumours are coming from homes”. ET 60-years female family caregiver,

Discrimination could impact the amount of food a patient might be given:

“…if they are discriminating you on some other things…while they have eaten good food but giving you things that ‘just give him a little something to eat’ then the disease continues.”

CB 66 years male patient

### Theme 4: Cancer as an illness

Co-researchers highlighted the worries, shock and concerns caused to patients and families when they hear the news that they have a diagnosis of cancer (4.2 ‘patients with illness’).

“The guardian is disappointed because the results from the hospital are saying (it) is cancer disease”.

**Fig 7:**
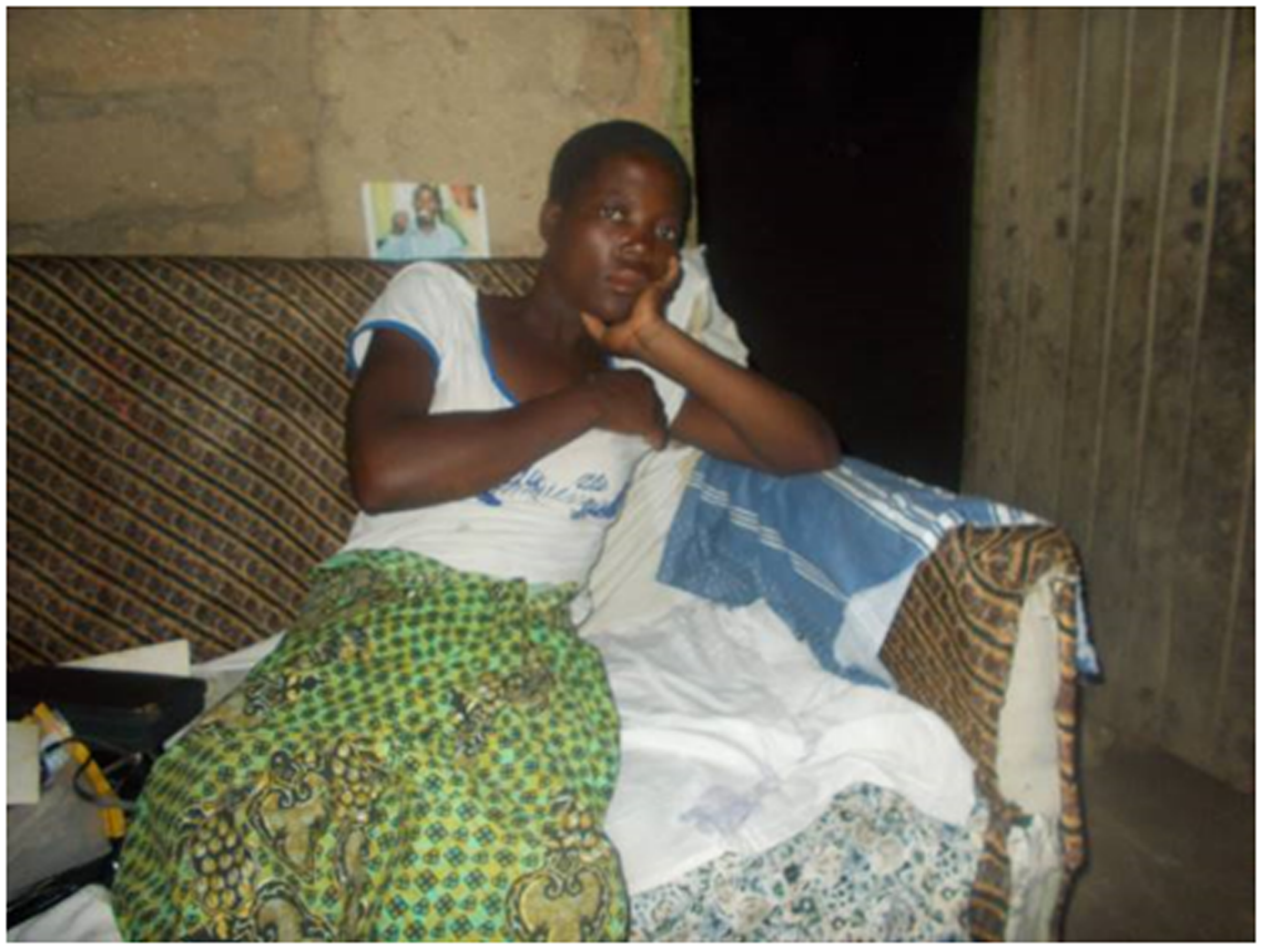
Cancer as a disease.

JM 44 years male patient (Fig 7)

Co-researchers expressed their concerns about cancer, including the lack of appropriate messages available in rural communities about the signs and symptoms of disease (4.1 ‘cancer as a disease’), resulting in delayed presentation to the hospital.

“I feel messages concerning cancer are not widespread…in the villages it is very difficult to reach out because some people have no radios. So if the country or the government can take part by using different ways to disseminate the message of cancer so (we) should know this problem earlier before it gets worse”,

JM 44 years male patient

### Theme 5: The help that you get from the hospital

Palliative care services (Tiyanjane) were reported to provide counselling and medication which assisted patients to return to work (5.1 ‘help from Tiyanjane’).

“I now work properly on my business and all my household chores after receiving medication from Tiyanjane”.

**Fig 8:**
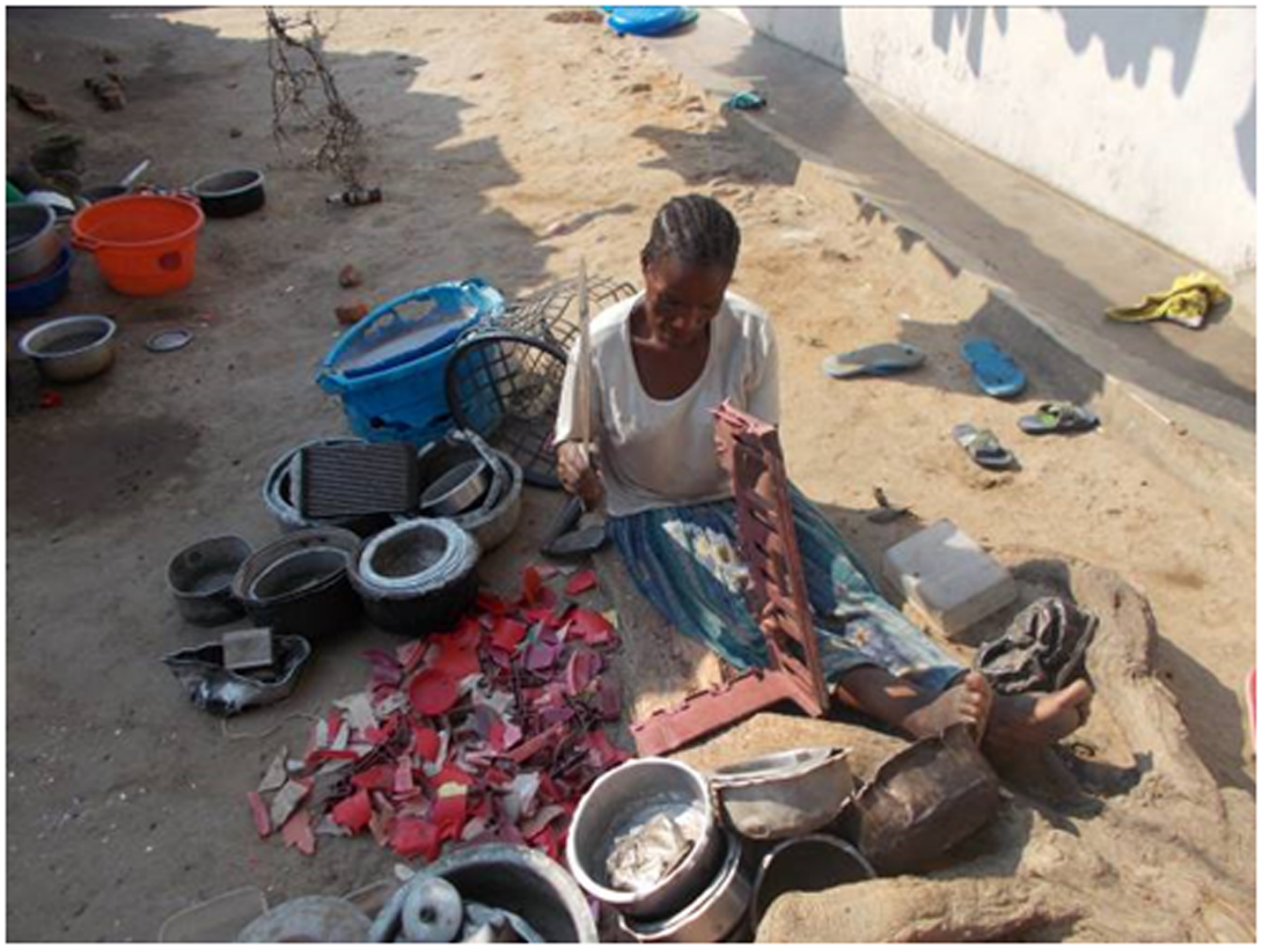
Help from Tiyanjane.

PJ 37 years female patient (Fig 8)

Patients and family caregivers were supported to stay together and care for one another by reducing discrimination:

“The children also getting closer to the patient…not being far away because of the counsel from Tiyanjane. Here, without Tiyanjane, our guardians taking care (for us patients) would have been far from us”.

JM 44 years male patient

This was contrasted with the experience of using traditional healers.

“the traditional doctor only knows how to make cuts on the body and to apply his medicine and this makes the wound get worse in the end…we say we have been bewitched and we end up hating other people. The situation gets worse and you are not free in your family”.

PJ 37 years female patient

Co-researchers highlighted delays in diagnosis and receiving care because of transport problems and (palliative care) services only being available at central hospitals rather than at health centres (5.2 ‘transport’).

“In my case I had to travel up and down for more than three weeks in order to be assisted. I did that because my guardian assisted”.

**Fig 9:**
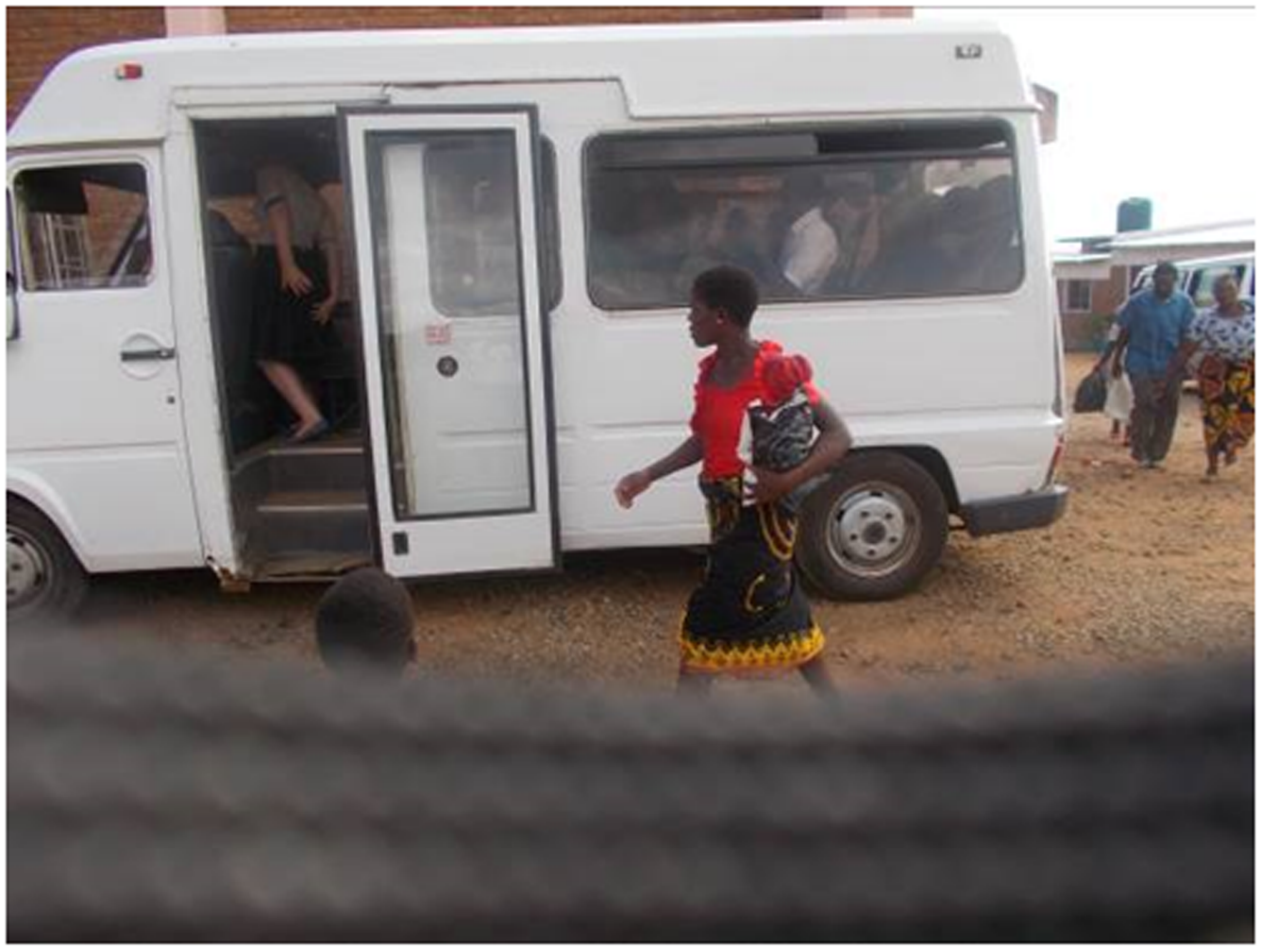
Transport.

JM patient 43 years male (Fig 9)

## Discussion

In this study, we used Photovoice—a participatory action research methodology—to explore household concepts of wellbeing and the contribution of palliative care towards wellbeing within the context of a well-established community based palliative care programme, enabling households to document, reflect on and report their everyday realities. Co-researchers (patients and family caregivers) played a central role in the analysis approach, to merge the rationale for use of Photovoice as a participatory (and thus empowering) tool with its use as a participatory research approach to ensure the presentation and interpretation of subjective viewpoints. Three themes relating to wellbeing (things that make us happy, courage givers and discrimination) and two themes relating to the contribution of palliative care (the help we get at the hospital, cancer as an illness) emerged from the process of participatory analysis.

Palliative care staff (physician, nurses and volunteers) well known to the co-researchers were included in the study team to monitor any changes in health status and to provide a point of contact in case of any difficulties between the dates of the group sessions. None of the co-researchers had used cameras or been involved research before. Long-term relationships between palliative care staff and co-researchers facilitated accountability and acceptability of the process, though is likely to have introduced some bias. For example, reports of care from the hospital were consistently positive, contrasting with reports of use of traditional healers (assumed to be widespread) which were consistently negative. Balancing safety, trust and study acceptability for co-researchers whilst eliminating bias was challenging. By using venues away from the health centre and qualitative researchers for individual and group discussions we tried to reduce this bias where possible.

Although this study relied on convenience sampling for recruitment, cancer type, co-researcher age, sex and educational levels were similar to those from more comprehensive cancer population reports in Malawi [38]. Households were selected from a peri-urban setting rather than from rural settings where the majority of Malawi’s population lives. Patients with advanced cancer had to be fit enough to use a camera and engage with group activities over a three-month period. The median age of co-researchers was 44 years (range 33–66); three older patients were excluded from the study due to their inability to use the cameras, thus views of older patients may not have been adequately represented.

Co-researchers’ first theme ‘things that make them happy’ was described through the categories 1.1: ‘what is a good day?’ 1.2: ‘after being sick then being happy’ and 1.3: ‘working’. Seeing improvement in the patient and being able to take part in daily activities such as work, cooking, cleaning, farming and religious meetings were important to wellbeing. These findings are similar to results from research done amongst healthy subjects in rural Malawi. Greco et al conducted fifteen focus group discussions amongst women’s’ groups to try to determine ‘what is a good life?’. They found that wellbeing was understood beyond basic needs (food, shelter etc.) to include the importance of emotional wellbeing, social functioning and contribution [39]. Wellbeing experienced through improved function may be related to the co-researchers’ relatively young age (and lack of readiness to accept chronic ill health) and could also be explained by the reduction in self-perceived notions of being a burden, though this was not explicitly stated.

The role (‘duty’) of family caregivers (known in Malawi as ‘guardians’) as ‘courage givers’ was the second theme identified. Receiving a diagnosis of cancer was one of the events co-researchers reported to be associated with anxiety, in common with studies from other settings[40, 41]. Anxiety has been linked to worse outcomes for patients with advanced cancer [42]. Caregivers were reported to be responsible for practical tasks such as provision of adequate food, medicine (2.3 ‘the cancer patient receiving medicine from the guardian’) and basic cleanliness of the patient, as well as reducing anxieties by providing company, distraction and ‘chatting’. Prayers (category 2.2) delivered by religious groups were included in this theme. Prayers gave courage to co-researchers, though criticism was directed to community members who promoted prayers in place of medication, with a ‘prayers plus medication’ approach favoured.

A third theme of discrimination was discussed at some length by co-researchers and chosen as their theme for the advocacy event, although few photographs were taken to illustrate this. This demonstrates that Photovoice can prompt discussion around negative and/or sensitive issues, even when it may be difficult to use photography. Co-researchers reported fear of infection being cursed and being considered as ‘prematurely dead’ as factors which contribute to this phenomenon. It was beyond the scope of this study to explore whether experiences of discrimination were subjective or objective though co-researchers suggested that discrimination could contribute to delayed presentation of disease, particularly in rural areas where disease understanding was limited. It is also beyond the scope of this study to distinguish between whether the discrimination is due to their cancer or their HIV status, however other published work suggests that internalized stigma in Malawi maybe less than in other countries. HIV was not mentioned by participants during group discussions. Much of the description of stigma related to a sense of whether or not someone was seen [or perceived themselves] as ‘useful’ in the context of society, this was related to the impact of the illness on their functional state rather than to any specific diagnosis. Discrimination was also reported to be the cause of being abandoned by a spouse and receiving less food. Discrimination through spousal abandonment is consistent with our recently published case series review of women receiving palliative care for cervical cancer at Tiyanjane, which notes relatively high rates of isolation (through divorce and separation as well as widowhood) at household level [43]. An understanding of the principle of ‘ubuntu’ may provide deeper insight into perceptions of discrimination. Ubuntu is an African concept which defines meaning and purpose through the identity of the group rather than the individual [44]. This collectivist (rather than individualist) mindset would support observations from practise which note that similarity within ‘the group’ is more highly valued than diversity in Malawi’s socially conservative culture. This could explain, at least in part, how households affected by cancer or other chronic disease may perceive themselves as different and thereby discriminated against by the wider community. There is an urgent need for further studies to explore the perceptions, frequency, experiences and consequences of discrimination amongst patients with cancer in Malawi.

Under theme four ‘cancer as an illness’ co-researchers expressed their concern about lack of appropriate health messages about cancer. This was felt to particularly affect people in rural areas, where beliefs that the illness is a result of a curse could lead to seeking advice from witch-doctors which resulted in delays in presentation to health services. They identified a role for themselves in carrying cancer messages to their communities, something which they discussed again in the closing stages of the project when the group met to review their identified categories and themes. This reflects the potential for ‘enhanced community engagement in action and advocacy’ discussed in Catalani and Minkler’s review of Photovoice projects [45] and would be an important area for future work.

Palliative care was reported to contribute to household wellbeing under theme 5 ‘the help you get from the hospital’. Under category 5.1 ‘help from Tiyanjane’, provision of medication for pain and symptom relief enabled patients and family caregivers to continue with or return to household roles which has been lost due to illness. There were links to theme 1 where seeing improvement in the patient through palliative care was highlighted under category 1.2 ‘after being sick then being happy’. The positive contribution of palliative care is reported in systematic reviews of the literature from high resource settings [46] though less has been described from low and middle income countries to date. This reflects the relative dearth of published research in palliative care from such settings [47] where locally validated quantitative outcome measures are still under development [48, 49]. Co-researchers reported that palliative care services helped to reduce stigma and discrimination through counselling of patients and families and by modelling close contact in clinical settings and through visiting patients at home. Results from our study suggest that ability to return to income generating activities/activities of daily living (for both patient or caregiver) and stigma/discrimination scores should be included in palliative care outcome measures, particularly in settings where younger patients receive palliative care and where other disease modifying approaches (such as chemotherapy and radiotherapy) are unavailable.

## Conclusions

This study reports the first Photovoice study in an African setting amongst households affected by advanced cancer who are receiving palliative care. Wellbeing for households affected by advanced cancer in Blantyre Malawi includes seeing improvement enabling patients or family caregivers to return to household and community activities. ‘Courage givers’ provide basic necessities (food, medicine and hygiene) as well as reducing anxieties relating to the illness. Discrimination negatively impacts wellbeing and may be implicated in late presentation of disease. Palliative care contributes to household wellbeing through improving pain and symptom management enabling patients and /or family caregivers to return to household and income generating tasks. Counselling and close interaction with cancer patients provided by palliative care services helps to reduce fears and discrimination. To achieve the United Nations Sustainable Development Goal 3, greater understanding of experiences and impact of discrimination and timely access to community based palliative care services will be needed for households affected by cancer and other life limiting non-communicable diseases.

## Supporting information

S1 File Anonymised transcripts. (DOCX)

S2 File Patient information sheet English version. (DOCX)

S3 File COREQ checklist. (PDF)

S4 File Example images. (ZIP)
