# Supplementary material for: Household concepts of wellbeing and the contribution of palliative care in the context of advanced cancer: A Photovoice study from Blantyre, Malawi
Source: PLoS One. 2018 Aug 22;13(8):e0202490. doi: 10.1371/journal.pone.0202490 (PMC6104988; doi:10.1371/journal.pone.0202490)
Supplement: S1 File — (DOCX) [file pone.0202490.s001.docx]

FGD1

I: Ndiye tikamalankhula ndikupemphani kuti muziyankhulako mokweza mawu kuti tizikatha kumva bwino bwino pamene tikamvesera zomwe mukufotokozazo komanso kuti anzanu ena azitani azitha kumva bwino bwino eee

Now I would like ask you that when speaking you should be raising up your voices so that we can hear you properly when we will be hearing from what you will be explaining and also for your friends to be getting you properly as well. Yes

01/01: Mmene muja mumayamba speech mmandimva ine ndikuyankhula?

Ever since you started your speech did you heard me talking?

I: Ayi

No

01/01: Mawu sakutuluka,

My voice can’t come out,

I: Owoo ayi palibe vuto

Ok, no there is no problem.

01/01: Mawu sakutuluka

My voice can’t come

I: Chabwino chabwino

Ok. Ok

01/01: Ndimakuwa kwambiri ndimabwebweta koma

I talk a lot on top of my voice but…

I: Mmmm

Mmmm

01/01: Mawu sakutuluka

My voice can’t come out

I: Ayi, enafe tikhoza kumalankhula bwino bwino moti tizimvana

Ok the rest of us here can be speaking properly so that we can be getting each other.

(interruptions)

(Interruptions)

I: Ndiye kwa amene, kwa amene ajambula chithunzi chikuoneka apocho timafuna mutifotokozele kuti chithunzi chimene mwajambulachi, mutifotokozele chithunzi chimene mwajambulachi inuyo

So to the one, the one who took that picture over there, we want you to explain to us about this picture, you should explain on the picture you have taken.

02/01: Owoo chithunzi chimenechi mawu ake ndi ochepa koma

Ok this picture here, there are few words for it

I&02/01: (kuseka)

(laughing)

02/01: Ndiochepa kwambiri.

They are very few.

I: Eee

Yes

02/01: Eya chithunzichi ndachijambula mmalomoti uyoyo ndi mwana wanga omaliza wavala choyelayo ya ku game’yo ndi amene akundisamala

Yes I took this picture, that one is my last born child the one putting on white shirt for the game is the one who takes care of me,

I: Owoo

Ok

02/01: Ngakhale ali pa sukulu koma amandisamala sanandinyozepo iyayi eya ndiye kuti ndizimujambula iyeyo

Although he is still in school, he cares for and he has never let me down yes thats why I took a picture of him

I: Owo

Ok

02/01: Eya

Yes

I: Ndi mwana wanu?

Is he your son?

02/01: Mwana wanga omaliza

My last born child

I: Ali ndi zaka zingati?

How old is he?

02/01: Anabadwa 1995

He was born in 1995

I: 95

95

02/01: Eya

Yes.

I: Amaphunzila kuti?

Where does he go to school?

02/01: Ku Namalimwe open

At namalimwe **open.**

I: Secondary school

**Secondary school.**

02/01: mmm

Mmm.

I: Chabwino chabwino

Alright alright

I: Winayo?

The other one?

02/01: Uyoyo ndekuti wantchito wathu amagwila ku shop

That one is our worker works at the shop

I: Owoo

ok

02/01 Eya tinali limodzi malingana ndikuthima thima kwa magetsi

Yes we were together due to the situation of electricity outages

I: Owoo

Ok

02/01: Eee

Yes

I: Ndiye pachithunzi chimenechichi chimachitika ndichani? anthuwo amatani pa chithunzipo?

now on this picture what was happening? what were those people doing?

02/01: Anthu amenewa ama nditangobwera poti aaa mwakuti mwakuti nde ndimawafotokozera mmene ndayendera kuno, eya kuwawuza kuti ondithandiza amene mukundisamalanu ndikuyenera kuti ndikujambuleni kuti anthunso azikuwonaniso akuziweni ndimmene mukundisamalila
These people, when I came back aaa this and that then I was telling them how I have come back from here, yes telling them that you as the ones who helps in taking care of me, am supposed to take a picture of you so that others should be seeing you and they should know the way you takes care of me.

I: Mmmm

Mmmm

02/01: Eeee

Yes

I: Nanga potoyo?

What about that pot?

02/01: Aaa mbale imeneyo inangokhala pakhonde

Aaa that plate was just at the veranda

I: Owoo

Ok

02/01: Eee

Yes

I: Chabwino chabwino

Alright alright

02/01: Ndithu

Sure.

I: Ndiye mwafotokoza kuti chifukwa choti iwowo ndi amene amakusamalani paliso chifukwa china chimene muwajambulila anthu amenewa, chithunzi chimenechi?

So you have explained that these are the ones who takes care of you, is there any reason why you took a picture for these people?

02/01: Owoo kungosangalatsidwa kuti ndizimuona

Ok for my brother I told him I want to be seeing him

I: Owoo chabwino chabwino

Ok alright alright

02/01: Ndithu

Sure

I: Owo, kuti muziwaona?

Ok so that you should be seeing him

02/01:Yes

I: Ok

I: Ndiye aaa mwafotokoza zambiri zokhuza chithunzi chimenechi kuti ndi amene amakusamalirani ngakhale ali mwana wa sukulu komabe samakunyozani nanga palinso kufotokoza kwina komwe mungatifotokozere za chithunzi chimenechi zokhuzana moyo umene mumakhala kumene mwachokerako?

So aaa you have explained a lot about this picture that he is the one who takes care of you although he is still in school and he doesn’t let you down, is there anything that you can explain to us about this picture looking on the life that your living from where you are staying?

02/01: Ayi moyo ndilibe kudandaula chifukwa ana anga amandisamala

No there is no complaining in my life because my children takes good care of me.

I: Mmm

Mmm.

02/01: Eee onse amene ndi azikulu ake amandisamala

Yes all of them with his elder brothers they take care of me

I: Mmm

Mmm

02/01: Nde kudandawula ndimakhala ndilibe matendawanso ndinachila (laughs)

So I don’t have any complains am also healed on the disease (laughs)

I: Chabwino chabwino

Alright, alright.

02/01: Ndithu

Sure

I: Kodi nanga chithunzi chimenechi chikupeleka mwayi otani kwaife kuti moyo wanu tithe kuwupitisa patsogolo kudera kumene mwachokerako?

What chances does this picture gives us so that we can improve your life from where you are coming from?

02/01: Aaa umoyo wake ndiwovutikila monga mene mukuyiwona nyumbayo mmene tikukhalira tikhala movutikila malingana kuti ndimagwira ntchito ya umisili koma pena sindimaipeza ntchito mwina ndimaipeza ndiyeno umoyo umakhala ulipo ovutikira koma chifukwa choti ndimthandizidwa ndi anawa chifukwa chake umoyo wanga umakhala ngati ukupitilira ganizo lililonse ndimakhala ndilibe

Aaa we are living a difficult life as you can see the house how we are living its very difficult though I work as a builder but I do get the jobs and some times I don’t, so a difficult life is there but because I do get help from my children thats why I still continue to live without any thoughts.

I: Mmm

Mmm

I: Eee

Yes

I: Chabwino mwati mumagwra ntchito yotani?

Ok what work did you say you do?

02/01: Yomanga

I am a builder.

I: Chabwino chabwino

Alright, alright.

02/01: Ndithu ndimakhala basi nditi ma contract ndikumamanga manyumba

Sure, I do get **contracts** and build houses

I: Mmm

Mmm

02/01: Eee

Yes

I: Chabwino chabwino. Ndiye chithunzi china chimene munasankha kuti tifotokoze timveko ndi chithunzi ichi tafotokozani za chithunzi chimenechi?

Alright, alright. So the other picture that you chose to explain, for us to hear from it is this one, can you explain about this picture?

02/01: Chithunzi chimenechi ndinajambuka choti ngakhale kuti mutafuna kundiyendera mukafika kungokuuzani kuti ooh mufike pamtengo uwoo ndekuti mwandipeza malingana ndiadiresi ndinafotokoza ndimmene anandifunira adokotalawo kuti tingakupezeni bwanji eya atafika pamenepo kungofunsa kuti ooh paja mtengo uwuwu nyumba iyiyo iyi ya a ujeni ndiyekuti mwandipeza

I took this picture so that even if you want to visit me, and they will tell you ooh to reach at that tree, it means you have found me taking it from where the doctor asked me on how they can get me, if they can reach at that tree and just ask at this house, then you have found me

I: Mmm

Mmm

02/01: Eee

Yes

I: Ndi map akunyumba kwanu

It is the **map** to your house?

02/01: Map akunyumba eee

Map to my house yes

I: Chabwino, ndiye pa chithunzi chimenechi chokhuzana ndi map akunyumbawa chikutiwuza zotani zokhuzana ndi moyo umene mumakhala kumene mumachokera?

Alright, now on this picture with the map to your house, what does it tell us concerning with the life that your living from where you are coming from?

02/01: Monga mmene ndikunenera kuti moyo wake siwokuti uli wosangalala ndimmene zikuchitikilamu malinga ndimmene ukuyendelamu ndiye muwone ndi manyumba akewo sakugwilizana ndi pamoyo wanga mene umafunira

As I am saying that my life is not as that happy with the way things are happening looking on the way I am living then you can see the housing, its not matching with the type of the houses I want to have

I: Mmm

Mmm

02/01: Eee

Yes

I: Mumafuna zotani?

What do you want?

02/01: Aaa nyumba yamaonekedwe

Aaah good looking houses

I: (laughing)

(laughing)

02/01: Ndizikhala nyumba ya ujeni iyayi.

I should be staying in just a house, no.

I: Mmm

Mmm

02/01: Eee

Yes

I: Chabwino, chabwino

Alright, alright.

I: Ndiye chithunzichi chikupeleka mwayi otani kwaife kuti moyo wanu tingaupitise bwanji patsogolo

So what chances does this picture has for us to help you improve your life?

02/01: Tsono ife ngati ukupitilira moyo umenewu uzakhala moyo otayika kwambili

If we are still living like this, our life is going to be curse.

I: Nde chithunzi ichocho mukuwona ngati ifeyo tingathe kuchitapo chanikuti moyo wanu upite patsogolo

So what do you think we can do to improve your life from that picture?

02/01: Mukhoza kuwona zimene mungapange kuti kodi titamuthandiza muthuyu tingamuthandize bwanji

You can see how you can do to help this person in what way can we help him

I: Chabwino chabwino

Alright, alright.

02/01: Ndithu

Sure

I: Nanga chithunzi ichi tatifotokozeleni zokhuza chithunzi chimenechi?

What about this picture, can you explain to us about this picture?

02/01: Chithunzi chimenechi mmenemo nditakhala pa admitt ndi amene amandisamala mzukulu wanga ameneyu mwana wa achemwali anga

This picture when I was admitted, he is the one who was taking care of me, he is my sisters son,

I: Owoo

Ok

02/01: Nditakhala ku admitt ndi amene amandithandizila pakalaipanonso ndili naye ndi amene ndikumuphunzitsa ntchito

The time I was admitted he was helping me, and am still staying with him now, I am teaching him how to do the work

I: Mmm

Mmm

02/01: Eee

Yes

I: Mumakhla naye kunyumba konkuno?

Are you staying with him at your house?

02/01: Wangobwera chifukwa choti ndimakhala ndekha ndi ana awiri basi tinalipo atatu nde wina wakwatiwa posachedwa pompa amagwila ntchito ku game ndiye ndasala ndi awiri

He has just come because I stay with two children only, we were three but the other one has just got married soon and she has started working at game, so am remaining with two children

I: Owoo

Ok

02/01: Eya

Yes

I: Chabwino

Alright

I: Ndiye

Now

02/01: Uyu wangobwera moti kumaloko tikayamba mawa kuti tizikagwira ntchitoyo imene inalipoyo koma uyuyu ndi amene amandisamalira nthawi yambiri

This one has just come because and the job has stopped we will start working tomorrow the job that has left, but this one is the one who helps me most of the times

I: Mmm

Mmm

02/01: Eya

Yes

I: Amakhala kuti?

Where does he stay?

I: Amakhala, iyeyu amakhala kuteleku ali mnyumba wabwera mnyumba mwanga kuteleko akachoka kuno ndekuti kuntchitoko kuchoka kuntchitoko ncthito ikazatha mwina azapita kunyumba kumudzi namtenga kuchokera kumudziko mnamuitanitsa.

He stays, he has come in my house, when he goes to work and after finnishing the job, may be its when he will go to the the village because I got him from the village I called him

I: Owoo

Ok

02/01: Eee

Yes

I: Chabwino

Alright

02/01: Ndithu

Sure

I: Nde pachithunzipa amachita chani?

What was he doing on this picture?

02/01: Chithunzi chimenecho basi anangosangalasidwa iyeyo kuti nayenso ndimtenge

On that picture, he was just interested to be taken a picture

I: Mmm

Mmm

02/01: Eee

Yes

I: Tipite kwa ena

We should go to another participant

M: mwina mai kalulu amaziwa kaombedwe kena kammanja

Maybe Mrs kalonga knows another style of clapping hands

MM: Ayi ifeso timawafunsa kuti tiwaombele mmanja mwa mtundu wanji

We also ask them how do you want us to clap hands for you

M: Tiwafunse

Lets ask him

MM: Tikuombeleni mmanja motani

a woman saying how can we clapp hands for you

M: Tikuombeleni mmanja motani dadi, Tikuombeleni mmanja motani?

How do you want us to clap hands for you dad? How should we clap hands for you?

MM: Mwachisawawa mwachifumu mwajombo

Should we clap in any way or the way the clap for the chiefs or as military parade

All: (laughing)

(laughing)

02/01: Ndikupempha mwachisawawa basi

You can clap in any way

MM: Mwachisawawa ndimmene taombamu

Clapping hands in any way is the way how we have done already

02/01: Basi

Its ok

All: (laughing)

(laughing)

SECOND PARTICIPANT

I: Anajambula chithunzi ichi ndindani?

Who took this picture was if you?

04/02: Eya

Yes

I: Chabwino chabwino. Ndiye mwamva mmene adadiwa amafotokozera, ndikufuna mundifotokozele chithunzi chimene mwajambulacho

Alright alright. Now you have heard how the dad has explained, I want you to explain about this picture you have taken.

04/02: Chithunzicho ndinajambula titabwera ngati kuno mmamawa wake anati sindinazuke bwino anazuka akudwala pamenepopo kuti nthupi mwanga sindikupeza bwino nde ndinawajambula kuti ndizikhala ndikuwakumbukila kuti apa mayi angawa sanatani sanazuke bwino eya nkona nawajambula chithunzicho

I took this picture in the morning of the day that we came here he said he was not feeling well he was sick on that picture so that I should be remembering that on this day my mother was sick thats why I took this picture

I: Ndi mai anu

Is she your mother?

04/02: Eee

Yes

I: Chabwino, ndiye chithunzi chimenechi chikutiuza zotani zokhuzana ndi moyo umenemumakhala kumene mwachokelako

Alright, so what does this picture tell us about the life you are living from where you have come from

04/02: Moyo umene timakhala timakhala kuti kukacha tsiku lina munthu amatha kuzuka bwinobwino tsiku lina osazuka bwino ndimmene timatani timakhalira eti

the way we are living here is that some times she woke up well the other day can woke up unwell thats how we live here

I: Mmm, ndiye nanga pali mwayi otani kwa ife kuti moyo wanu tingathe kuwupitisa patsogolo ku kupyolera ku kupyolera mu chithunzi chimene mwajambulachi

Mmm, now what are the chances for us on this picture so that we can improve your life from the picture you have taken?

04/02: Imeneyoyo ndi nthawi yoti ndizizatani ndizizakhalabe ndi kukumbukila ndikumaganizila kuti mai anga amadwala size yake iyiyi koma ineyo ndisaonese kukhumudwa kwina kulikonse

This is the time that I should be remembering that my mother was sick up to this size, but I should not be worried in any way

I: Ndiye pachithunzi mwajambulachi cha mai anu kusonyeza sanadzuke bwino chithunzi chimenechichi chikupeleka mwai otani kwa ifeyo kuti moyo wanu tithe kuupitisa patsogolo

So now on this picture you have taken about your mother that she did not woke up well, what does this picture tell us so that we can improve your life?

04/02: Apopo ndingayankhe kuti tisakhumudwe nazo koma tizikhalabe tikusangalala kuti ndikuona kuti mavuto amunthu ndi anthawi zimachitika

On there I can say that I should not get worried, but I should still be happy that it only happens that problems came come at any time

I: Chabwino chabwino, nanga chithunzi chimenechi tatifotokozelani?

Alright, alright, what about this picture, can you explain to us?

04/02: Pamenepopo ndekuti amalephera kutiazuke nkonano anangogona choncho kuti nthupimu mukuwatani mukuwasowetsa mtendele mukuwapweteka kwambiri

On there she was failing to wake up that’s why she just layed down like that, she was feeling some body pains

I: Mmm

Mmm

I: Ndiye chifukwa chani inuyo munajambula chithunzi chimenechi?

So why did you take this picture?

04/02: Ndinajambula ndikuona ndimmene anazukilamo eyaa, kuti amai sanazuke bwino mmenemumu

I took this picture looking on the way that she woke up yes, that my mother is unwell in this way

I: Ndiuthenga wanji umene inuyo mumafuna kuti muzafotokozele zokhuzana ndimmene amai anu anazukila tsiku limeneli chithunzi chimenechi

What massage did you want to tell us about how your mother woke up this day on this picture?

04/02: Ndi uthenga oti anzanga ndingathe kulimbikisa kuti tisamakhumudwe pamene tikuwaona kuti mwina sanadzuke bwino tsikulo ife tisakhumudwa tisaonese kukhumudwa kwina kulikonse koma ife kwathu kuzikhala chimwemwe chathu

The massage is that I want to incourage my friends that we should not be worried when someone has woke up unwell, we should not be worried but instead we should be happy

I: Chabwino chabwino, nangano chithunzichi chikupeleka mwai otani kwa ife kuti tithe kupitisa moyo wanu patsogolo.

Alright, alright. Now what does this picture give us chances so that we can improve your day to day life

04/02: Izozo pa moyo wa tsiku ndi tsiku ndimmene timatha kukhalila osaona chinthu kusi, kupanga kukhumudwa kapena nditero

in our day to day life, we still stay un worried

I: Mmm

Mmm

04/02: Eee

Yes

I: Chabwino chabwino, nanga chithunzi chimenechi tatifotokozelani, zokhuzana ndi chithunzi chimenechi?

Alright, alright, what about this picture, can you explain on it?

04/02: Apopo chithunzicho timawapatsa chakudya ndiye izo zakhala mbalizo ndizizukulu zimaachita agogo zidyani musakane kutani kudya pamenepo ndithu kunali kuumilizana kuti adye azidya chakudyacho chifukwa pena akamamva kupweteka amatani samakonda kudya ndiye timachita kuti tazidyani pang'ono pang'ono nde pamenepopo

On that picture we were giving her food and those around her are her grand children they were telling her that she should be eating dont refuse to eat, they were forcing her to eat because some times when she is not feeling well she doesn’t like eating, so we usually tell her to be eating little by little thats there

I: Mmm, mwati izo ndizizukulu?

Mmm, you have said those are the grand children?

04/02: Eee

Yes

I: Nazonso zimaathandizira ?

Were they also helping

04/02: Kuti azidya azikhala ngati akuwasangalatsa ndithu kuti aah agogo tiyeni muzitani muzidya

so that she should be eating and entertain her so that she should be eating

I: Mmm, chabwino. Ndiye chifukwa chani inuyo munatijambulila chithunzi chimenechi

Mmm, alright. So why did you take this picture for us?

04/02: Chithunzicho ndinajambula chifukwa chokuti azidya chokudyacho eyaa nde anawoso amabwera pompo kumathandizila kuti azitani azidya

I took this picture so that she should be eatin the food yes , the children were coming around so that she should be eating

I: Nanga uthenga omwe mumafuna kupeleka kwa ife kuchokera muchithunzi chimenechi ndiwotani?now what massage do you want to tell us from this picture?

04/02: Ndimafuna kupeleka uthenga kuti anthu otelewa ayenela kuti azidya

the massage that I wanted to say here is that people like these ones are supposed to be eating

I: Mmm. Chabwino chabwino, chithunzi chimenechi chikutiuza zotani zokhuzana ndi moyo umene mumakhala nthawi zonse?

Mmm. Alright alright, what does this picture tell us about the life that your living every time?

04/02: Chithunzicho chikutiuza moyo oti tikakhala umoyo wamunthu ndikudya

This picture is telling us that when we are living the life of a person is important

I: Mmm

Mmm

04/02: Eyaa

Yes

I: Chabwino, nangano chikupeleka mwai otani kwa ife kuti tithe kupitisa moyo wanu patsogolo

Alright, what chances does this picture give us so that we can improve your life

04/02: Kupitisa moyowu patsogolo zakudyazo ndikukhala kuti ndizidya

Improving life because I will be eating the food

I: Chabwino chabwino, tathokoza. kaya mufuna mmanja motani?

Alright alright, thank you. How do you want us to clap hands for you?

04/02: Mulimonse

In any way

All: (laughing)

(Laughing)

M: mwina asanapitilize kwa ife amene tabwera lero ndimwayi wathuso kuti pamene tichoke pano tikhale ndi chizindikilo cha zomweso tikuyembekezelaso tikabwera lachisanu nafeso tizadusa ndondomeko yomweyi tizabwelesa zithunzi zathu tizafunsidwanso nde zingokukonzekesani kutino mulindi mwayino kuti mwaphunzilakale musanapite azanthuwa anali atango mkoyamba eti?

Maybe before she continues, to those of you that has come here today, its our chance that as we will be living we should have a clue of what we are expecting, we will also do the same process when we come on Friday, we will bring our pictures and we will be asked to explain, this is should just prepare you that you have a chance and you have learn already before going home while our friends here it is their first time, is it?

Ps: Mmm

Mmm

M: Nde tizayembekezera kuti inuyo muzachitanso bwino

So we expect you to perform very good

Ps: Mmm

Mmm

THIRD PARTICIPANT

I: Amene atijambulila chithunzi chimenechi, tatifotokozeleni zokhuza chithunzi chimenechi?

the one who took this picture, can you explain to us about this picture?

05/01: Chithunzi chimenechocho

That picture

M: Mukuona kuchokera kumenekoko?

Are you able to see from there?

05/01: Eee ndikuona

Yes I can see

MS: Owoo chabwino

Ok alright

(Laughing)

(laughing)

05/01: Chithunzi chimenechocho ndinajambula ndine kujambula ka mzukulu kangako eyaa ndi mwana ameneyoyo kamsikana kakhala pamenepoko kamsikana kamenekoko ndikamene kamati kakabwera ku sukulu kubwera kuzatenga kamwanako kumakasewera eya ine ndimangokhala ndekha ndikachizukuluko ndiye ndimaona kuti akantenga mwana uja kupita kosewera ndekuti ineyo ndimapepukidwa zambiri kaya ndikufuna ndigone kaya ndikufuna nditani chifukwa mwana uja amapita kukasewera ndiye katabwera kuzacheza kuzamtenga mwana uja namuuza kuti ndikhoza kukujambula akuti eee ndijambuleni ndinkana ndinamujambula kamtsikanako mwana wa aneba eya amazatenga ka mzukulu kangako kuti azikasewera

I am the one who took that picture of that my little grand child, and that child there, the girl sitting there, it is that girl who when she is back from school she comes and pick my grand child to play, Yes, I stay alone with my grand child and I see that if she picks the child and go to play I become a little bit free and am able to do a lot of things, whether I want to sleep or do other things since the child has gone to play, now when she came to get the child and play, I asked her if I can take her a picture, she said yes you can, that’s why I took a picture of here, she is my neighbors child she came to pick my grand child to play

I: Inuyo mumakhala anthu awiri ndi kamzukulu kanuko?

You stay the two of you with your grand child?

05/01: Ayi ndimakhala ndi ana anga koma amakhala kuti atapiita ku zintchito koma kuti akapita kuzintchito timakhala anthu awiri ndi mzukulu wangayo

No I stay with my children but they all goes to work but if they go I usually stay with my grand child.

I: Chabwino nde uyu ndi mwana wa aneba

Ok, so this one is your neighbors child?

05/01: Eya mwana wa aneba

Yes my neighbors child.

I: Amabwera kuzatenga mwana kumakacheza naye

She comes to pick your child to go and play

05/01: Mmm

Mmm

I: Chabwino, ndiye pamenepopo chimachitika ndichani pachithunzipo

Alright, so what was happening on that picture?

05/01: Pamenepopo chimene chimachitika pomutenga mwanayo ine ndinali osangalala chifukwa akangobwera kusukulu kumutenga mwanayo ineyo ndimakhala ngati ndimamasuka chifukwa choti mwanayo nthawi yoti ndikumva ululu iyeyo amafunaso kuti mwina ndimubeleke kapena akufuna kuti adye ndiye akangibwera kuti ndabwera kuzamutenga mwana amakhalangati kuti mwana uja wanditani wandimasula kuti akupita kukasewera ndiye inechinachilichonse ndimatani ndimakhoza kuchi kamenekota mwanayu atatani atatuluka kupita kukasewera ndiye ndimakhala osangalala chifukwa chakamsikana kamenekoko komene kamandithandizila mwanako

on that picture, when she comes to pick the child I was very happy because when she comes from school I became free because the time that am having body pains, its now when the child wants me to carry her on my back, may be she wants to eat so when she comes and say she want to pick her, she then makes me be free when they are going to play then I can do anything when the child has gone to play, I am happy because of that girl who comes to play with the child

I: mmm

Mmm

05/01: Eee

Yes

I: Chabwino, nde mwatifotokozera kusangalala kwanu kuti akamazamutenga mwana wanu amakupepusani ndiyeno komano chifukwa chani inuyo munaganiza zoti mutijambulire chithunzi chimenechi?

Alright, so you have explained to us how happy you become when she comes to pick your child because she relives you, so why did you think of taking this picture

05/01: Ineyo ndinaganiza kujambula chithunzi chimenechichi kuti tsiku lina ndizizakumbukila kuti ndimadwala matenda a khansa koma podwalapanga kamwana akaka ndikamene kamandithandizila kulera kachizukulu kangaka ndikana ndinajambula chithunzichi

I thought of taking this picture because so that I should be remembering that I was suffering from cancer and by time of my sickness, this girl here was the one who was helping me in taking care of my grandchild thats why I took this picture

I: Mmm

Mmm

05/01: Eee

Yes

I: Chabwno, ndiye kupatula pazomwe mwafotokozazo mmene mumasangalalila iyayi mwanayu akazamutenga mwanayu, palinso zina zimene mungathe kutiuza zokhuzana ndi chithunzi chimenechi zokhuzana ndi moyo umene mumakhala kumene mumakhalako?

Alright, so apart from what you have explained to us that you become happy, when she comes to pick the child, is there anything that you can tell us about this picture about the way you live where you stay?

05/01: Moyo umene ndimakhala kunyumbako umakhala moyo ovuta chifukwa choti ndiochepa amene mwina mwake amabwera kuzatani kuzakhala nane kumacheza nane mazineba ndiochepa amachulukila ndi anawa

I live a difficult life at home because its only a few people who comes to see and chat with me, its only few neighbours who come the ones that comes mostly are these children.

I: Mmm

Mmm

05/01: Eee

Yes

I: Nde chithunzi chimenechi chikupeleka mwai otani kwa ife kuti moyo wanu tithe kuupitisa patsogolo?

So what chances does this picture give us so that we can improve your life?

05/02: Chithunzi chimenechi chikupeleka mwai moyo wanga kuti ndiupitise patsogolo chifukwa kuti ndimmene mukuonera kamwana kangako ndikakang'ono ndipo ndikachizukulu koyamba ineyo ndikamakaona ndimasangalala kuti ndamuoa ndisanatani ndisanasiye dziko lino lapansi ndiyeno ndimasangalala kukaona kamwanako

This picture is is giving chances for my life to improve with the way you can see my grand child is very young and she is my first grand child and am happy when am seeing her because I have seen her when am still alive, so am very glad to see the child

I: Nanga chithunzi chimenechi, tatifotokozeleni?

What about this picture, can you explain it to us?

05/01: Chithunzi chimenechichi ndi mnyumba nditajambula mnyumbamu polingalila ndimmene munalili kale ndimmene mulili pano ndi nyumba yosintha kwambiri ndiye zinandipasa chidwi kuti nditani ndijambule eyaa ndizizaona kuti kale mnyumba mwanga munali motere koma nditafika kuti ndadwala munazasintha munazafika size iyi

This picture its inside the house I took the inside house picture for thinking about the way it was and the way it is now the house has changed a lot, it is very different thats why I decided to take this picture so that I should be remembering the way my house was like this but this is the way it is looking because am sick, it changed up to this size

05/01: Mwina ndingafunseko kuti kusintha kwake kotani?

Maybe I can ask you, what are those changes?

05/01: Chisamaliro chifukwa choti abambo anamwalira 2008 ndimakhala ndi ana ndiyeno anawo inde ayamba timaganyu koma angoyamba kumene komabe akamachoka zimasiyanisa ndikuti mmene ndindalili wamphamvu ndimakhoza kuti mwina pene pake paonongeka kutani kukhonzakhonza eya koma pano olo ndiwone kuti pena pake pawonongeka ndimangosiya kunena kuti ndimmene mwina pakuti pakundipweteka ndingokhala ndingotani ndingogona nde ndimaonabe kuti mnyumba mwanga munasintha

Household care, because my husband died in 2008 and am only staying with my children although they have started working but still more its very different with the way it was when I was strong,I could work on the things when something wrong has happened, but now if I can see somewhere is not ok, I just leave it because I could be feeling some body pains, I would rather just sleep, so for that I see that my house has changed

I: Mmm

Mmm

05/01: Eee

Yes

I: Nde ndiuthenga wanji mungatipatse pokhuzana ndi chithunzi chimenechi?

what message can you give us about this picture?

05/01: Uthenga umene ndingakupaseni ndiwoti ineyo ndikulephera kutani kusamala makhomo panga panopa mmene ndilili ndimmene ndinalili kale chifukwa mmene ndinalilil kale olo abambo anamwalira koma chinachilichonse ndimatani ndimachita geni ndimapang koma zonsezo zinatani ndinasiya chifukwa chachani chamatenda

The message that I can give you is that, I am failing to take care of my house looking on the way I was and now, the way I was in the past although my husband died, I could do everything, I has a bussiness but now I stopped because of the sickness

I: Mmm

Mmm

05/01: Eee

Yes

I: Nde chithunzichi chikupeleka mwayi wotani kwa ife kuti moyo wanu tithe kuwupitisa patsogolo

what chances are there does this picture tell us so that we can improve your life?

05/01: Chithunzi chimenechichi pamenepopo nde (laughs)

On that picture there then, (laughs)

I: Chabwino chabwino, tandifotokozeleni za chithunzi ichi?

Alright, alright, now can you explain about this picture?

05/01: Chithunzi chimenechocho ndi mpongozi wanga amene ali gadiyaniyu amachokera ku msika nde anakhala pakhomo la khitchinipo amasazula nkhwani kuti aphike ineyo nde ndinakhalila chakunoko kuli mkeka chakunoko nde ndinamujambula ali pa khomo la khitchini paja akusazula mkhwani kuti aphike

That is my in-law on the picture who is my guardian, she was coming from the market and she sat on the kitchen door when she was preparing pumpkin leaves so that she should cook and in seated on this side there is a mat, so I took her a picture when she was at the kitchen door preparing pumpkin lives

I: Ndichifukwa chani mujambula chithuzi chimenechi

Why did you take this picture?

05/01: Nde chithunzi chimenechichi ndinajambula chiifukwa choti mpongozi wanga samakhala kunoyi amakhala ku zomba koma ndinachiona kuti ndichamtengo wapatali iyeyu kubwera kuzanditani kuzandidwazika ineyo ndimwana inde koma akulu akulu alipo koma zinakanika kuti atani abwele ndiye ndinaona kuti ndichamtengo wapatali iyeyo kuzipeleka kubwera kwaine kuti anditani andithandize ndimkana ndinajambula chithunzichi

I took this picture because my in-law doesnt stay here she is from Zomba, so I saw it that its something important that she came to take care of me, she is young though the elders are there but they failed to come so I see it very important that she came to take of me that’s why I took her this picture

I: Chabwino, nde chithuunzi chimenechi chikutiuza zotani zokhuzana ndi moyo umene inuyo mumakhala kwanu?

Alright, what does this picture tell us about the life that your living from where your staying?

05/01: Chithunzi chimenechichi chikulongosolabe kuti umoyo wakebe umakhala ovuta ndimmene ndanenela kwandani kwa adzinyemba chifukwa choti ndinganene kuti si azineba onse mmene ndimakhalira nawo poayamba ndiwochepa amene mwinamwake amati kukacha mwazuka bwanji mkubwera kuzanditani kuzandiwona ndebe zimangondipatsabe nkhawa mazineba oti ndili nawo pompano koma kukanika kutani kubwera kuzandiona mwina ukagona kuchipatala muja amakhoza kutenga bausket kupita kukakuona koma pakhomo zikukanika kuti abwere azakutani azakuone nde basino ine zinangondipasa chidwi choti ndijambule chithunzi chimenechichi kuti ndizimkumbukila mpongozi wangayo

This picture is explaining that we are living a difficult life, the way I told Mr (name) its not all the neighbors that we were staying together those that can come to greet and see me, it gives me worries that neighbors that I stay with them they are failing to come and see me, maybe when you are admitted they can get a basket to see you but when you are at home they fail to come to see you, this just gave me a feeling to take this picture

I: Mmm, nangano pachithunzipamenepa chikupeleka mwayi otani kwa ife kuti moyo wanu tithe kuwupititsa patsogolo?

Mmm, what chances does this picture give us so that we can improve your life?

05/01: chithunzi chimenechichi chikupeleka mwayi kwa inuyo kuti mupitise patsogolo ndimmene mukuwonera mmene palili pakhomopo komanso ndimmene ndajambulila chithunzichi kunena kuti pamenepopo inuyo mungapange bwanji kuti moyo wanga upite patsogolo chifukwa nditakhala kunena kuti mphamvu zanga zabwelera mmene zinalili kale ndizosavuta ndine munthu otakataka koma panopa zonse zinatani zinatha koma nditakhala kuti ndachila ndili ndi mphamvu nditha kubweleranso mmene ndimapangila kale

This picture can make you improve my life in any way you think you can do, the way I have taken the picture of my household so that you can see what you can do on that to improve my life because if I can be strong the way I was in the past its not difficult, am a person who does alot of things but everything stopped, but if I can well and strong, I can start doing things the way I was doing in the past

I: Mmm

Mmm

I: Nanga mwanena kuti ndinu munthu otakataka mwin kalelo mumkapanga chani?

you have said that you were doing a lot of things, what were you doing

05/01: Ndimapanga business

I was doing a **business**

I: business yanji?

What business?

05/01: Ndinali ndi okala pati pakhomo lampanda koma ndinatseka

I had a hawker at the entrance of the fence but I closed

I: mmm, tathokoza kwambiri. Aaa tiombe mmanja motani?

Mmm, We are very thankful. Aaa how do you want us to clap hands for you?

05/01: Mwachifumu

For the chiefs

All: (clapping hands, laughing)

(clapping hands, laughing)

FOURTH PARTiCiPANT

I: Tandifotokozeleni zokhuzana ndi chithunzi chimenechi ichi

can you explain to me about this picture

MS: fotokozani mmene mungakwanitsile

you can explain in any way you can

09/02: Chabwino

Alright

09/02: Ndingoyamba ndichonchi

let me just start like this

I: (laughs))

(Laughs)

09/02: Chithunzi chimenechocho kuti ndichijambule ndikufotokozerani motere kuti iyeyu nd mzukuluu nde iyeyo amadwala kapena zifike kumeneko?

for me to take on this picture, let me explain to you like this, this one is my grandchild she was sick, should I say on that?

I: Eee fotokozani ndithu

Yes you can explain

09/02: (laughs)

(laughs)

09/02: Amadwala nde kudwala kwachekono nde ineyo ndinasangalala nako kunena kuti ndimutani ndimujambule

She was sick, so with her sickness then I was happy with that to take her a picture

I: Mmm

Mmm

09/02: Eee

Yes

I: Munasangalala ndikudwala kwakeko?

Were you happy with her sickness?

09/02: Ndinasangalala ineyo kuti nndimujambule

I was happy to take her a picture

I: Mmm, chabwino chikuchitika ndichani pachithunzipo? Mnangoyima kapena chilipo chimene amachita chinachake?

Mmm, alright what is happening on the picture? Did she just stood up or was there something that she was doing?

09/02: Ndinamuuza kunena kuti tayima ndikujambule eya nde iye anngoima kuti agogo ndijambuleni nde ndinamujambula

I told her to stand up so that I should take a picture, and she said you can take a picture and I took her a picture

I: Mmm

Mmm

09/02: Eee

Yes

I: Nde chimene chinakupangisani kuti mujambule chithunzi chimenechi ndichani?

So what made you think of taking this picture?

09/02: Ineyo zinandisangalasa kuti ndimujjambule ndimmene iyeyu alili

I was just happy to take her a picture because of the way she is.

I: Mukati mmene iyeyu alili mukati alili mukunthauza kuti chani?

What do you mean when you say the way she is?

M: fotokozani amadwala kuti wachila kapena

M: Can you explain, was she sick and now that she is fine or else

09/02: Anadwala nde pamenepopo wachila

She was sick and now she is ok

I: Mmm

Mmm

09/02: Eee

Yes

I: Amadwala chani?

What was she suffering from?

09/02: Ameneyu amadwala zotupa komanso anazadwala mmimba koma amatsegulaso

She had a swelling and she was sick from stomach disease and diarrhea.

I: Mmm

Mmm

09/02: Eyaa

Yes

I: Owo ndipeshenti wanu?

Ok, is she your your patient?

09/02: Eee ndipeshenti

Yes she is a patient

I: Ooh ndipeshenti wanu uyuyu?

ooh is she your patient?

09/02: Eee ndipeshenti

Yes she is a patient

I: ooh inuyo ndi gadiyani?

ooh are you a guardian?

09/02: Eya patsikulimenelo ndinali gadiyani

Yes on that day I was a guardian

I: Ooh chabwino, nde chithunzi chimeenechi chikutiwuza zotani zokhuzana ndi moyo umene mumakhala?

what does this picture tell us about the life that you are living?

09/02: Chithunzi chimenecho chimandiuza zoti mmene timakhalila pakhomopo timakhala motani movutikila ndiye zikutiuza choncho pachithunzi chimenecho kuti kuti tizijambule

This picture is telling me about how we are living at our home that we live very difficult so thats why I took this picture because its telling us that.

I: Mungandifotokozeleko pang'ono kuti mumakhala movutikila motani?

Can you explain to us that what difficulties are they

09/02: Ndimmene timakhalila pakhomopo kumbali zina ndizina zoti zitithandize ifeso pamoyo wathu

That is the way we are living at our house in terms of other things that can help us in life

I: Mmm

Mmm

09/02: Eee

Yes

I: Nde chithunzichi chikupeleka mwayi otani kwa ife kuti moyo wanu tithe kuwupitisa patsogolo

So what chances are there on this picture so that we can improve your life?

09/02: Chithunzichi chikupeleka pamoyo wanu kuti mwinamwache mutha kumaliza inuyo kutsogoloko chikupeleka mwayi onena kuti ununso mumve pakati pachithunzi chimenecho kutiatilongosolera bwanji

This picture is giving your life so that maybe, you can continue it is giving the chances that you should also understand on this picture that how have I explained to you

I: Mmm

Mmm

09/02: Eee

Yes

I: Chabwino chabwino, nanga chimenechi mungatifotokozeleko?

Alright, alright, what about this one, can you explain to us?

09/02: Chithunzi chimenecho ndimasangalala nacho pomujambula mwanayo ndimmene iye akukhalila malingana mkunena kuti pachithunzipotu bambo wake wamwalira ndiye akukhala motani movutikila ndimmene iye wakhalila komaso anadwalakoso matenda a TB ndiye anamaliza

I was happy to take this picture on the child with the way she is living since her father died and she is living very difficult life and the way she is has stayed and also she suffered from tuberculosis but she finished her dosage

I: Ndindani wanu ameneyu?

Who is she to you?

09/02: Chidzukulu

My grandchild

I: Chidzukulu, mumakhala nacho limodzi?

Grandchild, do you stay together?

09/02: Eee

Yes

I: Ndiye chifukwa chani munajambula chithunzi chimenechi?

So why did you take this picture?

09/02: Chithunzi chimenechi ndinajambula pondisangalasa kuti ndimujambule mwana uja kuti kunokoso afike mumuone kuti ndajambula mwana zimene ndakambila ine

I was very happy to take this picture of the child so that you should also see her, so that I have taken a picture of this child on what I have explained

I: Mmm, nangano pankhani yokhuzana ndi matenda munthu amene mumamusamalira amene akudwala matenda a khansa ndi chithunzi ichi mungatifotokozele zotani?

Mmm, now on the issue of the person you are taking care of, the one who is suffering from cancer with this picture, what can you explain to us?

09/02: Tayambilani?

Come again?

I: Ndimafotokoza zokuti inuyo mumsamalira muthuu amene alindi matenda a khansa panyumba panu ndiye ndikukhalanso ndi mwana uyuy mungatifotokozele zotani zokhuzana ndi mmene unuyo moyo wanu umakhalila komanso mwanayu komanso matenda amene mulinawo pakhomo panu?

I was saying that you are taking care of a person who is suffering from cancer at your house, now staying with this child, what can you explain to us in terms of the life that you are living with the child and also the person suffering at your house?

09/01:: Matenda akhansa ndimmene amakhalila mwanayu matenda ake ndiyambira kunena kunena kuti munthu odwala matenda akhansa chisamaliro ndimachisamalira kwambiri ndipo panalibenso vuto linalililonse komanso peshentiyu amamva kukoma zimene ndimamupangira ineyo panalibe vuto linalililonse ngati amamva kupweteka amamva kupweteka poti ndithupi koma chisamaliro cha munthiu odwala metenda amenewowo moyo wake amamva kukoma

With the cancer disease and the way this child is suffering, I will start with saying that a person who is suffering from cancer, I was taking a very good care and there was no any problem and also the patient was feeling very happy with what I was doing for him, there was no any problem, if he was feeling pains it was because its the body that was paining, but when I was caring this person, he was feeling very happy.

I: Anachira?

Is well now?

09/01: Eee

Yes

I: Owoo (laughs) chabwino. Mumawajambula aja ndi iwowo?

Ok (laughs) Alright. Is he the one you took a picture of?

09/02: Ayi amenewo sindinawajambule amenewoyi

No I havent taken him any picture

I: Ndiye nanga chithunzi ichi mungatifotokozele zotani?

So, how can you explain to us about this picture?

09/02: Chithunzichimenechi ndingakufotokozeleni kunena kuti ndinakodwelesedwa kumujambula mwana ameneuja malingana ndikuti mmene mukuonera kakhalidwe mmene timakhalira panyumbapo zambiri zimakhala zopelewera ndimmene iye ana amene ndajambula pamenepo ndiye amakhal;a akumadandaula nthawi zonse komapo anadwalapo motelomu tinapita kuchipatala kukamuona ndiye ananena kuti mwanayu timudikile akule kuti tizaone kuti alibwanji mwina atha kutani kuyambapo zathu zimene timapangazi nde kuchipatala sindinapite naye koma akukukulabe choncho ali ndi zaka 10

what I can explain on this picture is that I was just happy to take a picture of this child with the reason that as you can see the way we are living at our home, a lot of things are not sufficient with those children I have taken there pictures, she always get worried but she was sick in the past we went to the hospital and they said we should wait for her to grow a little bit so that they should observe how she is so that she can start what we does, I haven’t gone to the hospital yet but she is growing like this she is now 10 years old.

I: Mmm

Mmm

09/02: Mmm

Mmm

I: Mukati zathu zimene timapangazi

if you say what we does

09/02: (laughs) Ndimasuketu

(laughs) Should I be open

I: (laughs) Pamenepotu

(laughs) I think so there.

09/02: Mankhwala thu amene timamwa ama ARV

Our medicine that we take the ARV's

I: Ooh

Ok

09/02: Eya

Yes

I: Chabwino chabwino, nde inuyo aah pachithunzichi chimachitika ndichani amatani mwanayu?

Alright, alright, now you aah what was happening on this picture, what was the child doing?

09/02: Mutanthauza mmene wayimira?

You mean the way she is standing?

I: Eee, chilipo chimene amapanga?

Yes, is there anything she was doing?

09/02: Anangopanga chonchi ndikumuchita kuti panga chonchi manja akuti iyayi agogo ndipanga chonchi ndijambuleni

She just did like this and I was telling her do your hands like this, and she said no granny I will do like this just take a picture

I: Ooh

Ok

09/02: Nde ndinamufunsa kuti ukapanga chonchi iweyo ukutanthauza kuti chani? Anandiuza kuti palibe

And I asked her what do you mean when you do like that? She told me that nothing.

I: Chabwino, nangano chithunzi chimenechi chili ndi uthenga otani kwa ifeyo zokhuzana ndi umoyo wanu mmene mumakhalira

Alright, what massage does this picture have for us about the life that you are live.?

09/02: Chithunzi chimenechi uthenga umene ndingakupaseni uthenga umenewowo mutaunyamule ndinu pochiona chithunzi chimenechi

The massage that I can tell you from this picture is, you are the one who is going to get the massage on seeing the picture.

I: Mmm

Mmm

09/02: Mmm

Mmm

I: Uthenga wake otani?

What kind of massage?

09/02: Muuziwa inuyo ineyo sindikwanisayi

You will know it, I can’t manage

I: (laughs)

(laughs)

09/02: Ndimmene waimilamo

With the way you have stood up

I: (laughs) Chabwino nangano ndimwayi otani kwaife woti aah kupititsa pasogolo umoyo wanu polingana ndi chithunzi chimenechi?

(laughs) Chabwino now what are the chances for us so that we can improve your life pertaining to this picture?

09/02: Titengelapo mwayi woti tipitise patsogolo patsogolopo ife sitingakwanise pasogolopo mwayi umene tingatenge woti tipite nawo pasologolo mutatitsogoze ndinuyo kuti tipite nawo patsogolopo

we will have the chance that we can improve, in the future we can not afford, for the future we can not afford you are the one who can lead us to improve so that we can improve

I: Inu mukufuna tikutsogozeni pa mwai wotani?

what chances do you want us to lead you on?

09/02: Aa mwayi wakuyankhula pamawu oyankhula pachithunzi chimenecho mmene mwachioneramo

Aaa, the chance to talk about the picture the way you have seen it.

I: Mmm

Mmm

09/02: Eee

Yes

I: Zoyankhula zake ziti?

What are those words?

09/02: Eee?

What?

I: Ndikufuna zolankhulazo zomwe zikulankhulidwa pachithunzio

I want what is being said on the picture

09/02: Tsono ineyo sindingathe kuyankhula inuyo ndamene mutayankhule pachithunzicho mmene mwachionera

I can not manage to talk, but you should be the one to explain on the picture the way you have seen it

I: (laughs) Inuyo mmene mumajambula koma mumaona kuti mumaziwa kuti aah ichichi chikutanthauza chakuti nde tikufuna ife tikufuna tikhale nawo pamene mumajambulapo

The time you were taking this picture, you knew that aaa this is what it means so we want to be like where you were taking the picture

09/02: Eee

Yes

I: Eee

Yes

09/02: Nde pamene ndimajambulapo ndakuuzani kuti ndachijambula malingana ndimmene mwana wayimira ndiyeno pa umoyo umene mukunena kuti mungatithandize bwanji mungatiuze zotani nde ine sindingathe kufotokoza mutamalize ndinu mmene wayimila iyeyu mwana

So the time I was taking this picture I have already told you that I did take this picture because of the way this child was standing, now on the way you are saying on how you can help us, what you can tell us I can not manage to say anything but you are the one who will finish looking at the way the child is standing.

I: Mmm

Mmm

09/02? Eee

Yes

09/02: Mwana ameneyu wayima bwanji?

How is this child standing up?

I: Mmm, chabwino chabwino, tathokoza. Kaya mmanja mwake motani?

Mmm, alright alright, thank you. How can we clap hands for you?

09/02: Aah mulimmonse

Aah in any way

All: (clapping hands)

(clapping hands)

FIFTH PARTICIPANT

I: Chithunzi chimenechi tatifotokozeleni nkhani yake?

on this picture, can you explain to us about the story?

06/02: Pamenepo ndi mai anga amene anandibeleka ine

that one is my mother who gave birth to me.

I: Mmm

Mmm

06/02: Uyo ndi mwana wanga

That one is my child.

I: Mutangokwezako mau pang'ono

can you raise your voice a little bit.

06/02: Awo ndi mai anga amene anandibeleka ine uyo ndimwana wanga ngati chidzukulu chawo eti

that one is my mother the one who gave birth to me, that one on the side is my child, like her grandchild

I: Eee

Yes

06/01: Eee

Yes

I: Ndiye chimachitika ndichani pachithunzi chimenechi?

What was happening on the picture?

06/02: Chithunzi chimenecho amaiwo amakana kuti ndiwajambule koma ineyo ndinawauza kuti inuyo mulibe chithunzi chinachilichonse mnyumba muno kuti tizikhala tikukuonani nde ine ndikujambulani olo mukane koma ine ndikutanini ndikujambulani kuti tsiku l inandizizakukumbukilani

on that picture, my mother was refusing to be taken a picture but I told her that she doesnt have any picture in this house, “ so that we can be seeing you, I am going to take a picture of you whether you refuse but am going to take a picture of you so that one day we should be remembering you,” thats why I took this picture.

06/02: mm

Mmm

I: Chabwimo. Nde pankhani yokhuzana ndi matenda omwe ali mnyumba mwanu mene mumawasamalira mungatifotokozele zotani zokhuzana ndi chithunzi chimenechi?

Alright. Now on the issue of the person suffering in your house the one you takes care of, what can you explain about this picture?

06/02: Mai anga amenewa amakhala akuwadandaula akazi anga malingana ndi miyendo yawoyi ndiye amakhalanso akundilimbikisa kuti mmene zililimu akazi anuwa ndimakhala ndikupanga chisoni komabe nanga si nyumba imene mukhalayo ofunika mutapeza malo ndingakhale okondwera kwambiri nde ndati aah tikazapeza ndalama tizagula malowo tizamanga nyumba chifukwa amandilimbikisa zimenezo

This is my mother, she always complain about my wife with the way her legs are, and she always encourage me with the way things are, I am the who work for your wife but still the way you are living you need to find your own place if you have money, so I said if we are going to have money we will buy our own place and build our house, that’s what she always encourages me.

I: Mmm

Mmm

06/02: Eee

Yes

I: Amadandaula kuti nyumba imene mumakhala

She gets worried with the house that you are live in?

06/02: Eee nyumba imene ndimakhalayo komanso ujeni matenda a akazi angawa cancer imene akudwala

Yes the house that we are living in, and again with the disease my wife is suffering from, cancer.

I: Amadandaula kuti nyumba imene mumakhalayo yatani?

what makes her get worried with the house you are living in?

06/02: Aah siyabwinoyi

Aah it not a good one.

I: Mmm

Mmm

06/02: Eee

Yes

I: Chabwino, ndiye pali uthenga otani umene mwatitengera kuti mutiuze okhuzana ndi chithunzi chimenechi?

Alright, what massage do you have for us that you want to tell us about this picture?

06/02: Chithunzi chimenechi ineyo ndimakondwera kwambiri kundiuza za madamu angawa zamatenda amene akudwalawa amandiuza kuti muziwasamalira bwino bwino musamawakhumudwise ndiye ndimaona kuti amandilangiza mau abwino ndichifukwa chake ndawajambula

On this picture, am always happy when am told about my wife with the disease she is suffering from, they tell me that I should be taking care of her so that she should not be worried, I see to it that they give me good advices that’s why I have taken the picture

I: Chabwino chabwino, ndiye chithunzi chimenechi chikutiuza zotani zokhuzana ndi chikupeleka mwayi wotani kwaife kuti moyo wanu tithe kuupitisa patsogolo?

Alright, alright, now what does this picture tell us about, what chances is it giving us so that we can improve your life

06/02: Chithunzi chimenechi ndawajambula maiwa chifukwa choti amakonda kudandaula za apongozi awowa ndichifukwa chake ndawajambula

I took this picture of my mother because she always get worried about her daughter in-law, thats why I took her a picture

I: Mmm

Mmm

06/02: Eee

Yes

06/02: Nthawi zonse ndikamapita kukawaona amakhala akundiuza za apongozi awo

every time when I go to see her, she always tells me about her in-law

I: Mmm

Mmm

06/02: Ndithu

Sure

I: Chabwino chabwino, nanga chithunzi chimenechi

Alright alright, What about this picture?

06/02: Pamenepo nthawi zina ndikamachoka kuganyu ndimawapeza atakhala ndithu panyumba pathu atakhala chonchi akudandaula ndendimawauza kuti mai musadandaule muzikhala omasuka ndithu

On that picture, most of the times when am coming from doing peace works, I found her sitting like that at our house very worried so I was telling her that she should not be worried, be at ease

I: Mmmm

Mmmm

06/02: Eee

yes

I: Mwati bwanji?

what were you saying?

06/02: Ndimawauza kuti mai musadandaule muzikhala omasuka ndiye akuti koma miyendo yanga ikundipweteka pepani komabe musamadandaule kwambiri muzakhala bwino

I was telling her that she should not be worried then she was saying that her legs are paining, I said sorry but do not be worried too much one day you are going to be fine

I: Mmmm

Mmmm

06/02: Eee

yes

I: Chabwino chabwino

Alright alright

06/02: Uyo ndimwana wanga uja ameneyo

That one is my child

I: Mmmm

Mmmm

Nde ndichifukwa chani munaganiza zoti mutijambulire chithunzi chimenechi kuti tizachione?

Now why did you think of taking this picture so that we should see it?

06/02: Aaah chimene ndinaganiza kuti ndiwajambure ndimafuna tsiku lina ngati kulikotheka tizizakhalabe tikuwaona ndikukumbukila kuti akazi anga anadwalaa chonchi

Aaah I thought of taking this picture so that some time we should be seeing it and remember that my wife got sick up to this extent

I: Mmmm

Mmmm

06/02: Eee

Yes

I: Chabwino

Alright

06/02: Ndithu

Sure

I: Nangano chithunzi chimenechi chikutiuza zotani zokhuuza ndi moyo umene mumakhala?

So what is this picture telling us about the life that you are living?

06/02: Aaah moyo umene ndimakhala ndimakhala ovutika kwambiri panthawi imene analibwino iwowa amakhala akuthamanga thamanga kupanga timabizinesi ineso ndikuthamanga mbali inayi zimakhala bwino bwino koma mmene apezeka ndi matenda awawa sakutha kumathamanga monga mwakale

Aaah we are living in a difficult life, by the time she was well, she was doing alot of things, doing businesses while I was also doing other things, we were living ok but now after she was found with this disease, she is not doing anything like she used to do before

I: Mmmm

Mmmm

06/02: Eee

Yes

I: Nangano ndimwayi wotani kwaife kuti moyo wanu tithe kuupitisa patsogolo kupyolera muchithunzi chimenechi?

What are the chances to us so that we can improve your life through this picture?

06/02: Aaah ine ndingakhale okondwa kwambiri madamu angawa atakhala atachira atamwetsedwa mankhwala ndikuchila kubwelera mwakale ndizakhala osangalala kwambiri chifukwa akamandiuza kuti miyendo ikupweteka kwambiri moyo wanga nanenso sindimapuma bwino bwino ngakhale chakudya chimene sindimadya chifukwa choti anzanga sakupeza bwino olo chakudya chimene iwowanso timachita kuwakakamiza nde banja lathu sitimakhala osangalalayi mpakapano

Aaah I can be very happy if my wife can be healed, if she can be given medicine and get healed I will be very happy because when she is telling me that her legs are paining very much, I also dont feel ok, even the food I fail to eat because my wife is not feeling ok, we even force her to eat, so with that we are not happy in our family

I: Mmmm

Mmmm

I: Chabwino chabwino

Alright alright

Anthu kuyankhula chapansipansi

People talking in the background and laughing

I: Nanga chithunzi chimenechi mungatifotokozele zotani?

What about this picture, what can you explain to us?

06/02: Uyu wanyamula mwanayu ndi mphwanga ujeni anatisiya ndi uyu wamng'ono uyu msikana winayu ndi ujeni mwana wamphwangaso ndingoti asikana aawiriwo ndiana amphwanga malemu anandisiya ndiamene ndikuwasamalila panopa makamaka uyonso nde alindivuto amakonda kudwaladwala wamng'onoyo wagwira chonchiyu ndimakhala ndikudandaula kwambiri akamadwala uyo ndimwana wa achemwali anga amunyamulayo wamng'ono kwambiriyu uyo nde mwamuziwa kale

That one who has carried a baby is my young brother but her wife passed on, and the other one this one that girl, is the child of my young brother or I can just say those two girls are my brother’s daughters, my brother died so we are the one taking care of them, mostly that has a problem she gets sick every time that one who has hold hands like this, I gets worried when she is sick, that one is my sisters child who has been carried, you have known her already right?

I: Wanuyo

Yours

06/02: Eee

Yes

I: Ndithu ndithu

Alright alright

Ndiye pamenepo chimachtika ndichani?

Now what was happening here?

06/02: Pamenepo ndinawauza kuti aah inu takhalani apa ndikujambuleni chifukwa pikitcha yanu inailiyonse ine ndilibe komanso ngakhale mphwanga mmene analipo ndinalibe maganizo woti ndimujambule panopo olo pikitcha yake ndillibe kenako ndikuti ineyo mwina sindimaganiza bwino kulibwino ndiwajambule anawa kuti ndikamawaona anawa ndimaona mphwanga eeh ndimaona mphwanga

I just told them that aaah you can you sit there so that I should take a picture of them because we dont have any picture and also when my young brother was alive, I did not think of taking a picture of him, I dont have his picture now, then I said I was not thinking alright but now let me take a picture of these children because when am seeing his children, I see my brother eeh I see my brother

I: Mmm

Mmm

Nanga pali nkhani inayililonse yomwe mungatifotokozele yokhuzana ndi chithunzi chimenechi ana mwajambulawa ndimatenda amene mulinawo pakhomo panu?

Now is there story that you can tell us about this picture, the photo of these children and the person who is suffering in your house?

06/02 Eee

Yes

Mzimai kuyankhula chapansipansi kuti ndili pafupi ku ndirande

A woman talking in the background saying I am near in ndirande

06/02: Makamaka uyuyu wamng'onoyu ndiamene alindi vuto kwambiri sipatha mwezi kudwala amadwala pafupi pafupi ndimakhala ndinkhawa kwambiri komanso ana amenewowo akulaa ndi agogo awo anawasiya ali khanda kwambiri

Mostly this young one is the one who has a problem, because a month can not pass without her getting sick, so am very worried, but also these children have been raised by there grand mother because they left them when they were very young

I: Mmmm

Mmmm

I: Agogo awo ndi

Their grandmother is

06/02: Mai anga aja

My mother

I: Oooh mai anu aja

Ok your mother

06/02: Eee

Yes

I: Mmmm

Mmmm

Chabwinoo

Alright

I: Nanga chithunzi chimenechi chikutiuza zotani zokhuzana ndi moyo umene mumakhala

What is this picture telling us about the life that you are living

Munthu kuyankhula chapansipansi

Someone talking in the background

06/02: Aaah moyo umene timakhala timakhalabe movutikabe kwambiri chifukwa chonena kuti ana awawa anawasiya ali ang'ono bambo wawo nde timakhala movutikila nangasi makolo palibe kuyambira mai awo bambo awo onse anatisiya nde ana amenewa palibe pothawirayi ngati kuli kothawira nde ndikwaifeyo basi

Aaah the life that we are living is very difficult because the father of these children died when they were very young then we live very difficult as their parents are not alive, their mother and father all died so these children have no where to go, if they have where to go its only to us

I: Chabwino chabwino

Alright alright

Nangano chithunzichi chikupeleka mwayi wotani kwaife kuti moyo wanu tithe kuupitisa patsogolo

What chances does this picture have for us to improve your life

06/02: Aaah chithunzi chimenechi ndingakhale okondwa kuti ana amenewa makamaka uyu wamng'ono amadwaladwala ameneyoyo atakhala kuti sakudwaladwala ndikhoza kukhala osangalala kwambiri ameneyi sipatha mwezi kudwala

Aaah this picture, I can be happy mostly to this young one who usually gets sick, so that if she could not be suffering now and again I can be very happy because she a month cant pass without getting sick

I: Nde kudwaladwala kwa iyeyu zimakukhuzani bwanji inuyo pokhuzana ndimatenda amene inuyo mulinawo panopa

How does her sickness concern you considering the sick person you have right now?

06/02: Ineyo zimandikhuza kwambiri ndikaona awa akunyumbawa matenda aaawo ndikuzaonaso ameneyuso pafupipafupi amandiuza kuti mwana uja sakupeza bwino ndimakhala ndinkhawa kwambiri nanga si mai ajaso sangathe kupita naye kuchipatala mmene mukuwaonera muja

It concerns me a lot because when I see my sick wife and seeing this one and they tell me that the child is not feeling well I become very worried, because my mother can not manage to go with her to the hospital the way you have seen her

I: Chabwino chabwino. Ichinso sichanu?

Alright alright. This is not yours aswell?

06/02: Ayi sichawo

No its not he's

I: Oooh tamaliza eti, ndiye mmanja motani

Oooh we are now finished right, how should we clap hands?

06/02: Aaah mmanja mulimonse basi

Aaah you can clap hands in any way

SIXTH PARTICIPANT

I: Tifotokozeleni za chithunzi ichi

Explain to us about this picture

03/02: Chithunzi chimenechichi ndinajambula friday last week umomo ndi mnyumba mwa sister wanga amene amene ndi mwanaso wa ayiwa nde tsiku limenelo friday last week nditafika amayiwa ananena kuti tapangani zoti mundiyikile filimu yomwe ndimayikonda ija ya zee world kuti atleast mwina penapake ndiziyiwala mavuto ena ndi ena nde apopo akuwonera filimu amayikonda ya zee world

I took this picture on **Friday** **last week**, this is the inside of my sisters house she is very young, now on that day last week when I came back home, they told me that I should put a film they like to watch, **zee world** so that **at least** may be I should be forgetting other problems, now on there they are watching **zee world**

I: Mmmm, kunyumba kwa sister wanu?

Mmm, at your **sisters** house?

03/02: Eya mnyumba mwa sister

Yes at my **sisters** house.

I: Chabwino chabwino

Alright alright.

I: Nde chifukwa chani inuyo munaganiza zokuti mutijambulire chithunzi chimenechi

So why did you think of taking this picture?

03/02: Aah chithunzi chimenechi ndinajambula kuti kutsogoloku ndizizakumbukira

Aah I took this picture so that in the future we should be remembering.

I: Mmmm

Mmmm

03/02: Eee

Yes

03/02: Tizizauza zizukulu kuti nthawi inayake amayiwa anali ndi favouright filimu yawo imene amayikonda chanel cha zee world

when we will e telling grandchildren that this woman had her favouright movie chanel she liked the most, the zee world

I: Mmm, nanga ndi uthenga wanji umene mungatipatse?

Mmm, what massage can you give us?

03/02: Uthenga umene ndingapeleke ndionena kuti penepake munthu ngakhale ali ndi vuto mthupi akapempha chinnthu penapake kuti mwina asangalase maso ndibwino kuti mpata ngati umenewo kumpatsa chifukwa choti amatha mwina kumaiwala mavuto kuti mene akuwonera filimu ija amakhala mwina akuona zinazake zosangalatsa zoti kaya nthupi penapake pakuvutavuta amakhala kuti waiwala zoti nthupi mwavuta concetration yawo kumangokhala pa zosangalatsa zija

The massage that I can give is that sometimes when a person has a health problem and has asked for something to entertain the eyes its good to give them that space because sometimes they forget about the problems that they have, the time they are watching the movie, they see something interesting and somewhere they can forget that they are sick because the concentration is on the entertainment.

I: Nangano chithunzi chimenechichi mungatiuze zotani zokhuzana ndi moyo umene inuyo mumakhala?

So what can this picture tell us about the life that you are living now?

03/02: Mmene timakhala mnyumba iyiyi mai kawiri kawiri amakonda kuti mmawa uja akamadzuka mwina kudandaula za nthupi ndebe ifebe timayesesabe kuti mwinamwake tiwasangalase

When we were staying in this house, almost every morning when she would woke up complaining that she is not feeling well and we try to make her happy

I: Nangano ndimwayi otani kwaife wokuti titha kupitisa patsogolo moyo wanu kupyolersa muchithunzi chimenechi?

So what are the chances to us so that we can improve your life through this picture?

03/02: Apopo ifeyo nzoti nangasi umomo ndi mnyumba ngati mwa sister mnyumba mwawo kutsogolo kuno mwinaa atazakhalaso ndizinthu zawo zoti mwina zawozawo zoti azisangalala chifukwa choti mwina kupezeka kuti asisiwa mwina asamuka mwina apita kwina ndekuti iwowo azakhalano zinthu zowasangalasa kuti aziyiwala matenda awowa zizasowa amakaoneramo filimu.

Since that is my sisters house where she just watches a movie, now we also want that in the future should also have things like these ones for entertainment, because maybe my sister can move out, then maybe she will have nothing to entertain her to forget about her sickness

(interruptions)

I: Nanga tifotokozeleni za chithunzi ichi?

Now explain about this picture?

03/02: Chithunzi ichichi linali saturday mammawa nditangodzuka ndiye anati kuti ndiwajambule uku akutsuka mbale kusonyeza kuti tsiku limenelo kutisonyezera ifeyo kuti tsiku limenelo atleast adzukako ndi mphamvu

This picture, was on Saturday morning soon after waking up, so she said that I should take a picture or her while washing the plates, meaning she at-least woke up strong that day.

I: Mmm, ndiye chifukwa chani inuyo munaganiza zoti mujambulile chithunzi chimenechi?

Mmm, now why did you think of taking this picture?

03/02: Ineyo atandipempha ndianafuna kuti ndikwanilise kuti ndiwajambule kuti nawonso ndiwasonyezere kuti nafeso kuti tiwasonyezere kuti nafeso tagwirizana nazo kuti tsiku limenelolo adzukako bwino chifukwa nangasi mwina akamadzuka amapezeka kuti ali chigonere komaq tinangodzidzimuka saturdaylo kuti akuti iyayi pasapezeke munthu otsuka mbale ndigwira ndekha nde tsiku limeneli tizizakumbukila kuti tsiku ililokha anatipempha kuti agwire ntchito zapakhomo okha popanda munthu kuwathandiza

When she asked me, I wanted to fulfill by taking a picture of her so that I should show her that we have agreed that on that day she woke up a bit well, because every time when she is waking up, she still remain in bed but we just wondered on **Saturday** when she said, no one should clean the plates I will clean myself, now on this we are going to remember this day that she asked us to do household chores without anyone help

I: Mmm, chabwino. Nangano chithunzi chimenechi chikutiwuza zotani zokhuzana ndi moyo umene mumakhala ?

Mmm, alright. What does this picture tell us about the life that your living?

03/02: Chithunzi ichichi chikutiuza moyo okuti ngakhale munthu anali ndi vuto linalake komabe ngati tsiku linalake mwamphamvu yamulungu kuti munthuyu wadzukabe kaya ndimphamvu athabe kumakumbutsa chikale kuti kalekalelija ndili ndi mphamvu ndimatsukabe mbale ndiyebe lelolo nditimphamvu ndadzuka natoti ndikumbukire chikale kuti ndimatsuka motere kaya kusesa kaya kutani

This picture is telling us that even if a person has a problem, maybe in one day with the power of God and the person has wake up a little bit ok, they can be doing things that they were doing when they were not sick like cleaning plates, that today I have wake up strong, I should remember in the past that we were cleaning plates like this, whether sweeping or doing other things.

I: Chabwino chabwino. Nangano chithunzi chimenechi chikupeleka mwayi wotani kwa ife kuti moyo wanu tithe kuupitisa patsogolo?

Alright, alright. So what chances are there on this picture so that we can improve your life?

03/02: Apopo ifeyo tinakakondwera kuti azachipatala atapitiliza mwina kumawathandiza amayiwo ndi mankhwala kuti apitilize moyo wathanzi kuti moyo wathanzi upitilile kuti ngakhale penabe zimavuta kuti kutenga mwina kwa one week ali chigonelebe komabe penabe umapezeka kuti adzukabe bwino nde azacipatala mutapitilizabe kutithandiza kuti mankhwala amene amalandilawa azithabe kumagwilabe ntchito zinanzina

on that we can be happy if health personnel can continue helping and giving her medicine so that she can continue to live a health life, so that her health life should continue though sometimes she stay for a week while not feeling well but still more sometimes she wakes up well, so if the health personnel can continue to help us that so that the medicine she is receiving should be continue be working.

I: Chabwino chabwino. Nanga chithunzi chimenechi

Alright, alright. What about this picture

03/02: Chithunzi ichichi sinduchimvetsa bwino bwino pakhomopo pali maluwa nde dzulo anayeserakoso kudzuka bwino nde mpameneno amakonzakonza maluwa monga momwe mukuoneramu nde ndinawajambula kuti tizizakumbukila kuti tsiku linalake atadzukako bwino nkzgwragwira maluwa kumakonzakonza maluwa tizizakumbukila

I cannot understand this picture clearly, we have flowers on our house and yesterday she tried to wake up a bit well and its when she was gardening them as you can see, so I took a picture of her so that we can be remembering that on this other day she woke up well, she was gardening the flowers. We will be remembering.

I: Owoo, ndiye pachithunzi chimenechi mungatiuze zotani zokhuzana ndi moyo umene mumakhala?

Ok so what can you tell us on this picture about the life that you are living?

03/02: Apopo ndizo penapake moyo umene timakhala ndiwovutabe masiku ambiri amadzukabe ndi mofooka komabe penabe amadzuka ndi mphamvu monga mmene mukuoneramu

On that some how the life that we are living is very difficult, most of the days she wakes up very weak but sometimes she wake up strong as you can see.

I: Mmm, nde ndimwayi wotani kwa ife kuti tithe kuwupitisa patsogolo moyo wanu kupyolera mu chithunzi chimenechi?

Mmm, so what chances are there for us to improve your life from this picture?

03/02: Ifeyo tinakakonda kuti amayiwo mutapitiliza kuwathandiza ndimankhwala monga amalandila tsiku ndi tsiku ku tiyanjane uko kutibe umoyo apitilizebe pena ndi pena kukhala ndi moyo wamphamvu nde inuyo mukathandize kuti mpata wa mankhwalawo ukapezeka kumawapatsa komaso kutsogolo kuno kutapezekaso mankhwala ena ake oposaso mwina awawa kumazawapasaso kuti mwinano azikhala ndimphamvu 100 percent kuti azizakumbukila zichani zintchito zimene amagwira monga kulambula kaya kukonzakonza pakhomo. Mmm

we would love if you can continue to help the woman with the medicine like the way she does at Tiyanjane so that she should continue having a health life, so you should help that the medication chances are there and she should be given, and also maybe if there can be found another medicine in the future better than these ones and should be given to her also so that she should be **100 percent** and remember chores that she was doing like sweeping or cleaning the premises .Mmm

I: Tathokoza kwambiri

Thank you very much

03/02: Zikomo

Thank you

I: Tiombe mmanja motani?

how do we clap hands for you?

03/02: Aaah basi mwabwino

Aah in a good way

All: (clapping hands)

(clapping hands)

SEVENTH PARTICIPANT

I: Nanga chithunzi ichi?

What about this picture?

06/01: Ndichanga

Its mine.

I: Eeeh?

What?

06/01: Changa

Mine

I: Owoo eee tatifotokozeleni zokhuza chithunzi chimenechi.

Ok, yes explain to us about this picture.

06/01: Ameneyo ndi mwana wanga ndinamujambulayo

That one is my child I took a picture of him.

I: Mmm

Mmm

06/01: Mmm

Mmm

I: Amatani pachithunzicho?

What was he doing on the picture?

06/01: Mwanayo amayenda nde ine basi ndinatuluka mnyumba ndinangoima ndinazamuitana akuyenda wazandiyang'ana ine ndamujambula

the child was walking, so I just came out of the house and I called him when he looked at me I just took a picture

I: Owoo

Ok

06/01: Eee

Yes

I: Nanga ndichifukwa chani munajambula chithunzi chimenechi?

So why did you take this picture?

06/01: Ndajambula chithunzi chimenechi chifukwa choti ndimafuna ndizikumbukila mwana ameneyu ndimmene zinalili nthawi imeneyo kuti iyeyu abadwe nthawi imeneyoyo ndili oyembekezera ndinali oti odwala ndikudwalika osati mmene ndililimu ndinali oti ndili pa waste koma ndimakhala ndi maganizo mwana ameneyu azabadwa wabwinobwino okufa wamoyo nanga zizandithera bwanji komabe ndinaona kuti mulungu anali mbali yanga komanso madokotala kundithandiza mwana wanga zochitika limozi zochitika kumandiuza kuti nkhawa usamakhale nazo nde ine kuzapeza mwana ameneyu alichonchi ndi chifikwa ndinamujambula kuti ndizizakumbukila history ya mwanayu

I have taken this picture so that, I wanted to be remembering this child with the way it was by then to give birth to him, I was very sick when I was pregnant than the way I am now, it was **waste** (worse) but I had a lot of thoughts thinking that, will this child be ok or dead and how will it be? then I saw to it that God was on my side and also the doctors assisting me in all the process and were telling me that, “you should not be anxious,” so finding out that this child is like this that is why I took a picture of him to remember the child’s **history**.

I: Mmm

Mmm

06/01:Mmm

Mmm

I: Ndeno mungatiuze zotani zokhuzana ndi moyo umene mumakhala pogwilizana ndi chithunzi chimenechi?

So, what can you tell us about the life that you are living in connection with this picture

06/01: Moyo wakebe umakhala ovuta monga mmene zinthu panopa zilili komabe umaonetsetsa kuti olo zizivuta choncho umangozisiya zinazo kuti zizikhala choncho komabe tiyenera kukhala mmene timakhalila

Its a difficult life with the current situation of things,but still more you makes sure that although things are hard like that you just leave those some other things like that but we should live the way we do.I

I: Mmm

Mmm

06/01: Eee

Yes

I: Mukati ndimmene zinthu zilili ndichani mukutanthawuza?

What do you mean when you say the way things are?

06/01: Moyo wamasikuano ndiokhala kuti osalidwa eti mchitidwe wathu tikamakhala anthu ena mwina kumatifunila zabwino ena ayi nde basi ndimmene ifeyo timangovomeleza kuti tiyeenera kukhhalila tsiku ndi tsiku

We live a discriminated life these days some people wishes us good while others not because of our behavior, so we just accept that that’s the way we are supposed to live each and every day.

I: Mmm

Mmm

06/01: Eee

Yes

I: Mmm, mukutanthauza kuti anthu amakusalani?

Mmm, you mean people discriminates you?

06/01: Anthu ena eti amatheka ena kukumasukila kumakuuza kukulimbikisa ndi mmene iweyo vuto lako ukudusa eti ndimmene zilili koma pamapezeka ena mwina kuku osamakuwuza ngati zimenezi komabe mwina kumangokhala ndi moyo uja kukhalangati kuti akuonesa kuti iweyo siofunikila ayi

Its possible that some people encourages become very free and encourage you with the problem that other people can be free to encourage you with the problem that you are facing or passing through right, thats the way things are but there are some maybe doing, they don’t tell you but still living a life that makes you feel like you are not important.

I: Mmm

Mmm

06/01: Eee, nde moyo timadusira

Yes that’s the life we are passing through.

I: Mmm, nangano chithunzi chimenechi chikupeleka mwayi wotani kwa ife kuti moyo tithe kuwupitisa patsogolo?

Mmm, so what chances does picture give us so that we can improve your life?

06/01: Zili ndi inu adokotala kumatilimbikisa ndimmene mwaonelamu kuti kodi mmoyo wa mayiwa ungapite patsogolo bwanji ndi mmene ndafotokozelamu

Its up to you the doctor to encourage us with the way you have seen that, “how can we improve the life of this woman, “with the way I have explained.

I: Mmm. Chabwino, chabwino.

Mmm. Alright, alright.

06/01: Ndithu

Sure

I: Nanga chithunzi chimenechi tatifotokozeleni?

What about this picture, can yo explain to us?

06/01: Chithunzi chimenecho ndi mwana wanga amaphika ndiye ine nde ndinamujambula

That is my child on the picture, was cooking so I took a picture.

I: Mmm

Mmm

06/01: Eee

Yes

I: Chifukwa chani munasankha kujambula chithunzi chimene mwajambulachi?

Why did you choose to take this picture?

06/01: Ndasankha chithunzi chimenechi kumujambula iyeyu chifukwa iyeyu ndi amene nthawi zambiri amatha kumandithandiza basi ndikakhala kuti mwina ndatopa iye amaphika kutani pamenepajapo anali atangovula kumene yunifolomu akuchoka ku sukulu mukuoneramo enawo ndi makope akulongedza koma atamaliza kuphika

I have chosen to take a picture of her because he is the one who mostly helps me, maybe when am tired she cooks, on there she was coming from school he just undressed his school uniform, as you can see those others are her books he was packing them after she had finished cooking

I: Mmm

Mmm

06/01: Eee

Yes

I: Ndi mwana wanu?

Is she your child?

06/01: Mwana wanga eya

Yes, my child

I: Mmm, nde chithunzi chimenechi chikutiuza zotani zokhuzana ndi moyo umene mumakhala?

Mmm, so what does this picture tell us about the life that you are living?

06/01: Chithunzi chimenecho chikutanthauza kuti mmene timakhalila ifeyo ndimmene mwanaso pena amatha kumatani kumandilimbikisa pazina ndi zina timatha kumamasukilana kumacheza nkhani zina ndi zina kumaiwalanso mwina timapologalamu tina timene ndilinato mwina kaya ndichani koma ndibwera ndi iyeyu kumacheza kumatani ndimatha kumaiwala nkhawa zina ndi zina zimene ndimakhala nazo

That picture means that the way we are living its also the way the child happen to what, encourages me on some other things. We happen to be open to each other, chat up to the point of forgetting some other programs that I have or whatever but will come and chat with her and I happen to forget the some anxieties that I have.

I: Mmm

Mmm

06/01: Eee

Yes

I: Nangano chithunzichi chikupeleka mwai wotani kwa ife kuti moyo tithe kuwupitisa patsogolo?

So, what chances does this picture give us so that we can improve your life?

06/01: Ndimmene ndafotokozelamo kuti mwana ameneyu ndimathandizana naye zochita mwinaso mmene ndimakhalila ndimmene zililimu ndiye adokotala mukhoza kuona kuti munthuyu ndimmene akukhalila ndimmene wajambulila chithunzi ndimmene zilili tingapitise patsogolo motani moyo wake

That’s the way that I have explained that we happen to assist each other on a lot of things with this child and maybe the way I live with the condition now so you can see as a doctor that, “with the way she is living and how the way she has taken the pictures and the condition also, how can we improve her life?”

I: Mmm

Mmm

06/01: Eee

Yes

I: Nanga chithunzi chimenechi

What about this picture?

06/01: Chithunzi chimenecho ndiku sunday school ku church ineyo ndajambula chithunzi chimenechocho sunday

on that picture is at **Sunday school** at **church**, I took that picture on S**unday.**

I: Mmm

Mmm

06/01: Eya

Yes

I: Chifukwa chani munajambula chithunzi chimenechi?

Why did you take that picture?

06/01: Ndajambula chithunzi chimenechi chifukwa choti munthu nthawi zina ukakhala kuti ukudwala ukuyenera kumamva mau a mulungu sikuti mmene ukudwalamo uzingokhala kudandaula osakhala kumva mau a mulungu ukamamva mau a mulumgu paja wekhaso pena umatha kuzisefa kuti ineyo inde vutoli labwera ndikuyenera kuvomeleza sindikuyenera kukhala odandaula mpakana kalekale nde ndichifukwa chake ndinajambula kuti ndikumbukile kuti ndimayeneraso kupezeka malo ngati amenewa

I took this picture because sometimes you are supposed to hear the words of God when you are sick you shouldnt just be worried and not hearing the word of God because you are sick, when you are hearing the words of God you happen to sieve your self that, “yes this problem came in but am not supposed to be disappointed for the rest of my life but accept,” that is why I took this picture since am also supposed to be in places like these.

I: Mmm

Mmm

06/01: Eee

Yes

I: Nde chithunzi chimenechi chikutiuza zotani zokhuzana ndi moyo umene mumakhala?

So what does this picture tell us about the life that you are living?

06/01: Apapa zikutanthauza kuti moyo umene ndimakhala umakhala moyo ovuta komabe tikuyenera kuziwa kuti nyengo zinazo zovutazo tizizisiya potelo koma tizikumbukila kuti tsiku lina ngakhale moyo utawawa bwanji ngakhaleso moyo utakoma bwanji koma pali tsiku lina limene ifeyo tiyenera mau a mulungu tizikhala nawo mumtima mwathu chifukwa munthu pakati pakudwala pamakhala zinthu ziwiri imfa kapena moyo nde poti nthawi imene ukupemphera zinthu zina zimatha kukuyendera bwino ndichifukwa chake ine ndinaganiza zojambula kumenekoko

here it means that the life that am living is a very difficult one but still more we have to know that those other hard times should be placed somewhere there but remember that although life can be very painful or can be very sweet one day, there is a day when are supposed to have the word of God in our hearts because there are two things while one is sick death or life so since things happen to work for you the time you are praying that is why I thought of taking a picture there.

I: Nde chithunzichi chikupeleka mwayi wotani kwa ife kuti moyo wanu tithe kuupitisa patsogolo

So what chances does this picture give us to improve your life?

06/01: Chifukwa chokuti anthu madokotala mumatha kumathandiza monga kumankhwala chani njila zina ndi zina zimene ife sitingakwanise komanso pa paokha abusa amatha kutaniso kukulimbikisaso mothandizana ndi inu madokotala ndichifukwa chake ndimaganiza choncho

It is because the doctors can help us in terms of medicine and other ways that we can not afford, but also a pastor alone can encourage you together with you doctors that is why I thought like that.

I: Tathokoza kwambiri kaya pali chinthu china chowonjezera anthu amene munajambula zithunzi

Thank you very much, is there anything to add, those who took these pictures?

M: Kwatsala munthu mmodzi

There is one more participant to take part.

I: Kwatsala mmodzi?

There is one left?

M: Eya

Yes

M: Tiwaombere mmanja

We should clap hands for her.

I: Tiombere mmanja motani?

how should we clap hands for you?

06/01: Basi mulimonse

In any way

I: (laughs)

(Laughs)

All: (Clapping hands)

(clapping hands)

EIGTH PARTICIPANT

I: Amene anajambula chithunzi ichi tatifotokozeleni

The one who took this picture, can you explain to us?

05/02: Chithunzi chimenecho chikutithandauzila kuti peshenti wathu pamene wazuka wangotsuka mkamwa ayenera kudya phala atatha kudya phala tiyenera kumupasa madzi kuti akasambe adye

This picture means that our patient should be eating porridge after cleaning teeth as soon as they have just woken up in the morning, after eating porridge we need to give them water to bath.

I: Mmm

Mmm

05/02: Eee

Yes

I: Chabwino, chithunzi chimenechi chikutiuza zotani zokhuzana ndi moyo umene mumakhal?

Alright. What does this picture tell us about the life that you are living?

05/02: Moyo umene timakhala pachithunzi chimenecho timasangalala kuti tikapeleka phalalo kwa peshentiyo amadya ndithu nde moyo wake ndimmene ukukhalila

the life that we are living from that picture, I am happy that when I give porridge to the patient he eats so that’s how we are living.

I: Sindinamvetu

I didn’t hear you.

05/02: Ndiye ndimmene moyo wathu ukukhalila

So that is how we live our life.

I: Owo nde ndiuthenga wotani umene mungatipase ifeyo kuchokera muchithunzi chimenecho?

Okay, so what massage can you tell us from this picture?

05/02: Tiyenera tisamunyoze peshenti pamene akudwala ngati akukana zakudya tiyenera kumulimbikisa kuti ayenera kuti adye chakudyacho

We are not suppose to insult the patient, We are supposed to encourage him to eat if he is refusing to eat.

I: Mmm, chabwino chabwino. Ndiye pankhani yokhuzana ndi moyo umene inuyo mumakhalali kumene kumene mumakhalako chithunzi chimenechi chikutiuza zotani?

Mmm, alright alright. So now on the life you are living where you stay, what does this picture tell us?

05/02: Chikutiuza kuti pamenepo ndiye pandivuta kuyankha

It is telling us that, that is difficult for me to respond.

I: Chabwino nangano chikupeleka mwayi otani kwa ife kuti moyo wanu titthe kuupitisa patsogolo kupyolela chithunzi chimenechi?

Alright so what chances does this picture give us so that we can improve your life?

05/02: Chithunzi chimenechocho chikupeleka moyo wabwino okuti peshenti olo akudwala choncho akamwa mankhwala ayenera kudya chakudya ngati chimenechi

This picture is providing good life to a patient, when sick like this and taking medicine, is supposed to eat food like this one after taking medicine.

I: Mmm. Nanga chithunzi chimenechi tatifotokozerani?

Mmm. What about this picture, can you explain to us?

05/02: Chithunzi chimeneechocho chikuti peshenti wathu zovala zake tiyenera timuchapile tisakhale kuti ayi chapani nokha koma tigwiritse ntchito ifeyo pomuchapira peshenti wathu uja

That picture is saying that we are supposed to wash our patients clothes, we should not leave them for her to wash , but we should be the ones to wash for the patient.

I: Chikuchitika ndi chani pachithunzipa?

What is happening on this picture?

05/02: Chithunzi chimenecho pamene chikuchitika ndichokuti zovala zake zizikhala zakilini zisamakhale zakuda

What was happening on that picture is that the clothes for the patient should be **clean**, they should not be dirty

I: Chabwino, apopo ndizoti mwachapa chapa mwayanika

So there you have washed and hanged the clothes.

05/02: Eee

Yes

I: Nde chithunzi chimenechi chikutiuza zotani zokhuzana ndi moyo umene mumakhala

So what does this picture tell us about the life that you are living?

05/02: Moyo umene tikukhala chithunzi chikutanthauza kuti peshenti wathu pamene wadya ayenera kusamba ndikuvala zovala zoyera

About the life that we live, the picture means that that when our patient ate, she is supposed to take a bath and wear clean clothes.

I: Nangano pali uthenga wanji umene mungafune kutiuza ifeyo kuti tiziwe zokhuzana ndi chithunzi chimenechi?

So what massage would you like to tell us, for us to know about this picture?

05/02: (quite)

(quiet)

I: Chabwino, chithunzi chimenechi chikupeleka mwayi otani kuti moyo wanu tithe kuwupitisa patsogolo?

Alright, what chance does this picture give us so that we can improve your life?

05/02: Chithunzi chimenechi mutha kupeleka mwayi chifukwa peshenti wangayu asamakhale okhumudwa kuti zovala zanga ndizakuda andichapile ndindani nde chikutilimbikisa kuti tizimusamalila peshenti ndi zovala zakezo

You can give a chance to my patient from this picture to not stay worried that, “my clothes are dirty who is going to wash them for me?” So its encouraging us to take care for the patient and her clothes.

I: Mmm, nanga chithunzi ichi?

Mmm, what about this picture?

05/02: Chithunzi chimenecho ndinajambula nditawaikila madzi ku bafa ndiye zinangondisangalasa kuti ati ndavala tawelo ndikumatuluka mnyumba kuti azikasamba nde zinandisangalasa kuti ndizizaakumbukila kuti nditawapasa madzi anandivomeleza ndikutenga tawelo yawoyo kumapita ku bafa kumakasamba

I took that picture after putting water in the bathroom, so it just made me happy when she got a towel coming out of the house going to the bathroom, so I was happy that I will be remembering that after giving her water to bath, she accepted and she got her towel going to the bathroom and start bathing

I: Mmm

Mmm

05/02: Eee

Yes

I: Chithunzichi chikutiuza zotani zokhuzana ndi moyo umene mumakhala?

What is this picture telling us about the life that you are living?

05/02: Chithunzi chimenecho chikutiuza kuti ndikwabwino kuti peshenti ayenera akhale waukhondo asakhale osazisamalila pathupi pake ayi

This picture is telling us that its good for the patient to be clean not one who does not take care for her body.

I: Mmm

Mmm

05/02: Eee

Yes

I: Nde mungatiuze zotani ifeyo zokhuzana ndi chithunzi chimenechi?

So what can you tell us about this picture?

05/02: Ndingakuuzeni kuti muthanso kutilimbikisa kuti ndingawapange bwanji a peshentiwa

I can tell you that you can also encourage us how I can treat my patient.

I: Chabwino, tathokoza kwambiri pofotokoza. Kaya tiombe mmanja motani?

thank you very much for explaining. How do you want us to clap hands for you?

05/02: Mwa manganya

like Manganyas

PS: (laughing)

(laughing)

MK: Anena kuti chani

What did she say

I: Akuti mwa manganya kaya

she is saying like manganya’s

MK: Ngati manganya

Like manganya’s

MM: kuomba ngati mwa manganya paja timatani tikamaomba mwa manganya

Clapping like Manganya, what do we do when we are clapping hands like Manganya’s?

PS: (laughing)

05/02: Timaimba timapanga chonchi (clapping hands)

We sing and do like this. (clapping hands)

MM: Mutisogolera ndiinuyotu eti

You are going to lead us right?

PS: (laughing)

(laughing)

MM: Tiomba mmanja ngati muja amachitila manganya kuwauza kuti akayankhulila anthu kumachita kuti phuphuphu ife tiziomba

We will clap hands like the way Manganya does, telling people that they should be clapping hands after he has spoken, we will be clapping hands.

05/02: (singing) Zomwe tamva kuno azimai ndi azibambo

(singing) what we have heard here ladies and gentlemen…

All: (clapping hands)

(clapping hands)

05/02: Zikakhale zogwira ntchito mu mitima mwathu

Should work in our hearts.

All: (clapping hands)

(clapping hands)

05/02: Tisakanyoze ma peshenti

We should not show to the patients…

All: (clapping hands…laughing)…(interruptions)

(clapping hands…laughing)…(interruption)

I: Tathokoza kwambili kwa aliyense mwagwila ntchito yopambana, zikomo chifukwa chomvesera

zikomo chifukwaa cholankhula tikudziwa komano zinthuzi, zinthu zimene tikumvetsera kunoko

komanso tikugawana nkhani zimenezi chifukwa ndinu odwalawo kapenanso amene timayang'anira odwalawo komano ndi chinthu chofunikila kuti ndithu tasamalankhule zinthu zimene tikukambilana munomo kwa wina aliyense kunjaku oti mwangokumana naye, tikugwilizana nazo?

Thanks to everyone, thanks for listening, thanks for speaking. I think we probably know that the pictures, the things that we are hearing here and also the stories that we are sharing is because you are the patient or you are a guardian but its necessary to not talk to anyone about the things that we are discussing here, agreed?

PS: Eeya

Yes

I:nde anthu mane akutigawira zokhuzana iwowo, tonse tigawana zinthu zimene timakumana nazo nde zilingati mwapasidwa chinthu chinachake chamtengo wapatali ndithu ngati mphatso nde mukuyenera kusamala mphatso imeneyi simungamangopeleka kwa wina aliyense mphatso imeneyo kukumana naye munsewu nde tikuthokoza kuti mwayankha mafunso komanso mwajambila zithunzi nde ndipoofunikilaso kuti tigwilizane kuti tisamakangoyankhula kwa wina aliyense zimene tamva munomo tikhoza kuphunzilabe kuzera muzinthu zimenezi kuti mwina tikhoza kuthandizana wina ndi nzake kapenaso kuthandiza athu ena apadera koma tisamangolankhula kwa ma neba athu mukuziwa izi mukuziwa izi ayi zimenezo, tagwizana nazo

so the ones who are sharing stories to us about themselves, we will both share this and its like a precious gift so we are supposed to take care of this gift you cannot just give it to an so are thankful that you have answered the questions and you have also taken some pictures for us so it is also necessary to agree that we should not be telling anyone else what we have heard here we can learn from these things that we might maybe assist each other pr assist other people also but should not talk about it to the neighbors, “ee you know this, you know this, do we all agree?

PS: Eee

Yes

I: Owo, zikomo.

Ok thank you.

MM: Ndimmene anenelamu akufuna wina aliyense kuti atani?

a woman telling participants the way they have said they want every body to what?

PS: kuti timvesese

To understand.

MM: Ayi mmene anenelamu aliyense azikhala otani?

No, the way she has said everyone should what?

PS: Wachinsisi

Should have confidentiality

MM: Womasuka, Mukamalankhulla muziziwa kuti zimene ndikulankhulazi zithela muti mommuno sizituluka kunja.

Should be open. When you are speaking you should know that, “what I am speaking will end in here, will not get out of here.

M: kutha kuona

FGD2

Can you see?

07/01: Eee ndikutha kuona

Yes i can see

M: Are we all able to see

Enafe tikutha kuona

Ps: Eee

Yes

I2: mwina tikhoza kusendera pakati apa kuti tiziona ngati takhala mbalimabalimu.

Maybe we can sqeeze to the middle so that we can be seeing to those sitting on the sides.

M: Koma zikuoneka

But we can see

I2: Zikuoneka?

You can see

PS: Mmm

Mmm

I2: Chabwino

Alright (interruptions)

I: Nde tiyambepo eti tiyamba, mafunso tikuwaona amene kutsogoloko eti, eya tikufuna kuti chithunzi chinachilichonse chaima patsogolo eti anajambula chithunziwo kwambili ndi amene angatsogolele kumafotokoza zachithunzicho malingana ndi mafunso amene alipatsogolopa week yathaso tinapanga zomwezi mwina ngati tikukumbukila bwino bwino nde ndikukhulupila kuti week inoyi tiyankhula mwina moposa mmene tinapangira week yatha nanga mwina sitimakhala ngati tikuphunzira kumene eti

Now we should start we will start, we can see the questions in the front right, So we want every picture that is in the front right, the one who took the pictures should be the ones to lead us in explaining about the picture looking at the questions that are in the front last week we were doing the same if you can remember well so i believe that this week we are going to speak well more than we did last week maybe because we were learning for the first time.

PS: Mmm

Mmm

I: Chabwino

Alright.

I: Ndiye paja mwati ndani dzinalo amene anakakonza ziujenizi, paja dzina ndani?

now what did you say is the name for the one who did this, what is your name?

07/01: (name)

(name)

I: (name)

07/01: (name)

I: (name) Chabwino. Ndiye mungatifotokozele a jeffrey makoka kumene, fotokozani kuti chithunzi chimene mwajambulachi mungafotokoze kuti chithunzi chimenechichi mungachifotokoze bwanji?

(name) Alright. Now (name) can you explain where, explain about this picture you have taken you can explain about this picture, how can you explain it?

07:01: Chithunzi chimenechocho ndimwana wanga ameneyoyo mwana ameneyo amanena kutino amayi wake akamagwira zintchito zina nde amakhonzaso ameneyo kumaphika kumandipasa nde ndichifukwa chake ndinamujambula kutino ndizimuona ndizimukumbukilaso zimene amandichitila

That is my child on the picture she says that when her mother is doing other jobs she sometimes cook and gives me food that’s why i took a picture of her so that i should be seeing her and remember what she does for me.

I: Basi ndizomwezo zimene mumafuna mufotokoze?

Is that all what you wanted to explain?

07/01: Ndizomwezo zimene amai chifukwa mayi wake amakhala akundipasa ine mankhwala iyeuja amakhala akumaphika chakudya kutino adzandipasire ine ndichifukwa chake ndinamujambula kutino ndizikhala ndikumamuona nthawi ina iliyonse.

That is all, because while her mother is giving me medicine she is cooking food to give it to me that is why I took a picture of her so that I should be giving her each and every time.

I: Owoo ngati kuti amawathandizila amayi wake

Okay its like she helps her mother?

07/02: Eee kwambiri

Yes a lot

I: Kukusamalirani

Taking care of you?

07/01: Kundisamalira

Taking care of me

I: Chabwino

Alright

I: Ndiye pachithunzi apopo chikuchitika ndichani? Pachithunzipo amatani pamenepo?

So what is happening on that picture? What was she doing there on the picture?

07/01: Pamenepopo wangokhala pa khonde ndeno mmene anakhala pakhonde panja ndinamuuza kunena kuti ndikufuna ndikujambule ukhale chithunzi chakuti nthawi inailiyonse ndizikuona zimene umawapangila amayi ako pamenepopo samagwira ntchito ina iliyonse wangokhala pakhondepo

There she is just seated on the veranda so while seated out side on the veranda I told her that I want to take a picture of you so that you should have a picture for me to see you doing that for your mother anytime. So she wasn’t doing any chore there she is just seated on the veranda.

I: Mmm. Apapa pachithunzipa wangokhala koma poyambilira paja munafotokoza kuti ameneyuyu ndi amene amathandiza mai ache kukusamalirani

Mmm, so she is just seated here on this picture but at first you said that she is the one who assists her mother on taking care of you.

07/01: Kwambiri

So much

I: Palinso china mwina chimene mukufuna mufotokoze kuti kodi chikuchitika ndichani pachithunzi chimenechi?

Is there any other thing that you want to tell us about what is happening on this picture?

07/01: Chithunzi chimenecho ofunika kutino apitilize moyo wake mmene amachitila kwa ineyo chifukwa ngakhale ngati amayi wake achoka amapanga zondisamalira ineyo amene ndili pa khomo chifukwa nthawi zina ndimadwala kwambiri osafuna kudzuka mwendo wanga utayamba kupweteka nde amene ndimafuna kuti apitilizebe khalidwe lake ameneyu amapitiliza iye kumandithandiza

That picture she need to continue her life as the way she does for me because even when her mother is not around she takes care of me as one at home because sometimes i become very sick that i don't want to wake up when my leg is very painful that's why I want her to continue having that heart of taking care of me.

I: Chabwino mwina mungafunenso kuonjezera eti chifukwa zambiri mwafotokoza zimene tikufuna tifunse ngati funso lanambala lachitatu kuti ndichifukwa chiyani munajambula chithunzi chimenechi koma kwinaku ndikufuna muonjezere chifukwa chani mwanena kuti amakuthandizani komanso ndichifukwa china chani munajambulira chithunzi chimenechi?

Alright maybe you would like to add more right because you have already explained a lot on what we want to ask you like on question number three that why is it that you took this picture but somehow I want you to add on why did you say that she assist you and also what is the other reason for taking this picture?

07/01: Chifukwa china ndichonena kuti iyeyu ndimakhala naye pakhomopa koma sikuti sikuti ali pakhomo pompo iyayi anabwera kuchokera kumene amakhala kwa mamuna wake kuzabwera kunyumba kuno ndeno kuyambila pamenepo kunena kuti iyeso akhale akamaona chithunzicho aziziwa kuti bambo wanga amandikonda kwambiri chifukwa ndinamuuza kuti takhala apa ndikujambule nthawi ina ine ndizikuona zimene umandichitila kuti upitilize kupanga zimenezo

The other thing is that I stay with her here at home but she doesn't really stay here she just came from where she stays at her husband so since then its that she should also be able to see the picture and know that my dad loves me a lot because I told her to sit there and I should take a picture of you so that I should be able to see you some other times because of what you do to me and that you should continue doing that.

I: Chabwino mwinaso chifukwa china chimene mungakumbukile chimene chinakupangitsani kuti mujambule munthu ameneyu?

Alright maybe there is another reason you can remember that made you take a picture of this person?

07/01: Mmm ndinganene kuti pali zifukwa zambiri zimene ndinajambulira koma kweni kweni chimene ndinamujambulira ndimafuna kuti iyeyu azikhala akuziwa kutino inenso ndimamukonda akamandipangira zinthu zimenezo kusonyeza kunena kuti naneso ndimamudalira kunena kuti ammandithandizira mayi wake akakhala kuti palibe

Mmm can say that there are several reasons why I took that picture but most thing that made me to take picture of her is that she should live to know that I love her also, she is doing that for me it means I also count on her meaning that she assist me when her mother is not around.

I: Chabwino nde tikupitilira chithunzi chake chomwechi tikufuna tifotokozeleko zachithunzichi nanga kodi chithunzi chimenechi chikutiuza zotani zokhuzana ndi moyo umene inuyo mumakhalamo moyo watsiku ndi tsiku umene inuyo mumakhala kunyumbako tikati tikangoona chithunzichi chingatifotokozere zotani muchione bwino bwino chithunzicho mmene munachijambulira nde mukachiona chimenechocho ifeyo chingatifotokozere kuti inuyo mmumakhala moyo otani kunyumba kumene inuyo mumakhala tsiku ndi tsiku

Alright so we are proceeding with the same picture, we want to explain this picture about what is it telling us concerning the life that you live, the day to day life at home when we see this picture what can it tell us, you should look at the picture very well the way you took it so when you see it what can it tell us about the life you live at home each and every day.

07:01: Inuyo mukaona chithunzicho ndekuti mukhonza monga mmene ndafotokozera kunena kuti amandikonda kwambiri ndeno ndizosangalasa kuti mtsikana ameneyu amakonda abambo ake chifukwa chokuti chinachilichonse chimene ndingamuuze ine salephera kuchita kapena angamuuze amayi wake salephera kuchita iyayi nde nanunso mukaona ntchito zamwana wanga mwana ameneyu amawakondaso abambo wake kwambiri

When you look at the picture then you can, like I have explained that she loves me so much so its really exciting that this girl loves her father because she cant manage to not do anything that I tell her or even what her mother tell her so you too will see my child’s works that this child loves her father a lot.

I: Chabwino koma funso lathu likufunsaso kuti moyo umene mumakhalira kunyumbako pachithunzipa mungatifotokozere zotani mwafotokoza kuti mwanayu amakukondani komanso chithunzicho mwina chingatimasulireso bwanji pamene inuyo mumakhalira kunyumba pamoyo wa tsiku ndi tsiku

Alright but our question is asking about what you can tell us from the picture about the life you live at home you said that this child loves you so maybe what can the picture clarify to us on the life you live at home each and every day?

07/01: Eee ndinganene kutino moyo wathu wa tsiku ndi tsiku ndekuti umayenda bwino chifukwa cha mwana ameneyu ndiyeso monga ndafotokozera kunena kuti chinachilichonse amandichitira komabe inunso mukachiona muziwe kuti mwana ameneyi amagwira ntchito yayikulu kwambiri kwa ineyo ndimamudalira kwambiri

Yes I can say that our everyday life is just fine because of this child and also like I said that she does everything for me but still when you see it you should know that this child does a very big job to me and I count on her so much.

I: Mwanayo amakukondani mumamunyadira komanso palinso china chimene mwina mukufuna mufotokoze za mmene mumakhalira panyumbapo molingana ndi chithunzi chifukwa pachithunzipo ndikuonapo pakuoneka nyumba kuselikonso kukuoneka zinthu zina nde mwina mkutheka palinso mbali ina imene mungafotokoze yokhuzana ndimmene chithunzichi chikuonekera

The child loves you and you adore her maybe there is also something that you want to tell us about the way you live at your home concerning this picture because on the picture i can see a house behind and i can also see some other things maybe there is something else that you can say about this picture.I

07/01: Ndikhoza kunena kuti palibe zambiri zimene ndingafotokoze inde palibe zina ndi zina pakhondepo komabe ineyo sikuti ndingafotokoze kwambiri mopita kwina kwake koma mmene ndafotokozeramu

I can say that there is nothing else to explain but yes there are some things on the veranda but i cannot say a lot going beyond but the way i have explained

I: mmene mwafotokozeramo ndi momwemo

This is just the way you have explained

07/01: Eeeeh

Yes

I: chabwino

Alright

I: nanga chithunzi chimenechi chikupeleka mwayi wotani kwa ife kuti moyo wanu uthe kupititsidwa patsogolo kudera kumene mukuchokera pankhani ya kukhala ndi matenda a khansa ndiye moyo umenewowo watsiku ndi tsiku tikaona chithunzicho kapena mukaona chithunzicho kodi chithunzi chimenechocho chikupeleka mwayi wotani kuti chikhoza kuthandizira kuti moyo wanu watsiku ndi tsiku upititsidwe patsogolo kapena zinthu zisinthe mwina kusiyana ndi kale nditangoona chithunzicho mungafotokoze kuti bwanji?

what chances does this pictre give us so that we can improve your life from where you are coming from with the fact that you have cancer now on the day to day life when we see on this picture or when you see on this picture what chances can this picture help you to improve your day t day life o change things compairing from the past after looking at this picture what can you explain?

07/01: Pamenepo ndikhoza kunena kuti tsopano chithunzicho ineyo ndimafuna kuti inuso muzinena kuti mukhonza kupereka mankhwala ondithandiza ineyo kunena kuti mwina mwake anzathuso amene akubwerawa mwina kutsogoloko asazapezekeso mwina akuvutika monga mmene tavutikira mwinamu ndeno chifukwa chake ndikupempha kuti patapezeka mankhwala zikhoza kuzachita bwino kuti azapitilize kulandilisa ndi chithunzi chimenechi

Actually on this Photo, i wanted that you should also be saying that you can be giving medicine that can help me so that maybe others who are coming in the future they should not be suffering as we are doing, that is why we are asking for the medicine so that can do much better with our lives

I: chabwino chifukwa chani mwafotokoza chonchi?

Alright why have you explained like this?

07/01: ndafotokoza chonchi chifukwa chonena kutino monga ngati mbuyomu mwa tapitamu timalandira jakisoni choncho jakisoni uja anatha nde ndikuona kuti panakapezeka mankhwala ngati amenewo amatithandiza kwambiri umbali yamatendawa

I have explained like this because in the past we were receiving injections where now we are not receiving it, now i see that if this medicine was available they were helping us for this disease

I: chabwino ndiye kuzera muchithunzi ichocho chikupeleka mwayi otani ku uthenga umenewu?

Alright, from that photo what picture does it give on this message?

07/01: monga inuyo amene mwandiitana kuno mukhoza kundithandiza mwina mwake kuti mwina mwake mankhwalawo akanabwera zikanachita bwino kunena kuti tizilandila mankhwalawo

As the one who called me, you can help with medicine so that we could be receiving that medicine here

I: ndimafuna mungofotokozerapobe kuti mwana wanu akuoneka pachithunzipo eti?

Is this your child on this photo?

07/01: eee

Yes

I: akupeleka uthenga otani kapena chithunzi chimenechi chikupeleka mwayi wotani kuti mmene mwanenera ife achipatala tichitepo kanthu mwina mbali imeneyoyo mutayifotokozerala bwino bwino

What message is she carrying, in other way round what opportunity does this photo carry so that as way you have said before you want us to do something can you just explain clearly?

07/01: mmmm tinganene kuti nanga si monga ndafotokoza poyamba ndimalandira jakisoni sopano mankhwalawo atha palibe iyayi ndiye ndimaona kuti nthawi imeneyo zimachita bwino ndikalandira jakisoni mankhwala amagwira ntchito mkati mwa thupilanga nde inuyo monga mwatiitana kunoko mankhwala amenewo anakakhala poona chithunzi chija zinakachita bwino kuti mwinamwake tikhale bwino

Mmm as I said before that at first that I was receiving injection but as I am talking that medicine is not available, so considering the time we were receiving that injection we were healed and just because you have called me here if that medicine is available I think it is better as you have seen this photo so that we can be better after being treated

I: chabwino ndiye tipite chithunzi chinatu eti palinso china chimene mukufuna muonjezele pa chithunzi chimenechi?

Okay, so can we go to another photo or you have to add something on the past photo?

07/01: chimenecho palibe

No, I have nothing to add

I: Chimenechi chipitetipete pena

So we should go to another photo?

07/01: mmmm

Yeah

I: chimenechi mukuchiona bwino bwino chithunzichi?

Do you see clearly this photo?

07/01: eee kwambiri

Yes

I: chabwino ndiye mungafotokoze chithunzi chimene mwajambulachi tangofotokozani kuti chithunzi chimenechi chiku mungofotokoza chithuzi chimenechi mmene munafotokozera choyamba chija

Alright, so explain why you decided to take this photo, you just have to explain the way you explained the last photo

07/01: chabwino

Okay

I: mmm

Yeah

07/01: ameneyonso ndi mwana wanga wanambala two nde nayenso alindimatenda pamene alipo nde matenda amene alinawowa kufananabe kukhala ngati pang'ono ngati angawa koma iyeuja amagwa nde ndichifukwa chake ndamujambula kuti mwinamwake kuti kapena angakhalenso ndimatenda ngati angawa ndichifukwa chake ndamujambula kuti ndizimuona pamene wakhalapo

That one is my second born child and he also has the similar disease as mine the only difference is that he also suffer epilepsy so I snapped him so that I should see where he is

I: chabwino mwati amadwala matenda anji?

Alright, so you said what disease is he suffering?

07/01: amagwa khunyu

Epilepsy

I: amagwa khunyu?

She has epilepsy?

07/01: eee

Yes

I: chabwino ndiyeno mukupitilizabe kuti chikuchitika ndi chani pa chinthunzi chimenechi pamene muumamujambula amapanga chani pachithunzi chimenechi?

Okay, so continue what was he doing by the time you were taking the photo?

07/01: pamenepopo palibe chimene akupanga iyayi koma ndinangomuika pamenepo kuti thupi lakero lilindi zilonda kwambiri nde ndinamuikapo pamenepo kunena kuti mwinamwake tizizakumbuka kutsogolo kuti nthawi ina ndinaza ndili pakafukufuku nndinamujambula mwana wanga nthawii imeneyoso asakupeza bwino iyayi

He was not doing anything but I took him a photo because his body is full of wounds so I did that to be a memory that I participated where a come with camera where I snapped my child by the time he was not feeling well

I: chabwino

Ok

07/01: eee

Yes

I: chabwino ndichifukwa chani munajambula chithunzi chimenechi?

Okay, why did you take this photo?

07/01: ndinachijambula chifukwa chokuti pamene ndimamujambulapo akumangozikanda kanda kuti ndinajambula pamenepopo tizizakumbukila kuti nthawi imene ndinamujambula nthawi imeneyo asakumva bwino nthupi mwake

I took this photo because by the time i was taking a picture he was scratching her body so i took it so that we should be remembering that on that time i was taking her a photo while she was not feeling well

I: asakupeza bwino

Was not feeling well

07/01: asakupeza bwino nthupi mwake

He was not feeling well

I: chabwino

Ok

07/01: eee

Yes

I: nde chithunzi chimenechi chikutiuza zotani zokhuzana ndi moyo wanu umene mumakumana nawo kudera kumene mwachokera mwangotionetsa mwana yemwe nayenso mwanti akudwala amadwalamatenda akugwa ndiyeno moyo wanu wa tsiku ndi tsiku chithunzi chimenechi chikutipasa uthenga wotani?

What does this photo tells us about the life that you are going through from the area you coming, you have just showed us your child who is also suffering from epilepsy so what message are you telling us on your day to day life?

07/01: chithunzi chimenechichi chikundipanga ine kunena kuti ndimafuna kuti mwina kapena matenda amenewoso nayenso alinawowo akanatha monga mmene zinakhalira chifukwa ndinamujambula kuti mwina ndikuwoneseni nanuso kunena kuti nayenso akumva bwanji eee

This photo tells how my son is suffering so is my wish that he can also be helped and my aim of taking a photo and bringing here is that you should see and help

I: nde mukuti bola nayeso matenda anakatha fotokozanibe kuti ndichifukwa chani muli ndi malingaliro amenewa mwina ndi mavuto wotani amene akupangitsani kuti mwina munene kuti koma mwanayu matenda ake akanatha?

So you are saying its better also your son to be healed from this disease, why is that you have this idea, what kind of the problems have made you to think it is better for son to be healed?

07/01: ameneyu chifukwa chonena kuti timapita nayeso kuchipatalako nde ndimamulandilila mankhwala nde ndimaonabe kuti ngakhalebe zimasintha komabe sizisintha motibwanji ndichifukwa chake ndinamujambula akudwala kunena kuti mwina kapena matendawa angathe mkati mwathupi lakelo

This is because I mostly him to the hospital though we receive treatment but there is no big change that is happening that is why I took a photo so that maybe can be helped

I: chabwino ndiye komalizira chikupeleka mwayi wotani chithunzi chimenechichi kuti mwina pangakhale kusintha ndimmene mumakhalira moyo wanu wa tsiku ndi tsiku kut titha kuupititsa patsogolo atati anthu achiona chithunzi chimenecho ngati mmene tachionelamu mungapeleke uthenga wotani umene ungakhale ngati ukutipasa mwayi kapena kubweletsa mwayi woti pakhale kusintha pa moyo wanu wa tsiku ndi tsiku?

Okay, what opportunity does this photo carry, is there any change with the you are leaving with your life day to day so that it can help if people have been able to see this photo as the way we are seeing, what message is there if your given an opportunity or else brings fortunes to your day to day life?

07/01: patapezeka mwayi woti mankhwalawo alipo ndikumpatsa ndikuchira ndekuti moyo wathu wa tsiku ndi tsiku uzakhala okondwa kwambiri ndichimene ndingapemphe eee

If there can be a chance of getting medicines and give him and get healed our day to day lives can be happy this is what I can ask

I: patakhala mankhwala ochilisa mwanayu

If there is such medicine of healing your son

07/01: eee

Yes

I: ndekuti moyo wanu wa tsiku ndi tsiku uzapita patsogolo

It means your day to day life can improve

07/01: ee uzapita patsogolo kwambiri pamozi ndi ineyo

Yes, it can improve a lot together with me

I: chabwino nde mwina tisanapite ku chithunzi china paliso nkhani ina iliyonse yomwe mukufuna munene pachithunzi chimenechi?

Okay, maybe before we go to another photo is there any anything that you want to tell us on this photo?

07/01: ayi palibe

No

I: palibe?

Nothing ?

07/01: eee

Yes

I: chithunzi chomaliza choti mufotokozeso ndichimenechi eti

This is the last photo that you are suppose to tell us

07/01: eee

Yes

I: mukutha kuchiona bwino bwino?

Are you able to see this photo clearly?

07/01: eee kwambiri

Yes

I: chabwino ndiyeno pepani ndizingofunsabe mafunso omwewo nde nanga si ichi ndi chithunzi chijachiso ndi china chijaso ndi chiina

Alright sorry that i will be asking you the same questions this picture is not the same as the other one and the other one as well

07/01: zoonadi

You are right

I: mungafotokoze kuti chithunzi chimenechi munachijambuliranji kapena mungafotokoze chithunzi chimene mwachijambulachi

Can you explain the reason you took this photo?

07/01: chithunzi chimenechocho ndi amai akwathu amene amandisamalira ineyo ndikadwala eee ndeno ndinajambula chimenecho kuti ndiziawona chifukwa nthawi inailiyonse ndikadwala amayesesa kundi thandiza ineyo ndithu kutino tipite ku chipatala kukandithandiza chifukwa chake ndinawajambukutino ndiziwaona

on that photo is my wife who always takes care of me when I get sick, then i took this one so that i should be seeing her because every time when I get sick she tries to help me so that we should go to the hospital to get the help

I: nthawi ina iliyonse ndi amene amakuthandizani

She is the one who always help youin all times

07/01: ndi amenewowo

She is the one

I: chabwino koma pachithunzipo chikuchitika ndi chani amatani tsikulimenelili pamene mumawajambula pachithunzipa?

Alright but what is happening on that Photo, what was she doing on that day you were taking her this photo?

07/01: pamenepopo amachokera kwa azinzawo kunali kubwera kumenekoko

She was coming from her friends

I: owoo akungobwera kumene

Okay, just coming

07/01: akungobwera kumene nde ndinawauza kuti tayimani pamenepo nde anayima nde ine ndikuwajambula ndati iyayi mukuziwa kale kunena kuti ndikufuna ndikakuoneseni kunena kuti amenewo nde imani pomwepo musanafike kunoko ndinawajambula akuchokera kumene anapita akufika pakhomo

She was just coming then i told her to stop on the spot and she stopped then i took her a photo while standing on the same spot up to the time reached home

I: amakacheza kwa azinzawo

She was chilling with her friends

07/01: Eeee

Yes

I: amatha ndithu kukacheza kwa azinzawo ndikubwera?

Does she really go to her friends and come back?

07/01: amatha kukacheza kwa azinzawo

She does chill with her friend

I: chabwino nangano ndichifukwa chiyani munajambula chithunzi chimenechi?

Alright but why did you take this photo?

07/01: chimenechi ndinajambula chifukwa chonena kuti nthawi ina iliyonse amandithandiza ndi iwowo tsopano kuti ndisawajambuule sizichita bwino komabe ngati nditafika kunena kuti mwinamwake ndadwala kwambiri ndipamene ndimaona kuti eee mkazi ndiamene amandithandiza panene ndikuvutika kwambiri eee

I took this one because she is the one who helps me every time now for me not to take her a photo could not be good still more if I get sick she is one who help me most

I: chabwino nde chithunzi chimenechi chinngapelekeso mwayi wotani kapena chithunzi chimenechi chikutiuza zotani zokhuzana ndi moyo wanu umene mumakhala tsiku ndi tsiku?

Alright so can this photo give any opportunity or what does this picture tell us about your day to day life?

07/01: chithunzi chiimenechicho mumoyo wanga wa tsiku ndi tsiku ndekuti zimachita bwino chifukwa chinachilichonse chimene ndingachifune ineyo ndikawafotokozera amayi akwathuwa amapanga chomwecho kwa ineyo ndiye osapita kocheza kapena kupita kocheza koma ameyesesa kundisamalira kwambiri kuti mwinamwake moyo wanga ukhale wabwino

This picture in my day to day life things goes well because everything that i want when i tell my wife she does for me whether she didn't go or she goes to her friends but she always tries to help me so that maybe my life can be ok

I: Ok, kutanthauza kuti moyo wanu ndithu umakhala osamalidwa tsiku ndi tsiku chifukwa cha amene ali pa chithunzipa

Ok, so you mean that your life is really been cared every day because of the one on this photo

07/01: kwambiri

Extremely

I: chabwino komalizila kumapeto kweni kweni nanga chikupelekano mwayi wotani woti pa moyo wanu zinthu zikhoza kusintha kupita patsogolo

Okay, before we finish what chances are you getting to your life that things can change towards the future?

07/01: moyo wanga ukhoza kusintha kwambiri monga mmene ndafotokozela muja kunena kuti patapezeka ndithu mwayi ofikira kwa athu ena nndikundithandiza kumbali ya mankhwala kuti moyowu upitilizekuchoka pamene ndili uzafike pa mtendere weni weni ndikhoza kuzasintha kwambiri

My life can change a lot as i have already said that if there can be a chance to the people that they can be helping me in terms of the medicine so that the life should continue from where i am now and reach a point of being feeling better

I: chabwino

Alright

I: tathokoza chithunzi chachitatu chinali chimenechi koma mwina tisanamalize kweni kweni mwina pali choonjezelaso china chofotokozera pachithinzichi

Thank you this picture was the third one but maybe before we finish there can be something that you can add to explain on this photo

07/01: mau amene ine ndingaonjezere ndionena kuti ine ndingopempha kuti mai akwathuwo apitilizebe kundisamalira mpakana pa moyo wanga onse mau amene ndinganene ndi amenewo

Words that i can add on is that, i just want to ask that my wife should continue taking care of me for the rest of my life that's what i can say

I: chabwino

Okay

I: tathokoza kwambiri eti

Thank you very much

SECOND PARTICIPANT (05/01)

I: Nde tikupitiliza tili pa mayi (miss) eti mukutha kuziona ooh aaah mwabwera ndi ziwiri eti

Now we are continuing we are on (name) right are you able to see

05/01: Eee

Yes

I: Mayi (name) chithunzi chili patsogolocho mukuchiona?

Miss (Edna) are you able to see the picture which is in the front?

05/01Mmm

Mmm

I: Chabwino tathokoza ndiye mungafotokoze chithunzi chimenechi munachijambula mungotifotokozera kuti aah chithunzi chimenechi mwakuti mwakuti

Alright thank you now can you explain about this picture you took just explain to us that aah this picture so so so

05/01: Chithunzi chimenechichi ndi okala imene ili pakhomo pano eya okola imeneyiyi ndiimene ndimapanga ine bussiness ndikumagulisa geni yanga koma chifukwa choti cha matenda ndinaitseka nde ndinajambula okalayi kuti ndiziona kuti okala imene ndinaitseka chifukwa choti ndinali pakati pamatendakoma ndiyoti ili pa khomo pomwepo

On this picture is a hawker that is on my house ye this hawker is the one i was doing business on i was doing business but because of the sickness i closed it and i took a picture of this hawker so that i should be seeing it that i closed this hawker because i was very sick, this is right at my house.

I: Mmm. Ngati ndakumvesa bwino bwino mwati chithunzicho ndi okala eti?

Mmm. If I have heard you properly you have said that on the picture is a hawker right?

05/01: Mmm

Mmm

I: Imene mbuyomo mumatsegula mumagwiritsa ntchito nde titai tifunse kuti chikuchitika ndichani ndi chani pachithunzipo mungati bwa?

The one that was working in the past now if we can ask what is happening on this picture what can you say?

05/01: Pamenepapa chimene chikuchitika ndichokuti ndinakakhala kuti ndinali ndimphamvu mmene zinaalili kale ndekuti basi siinakakhala iliyotsekayi bwenzi pano iliyotsegula kuti mwini wanga ndikutani ndikugulitsa komano malingana ndikuti kusowa kwa mphamvu ndinkana okala imeneyi ndinaitseka eya ndeno ndimaligalira kuti okala imeneyi imandipasa mwina zina ndi zina za umoyo wanga eti osapempha mwina kwa ana china chilichonse chikachitika mwina ndimangotulusa kutani kupeleka lelo ndinatseka ndekuti china chilichonse ndimangodalila ndani mwina ana nde basi ndinangopanga chithunzicho kuti ndiziziwa kuti mmene ndinali ndi mphamvu inali yotsegula koma panopa poti ndikudwala ndichifukwa ili yotseka

What is happening here is that had it been that am strong like I used to be in the past then it could not be closed it would have been opened so that i should be selling but because am not strong that's why i closed it, Sure, so I think that this hawker it was giving me maybe some other things in life without begging anything from my children if anything happens i was just getting money and pay but today i closed it and i depend on my children like in everything. That's why i just took the picture so that i should know that when i was strong it was open but now because am sick that's why it is closed.

05/01: Ndiye funso mwina lina lokhalangati tikupitiliza pang'ono ndilonena kuti ndichifukwa chiyani munajambula chithunzi chimenechi?

Maybe a question just a continuation continuing is that, why did you take this picture?

05/01: Chifukwa choti ndinajambula chithunzi chimenechi ndichoti okala imeneyi ndiimene imandithandiza chifukwa ndili ndekha ndi ana ana onnse anali ali pakhomo asanayambe ntchito koma chinachilichonse chimatuluka muti mmenemumu ndikumathandizila pakhomopa eya ndiyeno ndikutseka kwa okalayi chinachilichonse chinakhala ngati chatani chaima

the reason why i took this picture is that this hawker is the one that was assisting me because when I was alone all of my children were not working but everything was coming from this and assisting the family, sure. Now its like everything had paused because of the closure of this hawker e

I: Mufotokoze bwino bwino mukati chinachilichonse chaima mukuthandauza chani chifukwa timafunaso titaziwa kuti kodi chithunzichi chikufotokozera zotani zokhuzana ndi moyo wanu wa tsiku ndi tsiku

Can you explain better when you say everything has paused what do you mean because we also want to know what this picture explain to us about the life that you are living every day?

05/01: Chithunzi ichichi chikufotokozera moyo wanga wa tsiku ndi tsiku kuti ndimmene ndinalili kale ndimmene moyo wanga ulili panopa ndikusiyanisa ndizinthu zosiyana chifukwa kungoona okola yokhayo mmene ikuonekera ikuoneka kuti siikugwira chani siikugwira ntchito ikungokhala koma pa nthawi yoti ndili ndi mphamvu imagwira ntchito ndinkana ndinajambula chithunzi chimenechi kuti chizingondipasa chikumbumtima chotani chongoganizira kuti aah nthawi imeneyoyo ndikupanga bizinesi okala yanga siinali chonchi koma nditapezeka kuti ndadwala ndatha mphamvu ndipamene ikupezeka ilichonchi

This picture is explaining about my day to day life that the way i was in the past and now am comparing and there is a difference because just looking at the hawker the way it is looking it shows that it's not working it's dormant but the time when i was strong it was working that's why i took this picture so that i should be remembering that aah at that time when i was doing a business my hawker was not like this but because am sick am not strong that's why it is like this.

I: Mungatifotokozereko kuti mukanena kuti ndimmene munalili kale ndipanopa zikusiyana mungofotokoza kuti tikhale ndi chithunzithunziso kuti ndichithunzicho zikugwilizana bwanji kale mumapanga ziti pano zimene sizikutheka ndiziti

Can you explain to us that when you say the way you were in the past and now it's very different just explain so that we should have a picture that how relevant is this picture, what you were doing in the past and how its difficult now.

05/01: Kale ndimapita ku limbi kukaoda geni kubwelesa kusenza ndekha pamutu kufikisa pakhomo kusela kuyamba kugulitsa panopa sindingasenzeso geni kufikisa pakhomo mmapita mwinamwake ku southern bottlers kuja kukatenga zakumwa mwina ma crate atatu ndekha pamutu koma panopa sindingatani sindingapange

In the past i was going to Limbe to get things for my business bringing home and was carrying it on my head till home and start selling but now i can not carry the business on my head up to here i was going to southern bottlers getting maybe three crates of drinks alone carrying them on my head but now i can not make it.

I: Chabwino timafuna tingoziwako mbali imeneyo kuti ndithu moyo wasintha bwanji nde kumapetoko timafunaso tingodziwa kuti chithunzi chimenechocho okala yoti yangotsekedwa eti ikungokhala chikupeleka mwayi wotani kuti mwina pakhale mwayi onena kuti moyo wanu upite patsogolo ku dera komwe mukukhalako nditati anthu atati achione chithunzi chimenecho mukuona ngati kungochiona chingapeleke mwayi wotani woti mwayi uthandizire moyo wanu upite patsogolo

Alright we wanted to know about that issue to say how has your life changed and at last we also want to know that this picture or hawker has been closed right, its not working what chances does it give so that maybe their should be a chance that your life can be improved from where you are staying if we can say that people should see this picture you think that just seeing it can give a chance maybe a chance to help your life to improve?

05/01: Chithunzi chimenechichitu chikupelekabe mwayi woti mwina munthu wina atabwera wapadera eti kuti mwina azangopanga rent azindiptsa ineyo kangachepe iyeyo azipitiliza chani bizinesi kuti mwinamwake ineyo ndizitani ndizithandizika koma pa ineyo sindingakwanise eya

This picture is giving a chance that maybe if a person can come and rent it and should be giving me a little something and they should be doing business so that maybe i should be assisted but for me i cannot manage.

I: Kuteleko munthu abwere akhoza kupangamo rent?

It means if a person can come, will rent it?

05/01: Mmm

Mmm

I: Nanga anthu ena atachiona mukuona ngati chingawapase uthenga wotani umene ungapise moyo wanu patsolo

Now if other people see it what message can it give them that can make your life to improve towards your future

05/01: anthu ena atachiona ndimmene chikuonekeramu kupititsa patsogolo kwake ndikovuta nangasi ikuoneka kuti ndiyonyasa siikuoneka bwino pamene ikuonekerapo kuthandauza kuti olo kupentedwa inapentedwa pakanthawi ikungotani ikungokhala komabe ineyo ndingolankhula kwa anthu amene atani mdziko lathu la malawi lino kuti olobe mmene ndikukumana ndi mavuto osiyana siyana a matenda monga khansa ngatino tilipa bizinesi imene ndimachitila tizikhalabe ndichikhulupiliro kunena kuti izizi zachitika chifukwa cha matenda komanso mwina tsiku lina ambuye azatikomera mtima zizachitika kunena kuti azatipatsa mphamvu tizasegulaso tizabwelera mmene tinalili kale

if other people see it with the way it is looking, it is very difficult to improve it because it is not looking good the way it is looking it shows that it was painted long time ago it's not working but i can just speak to people that who are Malawians that even though I am facing different problems with the cancer disease if we are on business as i was doing we should be having hope that this has happened because of the sickness but maybe one day God will guide that we will be strong again and we will be ok the way we were before

I: chabwino tathokoza

Okay thank you

I: ndiye tipite ku chithunzi chachiwiri eti

Now we should go to the second photo

I: chithunzi china ndichimenechi mayi wanga mukutha kuchiona bwino bwino eti

The other picture is this one; I think you are able to see it clearly right

05/01: mmmm

Mmmm

I: chabwino chimenechocho mungatifotokozele kuti fotokozani chithunzi ichichi kuti munajambula

Alright that one can you explain about this photo you took

05/01: chithunzi chimenechocho ndinajambula ambuye yesu ali pakhoma mmenemo ndi mnyumba tsiku lina sindinadzuke bwino nde ululu umene ndimaumva ine ndinaziganizila kuti ineyo moyo wanga mmene ndikumvelera ndikulephera kudzuka ndikulephera mwinamwake kupanga kenakake pakhomo pano koma ineyo ndizapeza bwino ndiyeno ndili mkati molingalira ndinaganizila kuti aah inetu ndikotheka kuti ndizatani ndizapeza bwino chimozimozi ambuye yesu anakumanna ndizokhoma padziko lino lapansi koma iye sanataye mtima nde ineso ndisataye mtima ndikhale ndi chikhulupiliro choti kuti tsiku lina ambuye yesu yemweyo azandikhuza ndipo azaandichilitsa ndinkona ndinajambula chithunzicho

i took this photo of jesus christ on the wall in my house i did not wake up well the other day and the pain i was feeling i was thinking that my life the way i am feeling am failling to wake up am failling maybe to do something at home but will i be fine again then when i was still thinking i thought that it's possible for me to be well again the same as jesus christ met alot of difficult times on earth but he did not loose hope then i should not loose heart i should still have hope that one day the same jesus christ will tuoch and heal me that's why i took this photo

I: chabwino ndiye tinganene kuti chinthuzicho munango ndichopachikidwa mnyumba eti changokhala ngati chithunzi chimene chiimakongolesa mnyumba

Alright can we say that this picture you just i hanged in the house right it's just like a picture that beautifies in the house

05/01: eyaa chili mnyumba

Yes it is in the house

I: chabwino nde ndikupitiliza zokamba zathu khalangati mwafotokoza kale chifukwa chomwe munajambulira komaso mwafotokoza kuti chikuchitika ndichiyani nde ndimafuna tingodziwabe kuti kodi chinthunzi chimenechichi chikupeleka chikutanthauza chiyani zokhuzana ndi moyo umene mumakumana nawo tsiku ndi tsiku

alright now i am continuing with what we are talking maybe you have explained already the reason why you took this picture that what is happening then i wanted to know that what does this picture mean about the life you are meeting on day to day

05/01: chithunzi chimenechichi chikupeleka mwayi woti monga ifeyo anthu amene tikudwala matenda a khansa kuti tikhale anthu achikhulupiliro kuti tikhale anthu achikhulupiliro kuti olo tikupezeka ndi matenda a khansa ena akuti samachilitsika ena akuti amachilitsika koma ifeyo tingopanga chinachilichonse tingompasa ndani mwini wake mwini wake ndiwokutha chinachilichonse chifukwa iye satani salephera eya ndinkana ndinajambula chiti chithunzi chimenechocho

this picture is giving chances like we the peoplle who are suffering from cancer so that we should be people with hope that although we have cancer others are saying they don't heal other's say we will be healed let's do anything let's give it to the owner he knows averything because he never fails yes that's why i took this picture

I: chabwino nanga kumbali yonena kuti chikufotokozera moyo wotani umene inuyo mumakumana nawo tsiku ndi tsiku mukachiona chithunzi chimnechocho mungatifotokozele kuti mumakumana ndi moyo wotani tsiku ndi tsiku?

Alright to the fact that it is explaining what life you are leaving day to day when you look at this picture what can you explain the life you are meeting on day to day life?

05/01: chithunzi chimenechochotu umoyo wa tsiku ndi tsiku umakhala ovuta kwambiri

On this picture my day to day life is very difficult

I: mukati ovuta kwambiri mukutanthauza kuti chani?

What do you mean when you say it's very difficult?

05/01: ifeyo monga anthu adziko lino lapansi eti timakhoza kunena kuti mmene tikudwala mthenda imeneyi ya khansa chonchi mmalingaliro mwathu ndikumalingalira kunena kuti inetu zanga pamenepa zatani zada mmene zatelemu komano zinthu sizili chonchoyi apapa ndi mmene akuonekera ndi yesuyo ndiwoti chinachilichonse anasintha ndipo chinachilichonse anakumbusa ndinkona ndinajambula ambuye yesuwo kuti ndizikhala ndi chikhulupiliro cholimba kunena kuti aah chinachilichonse ndichotani ndichotheka ngati ndimalephera mwinamwake kukatumikira izafika tsiku ndizakatani ndizakatumikilaso

we as people from this earth right we can say that we are suffering from cancer now what we think is we think that everything for me is dead but things are not like that this is how they can see with jesus everything is changed and everything he reminds that's why i took this picture of jesus christ so that we should be having strong hope that aah everything is possible he never fails maybe if we follow time will come we will do things again

I: chabwino palinso china chimene mukufuna mufotokoze zokhuzana ndi chithunzi chimenechi?

Alright is there anything you want to tell us about this picture?

05/01: ineyo kufotokoza kwanga ndikoti monga ifeyo anthu amene tikudwala makamaka tingokhala ndi chikhulupiliro choti chifukwa ululu wambiri ndetikukumanawo koma ngati tikutaya chikhulupiliro tiyenera kuti tilichabe koma ngati tili ndi chikhulupiliro cholimba ululu uja timamva ululu uja nthawi tikamwa mwina panado uja umasiya pomwe ambuye yesu zimenezo sanakumane nazoyi anafikana kufa pamtanda alimkati mwachani kodi mwaululu eya ndeno chithunzi chimenechi chikufotokoza kunena kuti tikhale anthu olimba mtima ndiokhulupilira komanso ndifotokozera dziko lonse lachani la malawi kuti mmene anthufe tikudwala matenda a khansa atithandize pamodzi ndi madokotala kuti mwinamwake mankhwala ngati kuli kotheka apezeke kuzera mwayesu khristu yemweyo awapase nzeru awapase mphamvu awunikire bwino bwino kuti matenda awawa tiyenera kugonjetsa mwakuti mwakuti

the way i can explain is like we people who are suffering mostly we should just have hope that because we are feeling alot of pain but if we loose hope we are nothiing but if we have strong hope the pain we feel when we take panado it stops where jesus did not meet those on the cross when he was in deep pain yes now this picture is explaining that we should be people with strong hope but also i will explain to the whole country of malawi that we people suffering fromm cancer they should help us together with the doctors so that maybe the medicine if posible should be available through jesus christ he should give them wisdom give them power to check properly so that we should finnish so so so

I: chabwino nde mukuzafotokozaso kuti ngati tilibe chikhulupiliro pemepo tili chabe mungafotokoze kuti pamenepo mukutanthauza chani?

Alright so you are also explaining that if we don't have hope we aare nothing can you explaain what you mean?

05/01: chikhulupiliro ndichinthu chimodzi chofunikira chifukwa choti ngati tilibe chikhulupiliro ndiyekuti olo mulungu mwini wake palibe chimene angatipase chifukwa choti tikukaika kaika koma ngati ifeyo tikuima kwa yesu khristu kungatalike kungachedwe kungafupike koma tsikulo likangokwana kuti azatipase machilitso azatipatsa

hope is one important thing because if we don't have hope even God him self there is nothing he can do because we aare doubting but if we stand for jesus christ how long how late how short it maybe but if the day will come to heal us we will be healed

I: chabwino palinso china choti muonjezere?

Is there anything that you can add?

05/01: ayi ineyokuonjezera kwanga ndiku kungoonjezera kuti anthu amene ammalawi muno ndimadokotala atithandizile matenda amenewa komanso kafukufuku wakeyu akhale opambana chifukwa choti kwabwera makafukufu

No what i can add is those people inn malawi and the doctors should help on this disease and also this reaserch should be productive because there are alot of surveys that came in

I: chabwino tathokoza kwambiri

Alright thank you very much

05/01: zikomo

Thank you

05/01: nde musankhe mmanja moti tikuombeleni

Now you can choose how we should clap hands for you

05/01: mwachifumu

For the chiefs

I: akuti mmanja mwachifumu mumaziwamo

She is saying clapping hands for the chiefs

atengambali kuombera mmanja

Participants are clapping hands

I: kukhalangati kamuzu banda

Just like kamuzu banda

THIRD PARTICIPANT

I: Paja dzina ndindani

What is your name?

08/02: (name)

(name)

I: (name)?

(name)

08/02: (name)

(name)

I: (name) chabwino mayi (name) tifotokozeleni zachithunzi chimene chilikutsogolochi

(name) Alright miss (name) can you explain to us about that picture which is in front

08/02: Owo amene ndawajambulawo ndi amuna anga tinakhala kwathu mwendo unayamba kutupa utayamba kutupa ndinapita kuchipatala ku chilomoni kukafotokoza kuti tabwera ndivuto ili mwendo ungotupa okha amene nde ataugwira gwira madokotala aja ananena kuti ayi nthenda sitikuitani sitikuiona bwelelani nde tinabwelera muzabwere tsiku lina nde date lina litakwana tinabweranso kuchilomoni komkuja tinapita kuchilomoni konkuja anazatilembera chani kalata kuti panopa muyenera kuti mupite ku queens mukaonebe kuti kumenekoko nde ndinapita ku queens kuja date itakwana titapita tinapasa adokotala nde ananena kuti ayi muzabweleso tsiku lina tengani pepala lanuli nde ndinabwelelaso kumuzi nde date lija litakwanaso tinatani tinabwelera kuchipatala kuja nde nditabwelera kuchipatala kuja mmene pankandiuza iwo kuti ataiona vutoli kuti vutoli ndichani ndi khansa nde anandipasako mankhwala panado ndi mankhwala ena akeso

OK I took a picture of my husband we were at home when his leg started swelling when it started swelling we went to chilomoni hospital where we explained that we have come with a problem of the leg it is just swelling so after the doctor examined it they said they don't see any disease you can go back and come again next time when the **date** came we went again to chilomoni and they gave us a referral letter to go to queens so that you should be examined and when the date came we went to queens and gave it to the doctor and he said no you should come another day get your paper and we went back home and when the **date** came we went again to the hospital then when we went back to the hospital it's when they were telling us that this problem is cancer the they gave me panado and some other medicine

I: Chabwino

Alright

08/02: Okhala ngati capusozi koma ali a gilini

It was like capsules but in green color

I: Chabwino

Alright

08/02: Ndinabwerako titabwera kunyumba abambo anakhala anali ndichani anali ndi nkhawa nde anangokhala zawo phee

we came back when we get back home my husband sat down was very worried that's why he was very quite.

I: Ndipamene munawajambula

It's when you took him a picture?

08/02: Ndipamenepopo apopo ndinangowajambula malinga ndipanthawi imene ndinatani tinakumananawo tsiku limenelolo chithaandizo mmene ankachiyendeselamo osapeza chithaandizo mwachani mwachangu vuto limene analinalo pathupi pawo abambowo

Now there i just took a picture of him that day just a remembrance of what happened that day, the way the process to be assisted was like, not finding assistance quickly with the problem that was on my husband’s body.

I: Chabwino

Alright

08/02: Kuti ndiziwajambula pamenepopo

That's why i took a picture of him.

I: ndiye apopo akupanga chani pachithunzipo angokhala

Now what is he doing on the picture just sitted

08/02: pamenepo angokhala akuugwira mwendo wawo uja eya

There he has just sitted touching on his legg yes

I: chabwino chimachitika ndichani pamene amagwira mwendo amamva kuwawa chani chimachitika ndi chani

Alright what was happening so that he should be touching his legg was he feeling pain what was happening

08/02: chimachitika pamenepopo kuti azigwira mwendowo umatabwanyika ngati nthochi eee nde amasiyanisa ndi mwendo winawo ndi uwowo watupawo

What was happening here was that he was pressing on his legg which was doing like a banana eee then he was compairing with that one which is ok

I: chabwino mayi chabwino

Alright woman alright

I: ndiye mwina ndinene kuti chifukwa chani munajambula chithunzi chimanechichi

Now let me say this why did you took him this photo

08/02: kuti ndiziwajambula chithunzi chimenechocho malingana ndikuti tinayendera kupita ku chipatala ku chilomoni kuzafika mpakana ku queens kuzafika leloro kuzayamba kutiuza vuto limenelili lapezekali eti kuzayambaso kuwapasa mankhwala kuzamwa panopa nde ndinangokumbukila nthawi imene ndinkatani tinkayendera pa tsiku limenelolo nde ndakumbukano kuti apapa ndiyenera kuwajambula kuzafika tsiku la lelolo ndichisomo kuti ayambe kutani kulandila mankhwala chifukwa vutoli tinalitani talidziwa chifukwa panthawiyo sitinkaliziwa

for me to take him this picture with the way we have been going to chilomoni hospital upto going to queens until today when we were told about the problem that is found now yes and started receiving the medicine then i just remembered that on that time i was going to the hospital so i just remembered that am supposed to take him a picture reaching today is just by grace to start receiving the medicine because we now know the problem because at that time we didn't the problem

I: chabwino nde chithunzi chimenechichi chikuonesa kuti aah bambowa amakumana kapene inuyo mmene mumawasamalira mumakumana ndi moyo wotani tsiku ndi tsiku pokhala ndi amene mukuwasamalira amene akudwala matenda a khansa inuyo mukukumana ndi moyo wotani chithunzi ichocho tikachiona chingatifotokozele zotani kuti inuyo mukukumana ndi moyo wotani tsiku ndi tsiku

alright now on this picture it's showing your husband that aah this man was meeting or the way you takes care of him what kind of life do you meet on day to day leaving with the person you are taking care of the one who is suffering from cancer what kind of life are you meeting on this picture if we can look at it what can it explain to us on the life that you are every day

08/01: apapa ndinene kuti kukumana ndi zovuta palibe chifukwa choti ineyo awawa ndimawasamala mmene amakhalira nthawi zonse ndimakhala ndikuwmeeting evalimbitsa mtima kuti apapa musataye mtima ndipo musadandaule china chilichonse ife tizingomupempha ndani mulungu yemweyo chifukwa m'baibulo ananena kuti kuzabwera matenda osiyana siyanande matenda osiyana siyana akutanidi akuchuluka tingoyenera kuti tizingopemphera mulungu palibe chomutani chomulaka nde ndipamene nditani ndikuwasamalira amunanga patsiku limenelolo

here let me say that i don't meet with the problems because i take care of him the way he is everytime i always incourage him that he should not loose hope and don't worry in everything we just need to ask God because in the bible they say there will be alot of diseases coming and they are really coming we just need to be praying to God there is nothing that can fail him its when i was encouraging my husband on that day

I: moyo umene inuyo mumakumananawo tsiku ndi tsiku ndikaona ndichithunzichi ndi moyo wotani?

So the life that you are meeting on every day if we look at this picture what is such life?

08/02: ineyo

Me?

I: eee

Yes

08/02: ineyo chithunzicho chifukwa chakupezeka iwo anthu ambiri ndinene kuti azibale anga kapena azineba amakhala kumandinena kuti vuto ili iweyo ukuyenekera kuti amuna ako uwathawe kuti ndimetanda opasilana iweyo kuti uziwasamala utani kodi utengera koma ineyo chikhulupiro changa ndinanena kuti ayi ngati munthu wapezeka vuto ali ndi ine ndiyenekelaso ndisale ndisamale matendawa chifukwa chokuti ankandifunsira analibe chani panalibe vuto ilili ineyo ndiyenera kutani kuwasamala pazomwe anatani anakhala iwowa ndimathero a moyo wawo mmene mulungu wafunira khaya ndine wandipanga kukhala nawo limodzi

On thIS photo because i found a lot of people let me say my relatives or neighbours they were saying that I am supposed to leave my husband that he has transimitted disease if you will be taking care of him you will contract them but i said no with my beleif if a person is found with a problem while with me am suppose to stay and take care of the disease because the time he was proposing me he did not have this problem am suppose to take care of him with what he has till his death the way God wants because he made me to stay with him

I: chabwino ndiye chikupeleka mwayi otani kuti moyo wawo utha kupitisidwa patsogolo chithunzi chimenecho chingapereke mwayi wotani kuti pakhale pakhoza kupezeka thandizo linalake lopitisa moyo wawo patsongolo

Alright now what chances does it give so that your life can be improved what can this picture give chances so that there can be a certain help to improve your life

08/02: mmmm ineyo zimene ndipempha ine ndifunano ndikupempheni inuyo madokotala chifukwa pali mamkhwala amene akulandila pakanali pano muwapitilize kuwatani kuwapsa ndipo ngati alipo ena opyolera pamenepopo ofunika kuwapasa amene akudwala matenda awawa kuti tione kuti kuchira kwawo

mmmm with me what I’m asking is that i want to ask you doctors because there are medicines that they are receiving currently you should continue giving them if there are others more than these ones your also suppose to give those that are suffering from this disease so that they can be healed

I: chabwino

Alright

08/02: komanso tingoonesesa kuti ifenso amene tikusamala matenda ndizambiri zimene timanyozedwa ndi mazineba ndizambiri ngakhale abale athu ngakhale makolo athu amene amatha kunena mau wotani omunyoza munthu akudwala uja nde tisakhale ifeyo okhumudwa kuti munthuyo timutani timuthawe tiyenera kumusamala

but we should also make sure that we the ones who are taking care of the patients there are alot of things that neighbours talk bad us even our relatives or parents the ones that can say bad about the patient but we should not be worried so that we should live the patient we just need to help them

I: chabwino tikhoza kupita ku chithunzi china

Alright we can go to another picture

08/02: eee

Yes

I: chabwino chithunzi china chinthunzi chinaso ndichimenecho mungatifotokozere kuti chithunzi chimenechi pali ndani?

Alright another picture this is another picture what can you explain tht who is on that picture?

08/02: amenewaso ndi amuna angawa tsiku limenero mwendo wawo umawapweteka akuti umaotcha nde mkati mowaotcha anandiuza kuti ndiwapase chani mankhwala ndinayamba kuwaphikira phala adya kenako ndakawapasa madzi asamba ndiye atabwera kubafa kuja ndinawayalira mphasa kuti mukhale apa ndatenga mankhwala ndatani ndawapasa mmene mukuona mmanjamu munali mankhwala awowo ndi madzi kuti atani amwe

This one is also my husbandhis leg was full of pain while feeling piain he told me that i should give him medicine i first started cooking poledge for him then i gave hime water to bath now after finishing bathing i put a mat for him to sit on then i took the medicine the way you can see in the hands there was hs medicine and water so that he should drink

I: chifukwa chani munajambula chithunzi chimenecho?

Why did you take this picture?

08/02: chifukwa chakuti apopo ndajambula chifukwa choti munthu ayenera kuti akadwala tiyenera kumpasa mankhwala kuti amwe chifukwa umoyo wa awawa alinawo panopa ndi mankhwala nthawi zonse tiyenera kumpasa munthu amene akudwalayo kuti aapeze chani chithandizo chamankhwalawo kuti awone kuti zikhala bwanji malingana ndi matenda amene akutani akudwala ndimmene ndinawajambula chithzicho

because there i took it because a person is supposed that when sick we are need to give them medicine to drink because there life depends on medicine we need to give everytime to those who are sick so that hey get help from the medicine so that they should see how it is goinng to be with the disease that they are suffering from it's when i took this picture

I: nde chithuni chimenechocho chingationese inuyo ngati osamalira odwala moyo wanu wa tsiku ndi tsiku ndiwotani?

What can this picture show us about how your every day life is as the one taking care of the patient?

08/02: apapa moyo wanga poyambapo mmene ndanenera kuti ndinali ndi nkhawa ndipo ndingakhale ine mtima wanga umandipweteka koma panopa nditamutenga mulungu wanga panopa moyo wanga unakhazikika bwino bwino sindikuderaso china chilichonse chifukwa ndimangolimbika kupemphera kuti ambuye mwina atha kunditai kundithandizira komanso kundikhazika mtima wanga mmene unatani unalili poyamba ndimmene anandilengera mulunguyo usakhaleso odandaula china chilichonse iyeyo ndi amene amakwanisa china chilichonse mulungu yemweyo

here my life in the first place the way i have said that i was worried and i was feeling pain in my heart but now after taking God my life is now ok am not worried because i pray hard so that God help me and also my heart the way it was and the way God created me not be worried in anything he is the one who fulfills everything the same God

I: chabwino

Alright

08/02: nde basi sindimadandaula china chilichonse

Now i don't get worried in anything

I: nde mwati moyo wanu panopa ndiwokhazikika mungatambasule kuti mukati kukhazikika mukuthandauza kuti chani?

You have said your life now is strong can you explain what do mean when you say it is strong?

08/02: owoo ndikutani ndikuthandauza kunena kuti poyambapo ndinali ndi nkhawa ndikangoona mwendo wa amuna anga mmene unalili nkuona winawo moyo wangawu unali ndi mantha kuti vuto ilili lafika abambo awawa kodi ndingaikwanise mwana ngati ine kodi chifukwa kumudzi kwathu analiposo ena anadwala nthenda imeneyiyi eti ndimmene tinawaonera timawatcha ankolo anga eti ndimmene ndinawaonera amazunzika mpaka anatsikira kuli chani kuli chete komma ineyo ndinangomupemphabe mulungu pafupifupi ndinkangopemphera kuti ambuye muthe kukhazikitsa mtima wanga zimene ndikulingalira ine zisakhale ziti zimenezozo koma inuyo muthe kukhazikitsa mtima wanga ukhale mmene utani unalilli amba poyamuna anga asanatani analibe vuto ilili

Okay, i mean that at first i was worried when i see my husbands leg the way it was and seeing the other one i was afraid that this problem is with the husband am i going to manage because in our village there was someone who was also suffering from te same disease we were calling him uncle right with the way i saw him he was very troubled and now he is dead but i just asked God that i was almost praying that God should be with me so that what i am thinking should not be like that but yu should take my heart to be the way it was before he has this problem

I: ok

Okay

08/02: eee

Yes

I: chabwino ndiye mwina kumapeto chithunzi chimenechocho chikupeleka mwayi wotani kuti moyo wa tsiku ndi tsiku umene inu mumakhala nawo posamalira odwala kuti upite patsogolo?

Alright now maybe at the end what chances does this picture gives your day to day life when taking care of the patient to improve?

08/02: tabwelezani?

Can you repeat?

I: chithunzi chimenechocho eti chili apocho chingapeleke mwayi wotani kuti mwina pakhale thandizo linalake kuti moyo wanu wa tsiku ndi tsiku upititsidwe patsogolo?

This picture right that one there what chances can it give so that maybe there can be a help so that your day to day life can be improved?

08/02: wangawu wa abambo?

Mine for my husband?

I: wa inuyo tsopano

your's now

08/02: owoo

Okay

I: chifukwa abamboso ndiwafunsa chithunzi chanu

Because i will ask your husband your picture

08/02: owoo zikomo

Okay thanks

I: eee kaya munena mbali zonse koma yambani kaye mbali yanu

Yes you will say both sides but start with yours first

08/02: eya ineyo ndaiwala talibwerenzeni ujeni

Yes i have forgoten can you repeat the

I: ndimmati chithunzicho eti tiyelekeze ife tangochiona chithunzicho chingatipase mwayi otani onena kuti pakhale mwina mwayi onena kuti moyo wanu upite patsogolo ndichiithunzi choti chikupeleka uthenga onena kutino mwina mwake zinthu zisinthe kuti moyo upite patsogolo uthenga umene ulipo ndi uthenga otani?

I was saying that picture right let's say we have just seen the picture what chances can it give us so that maybe chances that your life can improve with the picture with the message to say maybe things to change so that your life can improve what message is there?

08/02: mmmm uthenga okuti ife oyang'anila odwala ife munthu akanena kuti tikumusamalara odwala uja tiyenekera kumupasa mankhwala amwe kuti tione kuti munthu akhala bwanji malingana ndimmene vuto lomwe apezeka nalo

Mmmm the message is that we who takes care oof the patient when a person says that we are taking care of the patient we are supposed to give them medicine so tht we should see how they are going to be with the problem they have been found with

I: chabwino tathokoza tikupita chithunzi chinachomaaliza eti palinso china chofunika muonjezere ngati?

Alright thank you we are now going to the last picture is there anything that you can add on?

08/02: ayi palibe

No nothing

I: chabwino chithunzi chimenechichi eya fotokozani

Alright explain on this picture

08/02: uyoyo ndi mwana wanga tsiku limenelo anapita kumadzi nde abambo akewa nthupi mwawavuta nde anamuuza kuti takondwa utenge mankhwala patebulopo undipatse nditani ndimwe nde mwana uja anatenga madzi ndi mankhwala aja kupita kunali bambo ake aja kuti

This one is my child on this day she was going to draw water then her father did not feel well then he her that takodwa get the medicine at the table and give me to drink then the child took water and medicine and went where her dad was to

I: dzanja linalo

The other hand

08/02: eee uyoyo ndi LA

Yes that one is LA

I: eee

Yes

08/02: awowo ndi mankhwala eti

Those ones are the medicine right

I: ohoo chabwino

Oh okay

08/02: nde amapanga abambo ake aja kuti izi amawafunsa eti

Then she was telling her dad that these ones she was asking him

I: owoo

Okay

08/02: nde amanena kuti eee nde amapita kuti abambo ake aja akawapasile kuti akataani akamwe nde kamaoneka pamoto paja ndipoto nthawi imeneyo tinali titateleka phala kuti azatani kuti awapase akachoka kumadzi kuja amwe ndiye ndinapeza kuti phala lija apungula okha amene kuti atani adye ndiye mmene ndinabwera nde mwanayu ananena kuti ndatenga mankhwala ndawapasa ndani adadi nde ndinanena unapanga bwanji nde pamenepo anali akusayiza nde ndinati tayima apa ndikujambule ndimmene mukuona chithunzicho

Then she was saying that yes she was going to give her dad so that he should take them then that one on the fire is a pot at that time we were cooking poledge so that she should give him when she is back from drawing water so that he should take then we found that he has already taking the poledge so that he should be eating he got it himself then when i was coming the child was saying i gt the medicine and give them to dad then i said how did you do now there she was showing the size then i said stand here i should take you a picture it's where you can see this picture

I: mmmm nde chifukwa chani munajambula chithunzi chimenechi?

Mmmm now why did you take this picture?

08/02: mmm chifukwa choti mwanayi abambo ake amamukonda nayeso mwanayi abambo ake amawataniso amawakonda chifukwa anakakhala mwana wina anakakana kuwapasa chani mankhwala koma anaziwa kuti abambo ake akudwala ndinkana anathamanga kuwapasa chani mankhwala

Mmm because her father this child love and she also love her father because if it was another child she could have refused to give him the medicinebut she knew that her father is sick that's why she gave himm the medicine fast

I: chabwino ndiye chithunzi chimenechocho chikuonetsa kuti moyo wa tsiku ndi tsiku umene inuyo mumakumana nawo ngati osamalira odwala ndiwotani ?

Alright what life is this picture showing about how you live every day as the one who takes care of the patient?

08/02: panopa ineyo ndi mmene ndafotokozera kuti moyo wanga uli bwino bwino nkhawaso ndilibe

As i have already said that my life is ok and i don't have any worries

I: angakhale mwana akuthandiza nawo

Even the child is helping

08/02: eyaa

Yes

I: ndizimene mukutanthauza

Is that what you mean?

08/02: eee mwana wathu akuthandizila kuwapatsa ndani bambo wake akuziwa kuti adadi ake akutani akudwala ndichifukwa chake akuwapasa mankhwala kuti atani amwe

Yes our child helps to give his father she knows that her dad is sick that's why she is giving him the medicine so that he should drink them

I: chabwino nanga mwina mwayi chithunzi chimenechi chikupeleka mwayi wotani kuti zinthu zingasinthe

Alright maybe what chances does this picture give about things that can change?

08/02: kwa mwanayo?

To the child?

I: pakhomopo inuyo ngati a gadiyani ngati oyang'anira odwala kaya ndikwawina aliyense oyang'anira odwala ife tikudziwa inuyo ngati oyang'anira wamkulu odwala eti titati tichione chithunzi odwala mwana watenga madzi ndi mankhwala akukawapatsa bambo wake mukuona ngati chingabweletse mwina maganizo mwathu kuti aah koma zakuti zisinthe mukuona ngati zingabweretse moyo wotani

At the hous eyou as the one who takes care of the patient or to anyone else looking after the patient we know you as the one taking care of the patient if we can see the picture the patient the child has taken water and medicine to give her father what do you think it can bring maybe in our thinking that aah but these should change what do you think they can bring what kind of life

08/02: apopo tinganene kuti kaya mwina molakwika eti mungaone ndiinuyo apopo kwamwanayo chifukwa sizingadziwike bwino bwino chifukwa chokuti chani bambo sakuoneka eti mtima wamunthu maganizo a wina azayesa ngati mwanayo afuna atenge mankhwalawo atani amwe kapena akusewelesa eti ndimmene zilili apopo koma ine ndingotha kunena kuti ndingonena kuti mmene zililimu tiyenera kuwapasa amene akudwalawo mankhwalawo tisamangowasunga mankhwalawo tiyeneka titenge mankhwala tiwatani tiwapase

There we can say that maybe you can see right you can see there to the child because it can not be known properly because the father is not seen well right and the heart of a person and there thinking they will think that the child want to take the medicine to drink or she is playing with them right with the way it is there but i can say that with the way they are here the one who is sick we should not just be keeping the medicine we should be giving them

I: chabwino

Alrigh t

08/02: eya

Yes

I: tathokoza

Thank you

08/02: zikomo

Thanks

I: chithunzi chomalizatu

This is the last picture

08/02: eee

Yes

I: palinso china chomwe mukufuna muonjezele?

Is there anything you want to add on?

08/02: iyayi

No

I: chabwino

Alright

I: ndiye musankhe mmanja kuti tikuombereni

Now choose the way you want us to clap hands for you

08/02: iyayi mwachifumu

No for the chiefs

I: mwachifumu mwa ngwazi?

For the chiefs like ngwazi

I: akuti tiombe mmanja mwachifumu

She is saying that we should clap hands for the chiefs

Atengambali kuombera mmanja

Participants clapping hands

FOURTH PARTICIPANT

I: Chithunzi china ichi anajambula ndindani?

who took this picture?

07/02: Ndi ine

It's me

I: Owo ndinu mami eti?

Okay, it's you mumie right?

07/02: Mmm

Mmm

I: Chabwino mmene tapangira ndi ena aja mafunso ake ndiomwewa eti nde koyambilira mutifotokozere chithunzicho chimene tikuchionacho

Alright the way we have done with others the questions are the same right, at first explain to us about this picture we are seeing

07/02: Chithunzi chimenecho eti ndi amuna anga ndimawajambula pamenepo chifukwa chakuti ndikumene ndachokera nawo matendawa eti ndazungulira muzipatala ndagonetsedwa gonetsedwa kuti adzafike point yopeza nthendayo kuti khansa eee nditagudubuka nde pomafika point yoti anazuka ndikumagwira ntchito okha nchifukwa ndinawajambula chithunzicho

That is my husband on the picture, i took this picture because from where we are coming from with his sickness right i have gone around hospitals, have been admitted to reach the point diagnosing this disease that it is cancer, you see, it was after passing through a lot so to reach this point that he woke up and working on his own that's why i took this picture.

I: Chikuchitika ndichani pachithunzicho?

What is happening on this picture?

07/02: Chithunzicho pamenepo kuti akutanganidwa okuti pamenepo afikapa akugwira gwira pamenopo mmene akugwira gwira pamenepo kusonyeza kuti moyo wangano ineyo ukutani kodi ukumasangalala kuti paja anafikapa afika mpakana kumazigwilira okha ntchito

He was busy on that picture that has reached this point of working so while he is working it just means that my life is happy that, the condition he was but he is working on his own now.

I: Mmm apopo akugwira ntchito?

mmm is he working there?

07/02: Pamenepo akusanja foundation ya nyumba

He is working on a foundation for a house

I: Aaha

OKay

07/02: Eee

Yes

I: chabwino mwafotokoza kale mafunso ena atatuwo nde tifunse kunena kuti inuyo ngati woyang'anira abambowo eti moyo wanu wa tsiku ndi tsiku tikaona chithunzicho moyo wanu wa tsiku ndi tsiku ndiwotani?

Alright you have explained on other three questions now let me ask this way as a person who takes care of this man right on your every day life on this picture what can you say about your day to day life?

07/02: mmm moyo wa tsiku ndi tsiku ngosaneneka kuti kunena kuti moyo wa tsiku ndi tsiku munthu amene ukuchiza matenda ukusamalira matenda ungakhale moyo osangalala iyayi unali moyo wamavuto chifukwa mavutowo nde ndi ambiri ndingofotokoza awiri chabe chifukwa chakuti kusalidwa kumakhalapo pokuti kumafika pokuti amene aja angadzachire kodi akati khansa mesa khansa ndi edziso imakhala thupi limodzi okuti ayi azinneba ndikumadusa kuseli kwanyumba

Mmm i can not say about my day to day life that my day to o day life as the one who is taking care of the patient can be a happy life no it was a life with problems because the problems are too many i will just explain two of them because there was discrimination up to the point that the y were saying that is he gooing to be well we thouhgt if they say cancer and AIDS the goes together that neighbours when passing behind my house

I: chabwino nde amayi ndimafuna kuti muone chithunzicho mmene chikuonekera chithunzicho mmene chikuonekera kuti kodi chikupeleka uthenga wotani okhudzana ndi moyo umene inuyo mumakumana nawo tsiku ndi tsiku ngati amene mumawayang'anira bambowa eya mwatchula zosalidwa mwina zimenezi zinali kwambiri koyambilira

Alright now i want younto see at this picture the way the picture is looking like what message is it giving about the life that you are meeting on every day as a person who takes care of the man yes you have metioned about discrimination maybe these were at the beggining alot

07/02: eya koyambilira

Yes at the beggining

I: eee komano mukachiona chithunzicho panopa chikupeleka uthenga wotani za mmene moyo wanu ukukhalira wa tsiku ndi tsiku ngati amene mumasamalira odwala matenda akhansa

Yes but if you can see it what message does it give with the way you are leaving every day as th one who is taking care of the person who is suffering from cancer

07/02: moyowo panopa khalangati kuti ukusinthilapo ndizokumana nazo kusiyana ndi nthawi imeneyo eeh anali woti samatha kudzuka samatha kutani pamene afika point yomatero moyo ukukhalano wosinthika ndithu kwa tsiku ndi tsiku zumakhalano kuti malingaliro ndi zoziwona eti nkumati aah bolani pano

The life is now abit changing with what we are meeting now is different with the past yes he was not even woking up doing what the point he is now life has changed in every day it is like when thinking and what we see and saying better now

I: bolani pano eti

Better now right

07/02: mmmm

Mmmm

I: chabwino ndiye chikupeleka mwayi wotani woti pangakhale kusintha pa moyo wanu

Alright now what cances does is it giving so that there can be a change in your life

07/02: apopotu pamenepa moyo wangono apopo mwayi kupelekedwa ndikovuta ndingopereka kwa inu adokotala kuti ngati munthu atati wakumana ndi vuto asamafulumire kukhumudwa kapena kuzilamulira mwa iyeyekha kuti akangopanga mwakuti ndilongo likufunikira kulimbikira kupita ku chipatala ndikumene angapeze thandizo loyenelera ndikufika point yoyi mulungu amazatani kodi amazakonza njira popanda njira

On there on my life it's very difficult to give chances to you the doctor like a person saying has met a problem they should not rush to get worried or comanding on them selves that they should just do something it just need to be very hard on going to the hospital it's where you

I: kuti chikupeleka uthenga woti anthu azipita ku chipatala kuti akalandire chithandizo can get best help and there is a point that God makes a way where there is no way

07/02: kulimbikira ku chipatala ndichinthu chofunikira

It is important to be going to the hospital

I: chabwino palinso china chimene mukufuna kuonjezera tisanapite ku chithunzi china?

Alright is there anything that you want to add before we go to another picture?

07/02: ayi

No

I: basi eti

That is all right

07/02: eee

Yes

I: chinanso ndichimenechi chithunzi tifotokozeleni kuti pali ndani pachithunzi mmene munapangila mwinamuja

This is the other picture can you explain who is on the picture and why did you do the other way

07/02: ameneyo ndimwana wanga panthawi inoyo imene ndimakhala

That one is my child t this time i stay

I: mulankhuleko mokweza pang'ono kuti mau mwina alowe umumu

Can you say abit loud so that your voice can be entering in here

07/02: ameneyo ndi mwana wanga panthawi imene ineyo ndimachulukidwa ndikusamalira abambo akewa chifukwa abambo ake nthawi zina amatha kuyenda moti mwina miyezi iwiri itatu mwendowu ulibwino bwino koma uli chitupileni koma akuyenda nde umadzafika point ina akangoti eee thupi langali ndikumva kuzizira kwambiri ndekuti pamenepaja simuwagwira ndimatenda ndithu a serious ndeno ndikakhala ineyo ndikakhala oti ndikusamalira awawa iye uja ndiwamamuna koma amafika point yokuti kusukulu mwina kutsanzika kubwera akabwera apitilize zimene ineyo ndimatani ndimapanga apapa powonetsa kuti amayiwa alibeso mpata woti ayesera adadi aja kuwasamalira kuti apange izizi mukuonawo ndi mbwibwi wa kamba umenewo amamanga.

That one is my child on that time i hard a lot of work in caring for his father because his father would walk maybe for two or three months with that fine leg but still swollen but he was walking with it and it could reach a certain point if he says, “my feeling very cold that means he is seriously very sick then if it's me who is taking care of him then she these what you see here is kamba’s flour, he is packaging it.

I: owoo nde pamenepo chikuchitika ndichani pachithunzipo?

Owoo now what is happening on that picture?

07/02: pamenepopo chikuchitika ndichakuti akuthandizira zimene ndimachita nthawi imeneyoyo.

What was happening there is that he is assisting on what I was doing that time?

I: Amaphika?

He was cooking?

07/02: Ayi akumanga geni imeneyoyo kuti akagulise

No he is packaging that is a **gain** he wants to go and sell.

I: Owoo bizinesi akumanga bizinesi

Okay, a **business**…she is preparing a **business**?

07/02: Mmm

Mmm

I: Chabwino nde mwati munajambula chithunzicho kusonyeza kuti ndi amene amakuthandizani

Alright you have said that you took that picture to show that she is the one who helps you.

07/02: Amene amandisamalira kwambiri

The one who takes care for me a lot.

07/02: Chabwino nde chithunzi chimenechocho chikuonetsa kuti inuyo mumakumana ndi moyo wotani tsiku ndi tsiku pamene mukusamalira wodwala

Alright so what does this picture show about the life that you meet each and every day as a guardian?I

07/02: Moyo umene ndimakumana nawo wa tsiku ndi tsiku amayi (xxx) moyo wanga ndithu ndiwovuta ndimavuto osiyanasiyasana amene ndimatani ndimakumananawo komano mmalamoti munthu nde wamavutoyo singachite kuwavomelera mavutowo kuti ine ndiwovutika chonchi ayi pakutino ukatere mavuto amoyo wa tsiku ndi tsiku chifukwa chake timapanga izi ndi izi kuti kapena mwina tizikhala kuti mwina mavuto aja adzitani tizikhala ngati kuti achoka

the life that i meet on day to day life (xxx) my life is difficult and I meat different problems but instead you cannot accept the problems that I am poor, no since being a human being is having problems and since when you do this there are every day problems that is why we do this and that so that maybe the problems should be leaving.

I: tatambasulani pang'ono mayi mungoti izizizi mutambasule pang'ono kuti chithunzicho chikufuna chitionese kuti inuyo ngati amene mumasamalira odwala mukukumana ndi moyo wotani wa tsiku ndi tsiku mwina mupeleke chitsanzo chamavutowo mungotchula eyaa

Can you explain a little bit you are just saying these one explain a bit that the picture want to show us what kind of life do you meet everyday as a guardian and maybe you can give an example of the problems, mention them, sure.

07/02: Apopo ndimbali yakudzithandizira

That is about helping myself

I: Kuzithandizira

Self help?

07/02: kuzithandizira kuti munthu wadziyimira pawekha eti nde ukapezeka ndi vuto kuti ufike point yodalira kuti aah kapena tizinena kuti achimwene aja andithandizira sizingatheke chifukwa achimwenewoso alindizawo eti nde kumati iiih ndipanga bwanji ndili ndi ana nditani mkumapezeka kuti mmalo motino uphelere mavutowo ndipamene mwanayu akundithandizira

Assisting yourself as being independent right, so once you have a problem for you to reach a point of saying that aa or to say that the brother would help that cannot be possible because the brother has his own too right so you say that iii what can I do, I have children so what should I do so It is found that other than finding a solution to the problems its when this child is assisting.

I: Chabwino ndathokoza kumapeto chithunzi chmenecho chikupeleka mwayi wotani kuti mwina moyo ungasinthe pakhomo moyo wa inu amene mukusamalira odwala tsiku ndi tsiku?

Alright, thank you. Lastly what chances is this picture giving that maybe life can change at your home, your own life as a guardian each and every day.

07/02: Chithunzi chimenechocho mwayi umene chikupeleka eti chikungopeleka kuti tikakhala pamavuto eti tiziyesetsa tisamangoyang'ana mbali imodzi tizikhala tikuyesetsabe kuti mwina mwake pang'ono ndi pang'ono mtendere ndikumaupezabe pang'ono kumbali yotani yothana kumavuto aja

The chances been given by that picture right, its just giving that when we are facing problems we should try our best, we shouldn’t just be looking on one side but try so that maybe we can find peace while getting rid of the problems.

I: Mukutanthauza kuti pang'ono mwina kupanga bizinesi mukunenayo kuti mupeze ndalama?

You mean that maybe doing some business that you are talking about to find money?

07/02: Kuti ndipeze chithandizo pakhomo paja.

For me to find assistance for the household.

I: Chapakhomopo.

For the household.

07/02: Eya nanga si bambo nde mwini mutu wazonse nde ngati iwo atere bambo tiyang'ana kuti

Yes since the man is the head of everything so if the man is like this then who are we going to look at.

I: Oho chabwino

Ok alright

07/02: Mmm

Mmm

I: Tathokoza tikuona chithunzi chachitatu eti palinso china chomwe mukufuna muyankhule?

Thank you, we are looking at the third picture right, is there anything that you want to say?

07/02: Ayi

No

I: Chabwino nde chithunzi chomaliza chimenechi tachifotokozeni chithunzi chimenechi ndikupemphani kuti mukwezeko mau chifukwa tikujambula apa nde ofunika kuti zikamveke bwino bwino

Alright then this is the last picture can you explain about this picture, I will ask you to raise your voice because we are recording so so it should be clear when listening to.

07/02: Mmm chimenecho eti najambula ndimmene mukuzioneramu mwendo wachibadwa ndi uwo uwowo ndiye ulipamavutowo pamavuto osanenekawo eya nde ndinawajambulano kuti azioneka pachithunzipo kuti nawonso adzitha kuona kuti mwendo wangawu unakali chitupireni choncho chifukwa nthawi imeneyo unali otupa kuposera pati pamenepo

Mmm, I took that one with the way you can see the leg that one was the one he was born with and that is the one which has problems, that huge problems so I wanted hi to be in the picture so that he can also see that, “my leg is still swollen like that,” because it was more swollen than that.

I: Oho pamenepo wasintha?

OK, so it has changed there?

07/02: Pamenepo wasintha chifukwa nthawi imeneyo ngakhale kuchucha madzi umachucha madzi lero zonsezo zinatani zidatha

It has changed there because that time it could even produce water, but both the water and all that stopped.

I: Mmm

Mmm

07/02: Eee

Yes

I: Chabwino ndiye pachithunzipa chikuchitika ndichani?

Alright what is happening on this picture?

07/02: Pamenepo chithunzicho chikuchitika ndichakuti iwowo angokhala pamenepopo eti akuwa ndi nthawi imene umakhala kuti wapeleka ululu chifukwa choti ukayamba kupeleka ululu kuugwira umazatabwanyika ndithu kumati mpakana kulowa cha size chonchi mkumkhala nde mkumati kodi kapena ndi madzi kapena ndi chani ukutabwanyika ndithu nde pamenepopo ukakhala kuti mphamvu ya ululu ija yatha umangokhalano owuma gwaa

What is happening on that picture is that he is just seated there, right. Its the time the swelling is causing alot of pain. When you touch it, you feel like its inflamed and when the pain ceases,it because hard.

I-chabwino ndiyeno mwafotokoza kuti munajambula kuti mukumbukire kuti apapa waphwa pang'ono eti kusiyana ndi kale chabwino mwinaso tinene kunena kuti chithunzi chimenechocho chikuonetsa kuti mumakumana ndi moyo wotani inuyo tsiku ndi tsiku ngati osamalira odwala

Alright now you have explained that you took this picture so that you should remember that there was a time when the leg improved,alright. Maybe let's say that this picture is showing that the kind of life that you live everyday as a guardian.

07/02: Ohoo chithunzi

Okey this picture

I-komanso chikupeleka mwayi wotani kuti mwina moyo upite patsogolo

So, what chances does it give to improve your life

07/02: Chithunzi chimenechocho eti chikupeleka mwayi wa tsiku ndi tsiku kukhala osasangalala chifukwa ngati munthu ukukumana ndi mavuto ngati amenewa ndikhulupilira sungakhale omasuka mu mtima chifukwa umaliingalira kuti kodi mavuto amenewa ndipo anangoti ineyo ndizivutika m’moyomu kodi mavuto amenewa kuti adzathe ndizapanga bwanji mmmm ndingapange bwanji nde chithunzi chimenechi chikupeleka ndithu dandaulo lakuti ngati inu madokotala mukhale, mukafukufuku wathu ali mu zinthu zofuna kutithandizira tithandizeni

Does this picture gives a chance to be happy in every day because if a person is facing problems like this one, i believe you can not be fine in your heart because you think that were these problems for me, to be troubled in my life. What should i do so that these problems should end? This picture is raising a concern that doctors should, in our research as shown in the pictures, help us.

I:mmmm mwayankha mbali ziwiri zonse eti palinso chowonjezera?

Okey, you have answered both questions, is there anything to add?

07/02: Ayi palibe

No, nothing

I:Tathokoza ndiye mutiuze kuti tikuombereni mmanja motani?

Thank you for your time. How should we clap hands for you?

07/02:aaah mwachisawawa basi

aaaa in anyway

I:akuti mmanja mwachisawawa, tikudziwamo?

She says, we can clap the ordinary way, do we know?

Ps: Mmm

Mmm

I: Tiyeni tiombe mmanja mwachisawawa

let's clap our hands the ordinary way

All: (Kuomba mmanja)

(clapping hands)

I: Chabwino, tathokozatu eti.

Alright, we thank you, right.

07/02: Mmm

Mmm.

I: Atsala anthu atatu? Chabwino. Emhe amayi ali kuti? Amayi anajambula ichi? Eya kumeneko akuonani. oo apo eee chabwino Chithunzi chimenechi mwa, chikuoneka bwinobwino?

We are remaining with three people? Alright. Yes mum, where is she? The lady who took this picture? Yes right there he has seen you. Oh there yes alright is this picture, can you see it properly?

09/02: Eya chikuoneka.

Yes it is.

I: Chabwino. Nde tifotokozereni, fotokozani chithunzi chimenechi kapena munajambulachi?

Alright, so explain to us, explain this picture, or the one you took?

09/02: Chithunzi chimenecho ndi chamwana wanga nde ndinamujambula kuti ndidzichiona ineyo.

That is my child’s picture so I took it for me to see.

I: Emhe

Yes

09/02: Mmm

Mmm

I: Chabwino, cholinga chake mmafuna kuti mudzichiona?

The reason is for you to see?

09/02: Mmm

Mmm

I: Ndiyeno, pachithunzipa chikuchitika ndi chani? Nthawi imeneyiyi akutani pachithunzipo?

So what is happening on the picture? What is he doing on the picture this time?

09/02: Nthawi imeneyiyi anangoima.

He was just standing this time.

I: Mmh.

Mmh

09/02: Mmm

Mmm

I: Chabwino. Chifukwa chani mwati munajambula chithunzichi, simnamve bwinobwino?

Alright, so what did you say is the reason why you took this picture, I did not hear clearly?

09/02: Chifukwa chake ndinajambula chithunzicho kuti ndidzikumbukira ndi nthawi imene iyeyi anadwala.

The reason why I took this picture was for me to remember the time when he got sick.

I: Oho.

Ok

09/02: Anadwala kwambiri mosazindikirika sindinadzindikire kuti ndingamuone chomchijayi.

He got very ill unrecognizable, I didn’t know that I can see him like that.

I: Oho, kuti mukumbukire ndi mmene kale amaonekera?

Okay for you to remember how he was in the past?

09/02: Mmm

Mmm

I: Kumujambula kuti panopa wasintha?

Taking a picture since he has changed now?

09/02: Eee kusintha kwabasi.

Yes, he has changed a lot.

I: Chabwino. Ndiyeno chithunzi chimenechi chikuonetsa kuti inuyo ngati osamalira odwala amene ali ndi cancer moyo wanu watsiku ndi tsiku kuti mmakumana ndichani?

Alright. So what is this picture showing us about your day to day life you live as a guardian for a cancer patient?

09/02: Moyo wanga wa tsiku ndi tsiku, moyo wanga wa tsiku ndi tsiki ndimakhala nawo osangalala, kusangalala ndithu poona chithunzi chimene chijacho ndimakhala ndikusangalala kwambiri chifukwa sindimkadziwa kuti ndingamuone chomchija iyayi.

My day to day life, I live a happy day to day life, Very happy and I become very happy also to see that picture because I didn’t know that I can see him like that, no.

I: Mmh, mukaona chithunzicho mumasangalala kwambiri?

Mmh, so you become very happy to see that picture?

09/02: Eee kobasi

Yes very much.

I: Nde chithunzi chimenechocho chikupereka mwayi otani kuti mwina moyo usinthe, tiupititse patsogolo.

So what chance is that picture giving us so that your life can improve?

09/02: Chithunzi chimenechocho, chithunzi chimenechija ndi, chikupereka moyo onena kuti malinga ndi mmene iyeyo anadwalira panthenda yake ya cancer ndinaona kuti wasinthika ndi mmene iye alili.

That picture, the picture is, its giving the life that, regarding how he got sick from cancer disease I saw that he has changed comparing to the way he was.

I: Mmm

Mmm

09/02: Eee zikusiyananso kwabasi.

Yes, there is a huge difference.

I: Nde nanga kupereka mwayi oti mwina zinthu zisinthe zipite patsogolo, pali uthenga wina uliwonse umene chithunzi chimenechi ukupereka? Pali uthenga otani umene chithunzi chimenechichi chikupereka?

What about a chance fro improvement, is there any message given by this picture? What message is this picture giving?

09/02: Chimenechi chikuonetsa uthenga onena kuti mwana ameneyu anachira ndipo ali ndi mphamvu zake ndipo sindimayembekezera kuti anali chomcho panopa ntchito inailiyonse imene iye amayenera kugwira yau dalaivala eti, akugwiranso bwinobwino ndikuonanso kuti palibe vuto lililonse limene iye ali nalo.

Its showing that this child is fine now and strong and I wasn’t expecting him to be like that and now he is able to do any kind of work especially the driving which he does without any problem that he have.

I: Okay, chabwino. Palinso china chimene mufuna muonjezere pachithunzichi kapena tipite kuchithunzi china?

Okay alright. Is there anything else that you want to add on this picture or we should proceed to another picture?

09/02: Palibe.

There isn’t.

I: Tipite kwina?

We should proceed to another one?

09/02: Mmm

Mmm

I: Chuthunzi chachiwiri chimenechi, mungafotokoze kuti kodi pachithunzipa pali ndani ndipo chifukwa chani munajambula chithunzichi ndipo chimachitika ndi chani pachithunzi chimenechi?

This is the second picture, can you tell us who is on the picture and why did you take this picture and what was happening on this picture?

(interruptions)

(interruptions)

I: Fotokozani mayi. Chithunzichi. Mukutha kuona bwinobwino, mukuona bwinobwino, bwerani olo chapafupi. Tengani mpandowo.

Mum explain, this picture. Are you able to see clearly, Can you see clearly, You can even come closer. Use that chair.

09/02: Ok ndamuzindikira.

Okay, I have recognized her.

I: Mwamuzindikira?

You have recognized her?

09/02: Mmm

Mmm.

I: Eya ndiye fotokozani. Fotokozani kuti pachithunzipa pali ndani?

Okay so explain. Explain who is on the picture?

09/02: Pachithunzipo pali mtsikana wanga wa neighbor ndiye ndinamujambula malingana ndi vuto limene iye amakhala nalo. Anadwala kudwala kwake anadwala TB ali wang'ono ndi nkuzabwera nkuzadwala amamwa ma ARV ndiye pakali pano amangozikandakanda nthupi mwake, osapeza bwino bwino amatha one week asanapite kuschool ndiye ndi nthawi imene mdamujambula kuti ndizimuona pachithunzicho.

There is my girl from the neighbors so I took a picture of her because of the problem she have. She got sick from TB when she was young and then got sick and takes ARV’s so right now her body itches, and doesn’t get better that a week can pass without going to school so its the time when I took that picture so that I can see.

I: Ok

Okay.

09/02: Mmm

Mmm.

I: Ok, Chabwino. Nthawi imeneyo apapa pachithunzipa amapanga chani?

Okay, alright. What was she doing that time on the picture?

09/02: Pamene paja anangoima.

She was just standing there.

I: Anangoima?

Just stood up?

09/02: Eee

Yes.

I: Chabwino, ndiye mwina mungabwerezenso kuti chifukwa chani mwati munajambula chithunzichi?

Alright, so maybe can you come again on the reason why you took this picture?

09/02: Chithunzi chimenecho mnangomujambula chifukwa iyeyo malingana ndi mmene mavuto amene ali nawo.

I just took that picture because of the problems that she has.

I: Owo, pavuto limene ali nalo?

Okay, on the problem that she has?

09/02: Ali nalo, amene ndakamba aja.

The one she has, the ones I have already said.

I: Ok, chabwino. Chithunzi chimenchocho chikuonetsa kuti ndimoyo otani umene inuyo mumakumana nawo tsik undi tsiku? Ngati osamalira odwala?

Okay, alright. What is the picture showing on the kind of life you live each and every day as a guardian?

09/02: Chithunzi chimenecho…

That picture…

I: Mmm

Mmm

09/02: Chithunzi chimene chijacho ndinachijambula moyo wa tsiku ndi tsiku oti umene timakumana nawo eti. Mwina tsiku limenelo wachisowa chinthu mwina wachipeza ukatha kuti mm kukachita ganyu ndiye kuti moyo watsikunditsiku pamenepo ukutani, ukupezeka.

I took that picture on the life we live each and every day right. Maybe you don’t have something that day or maybe you have found it after mmm doing some piece work then the every day life is there.

I: Chabwino ndikutanthauza kuti tikaona chithunzicho chikufotokozera zotani kapena chitifotokozera zotani kuti inuyo mukukumana ndimoyo otani watsikundi tsiku?

Alright I mean that when we look at the picture what is it saying or what is it telling us about the life that you live each and every day?

09/02: Tikachiona chithunzi chimene chija chikufotokoza kunena kuti tikukhala osangalala ku moyo wa iyeyo ndi mmene alili kusiyana ndi kale.

When we look at that picture its saying that we are living a happy life for her life with the way she is comparing to the past.

I: Mmh chabwino. Nanga chithunzi chimenechocho chingapereke kapena chingapereke mwayi otani kuti moyo wa inuyo amene mumasamalira wodwala upite patsogolo?

Mmh alright. So what picture can it give or what chances can it give for your life to improve as a guardian?

09/02: Chithunzi chimenecho chikupereka mwayi onena kuti ti, moyo wake umene amadwala tinamusamalira bwinobwino kuti adzifika size imene ijayi yaubwino.

That picture is giving a chance for, we were able to take care for the sick life up to reaching that good **size.**

I: Mmh, koma kuti moyo wa inu upite patsogolo, chithunzi chimenechi chikupereka uthenga otani?

Mmh, but for your life to improve, what message is this picture giving?

09/02: Moyo wake tikukhala osangalala kwa iyeyo ndimmene iye alili pamoyo wake.

We are living a happy life for her with the way her life is.

I: Mmm, chabwino. Chabwino. Tipitirize eti, palinso china chimene mufuna muonjezere?

Mmm, alright. Alright lets continue right, is there anything you want to add?

09/02: Mm mm palibe.

No, there isnt.

I: Chabwino, tufuna tione chithunzi chomaliza eti?

Alright, we want to look ate the last picture right?

09/02: Chabwino.

Alright.

I: Chithunzi icho, fotokozani kuti pachithunzipo pali ndani ndipo mmene, munajambula chithunzipo chifukwa chani, eya?

That picture, explain who is on the picture and when you, why did you take that picture, sure?

09/02: Chithunzi chimene chija ndimachijambula monga aneighbor…

I was taking that picture as a neighbor.

I: Mm mmh.

Mm mmh

09/02: Eya ndiye...(quite)

Yes, so…(quite)

I: Mm mmh mmajambula aneighbor?

Mm mm you took a picture of a neighbor?

09/02: Eya

Yes.

I: Munajambula aneighbor mwati chifukwa chani munajambula chithunzichi?

You took a picture of a neighbor and what did you say is the reason for taking this picture?

09/02: Amenewo ndidawajambula chifukwa choti ndimmene awo, akudwala matenda awo.

I took a picture of her because of her, the disease she is sick from.

I: Mm mmh

Mm mmh.

09/02: Amadwalabe TB komanso anadwalapo ndi matenda amamwa mankhwalawa komanso miyendo imakhala totupa ndi manja omwe ndipo akamayenda amakhala akumanjenjemera amayenda ndi ndodo.

She is still sick from TB and she also got sick from the disease and takes medication and the legs gets swollen together with the arms and she shivers while walking and uses a walking stick.

I: Chabwino.

Alright.

09/02: Ndiyeno ndinawajambula kuti ndiziaona ndimmene iwo alili, akhala pamene paja.

So I took a picture of her so that I should be seeing her and her condition, she is seated right there.

I: Chabwino. Apapa pachithunzi amapanga chani pamene mmawajambula?

Alright. What was she doing here on the picture when you ware taking the picture?

09/02: Pamene paja anangokhala pampando.

She was just seated on the chair.

I: Anangokhala pampando?

Just seated on the chair?

09/02: Eee

Yes

I: Ndiye chithunzi chimenechichi chikutionetsera kuti inu mumakumana ndi moyo otani tsiku ndi tsiku ngati osamalira odwala?

So what is this picture showing us about the life that you live each and every day as a guardian?

09/02: Chithunzi chimene chija, chithunzi chimenecho ndimaona kuti moyo wanga tsiku ndi tsiku umandisangalatsa chifukwa ndikuona kuti pakadali pano akumakhala akumayenda, kuyenda kukatenga poto kumaika pamoto kuphika msim ndiye ndimaona kuti ndimasangalala.

That picture, I can see my every day life as interesting to me because she does walk now, goes to take a pot, put it on fire and cook msima so i see that I am happy.

I: Koma ndimatanthauza kuti ndimafuna kutanthauza kuti chithunzi ichocho eti, chikuonetsera kuti inuyo moyo umene mmakumana nawo tsiku ndi tsiku ndi moyo otani? Osati iwowo koma inuyo ngati a neighbor awo…

But I meant that, I wanted to mean that the picture right, what picture is it giving on the life that you live each and every day. Not her but you yourself as her neighbor…?

09/02: Koma ineyo?

But myself?

I: Mmm, amene komanso muli a guardian a patient.

Mmm, who is also a guardian.

09/02: Chabwino.

Alright.

I: Eya

Yes

09/02: Ineyo moyo wanga wa tsiku ndi tsiku kwa iwowo ndikuona kuti zimatha kundisangalatsa ndi mmene mbuyomu anachokera chifukwa amatupa mphunoyi yonse nde ndikuona mmene alili muja ineyo ndikuona kuti ineyo mmoyo mwanga monga mmene mnawaonera ndikuona ngati kunena kuti ineyo ndikusangalala nazo pamoyo wanga kwa iwowa.

My everyday life to her I can see that it makes me happy comparing to where she is coming from in the past because her whole nose would swell so I can see the way she is, myself I can see that my life regarding to the way I saw her my life is happy with that for her.

I: Ok

Okay.

09/02: Mmm

Mmm.

I: Nanga chikuperekano mwayi otani kuti moyo wanu upite patsogolo ngati osamalira odwala?

So what chances is it giving for your life to improve as a guardian?

09/02: Zikuonetsa kuti moyo wanga kwa iwowo ndimmene iwo alili ndikuona kuti inebe, ndibwerezabe ndikuona kuti ndiosangala pamoyo wawo kapena pamoyo wanga ndikuona tikumasangalala chifukwa zina ndi zina akumapezeka nazo akumazithandiza.

It shows that to her my life with her condition I can still see that I, I will repeat, I can see that her life is happy even in my life we are happy because she is being found with some other things and assisting herself.

I: Mm mmh.

Mm mmh

09/02: Mmm

Mmm

I: Chabwino, palinso china chimene mukufuna muonjezere pachithunzipa?

Alright, is there anything that you would want to add on the picture?

09/02: Aa aah, palibe.

Aa aa, there isn’t.

I: Palibe?

There isn’t?

09/02: Mmm

Mmm

I: Chabwino nde tikuombereni mmanja motani, tikuyamikeni motani?

Alright, so how can we clap for you, how should we thank you?

09/02: Mwachisawawa.

Ordinary way.

I: Ati tiombe mmanja mwachisawawa eti, tiyeni tiombe mmanja mwachisawawa.

She says we should clap in ordinary right, lets clap hands in ordinary way.

All: (clapping hands)

(Clapping)

I: Tathokoza, tasala ndi anthu awiri, sitichedwa kwambiri. Eya tili apa. Anajambula chimenechi ndi ndani?

Thank you we are remaining with two people we will finish soon. We are here. Who took this picture?

08/01: Ineyo.

Myself.

I: Eya nde nenani. Chithunzi chimenecho fotokozani kuti pachithunzipo pali ndani?

OKk so talk, tell us who is there on that photo?

08/01: Pachithunzi pamenepo pali akazi wanga ali pamene paja panthawi imene ataulandira uthenga nditapita, nditapita kuchipatala ndikuulandira uthenga wa vuto lamwendo kuti ndi cancer anali okhumudwa kwambiri ndipo anakhala khuma panthawi imeneyo.

On that photo is my wife mind you by that time she received the message, I went to the hospital where I received the message of my leg that is cancer, she was very worried that time

I: Chabwino.

Okay

08/01: ndithudi.

Sure

I: Ndiye chithunzicho munachijambula chifukwa chani mwati?

So why did you took that photo?

08/01: Chithunzicho ndinachijambula chifukwa choti adaonetsa okhumudwa kapena kukhumata kapena zinawapatsa maganizo kwambiri chifukwa cha vuto lime lidapezeka.

I took that photo because she wsa worried or shocked because of the problem that was found

I: Chabwino. Panthawi imene mumawajambuka amatani apopo?

Okay, what was she doing on that when you were snapping her?

08/01: Panthawi imene ndimawajambula nkuti atachoka, atatha kugwiragwira ntchito zawo madzulo aaa ndipamene timakambirana kuti vuto limene lapezekaro ndi ili ndeno atakhala pampando paja anazakhala choyamba chomchi atamva uthengawo ndipamene anadzapanga chomchi kusonyeza kutani kukhumudwa panthawi imene timakambirana zavuto limeneli.

By the time I was taking a photo she was away, after doing everything in the evenining and its when we start discussing about the problem that I have while sitted on the chair, this was the style that she sat at first after hearing the message its when she changed and she really showed that she was worried by the time we were discussing.

I: Chabwino. Ndiye chithunzi ichocho chikuonetsa kuti inuyo moyo wanu watsikundi tsaiku mumakumana ndi zotani?

Okay, so that photo it shows what challenges you meet on your day to day life ?

08/01: Aaa chithunzi chimenecho pokhuza moyo watsiku ndi tsiku timakumana ndi moyo ovuta chifukwa kwambiri monga bambo amene umatha kuyang'anira pakhomo kuti pakhale bwino chifukwa cha vuto limenelo nthawi zina umapezeka kuti ukujomba sukugwira ntchito ndiye umakhala ndi nkhawa

kuti kodi anthu amene ndili nawo pakhomowa kuyambira akazi wangawo ndi ena onse amene timakhala nawo ndiwathandiza bwanji. Komanso thandizo loti ndingapeze bwino ndingapeze bwanji komanso aa kuti mauthenga ochokera kwa anthu amene amadziwa zinthu zimenezi tingawupeze bwanji ndipo tingalangizidwe bwanji. ndizina mwa izo zimene ndimakhala nazo nkhawa.

In that photo it shows the challeges that I meet in day to day life being a bread winner sometimes I fail to support my family just because of the problem that I am facing, I’m even failing to help my wife and the entire family. And also how can I reach people who have the same problem so that I can give them advice that is some of the things that I’m most worried

I: Mm mmh. Tsiku ndi tsiku.

Mmm , everyday

08/01: Tsiku ndi tsiku.

Everyday

I: Nde chithunzicho chikupereka mwayi wotani oti mwina zinthu zikhoza kusintha pamoyo wanu?

So what chance are you getting from this photo that your can change?

08/01: Aaa chithunzi chimenecho chikupereka uthenga onena kuti kwa azimayi onse kapena kwawina aliyense amene amakhala akuyang'anira odwala kapena amakhala ndi mnzache zakeyo wapezeka ndi vuto uthengawo wampeza kuti munthuyo ali ndi vuto lamgonamgona asakhale okhumudwa akhale osangalala komanso upeleka mwayi oti kwa adindo amene amakhala ndi udindo otha kuyang'anira zamavuto ngati amenewo athe kupeza njira yamankhwala amene munthu oti amajeckson akumavutavuta akusowasowa majecksoniwo athe kupezeka komanso akafukufuku ngati mmene achitiramupathe kafukufuku kupitirira kuti mwina kapena Mulungu atha kutichitira chisomo kupezeka mankhwala enieni othetsera vuto limeneli kuti ena kutsogoloku adzathe kuthandizika asazakumane ndizimene tikukumana nazo ife pano.

The message that this photo is carrying is that to all women or everyone who is taking care the patients or thet live with their friends who have the same problem this message should reach them that that person is suffering a long term disease they should not be worried they should be stress free but it also it gives an opportunity for the guardian of this problemthey should found the means of getting injection if it happens that there is no this injection then the studies like this one the way they have done so that God should have mercy so that we you found the really medicine so that we should end up this problem in the future so that they should not meet with the problem we are meeting

I: Chabwino. Palinso choonjezera tisanapite kuchithunzi china?

Okay , do you have anything to add before we go to another photo?

08/01: Ayi palibe choonjezera.

Nothing to add

I: Chabwino. Chithunzi china ndi chimenecho fotokozaninsoni pazachithunzi chimenecho.

Okay, another photo is that one, explain about that photo

08/01: Pazachithunzi chimenecho awo ndi wakazi wanga amene nthawi zonse ndimati ndikaweluka kuntchito kwanga kupita kunyumba tikalandira chakudya amandilimbikitsa kumwa mankhwala popeza moyo wodwala sizimalephera nthawi zina zake sukufuna kumwa koma iwo aja amndikakamiza kuti ndimwe mankhwala cholinga choti ndithe kupeza bwino ndithe kukhala ndi moyo wamphamvu watsiku ndi tsiku.

On that photo is my wife who always when I nock off from work and going home after the dinner she incourage me to take the drug because getting sick sick is just part of lifesometimetimes it happens that I don’t want to take the drug but she force me to drink so that I should get well and to be strong

I: Chabwino ndiye chifukwa chani munatenga, munajambula chithunzi chimenechi?

Okay fine so why did you take that photo?

08/01: Mnatenga chithunzi chimenecho chifukwa cha chikondi chimene amachionetsa paineyo chifukwa sizinthu zimene ndimatha kuziyembekezera akanakhala ena bwenzi akukhala asakukhala ndi chidwi chondilimbkitsa kumwa mankhwala.

A took that photo just because of the love that she has on me because that wsa not what I was expecting if it happens to be someone like my wife I think she cold not show the same love

I: Mmh, chabwino. Nde apopo amapanga chani nthawi imene mumatenga chithunzichi?

Mmm, okay, what was she doing by the time you wre taking that photo?

08/01: Pamene paja ndipamene titatha kudya ndipamene anatenga mankhwalawo ndikutunga madzi ndikugwada ndikumandipatsa ine kuti ndimwe mankhwalawo.

Thai is when after eating is when she takes water and medicine and kneel down then giving it to me so that I should drink

I: Chabwino. Nde chithunzi chimenechi chikuonetsa kuti inuyo mumakumana ndi moyo wotani tsiku ndi tsiku?

Okay, so picture does this photo telle in your daily life?

08/01: Aaa chithunzi chimenechi ndimakumana ndi moyo umene ndimaona kuti iwonse umawakhuzachifukwa ndi munthu okuti amatha kukhala, munthawi yokuti angathe kupuma ndipamene amatha kukhala kunena kuti aa mamuna wanga uja akafika ndiye kuti ndimuyang'anire kuti wabwera bwanji ndifunse kuti ali bwanji komanso ngati pali kusintha ndiyesese kupeza njira yonena kuti akhale ndi moyo osangalala. Nde kukhala ndi moyo osangalala kwambiri monga banja kumakhala ndi malire chifukwa chavuto likabwera kuganizira pamene pajapa nde ndi chifukwa chake chidandisangalatsa.

It just shows that she wsa also concerned because the time she could maybe rest is when she think of me and ask me how I am feeling so that she should be happy, so the happiness that is supposed to as afamily is too limited because of this problem that is the reason I decided to take that photo

I: Chabwino. Ndiyeno timakamba zoti chingapereke uthenga wotani kuti pakhale mwayi oti moyo wanu upite patsogolo.

Okay, so we were discussing on how it can improve your life

08/01: Aa moyo wangawu kuti upite patsogolo nkhani yaikulu ndikupezeka kwa chithandizo monga mankhwalawo. Aaa mankhwala atamapezeka ndikukhulupira kuti ululu utamakhala kuti ukuchepa mu thupi pamwendo mmene uliri titha kumapezeka kuti tikugwira ntchito tikamagwira ntchito ija zizipangitsa kuti ngakhale apabanja amene tikuwasamalira adzikhala osangalala pazinthu zina zimene angathe kufuna chifukwa moyo wamunthu umakhala ufunika zinthu zambiri kuti akathandizike kudzera mwa bambo.

My life can improve the biggest issue is when I get good treatment, if the medicines are available I really believe that the pains that I’m feelling could change my leg which could help to do some other business this help a lot in the family we live happily as you know we men we are the head of the family

I: Chabwino, tathokoza palinso china kapena tipitirize chithunzi china?

Okay thanks, anything to say before we go to another photo?

08/01: Ayi palibe tiyeni chinanso.

No I don’t have

I: (interruptions) Chomaliza chimenecho. Fotokozaninsoni ndithu.

The last photo just explain for us

08/01: Awo ndi akaziwo ndi nchemwali wanga amunawo ndi mphwanga amene ali wanumber 2 kwa omaliza. Amenewowo nditawafotokozera za vuto limene ndinapezeka nalo oyamba anali chemwali angawo anandilimbikitsa kuti aa achimwene musadandaule ife tili pambuyo panu simathero amoyo zimachitika musakhale okhumudwa, mphwangayonso anafotokoza chimodzimodzi ife tili pambuyo panu kukuthandizani munjira ina iliyonse imene ingathe kupezeka imene ife tingathe kukwanitsa nchifukwa chake mnawajambula pamenepo.

That is my inlaw, a wife to my young brother, last but one in our family. Afre explained to her about my problem she encouraged me that I should nt get worried we are on dide this is not the end of your life it happens and my brother also said the same thing that is why I took that photo

I: Nchifukwa chake munawajambula?

That is the reason you took the photo?

08/01: Amha

Yeah

I: Pamenepopo amapanga chani?

What was she doing?

08/01: Pamenepopo nkuti tili mnyumba ndimawafotokozera za vuto limenelolo monga ana amodzi sindikuyenera kubisa vutolo chifukwa ndikabisa vutolo mwina atha kukhala ndi thandizo lawo linalake lapadera kapena ndakumana, ndadwalika iwo aja ngati akudziwa vutolo ndiye kuti adzatha kuthandizana ndi alamu awowa popanda vuto linalirilonse ndiye anakhala pamene paja ndikumawafotokozera ineyo mpamene amandiyankha zimenezo.

By that time were in the house and I ws explaining the problem that I havebeing one family I don’t have to hide anything because if I hide my problemthen I can not get their help, if I am serious with this disease, my inlaw can help me so she was there while I were explaining to her

I: Chabwino. Nde chithunzi chimenechocho chikufotokoza kuti mumakumana ndi moyo wotani tsiku ndi tsiku?

Okay, what challenges does that photo tells about your everyday life?

08/01: Aaa chithunzi chimenecho ndi moyo watsiku ndi tsiku umene timakumana nawo umakhala ovuta koma ukakhala ndi abale okukonda namakulimbikitsa zimakuthandiza kuti mavuto ukukumana nawo aja adzichepa mmaganizo mwako kuti udzikhala omasuka.

That photo is showing that we meet with different challenges but when you are with your relatives who are lovely they really give courage that this problem we are meeting they will decline so that we can be free

I: Mmh.

Mmm

08/01: Mmm

Mmm

I: Nanga kupititsa patsogolo moyo wanu chithunzi chimenecho chikupereka mpata wotani kapena mwayi otani?

What picture does this photo tells about your future?

08/01: Aaa chithunzi chimenecho chikupereka mwayi ndinenanso kwa monga inu amene mukundifunsa mafunso ndi amnzanu onse muli kutsogoloko kunena kuti pali ena zimatheka kupita kuchipatala kuja nkumatha kukusala kumene kuti akuthandize. Koma chifukwa cha mmene inuyo mumachitira pomatilandira kuchipatala nkutilandira mwadongosolo, mwaulemu, motithandiza aaa mwayi umene ndingapemphe ndi onena kuti atapitiriza, mutapitiriza pamenepopo kapena kutenga uthengawu kukafikitsa kwa anthu akuluakulu amene amatha kudziwa zakayendetsedwe kamakono ngati amenewa kuti mwina pathe kupezeka njira zina zimene nkupita kwa nthawi tingathe kukambirana kupatsana mzeru zimene zijazo zitipeza ife odwala kapenanso kwa maguardian ndikukhulipira kuti kutsogoloko vuto limeneli lidzatha kudzachoka.

That photo is giving me an opportunity especially to you who are asking me questions and your friends together who are in front that there are some people that they happens to go the hospital but being dicrimanated to be helped, but because of the way you did in terms of welcoming us here in the hospital we get a very marvelous treatment freely its my pre that you should continue, continue and even take this message to other people so that they should know how to thissharing knowledge that will give us the victims and the guidians hope that this problem will end

I: Chabwino. Palinso china choonjezera?

Okay, anything to add?

08/01: Aa choonjezera ndikungothokoza chifukwa chamwambo umene mwatipatsa kuti tidzafike pano tidzalankhule ndizambiritu titha kulankhula kutsogoloku sitingathe kulankhula leroyi koma kutsogoloku...

I would like to thank you because of this function that have given us courage but I will talk much in future here I can not say much

I: Kutsogoloku?

In the future?

08/01: Kudzikhala kukubwera nzeru zosiyanasiyana zimene tikathandizane.

I will be having some different ideas that will help us

I: Chabwino.

Okay

08/01: Zikomo.

Thanks

I: Ayi tayamikira ndiye tikuthokozeni bwanji?

We are very grateful, how are we going to thank?

08/01: Inetu mmanja mwa chifumutu zidzukulu za kwatainetu zino.

I want you to clap hands for a king, I am a grand child of chief kwataine

I: Owo kutereko mndzukulu wa a mfumu eti, mudakanena kalekaletu, tiyeni tiimbe.

Ok you are a grand child of chief kwatani, you could tell us before, lets clap hands

All: (clapping)

(clapping)

I: Tathokoza, tili pa omaliza eti? Chabwino. Chithunzi ndi chimenechi, anajambula ndani?

Thanks, the last one right? Okay, this is the photo, who took this photo/

09/01: Ine mnajambula ndine.

I am the one who took that photo

I: Aa chabwino ndimayang'anatu mbali iyi. Chabwino fotokozani achimwene pa, kuti chithunzichi pali chiyani?

Okay I was looking to this side, okay explain my brother, what is ti there in this photo

09/01: Okay ndikhonza kuimirira?

Okay may I stand?

I: Aaa olo mutakhala pansi vuto palibe.

While sitted there is no problem

09/01: Owo, owo chabwino. Chithunzi chimenechi mnachijambula kuona kuti anawo amadwaladwala ana amapasa awiriwo, uyo pali wakhala mtsikanapo, wanyamula mtsikanawo, wangwira mtsikanako wang’onoyo ndi wina ali mmanja mwa mai akewo ndiye mnachijambula chithunzicho.

Okay fine, I took that photo after seeing that this kids were getting sick frequently they are twins, there where is that girl, she have carried a girl, while the young one is in the hands of her mum so I took that photo

I: Okay, ndiye kuti chithunzicho tikuona pali ana amapasa awiri eti?

Okay, so we are seeing the twins

09/01: Mmm

Mmm

I: Nanga enawo, enawo ndi ndani?

What about others who are they?

09/01: Aaa enawo ndi mwana wa neighbor anamgwira mwana chithunziyo winayo ndi mwana wanga,

The other one is the child for my neghbour while the other oine is my kid

I: Okay, chabwino...(Overlaps)

Okay fine

09/01: Tsono enawo ali kumapetowo ndi abale awo aujeni a landlord amenewowo ali pamenepowo akumwetsa mwana belewo.

While others are the relatives of my landlord she is breast feeding her child

I: Owo, pameneponso palinso a landilordinso?

Okay there is also your landlord

09/01: Eee alandilord ndi amenewowo.

Yeah thus my landlord

I: Oho, chabwino. Ndiye mukunena kuti munajambula chithunzichi chifukwa choti ana awiriwa amadwaladwala?

Okay fine, so you just said you this photo just because these kids were gretting sick frequently?

09/01: Eya

Yeah

I: Chabwino. Koma pachithunzipo anthuwa akupanga chani?

Okay, but what are they doing?

09/01: pachithunzipotu, pachithunzipo akuyamwitsa mwana bere winayo akuphunzira kuima komanso pachithunzi chinacho chamwana ali mmanjapo akudwala.

On that photo, she is breast feeding her child while that one is learning how to stand and the other photo is a sick child in the hands

I: owo nde ali pakhomo anthuwo...

Okay so there at home

09/01: Eya ali pakhomo.

They are at home

I: Mmene timakhalira tili kunyumba kuyamwitsa mwana, chanichani?

As the way we are home breast feeding the child, like that?

09/01: Eya.

Yeah

I: Owo, chabwino. Ndiye tinene kuti chithunzi chimenechocho chikutionetsera zotani zokhuzana ndi moyo wanu umene mmakumana nawo tsiku ndi tsiku ngati amene mukudwala matenda a khansa mukukumana ndi moyo otani pachithunzicho?

Okay fine, so what should we say about that photo concerning your life that you meet in everyday life as the way you are suffering cancer what challenges are you meeting on that photo?

09/01: Chithunzi ichocho chikutionetsera mwayi okuti anawo kwenikweni ndikudandaula za anawo chifukwa anawo amakonda kudwaladwala akadwala modziyo winayo akasiya kudwala amazadwalanso winayo akasiyanso winayo kudwala amadzadwala, amazadwalanso winayo amangopanga choncho amasinthana sinthana.

That photo it just tells how worried I am concerning these kids becausethese kids they get ill frequently when one gets well she restart after few time

I: Chabwino ndiye chingapereke mwayi otani oti moyo wanu upite patsogolo kapena usinthe ukhale ndikusintha chithunzi chimenechichi?

Okay so what changes can it brings about your life?

09/01: Mwayi umene ungasinthe, ungasinthe...(Overlaps)

There can be some changes

I: Kapena chikupereka uthenga otani umene ungathandizire moyo wanu usinthe kapena ukhale wabwino kupititsa patsogolo.

Or what message does this photo carries, how can it help your life to be health

09/01: Mmm, tandifunseninsoni bwinobwino.

Mmm ask me clearly

I: Ndikunena kuti chithunzicho eti, ndikunena za chithunzicho eti mmene mwachioneramo chithunzi chimenechocho chikupereka uthenga otani oti mwina ngati pangakhale mwayi, mwayi opititsa moyo wanu patsogolo ungakhale otani?

I am saying about that photo right, as the way you at it what message does it shows if there is chances of getting well

09/01: Mwayi umene ungakhalepo pamenepajapo pachithunzi chimenecho opititsa patsogolo ndiokuti (xxx) pa, ndikupempha ineyo kuti papezeke mankhwala othandizira ana amene ajawo tikuwaona pachithunzipo uyu wang'onoyu wina wang'ono kwambiriyo.

Chances that can be there is that I am just praying that I should found that can help the health of these kids especially the youngest one

I: Mmmh

Mmm

09/01: Eya ngakhale kuti sizikuoneka bwino bwino.

Although I’m not able to see claearly

I: Chabwino.

Okay

09/01: Eee

Yaeh

I: Tipitirize palinso zina zoti mukufuna muonjezere pachithunzipo?

Should we continue or there is anything to dd?

09/01: Ayi ayi sindingaonjezere basi zomwezo.

Nothing to add

I: Tipite pachithunzi china?

Should go to another photo?

09/01: Mmm

Mmm

I: Chithunzi china ndi chimenecho. Tifotokozereninso za chithunzi chimenechi.

That is another photo, explain about that photo?

09/01: Chithunzi ichocho ndi map akunyumba kwanga amene ndimakhala ine koma muziona kuona kuti mmene kukuonekera muja ndi pamyala moti ndimavutika kuyenda nkafika monga ngati usiku kwina ndi ma punthwa kuyenda ndimakhala movutikira nyumba chifukwa choti ili pamtunda. Mpamiyala ndithu ndiye ndimavutika kuyenda ndiponso ndi map amene ajawa.

That is my house’s map where I live but you should see that the way it is its on stones and I find it hard to walk and reach there especially when I come back in the night

I: Oho, muna, chithunzi chimenechichi munachijambuiranji, kapena munajambuliranji chithunzi chimenechi?

Ok, so you, why did you take this picture, or why was this picture taken?

09/01: Chithunzi chimenecho mnachijambula poona kuti ine ndimavutika mayendedwe.

I took that picture since I saw that I have difficulties in walking.

I: Mmm, kuti muonetse kuti mmavutika mmayendedwe kuti mmakhala kutali.

Mmm, to show that you have difficulties in walking since you stay far away.

09/01: Eee

Yes

I: Chabwino koma ndiye chikuonetsa kuti mmakumana ndi mavuto otani kapena ndi moyo otani tsiku ndi tsiku?

Alright, so what does it show about the problems that you face or the life you live each and every day?

09/01: Moyo, moyo wa tsikundi tsiku umene ndimakumana nawo ndi ovuta kwambiri ndimmene timakhalira mnyumbamo.

The life, the life I live each and every day is very difficult because of the way we stay in the house.

I: Mungafotokoze mukamati ovuta kwambiri…

Can you explain, when you say very difficult…

09/01: Eee

Yes

I: Mukutanthauza kuti chani?

What do you mean?

09/01: Kufotokoza kuti mmene timakhalira mnyumbamo movutana ndi vuto chifukwa choti chakudya chimavuta kuti ndichipeze.

explianing that we live with problems in the house because it is hard to find food.

I: Mmh, china? Tizi, muziyankha malinganso ndi mmene chithunzicho chikuonekera vutonso lina?

Mmh, what else? We, you should be responding in regards to the picture, another problem?

09/01: Komanso nyumbayo imene ndimakhalayo ndi yosalongosoka.

But also the house which i live in its not a good house.

I: Mmh, china?

Mmh, what else?

09/01: Basi.

Thats all.

I: Basi eti?

Thats all right?

09/01: Mmm

Mmm

I: Nde chithunzi chimenechocho chingapereke mwayi otani kuti moyo wanu upite patsogolo olo anthu atachiona chithunzicho mukuona ngati chingabweretse uthenga otani kwa munthu amene akudwala khansa kuti moyo wake usinthidwe, kungochiona chithunzico?

So what chance can that picture give for your life to improve, if people look at the picture what message can it give to a cancer patient to improve hie/her life just looking at the picture?

09/01: Chithunzi chimenecho chitha kupereka mwayi wa odwala khansa ndi mmene chikuonekera mmene ndinajambulira ine.

That picture can bring a chance to the cancer patient with the way it can be seen and the way i took it.

I: Mmm, mwina mwayi wake ungakhale otani? Umene ungabwerepo pamoyo wamunthu amene akudwala cancer anthu atati achiona chithunzichi.

Maybe what can be the chance? Which can be brought about on the life of the cancer patient if people can see the picture?

09/01: mwayi wake ndi ovuta kwambiri chifukwa monga mmene talongosolera kunena kuti mayendedwe amavuta, ndimavutika kuyenda mayendedwe nkamabwera kunyumba kwangako.

The chance can be very hard because like i have said that i find it difficult to walk, walking becomes difficult when am coming home.

I: Chabwino, palindo china chimene mufuna muonjezere?

Alright. is there anything that you want to add?

09/01: Ayi pamenepo palibe china choonjezera.

No there is nothign to add there.

I: Chabwino tufuna tione chithunzi chomaliza eti?

Alright we want to look at the last picture right?

09/01: Mmm

Mmm

I: Chabwino. Chithunzi chomaliza ndichimenechi tifotokozereninso mmene tinapangira mwina muja...

Alright. This is the last picture tell us also just as we did in the others.

09/01: Sono uyo ndika, ndi mwana wanga. Nthawi imeneyi ndikudwala ndiye mwana uja anali asakumvetsetsa bwino bwino ndimati ndikamutuma kanthu kanditengere madzi, ukanditengere chopukutira ndikamtuma amabwerera osatenga zinthu zija ndiye amandiuza kuti mwandituma chani ndi chani ndiye ndimamuuza kuti ukatenge madzi akumwa ndi chopuputira mthupi ndiye mwana uja amazitenga zinthu zija kuti azi, kudzandipatsa ine nkugwiritsira ntchito ndiye chithunzicho chimandikumbuktsa kuti pamen pajapo kuti ndimujambule ndizimuona.

That is my child, that time when i was sick she wasnt hearing properly so when i ask her go and take some water fro me to drink and a towel she would come back without those things and ask again what did you ask me to take for you so i would tell her that go and take water for me to drink and a towel for my body so she could then go and take those things and give them to me to use so that picture reminds me to take a phot for her so that i should be seeing her.

I: Kuti ndiamene amatumikira?

That she is the one who does those things?

09/01: eee

Yes

I: Ndiye apopo amatani tsiku limenelo?

So what was she doing that day?

09/01: pamenepopo amasewera.

She was playing there.

I: Owo, amangosewera?

Ok, she was just playing?

09/01: Eee

Yes

I: Mmm

Mmm

09/01: Amasewera

Was playing

I: Chabwino, ndiye chithunzi chimenechocho chikutionetsa kuti inu mumakumana ndi moyo otani tsiku ndi tsiku.

So what does that picture show us about the life you live each and every day?

09/01: Chithunzi chimenencho chikutionetsa mwayi okuti mwanayo 1 amadwaladwala, 2 mwanayo mmene mukumuonera sali pa sukulu iyayi, amangokhala.

That picture is giving us a chance that that child gets sick more often, thats 1 and 2 as you can see the child is not at school, she just stays.

I: Amangokhala?

She just stays?

09/01: Amangokhala pakhomo mpata okuti angakhale ndi mwayi oti mwana angapite ku sukulu tilibe, chifukwa panopa mphamvu zabwera kumene.

She just stays at home, there isnt a chance for her to go to school because am just starting being strong now.

I: Mmh kusiyana ndi kale?

Mmh, comparing to the past?

09/01: Eee kusiyana ndi kale.

Yes compairing to the past.

I: Oho ndiyeno chithunzi chimenechi chingapereke mwayi otani kuti moyo wanu upite patsogolo?

Okay so what chances can this picture give to improve your life?

09/01: Chithunzi chimenechichi chingapereke moyo wanga okuti upite patsogolo mnakakonda ntapeza ntchito yoti ndizigwira ndizithandizira mwana ngati amene ujayo.

This picture can give me chance for improving my life, i would love if i can get a job to work so that i should be assisting that kind of a child.

I: Chabwino, palinso china.

Alright, si there anything else?

09/01: Ayi basi

No, thats all.

I; Chabwino mwina pazithunzi paja talankhula paja palinso ndemanga iliyonse imene mukufuna mulankhule?

Alright, maybe on the pictures that we have talked about is there anything you want to say?

09/01: Ndemanga ilipo kwaineyo kungoti tipemphe kwamadokotala, mwayi wa mankhwala ngati ungapezeke mukanene kumeneko kumene mumanenako kaya ndikumphikako mukanene kuti anthu anu kuno akufuna mankhwala adzilandira adzimwa kapena jackson adzipezeka. Ineyo ndiye ndemanga imene ndingapereke ndi imeneyo.

There is something to say, i should ask the doctors, if there can be a chance for the medicineto be available there, where they report to you should tell those that your people here wants to be getting and taking the medicine even the injection should be available. Thats my word that i can say.

I: Chabwino. tathokoza kwambiri eti ndiye tiku, tikuombereni manja motani.

Alright, thank you very much so how can we clap hands for you?

09/01: Ine amfumu.

The chiefs one.

I: Amfumu eti, tiyeni tikuombereni mmanja mwachifumu.

Lets do the chiefs clap.

All: (clapping hands)

FGD3

I: Nde mwina titayandikira kwamene akhonza kuima akhonza kuima kwamene sangathe kuima angofupikira akhale pamenepo tidziona zithunzi eti. Ama tafikanitu pafupi.

So lets be close here and those that can stand can stand and those that cannot stand they can sit close so that we can be able to see these photos, mum come close

01/02: Eee

Yes

I: Tafikani pafupi mungotenga mpandowo mudzakhalire kaya cha apa. Tonse tikutha kuona zithunzizi eti?

Come close and just take this chair and sit here, so are we all able to see these photos?

01/02: Mmm

Mmm

I: Mmm, tadziika mmagulu ndiye mmaika ndinu eti ndiye tikupemphani kuti mutifotokozere kuti pamene mwaika zithunzizo gulu limenelo likutanthauza chani, ndiuthenga wanji umene tingapeze kuchokera pagulu limenelo. Eti?

Mmm, we have categorized them so we will ask you to tell us what that picture category means and what message can we get from that category. Right?

P: Mmm

Mmm

I: Chabwino. Nde mwina tiyambe ndi gulu ilo mwaika apolo, wina angotifotokozera kuti gulu limenelo likutanthauza chani.

Alright. So maybe we should start with that category over there, someone should tell us what that category mean.

09/01: Ili

This one?

I: Eee

Yes

07/01: Gulu limeneli ndi la odwala amene sakumva bwino mthupi. Odwala onse ali pamenepapa sakumva bwino.

This is a category for the patients they are not feeling well in their bodies. All the patients are here, they are not feeling well.

I: Mmm, ndiye kuchokera pagulu limeneli ndiuthenga wanji umene mungatipatse okhuzana ndi zithunzi zimenezi ndi kukhala ndi matenda a cancer mungatiuze zotani kapena mungaliuze dziko zotani zokhuzana ndi zithunzi zimenezi?

Mmm, so from this category what message would you give us concerning these pictures and having cancer, what can you tell us or what can you tell the world about these pictures?

07/01: Apopo monga ifeyo odwala tikuliuza dziko lonse kunena kuti mwina mwake atipatse mankhwala oonjezera pamene tikulandirawa ngati pali ena kuti mwina anthu akubwerawa asazaonensovuto lina ngati limene taliona ifeyo.

On there we as patients are telling the whole world that maybe they should give us additional medicine on top of the ones we are getting so that maybe the other people to come should not experience the problem that we have experienced.

I: Mmm, Ena, tiyeni timasuke eti. Aliyense akhonza kulankhula. Enanu mungatiuze zotani zokhuzana ndi gulu limeneli?

Mmm, some others, lets be free right. Anyone can talk. What are the others telling us about this category?

08/01: Aaa gulu limenelo uthenga wake ukupereka onena kuti tiyenera kupereka uthenga kapena kudziwitsana kapena kuphunzitsana kuti nthenda imeneyi ikufalikira kwambiri pamene ikufalikira kwambiri ikusokoneza zinthu zosiyanasiyana zimene pamoyo wamunthu angathe kufikira kuti akathe kutukula mabanja awo.

Aaa the message from that category is that we are supposed to give the message, or tell each other or teach each other that this disease is spreading so much so while its spreading its interfering with different things that in life a human can manage in order to improve their families.

I: Mmm. Chabwino. Uthenga wina umene tingautenge kuchokera pamenepa?

Mmm okay, whats the other message that we can get from here?

04/02: Uthenga wina ndi okuti ifeyo maujeni maguardian amene tikusamala matenda tisamakhumudweyi koma tidzikhala tikutani tikudziwa, tidzikhala tikulimba mtima kuti tidziathandizira odwalawo tiziasamalira moyenerera kuti tisamakhumudwe chifukwa ifenso tikakhumudwa ndiye kuti matendawo sangathe kuchira msangayi koma tidziwalimbikitsa ndikuwathandiza chinachilichonse chawo pamene akudwala pamoyo wawo tsiku ndi tsiku.

Another message is that, we the guardians who are taking care of the sick we have not to be disappointed and we should always know, and stay strong hearted so that we should be assisting the patients we should be caring for them properly so that we should not be disappointed because if we are also disappointed then the sick one will not get healed quickly but we should be encouraging, and assist in anything while they are sick on their day to day life.

I: Mmm, ena. Ndi uthenga wanji umene mungalipatse dziko okhuzana ndi anthu amene, guli limene mwaika apa la anthu amene akudwala?

Mmm, anyone else, what message can you give to the world about the people who are, this category here of people who are sick?

06/01: Aaa anthu amenewo ndi gulu limene tikudwala ndiye tikuyenerabe kuti mmene tajambulamu chomchi nkutheka alipo ena akufuna kwabwino amene angatengeko mbali yothandizira mapatientiwa komanso mwina pali mnzeru zina kaya manhkwala ena komanso kuti dziko lidziwe ndithu mmene nthendayi yafalikira. Ndiye gulu lake ndilimenelolo.

Aaa these people, it’s a category of us who are sick so we are supposed that, now that we have taken pictures it might be possible that there are some well wishers who can play a role in assisting these patients and maybe there is also somewisdom or some medicine and also for the world to know the way this disease has spread. SO that’s the category.

I: Mmm, aliponso ena amene akufuna kuonjezera uthenga umene mungatipatse ochokera pazithunzi zimene mwajambula apazi, zili apa gulu limeneli la anthu amene akudwala?

Mmm, anyone else who wants to add on the message that you can give us from these photos that you took,which are on this category for sick people?

07/01: Kuonjezera pamenepo pali mau onena kuti matupi athu sakuyenda bwino ngakhale mmene tangojambuliramu ngakhale wina obwera kuti aone adziwa kuti ii anthuwadi akudwala ndiye kunena kuti amene otisamalirawo tiapatsebe chilimbikitsa chonena kuti adzitiona ngati anthu monga amene amatisamalira ifeyo.

Just to add there is a word that says our body are not okay even the way we took these photos if someone come he/she can know that these people are are sick so for those who are taking care of us we should give them the courage that they should take us like human beings like to our guardians.

I: Mmm, mukati adzitiona ngati anthu?

Mmm, what do you mean when you say they should take you as human beings?

07/01: Chifukwa chonena kuti ena amatisala amaona ngati siife anthu amnzawo iyayi (xxx) nde tingonena kutibe adzionetsa kuti ndife anthu Mulungu monga mmene anatipangira apitirize kutisamalira.

Because there are some who discriminate us as if we are not humans (xxx) so we should just say that they should show that we are Gods people just like the way Hw made us so they should continue taking care for us.

I: Chabwino. Pali china choonjezera?

Alright, is there anything to add?

05/01: Eee, gulu limenelobe likutanthauza kuti ife anthu amene tikudwala matenda a cancer tisakhalebe anthu otaya mtimayi komabe tikhale anthu achikhulupiriro kuti tsiku lina Mulungu adzatichitira chifundo tidzatani, adzatichiza komanso dziko lidziwe kunena kuti anthu tikuzinzika ndi matenda amenewa a cancer, ngati kungakhale akufuna kwabwino kaya mabungwe kaya achipatala ngati angapezeke mankhwala atithandize.

Yeah, that group means that, we the people who are sick from the cancer disease we should not lose our heart but we need to have faith that one day God will have mercy on us, He will heal us but also the government should know that we are suffering with the disease of cancer, if there are well wishers like the organizations or the medical personnels, if medication can be found, they should assist us.

I: Mmm, chabwino. Tikupita kugulu lina, chilipo choonjezera?

Mmm alright, we are going to another category, anything to add?

08/01: Mmm, choonjezera chilipo chonena kuti apo zikuonetsa zamatenda acancer wa kuli ena, awa ndi anthu amene atha kufika nkuonekera poyera koma kuli ena amene sakudziwa kuti ndi khansa akumadzibisa ndiye mwina patakhala kuti uthenga umenewo wakwera ndi kusaka anthu amenewo akanadziwitsidwa kuti iyi ndi khansa tisadzibise ndiponso ngakhale boma limene likathe kudziwa koma kuli matendadi akhansa awa ndi matenda akhansa okuti akuonekera koma pali ena oti ndi amkatikati amene sakutulukira kudziwika ndiyeno ndi mbali imodzi yokweza kuti ngakhale dziko lonse lapansi likathe kudziwa kuti ku Malawi kuli vuto la khansa limene likusowekera chani chithandizo.

Mmm, just to add on, here it shows the cancer disease, there are some,these are the people who have come openly but there are some poeple who are not aware that they suffering from the cancer, they do hide themselves so I think if there can be that message have reached high and search for those people and they could have been told that this is cancer and we should not hide ourselves and even the government should know that there is really cancer disease, these are cancer diseases that are able to be seen but there are some in the midst whch are not becoming out here they are not known so its another thing to raise that the whole world should know that there is a cancer problem in Malawi should is lacking assistance.

I: Mmm

Mmm

08/01: Ndithu

Sure

I: Chabwino. Titha kupita pagulu lina kapena aliponso ali ndi choonjezera?

Alright, so we go should to another category or is there anything to add?

All: (Silence)

(silence)

I: Ndiye pali zithunzi mmm izi zochepa zili apazi, tikutha kuziona eti izi gulu limeneli wina angotifotokozerako kuti ndi gulu lotani limeneli, ndi zithunzi zotani zimene zili pa gulu limeneli?

So we have these photos mmm these are few, we are able to see right, can someone tell us about this category, what kind of photos are in this category?

03/02: Ndi zomwe maguardian monga icho mmm khansa patient akulandira mankhwala kwa guardian.

Mmm, its what the guardians like that one, a cancer patient is receiving medicine from the guardian

I: Mmm. Ena kuti ndi gulu, ndigulu lotani limeneli?

Mmm, what are the others saying about this category?

08:01: Aaa choonjezera pamenepo ndi chisamaliro chakwa odwala chokhuza mankhwala kuti guardian kapena munthu wina amene angathe kutengapo nawo mbali athe kumuthandiza odwalayo mosatopa pomukumbukira nthawi imene amayenera kumwa mankhwla nthawi zonse. Ndikumankakamiza kuti amwe mankhwalawo ngakhale asakufuna koma amwe mankhwalawo mwachidule tingoti chisamaliro chokhuza kamwedwe kamankhwala.

Aaa just to add on that is the caring from the guardian concerning medicine, it’s the duty for the guardian or anyone who is taking care for the patient to take part on assisting the patient without getting tired and to keep time that is necessary for the patient to take drug all the time, and force the patients to take the drug although she/ he don’t want to take the drug in short we should say the procedures of taking the medicine.

I: Mmm, chabwino. Nanga ena choonjezera, ndi uthenga wanji umene tingapereke kudziko okhuzana ndi gulu limeneli? Anthu amene akulandira, mapatient, maguardian akupereka mankhwala kwa ma patient awo?

Mmm alright, anyone to add, what message is there concerning this category? People who are receiving, patients, guardians who are giving drugs to their patients

All: (silence)

(silence)

I: Ndi uthenga otani umene tingathe kupereka kudziko, tingalifotokozere zotani dziko zokhuzana ndi gulu limeneli?

What message can we deliver to the world, what can we say about this category to the world?

08/01: Tionjezereponso, uthenga ndi onena kuti kwa amene amathandiza ndi mankhwala kuti guardian aoneke kuti ntchito yake akugiwra bwino zitengera mankhwalawo ngati akumapezeka ndiye uthenga umene ndikupereka kudziko lapansi ngakhale adzitsogoleri athu kunena kuti ayetsetse kuti mankhwalawa adzitha kupezeka pafupipafupi kuti nayenso guardian chidzikhala chopepuka, opepukitsidwa kukhala ndi katunduyo pomuthandiza odwala.

To add on, the message is that those who are helping with the medicine so that the guardian should show that they are playing their task it all depend on the availability of the medicines, so the message that I can deliver to the world even our leaders is that they should try their best so that these medicine should be available frequently so that it should be easy on the guardians by having the things fr assisting the patient.

I: Mmm

Mmm

03/01: Chinanso choonjezera nchonena kuti guardian asatope kumusamalira munthu odwala. 2, guardian kaya ndi akudwalawo kaya mwina ndi abambo ndiye akusamalidwa ndi akazi awo zomva imva asazigwiritse ntchito chifukwa ndinazamva week yatha ija amayi ena ake amanena zoti aaa anthu mwina ngati ma neighbors amatha kumawauza zoti aa cancer imeneyo akupatsirani amuna anu athaweni ndiyeno penapake zomvaimva kwa maneighbors asazigwiritse ntchito. Ee kaya iweyo kaya ndi mkazi wako kaya ndi mwana wako akudwala musamalire mosatopa.

The other thing is that a guardian should not be tired with of caring for the patient, 2, the guardian whether the patients is father and being cared by his wife, they should not take any rumors, because last week I heard a certain lady complaining that she heard some rumors from their neighbors that its cancer and he will transmit t to you disease so just run away from your husband so sometimes such rumors from neighbors should not be taken into consideration. Yes whether being you or your wife even your child just care for them without getting tired.

I: Mmm

Mmm

03/01: Ya, kutseka khutu zomva imva za negative zomwe ungamve mwa anthu ena akuti sakufunira zabwino.

Yeah, it is better to close your ears to avoid such negative rumors that we can hear from some people that are bad wishers.

I: Mmm, chabwino chabwino. ena choonjezera?

Mmm okay fine, anyone to add?

07/01: Choonjezera pamenepa tikayang'ana pa ma picture amenewa tikuona kuti mwana wachichepere watenga mankhwala ndi cup, tidziwalimbikitsanso ana athu kuwapatsa mankhwala, kawapatse kaya ndi, odwalawo adzikhalanso ndi chilimbikitso kumpatsanso munthu mankhwala amene wadwala.

To add on that when we see these photos, we are able to see a little child taking a drug and water, we have to encourage our children to take the drugs, giving them, a patient should also get that courage get the drug that is related to his or her disease.

I: Mmm. Chabwino. Azimayi tikuti chani tangokhalatu chete (laughs) Tili ndi chilichonse choonjezera pagulu limeneli la maguardian akupereka mankhwala kwa ma patient awo kapena patient akumwa mankhwala?

Mmm, okay. What are we doing ladies we just quiet (laughs) do we have anything to add on that category of guardians who are giving drugs to their patients or patient taking drugs

05/01: Apapa gulu limenelili likutanthauza kuti guardian akuyenera kupanga chikondi kwa patient chifukwa zimakhonzadi munthu kudwala chigonere kulephera kunena kuti atenge mankhwala aja ali pampando poterepo kuti akoke guardian ali pati, ali pomwepo kukanika kuti ampastire koma nthawi akudziwa kunena kuti yatani, yakwana. Ndiye apopo zikulimbikitsa kunena kuti guardian ndi udindo wake kuti asatope, asakhumudwe olo atamva chilichonse chokhukudiwtsa pakati pamatenda ndipamene pamakhala zokhumudwitsa zambirmbiri koma iye asatengere zimene zijayi koma atengere ndi udindo umene watani wausenza panthawi imeneyoyo nde apopo gulu limenelo likutanthauza kunena kuti nthawi yomwera mankhwala ngati ikwane guardian ndi udindo wake kuti atani agwiritse ntchito kuti patient uja akhale kuti wamwa mankhwala nthawi yake.

This category is trying to mean that the guardian has a duty of showing love to the patient because it happens that a patient is in the bed and is failing to say take the drug on the chair so that she/he can pull while the guardian is there watching while the guardian already that its time for the patient to take drug, the guardian should not be worried even though he/she hear something bad concerning the disease that’s what happens when there is a disease but she/ he should just take the duty of what they have carried on that time so that category means that is the duty of the guardian to give the drug to the patient on time.

I: Aliponso ali ndi choonjezera, tisanapite ku gulu lina? Titha kupita kugulu linatu eti.

Is there anyone else to add, before we go to another category? Hope we can go to another category right

09/02: Koma inenso ndingoonjezera mau anena asisiwa.

I just want to add what that sister has said

I: Mmm

Mmm

09/02: Guardianidi samatopa pali anthu ena amene amakamba kuti matenda amenewawa munthu akudwala chomchiyi ameneyi amwalira nkhani imeneyiyi imayankhulidwadi mmakomomu. Komanso kuchokera mmagadiyani momwemu monga ngati ife tagwira ntchito imeneyi mankhwala timatha kumapereka bwinobwino kuchipatala mpakana munthu uja kupeza bwinobwino pamakhalapo popanda chovuta bola iweyo kudzipereka kumampatsa guardianiyomankhwala kapena ku chakudya.

A guardian should not get tired there are some people who said that if a person suffers from cancer will automatically die these rumors are coming from homes, but on the same guardian like us we have had this same kind of work and giving medication properly at the hospital up to the point when the patient is feeling better without any problem as long as you are out yourself giving the **guardian** the medication or food.

I: Mmm.

Mmm

09/02: Eya.

Yes

I: Titha kupita kugulu lina kapena alipo ali ndi choonjezera. Tipite pagulu lotsatiralo tikuona pali mafuwa, akuphika, gulu limenelo. Tatifotokozereni zomwe zikuchitika pagulu limenelo?

Can we go to another category or there is somebody who wants to add. We should go to the other category, we are seeing this category cooking in an open fire, tell us what is happening on this category?

08/01: Aaa mbali imeneyo ikukhuzana pang'ono ndi iyi ntchitozo ndi zosiyana pamenepo ndi chisamaliro chapambali yazakudya ngakhalenso malo amene munthu amayenera kukhala bwino akhale a clean. Pamenepo ndikutanthauza kunena kuti guardian ngakhalenso munthu amene ali naye pafupi odwala auja ayenera kuziwa kunena kuti ayenera kulandira chakudya koma chakudyacho kuti achilandire ayenera kuchilandira muzipangizo zosamalika bwino komanso munthu opereka chakudyayo asaonetse okhumudwa adzikhala osangalala kumulimbikitsa odwala.

That is similar to this one, but the roles are different that is about the food care and also the places where a person is supposed to be clean. What I am trying to say there is that the guardian or the people around the patient are supposed to know that they are supposed to get food but for them to get the food is also supposed to get it in clean utensils and the person giving the food should not be worried but happy to give courage to the patient.

I: Mmm. Ena? Tikuti chani zokhuzana ndi gulu limeneli?

Mmm. The others? What are we saying on this category?

03/02: Gulu limeneli ndi zoti amapangitsa mankhwala kuti agwire bwino ntchito umafunanso kuti udzidya. Nde apopo zikutilimbikitsa kuti cancer, cancer patient nthawi zina zake kumamupatsa chakudya choyenerera chifukwa ngati mwina akudandaulirani, "ndili ndi njala," Osamuphikira atha penapake kumakhala ofooka ngakhale mumpatse mankhwala penapake mankhwala aja sangagwire bwino ntchito chifukwa choti sakudya mthupi akukhala ndi njala ndiye pena pake chakudya ndi mankhwala zimayenderanso limodzi.

This is the category that makes the medicine to work but also need food. So there it is encouraging us that the cancer patient is supposed to get proper food because maybe he is saying that, “am hungry,” if we are not cooing for her he might be week that even if given the medicine they cannot work properly because is not eating and is hungry so sometimes medicine work together with food.

I: Mmm ndiye ndi uthenga wanji umene tingapereke kudziko zokhuzana ndi gulu limeneli ndi matenda a cancer?

Mmm, so what message can we give to the world about this category and cancer disease?

08/01: Uthenga umene ungapite kudziko tikatengera ndi dziko lathu, mmene liriri dziko lathu ndimavuto amene ali mu dziko, pamapezeka kuti munthu akudwala koma thandizo lokhuza chakudya limakhala lopelewera ndiye kumapezeka kunena kuti munthu angodya msima ndi masamba daily pamene mankhwalawo amatha kufotokoza kunena kuti ofunika udzidya zinthu zamagulu atatu nde pali ena amalephera kukwaniritsa zinthu ngati zimene zijazo nde ngati kungakhale kotheka dziko litha kuthandizirapo kumbali zina kumbali yazakudya zofunikirazo kwa anthu amenewawa chifukwa pali ena oti atha kusowa ngakhale ndi kaphala kamene kukhala kungodya kamodzi patsiku mavuto amene amabwera kwa munthu odwala chifukwa ngati munthu wadwala mmene alilimu mkono wamanja omakasaka zakudya kusonyeza kuti munthu wamayi achita bwanji kapena munthu othandizira uja achita bwanji kuti azapeze zakudya kumakhala kovutika ndiye nthawi zina zake zimafunika munthu akadya amwe mankhwalawo kumapeza akumwa mmawa koma manhkwala aja opanda kudyera mapeto ake udzapeza kuti munthu uja aaa matenda aja amutenga nsanga koma kutakhala zotheka timaonapo mabungwe amatha kuthandizira anthu odwala mbali zina matenda ena amatha kuwathandizira tizakudya and kaphala kamene kumawathandizira ndiye atatenganso gawo kumbali ngati imeneyo ndikhulupirira kuti dziko litha kuchita bwino kwa anthu amenewa a cancer kuti mwina mwake adzikhala mosalekeza.

The message to the world regarding the way our world is, its found that a person is sick but does not have the food assistance and is found eating msima and vegetables each and every day while they happen to say you should be eating according to all groups of food with the medicine but some they fail to do that so if possible the world can assist on the food necessities to those people because there are some who might not have even porridge just eating once a day these are the problems that comes to a patient because if a person is sick the way he is the right hand which is supposed to go find food it means what can that lady do or what can the guardian do to find food its hard sometimes it is necessary to eat the take medicine it is found that he is taking the medicine in the morning but without eating as a result it is found that the disease has taken him so quickly but if it can be possible we see some other organization who happen ti assists the patients on some other diseases with food and even assisting them with porridge so if they can also take part on that side I believe that it can do good to these cancer people so that they might live ever after.

I: Mmm chabwino. Kaya ena tingapereke uthenga otani?

Mmm alright, what about others what message can you give?

06/01: Kumbali ya zakudya amapezekadi ena amatha kuwasala mapatient ndaonapo ine munthu akampatsa patient chakudya koma wavala ma gloves pamenepo olo patient kuchilandira adya momasuka, amakhala ndi nkhawa kuti ineyo nde kuti ndafika pa worse ngati guardian wanga wavala ma gloves kusonyeza kuti ineyo basi chabwino palibe ndiye tikuyenera aguardian kutenga mbali yokwanira ndithu yosangalala yomasuka kuti patient adyenso chakudyacho momasuka kumupatsa patient sambani mmanja ii tipemphere tidye zimene zijanso amadya patient uja momasuka osati chakudya icho akuyang'ana kumbali pamenepajanso pamatha kumpatsa patient kutani kukhumudwa osadya momasuka chinanso chokuti pali anthu ena akufuna kwabwino eti amene amatha kuona kuti aaa mmene anthuwa akuonekera tiwathandizire motere anthu amenewa ndithu ali ndi ufulu kulowerapo kunkhani ngati imeneyi yachakudya monga dziko lathu likuvutira.

On the issue of food there are some who are found discriminating patients I have seen a person giving food to the patient while putting on gloves so would the patient eat the food freely after receiving it? He has anxieties that it means I have reached the worse point if my guardian is putting on gloves then it means there is nothing good about me so as guardians, we are supposed to take a big role of being happy and free so that the patient should also eat the food freely, giving the patient “wash your hands, oh lets pray and eat,” the patient eats freely because of that not like, “there is your food” while looking on the other side you make the patient worry there and does not eat freely. The other thing is that there are some well wishers who happen to see that aaa we should assist these people in this way according to the way they are they have the freedom to do that on this kind of issue about food like the way our world is having problems.

I: Mmm, kaya bambo kumeneko mwakhala chetetu?

Mmmm, what about my father you are just quiet there?

02/01: Aaa ndi mmene akunenera anthumu.

Aah that’s the way the people are saying.

I: Mmm

Mmmm

02/01: Eee tiyenera kuthandizidwa moyenerera ndithu munthu umachira chifukwa chakuti wina akukuthandiza moyenerera koma ngati sakukuthandiza moyenerera matenda aja amapitirira chifukwa cha maganizo.

Yes, we are supposed to be assisted in a proper way and a person happen to be healed because someone is helping you in a proper way but if they are not assisting you in a proper way the disease proceeds because of the thoughts.

I: Mmm

Mmm

02/01: Eee

Yes

I: Kuthandizidwa moyenerera mukutanthauza chani?

What do you mean by being assisted in a proper way?

02/01: Monga mmene akuneneramo kuti ngati akukusala zinazina, kuvala maglovesi, kutani, kaya kupereka zakudya zosayenera kwa iwe iwo mwina adya zabwino iwe akakupatsa zoti, tangowapatsanitu pang'ono awowo akangodya ndekuti munthu matenda aja amapitirira ndi maganizo sangachizike iyayi. Ndithu.

As they are saying that if they are discriminating you on some other things, putting on gloves, what, maybe giving you inappropriate food while they have ate good food but giving you things that, “just give him/her a little something to eat then the disease continues with the thoughts and cannot be healed. Sure.

I: Kaya alipo ena amene angathe kuonjezera pankhani yokhuzana ndi gulu limeneli la zakudya ndi matenda a cancer? Uthenga umene tingathe kupereka kwa dziko.

Is there any other who can add on the issue about this category of food and cancer disease? The message that we can give to the world?

04/02: Uthenga opereka kudziko ndioti amene tili ndi matenda akhansa timakumanadi ndi zambiri monga zosowa zakudya inde tili nazo zakudyazo koma ndi zoperewera chifukwa enafe timangokhala nde ukakhala chomchija chakudya chikutisowa chikumatisowa mmanyumbamu chakudya koma ngati kuli mtundu okuti uthandize kunjako kaya nkoseko ntundu wa malawi onse uyenera kuti utithandizire anthu amene akudwala cancer chifukwa akatipatsa ndiye kuti ife ife sititani sitiziona kuvutika kwambiri kapena ku pangayi kumati kumakhala nkumati ii ine chakudya lerotu ndilibe koma mwina akakugaira umadziwa kuti kamene andigairaka ndikuthandizika matenda anga akutani akusintha.

The message to the world is that we the people suffering from cancer meets a lot of things like lacking food, so yes we have the food but its not enough because some of us just stays so if you are just staying we are lacking food in the homes but if there is people out there who want to help either the Malawians they are supposed to help these people who are suffering from cancer for us because if they can give us we will not be facing problems or doing, reaching the point where you say iii I don’t have food today but maybe if they can share to you.

I: Mmm, chabwino. Aliponso ena amene ali ndi choonjezera?

Mmm, Alright. Is there anyone who wants to add?

09/02: Apo tingoonjezerapo pamawu anena asisi aja timatha ife mmakomomu kusiya matenda mnyumbamu kukasaka chakudya kuti patient uja atani adye ndiye kumene wapita kuja pobwera patient amachita ukali munali kuti ndingokhala ndi njala ine oasdya pamene paja ndiye umangokhala phe sungapikitsane ndi patient iyayi ndiye udzingopanga zimene wabwera nazo kumene kuja kuti patient uja atani adye ndiye mwina mwache timakumana nazo kuti mmakomomummaka kumbali ya mapatient.

We should just add on that the words spoken by that sister, we happen to leave the sick in the homes to go and search for food for the patient to eat so when you are coming back from where you went the patient gets angry, where were you leaving me hungry here so you just keep quiet because you cannot wrestle with the patient so you just work on what you have brought from thee for the patient to eat so maybe we do meet things in the homes especially on the issue of patients.

I: Mmmm

Mmmm

09/02: Eee Chufukwa patient nthawi ina iliyonse amanena kuti chinthu chikakhala pafupi uyenera kumpangira mwansanga koma pakakhala palibe ndiye kuti zinthu zija zimakhala zotalikira kumpatsa patient ndiye kumpeza patient amanena kuti kodi simukundiphikira chifukwa chani ndiye kuwapeza mapatient ena amafuna kukumenya mwina kufuna kukuluma akungokuyang'ana chomchi iwenso kuchita kunena kuti munthu akundiyang'ana chomchiyi akufuna chakudya kodi nanga ndipanga bwanji. Ndiye pankhani zimenenzozo muyenerapo kunena kuti dzikolo liziwe kuti ma patient ena tiyenera kuwathandiza kuti kodi tingapange bwanji. Eee

Because they say that each and every time we are supposd to do things for the patient quickly but if its not available then its far away from giving it to the patient so you will find a patient saying that why are you not cooking for me, and you will find some patients will even want to beat you up maybe to bite you and they are just looking at you like this and you say this person looking at me like this meaning s/he wants to food so what am I going to do.So on those issues you are supposed to say that the world should know that we are supposed to assist some patients what can we do. Yes.

I: Mmm, chiliponso china choonjezera pagulu limeneli?

Mmm, is there anything to add on this category?

05/02: China ndi chokuti ma pateient ayenera kudya zokudya zakasinthasintha ngati zikupezeka.

The other thing is that the patients are supposed to eat a balanced diet food if its available.

I: Mmm

Mmm

05/02: Eee

Yes

I: Chabwino. Tipite kugulu lina? Gulu lili pakati pakuoneka zithunzi zambiripo, pamenepo.

Alright. Should we go to another category? The category which is on the middle with a lot of pictures, there.

06/01: Ilitu malire apa.

This is the boundary for this.

I: Kodi pali malire eti?

So there is a boundary right?

Ps: Eee

Yes

06/01: Eee ndiye kuti awa ndi ena, line ina awanso ndi line ina, awanso line ina.

Yes then these are on their own, another line and these, another line, and these another line.

I: Oho, ndiye tiyambe gulu limeneli lili pafupili. Tatifotokozereni gulu limenelo ndi...(overlaps)

Okay so we should start with this category which is close. Tell us about that category is…(Overlaps)

09/02: Kudutsa pakatipa...(Overlaps)

Passing through the middle

06/01: Gulu limeneli ndi lanthu odandaula akuoneka kuti ndi anthu okhuzidwa. Ndayamba tiye nawoni.

This is a category for worried people and they show that they are concerned. I have started lets proceed.

All: (laughing)

(laughing)

I: Ati ayambatu, tiyeni naloni.

She is saying that she has started, so lets proceed with it.

08/01: Gulu limeneli ndi lanthu okhudzika ndipo ndi odandaula nde ambiri mwaiwo tikawaona ndioti uthenga okhuza matenda akhansa mwina angoulandira kumene koma amatha kumva munjira zosiyanasiyana kuti khansa siimachiritsika, khansa imantengera munthu kumanda ndiye pamenepo tikuonapo anthu anagapo akudandaula pamene paja pali ana akudandaula kuti bambo athu chiwachitikire nchani pali mwina adzichemwali akudandaula pamene pajapo kuti adzilongo athu kapena adzichemali athu chiwachitikire nchani pali adzinyamata pamenepo palinso amayi ndi atsikana amene akudandaula chifukwa cha uthenga umene aulandira mosiyanasiyana.

This is a category for the concerned people and are worried,if we look at most of them its that they have just received the message about cancer disease but they happen to hear in different ways that cancer is incurable, cancer takes a person to the grave so we can see a number of people worried there and there are children worried that our what will happen to our dad there are also sisters there that what will happen to our brothers or sisters, there is young people there, there is also mothers and girls who are worried because of the message that they have got in different ways.

I: Mmm

Mmmm

08/01: Ndipitirizanso za funso lanu kuti ndi uthenga wanji umene ukupita kudziko?

I will also continue on your question that what message is going to the world?

I: Eee

Yes

08/01: Uthenga umene ukuyenera kupita kudziko ndionena kuti pali njira zosiyanasiyana zimene kaya ndi boma kaya ndi mabungwe ngakhale anthu amagwiritsa ntchito pofuna kufalitsa ndi kuphunzitsa anthu zamatenda osiyanasiyana chifukwa nthenda imene zikuoneka kuti kwa ineyo ndimaona ngati ikuthamangira Panumber 2 ndi HIV mwachina imeneyi imapereka ngati manyazi kuchititsa manyazi kwa munthu ndiyeno ambiri akachilandira chinthu ngati chimenechi amatha kukhumudwa ndi odandaula koma atalimbikitsika ndi kuphunzitsidwa bwino atha kunena kuti chinthuchi atha kuchilandira ndipo osakhumudwa ndikutsatira malangizo amene angayenere kupatsidwa ndimaphunziro amene angauzidwe kuti azichita ndi odwala kapena enu ake odwalawo kuti athe kulimbikitsika asakhumudwe asakhale okhumata ndipo asdziyang'anire pansi kwawo nkungolimbika kugwira ntchito kunsamalira odwala kapena kudzisamalira odwalayo payekha.

The message that is supposed to go to the world is that there are several ways in which either the government or organizations even the people does use inorder to spread and teach people on different diseases because the most disease which to me I see that its on number 2 is HIV on some other thing this disease gives like shame to a person so most of the people when they have received this they become very disappointed and worried but if they can be encouraged and taught very well they can say that, they can receive this thing and they cannot be worried and they can follow the advises that they are supposed to receive and the education that they can be told to do with the patient or the patient him/herself so that they can have courage and not worried not in anxieties and they should not look down on themselves but to work hard and take care for the patient or the patient taking care for him/herself.

I: Mmm

Mmmm

08/01: Zikomo kwambiri.

Thank you very much.

I: Kaya ena tikuti bwanji, titha kuonjezera motani pauthenga umene tingapereke kudziko pazinthumzi zodandaulazi.

What about others what can we add on the message that we can give to the world on the worrying pictures?

07/01: Zithunzi zodandaulazi amadandauladi zoona komabe nanga poti munthuyo ali naye ndiye akalangizidwabe nthawi yaitali poona kuti aa munthu uja tili naye amadzasintha makhalidwekumadzakhala nayenso bwinobwino ngati mmene amachitiranso gulu linalo kumakhala nalo. Mmm

These worrying pictures they were really worried but since they have the person so when they are counseled for a long time and seeing that aa we still have the person then they happen to change their behavior and stay with him/her properly like the way they were doing also on the other category. Mmm.

I: Mmm (laughs). Nanga ndi uthenga wanji umene tingathe kupereka kwa anthu nkhani yokhuzana ndi munthu amene akudandaula.

Mmm (laughs). What message can we give to people about a person who is worried? a sick person who is suffering from this disease?

09/01: Pamenepa zithunzi (xxx) monga ngati zithunzi izi.

Here on the pictures (xxx) like these pictures.

I: Owo (laughs)

Okay (laughs)

09/01: (laughs)

laughs

I: Chabwino, kaya ena tikuti bwanji?

Alright, what about the others what are we saying?

03/02: Uthenga umene tingapereke ndi oti sikuti munthu odwala cancer ndiye kuti wafa kaleyi.

The message that can delivered is that it doesn’t mean that a person who is sick from cancer is aready dead.

I: Mmm

Mmm

03/02: Mmm, atha kukhalabe zaka zingapo akadali ndi moyo kupezeka kuti munthu wina wake okuti ali ndi mphamvu ndi amene mwina angayambe kufa kusiyana mwina ndi munthu uja ali ndi khansa uja.

Mmm, h/she can stay for some years while alive and it can be found that some one who is strong maybe is the one to die first unlike that person who has cancer.

I: Mmm

Mmmm

03/01: Eee bola munthuyo angokhala ndi chikhulupiriro chifukwa anthu ena amathadi kumalankhuladi kuti aa mmene cancer yabweramu ndekuti basi 3 years mwina siitha, kumati ee siitha anthu zolankhula lankhula zimachuluka.

Yes its better off if the personhas faith because some people do talk like, aaa now that there is cancer then it means that maybe 3 years will not pass saying yes it will not pass, people talk a lot.

I; Mmm

Mmm

03/01: Eee sizimalephera iyayi chifukwa ineyo ndazi, ndazionapo ndikumva anthu akulankhula.

Yes it happens because I have seen and have heard people talking.

I; Mmm

Mmm

03/01: Eee koma mayi ndi awo tikukhala nawo 3 years koma anthu komaziti ndiye kuti basi chaka sichitha, anthu kumalankula ndithu.

Yes but there ismy mother wo we are staying with for 3 years now but people were saying that a year wont pass, people do talk.

I: Ndiye mutiuze zotani anthu akudzikofe pamene anthu akulankhula zoterozo, mutiuza zotani?

So what are you going to tell us the people from the world while people are talking ike that?

03/01: Aaa ndi zoti pena pake zolankhula lankhulazi tisamazigwiritse ntchito chifukwa zimenezizi kuti uzigwiritse ntchito zotsatira zake ndiye kuti iweyo utha kumangokhala khuma nthawi inailiyonse kuti aaa akuti akuti aja anena kuti makolo angawa momwe akudwala cancer chaka sichitha kapena 2 years apa sindiye kuti basi munthu ndiye kuti wafa kale. Ndiye pena pake zomvaimvazi tisamazigwiritse ntchito kwenikweni chifukwa chakuti zitha kumatikhumudwitsa.

Aaah sometimes we should not be using the things that people talk because if you use that the results are that you will be very worried all the time that aaa they are saying that my parents who are suffering from cancer wont lve for 2 years here then the person is dead already. So sometimes we should not use these talkings we hear because it will only disappoints us.

I: Mmm. Kaya ena ali ndi choonjezera pagulu limeneli.

Mmm, does the othershave anything to add on this category?

All: (Silence)

(Silence)

I: Palibe, tipitirire?

Nothing, should we continue?

06/01: Mmm

Mmmm

I: Nangano gulu liri apali tingali, lonseli eti

What about this group,what can we, the whole of this right?

07/01: Mmm

Mmm

I: Chabwino tatifotokozereni zithunzi zimene zili mugulu limeneli, ndi gulu lotani?

Okay tell us about these photos that is in this category,what category is this?

08/01: Ukutu sikukuyankhidwa funso loyamba uku.

This side is not answering the first question.

All: (laughing)

(laughing)

I: Anthu auku akhala chete eti.

People of this side are just quiet right.

05/02: Uku zafika uku mpaka uku.

From here up to here

03/02: Kuti, ndi uku.

Where, here

07/01: Eee layambira apapa.

Yes its starting from here.

06/01: Ili ndi gulu lolimbikitsa. Anthu amene amatha kukulimbikitsa pazina ndi zina monga tiyerekeze muli panyumba wina wake mwina, mwina ukufuna chinachake kaya chi, tiyerekeze chakudya chimene amatha kukupangira anthu ngati amenewa komanso mwina kumakumasula, kumakumasukira malankhulidwe, sukuyenera kudandaula ichichi sichithero koma ndi chiyambi choti iwe ugwanso step ina kumakulimbikitsanso kuti mwina pita kuchipatala magulu ake amakhala ngati amenewo kukuthandizira moyo wako wa tsiku ndi tsikumonga ngati zokudya zochitika zatsikundi tsiku magulu ake ndi amenewa.

This is a category of the courage givers. The people who happen to encourage us on some other things for example maybe at home,maybe someone wants lets say like the food that some people like these do make for you and also maybe freeing you, being free with you by talking to you that you are not supposed to be worried this is not the end but the beginning for you to ge to the next stepand encourage you tht maybe you should go to the hospital those are the category of people assisting in your every day life like the every day food and activities, these are the categories.

I: Mmm. Nde anthu otani amene ali pamenepo, a...(overlaps)

Mmm. So who are kind of people are there,..(Overlaps)

06/01: Anthu o, olimbikitsa

These people the courage givers.

I: Anthu ake ndi ndani?

Who are these people?

06/01: Apapa pali ma guardian, ena ongotumikira mmanyumbamu, ana amene amakuthangatira pachinachina kaya kucho, kupanga zapakhomo magulu ake ali pamenepa.

Here there are guardians, some other who serve us in the homes, the children who assists you on something either, doing household things, they are here.

05/01: Apapanso ndi guludi lachilimbikitso ndikuona kuti ena atenga ma baibilo mmanja kutanthauza kunena kuti anthu ngati amene aja amabwera pakhomo kuzamakulimbikitsa mwina ku zamau aMulungu kuti iweyo ukhale munthu wachikhulupiriro osati kuti ngati wapezeka ndi matenda a cancer ndiye kuti mmoyo wako wathera pomwepo ayi ndiyeno anthu aja akabwera amakhonza kukulimbikitsa mau aja amu baibulo komanso apapa apa ndikuonanso ambuye Yesu ali pomwepo kusonyeza kuti gulu lachilimbikitso lokhalokha kuti tikaganizira za ambuye Yesu zimene anakumana nazo ifeyo zathuzi ndi zochepa ndiyeno poti ifeyo ndife anthu si, timafulumiza kwambiri kufooka tikakumana ndi chani, tikakumana ndi mavuto koma apapa tiyenera kukhala anthu achani anthu achikhulupiriro tikaganizira ambuye Yesu komanso tiganizire baibulo, mabaibula atenga, pali akulu ampingo ndaona atenga baibulo mmanja kusonyeza kuti amafalitsa uthenga ngati wachilimbikitso otilimbikitsa chani ife amene tili ndi mateda a cancer kuti mmene zateremu osati kuti umoyo wathu wathera pomwepayi tiyenera kuti tikapeza mphamvu tidzuka tipita kutchalitchi tikamuthokoze ambuye ayi tsiku limenelo tilibe mphamvu tsiku limenelo titani tikhala komanso pamapezeka kuti mwina mwake anthu ena akutchalitchi atiyendera kudzatipatsa mau mwinamwake kaya ndi akulu ampingo kaya ndi ansembe kaya ndani amakhonza kuyendera mmakomo kuti tikuyendera odwala amafuna atani, kutilimbikitsa cholinga choti moyo wathu usakhale otani otaika osati kuti pamene tapezeka ndi matenda a khansawa moyo wathu wathere pomwepa ayi.

Here this is also a category of courage I can see some carrying bibles meaning that these kind of people comes home to encourage you on the words of God so that you can be someone with faith its not that your life has come to and end just because you have been diagnosed with cancer so those people comes and happen to give you courage with the words from the bible but I can also see Jesus right there it just show a courage category that whenever we thinks about what Jesus experienced whatwe are experiencing is less than that so we get weak when we meet what when we meet peoblems just because we are human beings but here we are supposed to have faith when we think about Jesus the Lord and we should also think of the bible, they are carrying bibles, there is also a church elder ave seen them carrying a bible meaning that they were preaching courage message encouraging us the people who have cancer so that although things are like this it doesn’t mean that our lies have ended here but whenever we are strong that day we should wake up and go to church to thanks the Lord if we don’t have strength that day then maybe the church people happen to come and give us the word be it the elder or the priest or whoever they happen to visit the houses hat we are visiting the patients they want t what to encourage us so that we should not loose our lives not that our lives have ended just when we have been diagnosed with cancer.

I: Mmm

Mmm

05/01: Mmm

Mmm

05/02: Chinanso ndi chokuti pamene tili takhala pamodzi ndi patient wathu tisaonetse nkhope yokhumudwa tiyenera kumaseka ndi patient uja nayenso kuti ululu uja ngati akumva kupweteka umakhala ngati ukuchepa chepa pamene tikusangalala naye limodzi.

The other thing is that we should not show a worried face when we are with our patient we shoukd be loughing with the patient so that the pain if is feeling pain it gets better little by little when we are happy with him/her.

I: Mmm

Mmm

05/02: Eeetu

Sure

I: Ena ndiuthenga wanji umene tikupereka mudziko lonse?

What about others what message can we deliver to the whole world?

08/01: Uthenga ukupereka pamenepa monga akhala akutisonyezera gulu limeneli ndi lachilimbikitso, chilimbikitso chimenechi chikutanthauza kunena kuti wina aliyense amene amuona ngati guardian kapena patient akhala naye pafupi kumulimbikitsa munjira zosiyanasiyana kucheza naye kusangalala naye kugwirana naye chanza, kumuphikira kapena kumutengera malo osiyanasiyana amene angathe munthu uja kukakhala kukasangalala kutengedwa ngati nayenso ndi munthu ofunikira padziko lapansi. Nde uthenga waku dziko lapansi kwa onse ndionena kuti padzikhala chilimbikitso mauthenga achilimbikitso owalimbikitsa anthu odwalawo potengera kuchita nawo mmagulu osiyanasiyana kaya ndi moimba kaya zisangalalo zosiyanasiyana zimene zingathe kukhalapo munthu uja akamatengedwa chomchija amatha kumalimbikitsika akamaona abale ndi alongo anansi akukhala nayo pamodzi.

The ,message that can be delivered here as they have been showing us on this courage category, this courage means that everyone who sees a patient or guardian if can be close to him/her and give him courage in different ways, chat, be happy, hand shake, cook and take her to places as an important person in this world. So the message to the world there should be some courage, courage messages to the patients and being with them in different groups be it in singing different available entertainments if the person is taken like that and is seeing his/her relatives and friends and being with them together happen to have courage.

I: Mmm, chabwino. Ena choonjezera pauthenga umene tingapereke ochokera ku zithunzi zimenezi?

Mmm, okay. Others what can add on the message we that we can deliver to the nation from these pictures?

MK: Mai (name) simukulankhula, mwatani. Aa aa zakhala bwanji?

Mrs (name) you are not talking,whats wrong? What has happened to you?

09/02: Tingonena kuti kumbali yachilimbikitsocho ingakhale maneighbors amakhalanso olimbikitsa komanso adokotalanso amalimbikitsa olo upite kuchipatala kapena akuyendera pakhomo. Amakhala achilimbikitso chachikulu ngati mmene chikukhalira sikuti ndi maneighbors okhanso iyayi koma madokotalanso amatani amalowerera pamenepopo chilimbikitso chimakhalapo.

Just to add on what my friends have said on the part of encouragement even neighbors they do give courage and also the doctor does give courage also even when you go to the hospital or they have visited you ate home. They do give you huge courage like the way it is its not the neighbors only but even the doctors does what take part on that and there is courage.

I: Uthenga wina omwe tingapereke?

What other message can we deliver?

07/01:Uthenga wina ndi onena kuti anthu amenewa amatithandiza kwambiri chifukwa ngakhale tadwala ngakhale tisadwale chilichonse chimene ukufuna ukawauza anthu amenewawa amatichitira nde tifunabe kuti apitirize kutithandiza mmene iwowa amapangiramu.

Another message that can be delivered to the nation is that these people assist us so much because even when we are sick or not and everything that we want if you tell these people they do it for us so we want them to assisting us like the way they do.

I: Chabwino chabwino.

Alright, alright.

06/01: Komanso tingomaliza motere anthu awawa kuti chisangalalo chawo chipite patsogolo zikuyenerabe anthu akufuna kwabwino awathandize kuti kodi anthu amenewa asangalala ali, tiasa, tipange motani kuti anthuwa akhalebe osangalala chomcho, akhalebe othandizira omasuka chomcho mwina ndithu ntundu wa a Malawi udziwe zimenezo.

But also I should finish by that there is a need for the well wishers to help these patients for them to leave a very happily life people of Malawi should know this

I: Mmm. Aliponso ena amene ali ndi choonjezera?

Mmm. Is there anyone else who want to add on what we have said?

09/02: Tingoonjezerapo chisangalalo chimene akunena asisiwo eti tingoonjezerapo kuti chisangalalocho chikhalepo moti madokotala akumva, dziko likummva ndiyeno atapange zonena kuti kodi chisangalalochi chikhala bwanji popeza anthuwo tiwathandiza bwanji kuti nawonso tsiku lina adzasangalare.

On addition to what that sister has said is that I hope the doctors and the entire nation is hearing this message they have to think of what can they do to bring back happiness to people who are suffering from cancer so that it should be like a rememberance to these people one day

I: Mmm, apa chisangalalo tikunenachi tikutanthauza anthu amene amathandizira odwalawa olo kapena odwalawa?

Mmm, so the happiness which we are talking about here is for the guardians or the patients?

06/01: Iyayi

No

I: Othandizirawo

The guardians

06/01: Othandizira eee.

The guardians yes

I: Mmm, nde chisangalalo chake mwa zina chingathe kukhala chotani, choti dziko likhoza kuwathandizira anthu amenewa?

So what happiness can be there, the one the world can assist them with, just to mention a few?

06/01: Zikhonza kuchita bwino ngati dziko likuthandiza mapatient awo a anthuwa eti kupezeka zoti kuwathandizira monga mmene, mmene iwo angakwaniritsire kuwathandizira mapatient anthu amenewowo akawathandiza mapatient ndiye kuti anthu awawa chisangalaro chawo chidzakhala choposera pamenepa.

It can be good if the world is assisting the patients finding out that its assisting them in any way it can, if the patients are assisted then these people would be very happy than here.

I: Mmm

Mmm.

06/01: Mmm

Mmm.

I: Chabwino. Titha kupita kugulu lina kapena alipo ali ndi choonjezera? Anachalirira kuti iwo afotokoza gulu ili ndiye tatifotokozerani.

Alright. Can we go to another category or there is something to add? You said that you will explain this category so tell us.

09/01: (laughs)

(laughs)

I: (laughs)

(laughs)

09/01: Makamaka gulu ilolo ndifunseni.

Especially that category, ask me.

I: Eee, liti limenelo?

Yes, which one is that?

09/01: Ilo la amayi ali pamwambalo.

Thats one for the lady on top.

I: Oh ilo lili pamwambalo?

Okay, that one on top?

09/01: Eee

Yes

I: Tatifitokozereni gulu limenelo likuti chani?

Can you tell us about this category what is it saying?

09/01: Gulu limenelo likutifotokozera amene munthu ali wodwala cancer kuti wadzuka bwino tsiku limenelolo adzitha kumagwirako ntchito ngati bambo awo ali pachithunziwo.

That category is telling us that when a cancer patient has woken up well that day he should be working just like that man on the picture.

08/01: ichi chili pakonachi?

This one on the corner.

09/01: Eee chimenecho, chimenecho osamangokhala iyayi. Pamene adzuka bwino kumazilimbikitsa kuti tidzigwira ntchito.

Yes, that one, that one not just staying. Should be making ourselves strong so that we should be working.

I: Mmm, akutani amayi, abambo amenewo?

Mmm, what is that lady, that gentleman doing?

09/01: Abambo amenewo khala ngati aku...(overlaps)

This gentleman I think is…(Overlaps)

05/01: Khala ngati limodzi ndi izozi izi.

It seems its the same with these.

I: Mmm

Mmm

09/01: Akumanga, akumanga foundation ya nyumba.

He is building, building a house’s foundation.

I: Owo

Okay

09/01: Eee

Yes

I: Chabwino.

Alright.

08/01: Ooh

Okay.

09/01: Pamenenso tadzuka bwino tidzitha kuyenda ngati agogo awa, kumayenda bwinobwino kuti miyendoyi idzikhala yolimba bwino bwino.

We should be able to walk like that granny when we have woken up better, walking so that the legs should be strong.

I: Mm mm. Nde zina mwa zimene anthu akuchita mu gulu limenelo ndi chani? Mwati ena akumanga foundation ena akuyenda ena?

Mm mm, so what are some of the things which people in that category are doing? You said that some are making a foundation, some are walking, what about some?

09/01: Monga ngati pamenepa momwe zikuonekera apa apopo ndangoima pagalimoto(p 71, 1.11.16) ndinangokhala.

Like here the way it shows am just standing on a car, was not doing anything. (P71, 1.11.16)

I: Awo kodi akuphika?

That one, is she cooking?

05/02: Akudula kabichi (P 66, 1.11.16).

She is cutting cabbage (P66, 1.11.16)

I: Owo akudula kabichi. Nanga agogo awo? pamenepo.

Okay she is cutting cabbage. What about that granny? There?

05/01: Angokhala

She is just seated.

05/02: Angokhala.

She is just seated.

02/01: Akusoka zigambatu.(p 22, 1.11.16)

She is sowing piece of clothes.(P 22, 1.11.16)

06/01: Oo akusoka eti, eee akusoka. Gulu lake ndi lokhalokhalo.

Oh she is sowing, yes she is sowing. That is the same category.

05/02: Gulu lake ndi lokhalokhali.

This is the same category.

I: Tatifotokozereni zagulu limeneli kuti likutanthauza chani?

09/01:Pamene odwala cancer kapena matenda a TB kapena, kapena HIV tizitha kunchapira munthu odwala adzinchapira munthu adzisitasita zovala kuti adzivala zoyera.

When there is cancer, TB or HIV patient, we should be able to wash for that paerson, iron her/ his clothes to be putting on clean clothes.

I: Mmm. Ena tikuti bwanji?

Mmm. What are the others saying?

05.01: Gulu limenelolo lili pachisamaliro pomwe paja chifukwa chokuti pakuoneka zovala pamenepo, pakuoneka pogona chifukwa choti odwala ngati akungogona malo osayenera kudzapezeka kuti matenda aja atha mwinanso sikhala khansa yokha enanso abwera pati pomwepo chifukwa choti malo ogona aja ndi osasamalilika komanso zovala zimene akuvala mwina week yonse wakhala akuvala zomwezo zosatani zosachapidwa. Ndiye apopo ndikungosonyeza kunena kuti mmene anthu tikudwala khansa kapena mmene tili a guardian tiyenera kunena kuti tidzipanga check kwa odwala uja asamabwerenze bwerenze zovala komanso malo ake amene akugona adzikhala otani aukhondo kunena kuti olo ena kaya madzinebor kaya ndani akubwera kudzamuona munthu uja pasamapereke mwinamwake kafungo kenakake chifukwa chokuti ukamangogona pomwepomwepo pamabwera ka sound kenakake kuti aaa chisamaliro chikutani chikuchepa koma ngati ukuchotsa zonsezo ndi kuchapachapa kapena ndikukuntha mikekayo kusesa ndikukoilopa ndikuyalanso ndekuti munthu uja olo obwera kudzakuona uja amangoona chinachilichonse chili chimodzimodzi, akudwala komatu olo akudwala koma mmm ndithu zaukhondo zokhazokhatu pakhomo pawo kutanthauza kunena kuti zonsezo zikuchitika chifukwa cha chani ma guardian komanso anthu apabanja amene akutani akusamalira odwalawo. Mmm

This is a category on the same care because we can see clothes there, a sleeping place because if a person is sleeping on untidy place it will found that it is not only cancer but will be additional ones just because of unclean place of sleep but also the clothes that she is wearing maybe putting on the for the whole week without washing them. So what am trying to say there is that when we are sick from cancer or we are guardians we are supposed to be checking the patient to not wear the same clothes over and over again and also where he is sleeping should be a clean place so that even if the neighbors or whoever has come to see him he should not be smelling. If you are just sleeping there time and time again there comes a certain sound showing that the care is not enough but if you remove all of it and wash them and cleaning the mat, sweeping and mopping and put them back then the visitors see everything as the same, “she is sick but but mm everything is clean at her home,” meaning that all is happening because of the guardians and the family members who are taking care for the patient.

I: Ena, uthenga wanji umene tingapereke kwa anthu okhuzana ndi gulu limeneli?

The others, what message can we give to people concerning this category?

04/02: Tikakhala ndi anthu odwala tinganene kuti tidziwasamalira tidziachapira dzovala zija tizia, chifukwa tikamaasintha sintha odwala wathu uja amatha kuoneka bwino, tikapitanso olo nzipatala amatha kutilangiza monga ngati madokotala athu amene akutiyendera mmaujeni mmakomo muyenera kumawasinthira kumaasambitsa bwinobwino chinachilichonse pathupi la munthu amafunika kuti munthu adzioneka bwino olo akudwala amafunika kuti adzioneka bwino, asambe.

When we have patients we should take care of them, we shld wash their clothes for them, because a patient happen to look good when we are changing her even when we go to the hospital the doctors happen to advise us like that like also our doctors who does come and follow us in our homes, you are supposed to change them, bath them properly including everything in a humans body because a person has to look good even though is sick, should be bathing.

I: Mmm, palinso zina zoonjezera.

Mmm, is there anything to add?

09/02: Eee zilipo. Tingo, tingoonjezerapo kuti mwa ife tokha ma guardian sitingakwanitse bwinobwino tithanso kapena kukwanitsa chifukwa munthuyo tikukhala naye komanso madokotalanso amakhala patsogolo ndi amenenso amatani, amatithandiza kumbali yamatenda ija chifukwa mwa ife tokha sitingakwanitse matenda amenewawa iyayi monga ife maguardian koma timakwanitsa maguardian timamuonerera munthu uja zikavuta timathamanga komanso adokotala ali patsogolo kuyang'aniranso ziti zomwezo ndiye tingopemphabe kuti madokotalawo adzipitirizabe kutitsatira ifenso tidzikhala pambuyo pawo. Komanso chisamalirocho akakhala munthu odwala sikuti amangogona chimodzimodzi umatha, akakhala kuti nthawi yaitali umatha kumutenbenuza kaya ali chigonere uchotse nsalu ukaichape uyale ina umgonekerepo ndiye amaoneka kuti mwina mwake thupi lake limakhala labwinobwino oti kubwera olo mulendo suchita manyazi ungoti aaa lowani chfukwa choti zinthu wazitani wazisamalira.

Yes there is. We should just add that on our own the guardians cannot manage or maybe we can because we are staying with the person but also the doctors are the ones ahead, they assist us on the disease because we as guardians can not manage this disease on our own but we manage to be looking at the person but when we see that its getting worse we rush to the hospital and the doctors are ahead to look after the same so I should ask that the doctors should continue to follow us and we should follow them behind. And if its a patient its not that does sleep the same all the time you happen to help him sleep from the other side while is aside then take out the clothe and wash it and make the place with a new one for him to sleep on so he seems to have a health body that even if a visitor comes you just say aa enter please because you have taken care of yourself.

I: Chabwino. Palinso choonjezera? Pali gulu linanso ili mnaiwala. Ili liri apali.

Alright. Is there anything to add? There is another category here I forgot it. Here it is.

02/01: Limenelo la mageni omuthandizira munthu. Chithandizo mwina chitha kumapezeka pali ma shop pamenepo, maokala kuti ngati munthu angakhale kuti wapezerako pang'ono ayenera kupangabe ka geni kenakake kokuti kazimusamalira pang'ono.

Thats for **gains** to support a human being. Then assistance might be present there are shops there, little hawkers so that when one is a little better should do the **gain** to take care of himself.

I: Mmm. Ndiye ndi uthenga wanji umene tingapereke kudziko okhuzana ndi gulu limeneli lopanga mageni.

Mmm. So what message can we give to the world about this **gain** category?

02/01; Uthenga wake ndi oti mpamba umachepa kumaloko.

The message is that there is little capital.

I: Mmm

Mmm

02/01: Eee patapezeka otithandizira mwina kapena mwina kapena aa ambirinso tikumapanga timageni toyenerera ndikumazithandiza tokha eeetu.

Yes if assistance can be found then maybe most of us would have been doing little businesses to support ourselves.

I: Mmm, ena. Ndiuthenga wanji umene tingapereke ku mtundu wa amalawi kaya kudziko lonse zokhuzana ndi gulu limeneli?

Mmm, any other. What message can we give to the people of Malawi or either the whole world concerning this category?

All: Silence)

(silence)

I: Mmh, basi ndi zomwezo mmene aneneramu, palibe choonjezera?

Mmh, is that all, just the way he has said, there is nothing to add?

All: (silence)

(silence)

I: Nanga pa, alipo amene ali ndi choonjezera pamagulu onsewa, mau ena aliwonse amene tingathe kufotokoza kaya uthenga uliwonse omwe tingathe kupereka kudziko lonse?

What about on, is there anyone with something to add on all these categories, anything to say or any message that we can give to the whole world?

07/01: Apo mauthenga ndi ambiri omwe tingafotokoze, chifukwa choti enafe sitinayambe talowa nawo mugulu ngati limeneli otiitana kubwera kuno taona kudwala kwathu ndi mmene takambirana zithunzizi tikuona kuti pali uthenga ofunikira kwambiri onena kuti dzikoli lidziwe kuti ife anthu odwala ife tikuvutika kapena kuti mwina kutsogolo kuno kusazakhale mavuto ngati amenewawa.

Eya

There are a lot of messages that we can give on that because some of us have never been in these groups where you are called to come, we have looked at our sickness and the way we have discussed these pictures we can see that there is a very important message for the world to know that we the patients are in trouble maybe there should not be these kind of problems in the future.

I: Mmm, ena.

Mmm, others.

08/01: Mmm uthenga opita kudziko ndimanena paja kunena kuti mmene zayalidwira apa ndi nkhani titha kuchoka poyamba mpaka kukafika kumapeto. Ndiye ndiuthenga opita kudziko mmadera osiyanasiyana munthu akapezeka kuti ndi odwala zikutengera guardian amene ali naye pafupi athe kupereka chisamaliro chithandizo kwa odwala akachoka pamene paja athe kumutengera munthu uja kupita kuchipatala athe kukalandira mankhwala komanso patakhala chiphunzitso ndi, chotanthauza kunenan kuti chifukwa ambiri timataya nthawi tikaona vuto timakonda kuthamangira mzikuda mpamene timakachedwera kutiko kumene kuja pokaapeza achipatala nthawi yatani yatha ndimamvera paraido kodi ndi dzana amanena kuti munthu oti wapezeka ndi matenda a cancer akangokhala kuti wayamba kupita kudzikuda amakachekeredwa ndi zina zoterezo izi ndi izi kumenekujako ndi akafika kuchipatala kuja amapatsidwa thandizo loyambirira limene lima, amapereka mankhwala kuyamba kumachotsa zitizo zimene zija zimene zimakapangitsa kuti kuchedwa koti alandire thandizo loyenerera ndiye uthenga ukupitanso kwa ma guardian kapena achibale amene amakhala pafupi ndi munthu odwala kuti wapezeka ndi vuto limenli jalo kunena kuti akaona vuto lija choyambirira athamangire kuchipatala akapita kuchipatala kuja akauzidwa zachani zotsatira zavuto lija zotsatira zake amuuza zoyenera kuchita koma kuti zikathe kufika pamenepo vuto ndi lakuti kwa ineyo mmene ndimaonera ndimaona kuti uthenga okhunza cancer ndiochepa umene ukutha kupita mmadera ngakhale mma, mtown muno utha kumapezeka pafupi pafupi koma kumudzi ukumakhala ovuta kufikako ena mawailesi alibe ndiye ngati dziko kapena boma kapena mabungwe atha kutengapo mbali ndikutha kugwiritsa ntchito njira zimene amazidziwa kufalitsa mauthenga a cancer kuti anthu adzitha kuzindikira mwansanga vuto lisanafike poipa ndimatenga chitsanzo ndikukumbukira m'bale wanga wina wake ndinapita kumudzi ndinakampeza miyendo yonseyi itatupa mpaka ndinafika size yomugwira mwendo uja sindimadziwa kuti ndi chani ndinamugwira mwendi uja ndikupeza uli gwa zotsatira zake mnamuuza kuti iziz ziyenera kukhala kuti ndi cancer musathamange kwasing'anga thamangani kuchipatala anthu aja sanamve then panangotha miyezi itatu munthu uja anatani kodi anamwalira koma kuthamanga kumene amathamangira amathangira kutiko mwa asing'anga ndiyeno patakhala kuti pali uthenga umene ukufalikira kumapita mmidzi paliponse njira zosiyanasiyana kaya ndi nyimbo madrama ngakhale mmawailesi ngakhale muzochitika zosiyanasiyana mmasukulu ngakhale mmatchalitchi ndikukhulupira kunena kuti odwala kapena oyang'anira odwala aja atha kuzindikira nsanga chinthu chimene chijacho then ndikuthamangira nsanga kuchipatala. Uthenga wina umene ungapite kudziko kapena kuboma lonse muli ma health centre ambiri amene ali mmafupi, mmafupi ndi kumadera kumene amachokera. Matenda ena ukadwala ukapita ku health center kuja ngati uwapeza kuchipatala chachikulu amanena kuti pitani ku health center yakwanu kumenekujako ukafikako akutumiza kuchipatala chachikulu, kutakhala kotheka atatumiza nsanga kuti anthu amene apezeka ndi vuto limeneli adzitha kulandira mankhwala ku madera kumene kwa afupikira zitha kuthandiza kunena kuti munthu uja asamathe kuyenda mtunda wautali komanso adzikatha kuonana ndi madokotala pafupipafupi chifukwa chavuto limene watani kodi wakumana nalo tingoyerekeza ngati ineyo kunyamuka kuchilimba kupita ku chilomoni, kuchilomoni ku Queens, kubwerera ndi ntunda wauitali nde umakhala otani ovuta oti kukhala munthu offoka kapena okuyang'anira kukhala kuti ndi munthu ofooka mmaganizo ngati mmene amakhalira ena angoti inu khalani musatitani, musativute nazo koma olimbikitsa ndimatenga chitsanzo ngati mmene amandilimbikitsa mayiwa, nyamukani tipite kuchipatala anakhala akulimbana nane mwina ndingoti 3 weeks, inu kodi simupita kuchipatala, mwendowo simukuwuona, simupita kuchipatala kodi simukuuwona ndimangoti aa ndipitabe ndili busy nthawi imene ndinazapita ndipamene ankakandiuza kuti ii achimwenetu vuto ndi liti ndilimeneli chifukwa chani, kutalikira. Utakhala kuti zili pafupipafupi zitha kuthandizira. Pempho langa limene lingapite kudziko ndikunena kuti atayesesa mwina mma health center mu kungotsekula ngati pali kochepa kokha kuti mwina munthu uja kungolandira mankhwala kaya kumene kuja akamva zotsatira koma akathe kulandira mankhwala kutiko kumene kujako komanso maguardian kapena achibale akhale ochilandira chinthu chija ndi mtima onse asamuone mb'ale wawo kapena guardian kapena amamuna wao kapena mkazi wawo kuti izi nde basitu zatha kale izi chifukwa ena amakhala ndi mtima oti zatha kale ndikagwira apapa ndiye ndikutengera koma kumva kwanga ndimanva kuti cancer siyopatsirana iyayi utha kumuthandiza mmene ungamuthandizire sungatengere sindikudziwa kuti ngati ndimanva udyo kaya ndi momwemo ndiye zinthu ngati zimene zijazo zitha munthu kulimbikitsika ndikumusamala munthu uja chifukwa chake akasamalidwa monsemu amakapezeka munthu uja akukagwira ntchito ndi manja ake chifukwa chokuti walandira chithandizo, kusangalatsidwa, kulandira mankhwala kupatsidwa chakudya ndi kulimbikitsidwa ndi munthu amene ali naye pafupi. Tingoyerekeza akazi angawa apange zinthu zondikhumudwitsa ngakhale ndikumwa mankhwala agwira ntchito ngati zotsatira zake umpeza wina iii ine ndikuvutika atenga mankhwala akumwa akutani akuzipha, waonong moyo wake pansi zikusokoneza mwana sukulu ndi ena ang'onoang'ono amene akadathandizika kutsogoloku komanso mwina iyeyo akadalandira mankhwala ndi chisamalira kuchokera kwa munthu amene akukhala naye pafupi kapena ku boma kapena kuthandizidwa munjira zisiyanasiyana atha kukhala chitsanzi cha anthu ena kapena kuphunzitsa anthu ena amene ali nawo pafupi kudera kumene ali..

Mmm, the message to the world as I was saying that the way the pictures are displayed we can start from the beginning to the end. So the message to the world in different areas, when a person is sick it is the duty of the guardian around to provide proper care to the patient then he/she should take the person to the hospital because most of us we waste our time when we have a problem by going to the traditional healer that is where he become late and get to the hospital too late. I was listening to the radio the day before yesterday they were saying that if a person who has been diagnosed with cancer rush to the traditional healer first she/he gets cuts and the like there so when she/he gets to the hospital you are given the first treatment, you are given the medicine to remove those as a result you are delaying the proper treatment so the message is also going to the guardians or other relatives who are close to the patient that he/she has been found with that problem, so when they see the problem they should rush first to the hospital and when they get to the hospital they will be told the results for their problem and what they are supposed to do but the way I look at it, to reach that point the problem is that the cancer messages are just a few those that goes to the areas or, you can found the messages in town very frequently

but it is hard to reach in the villages since some do not have radios so if the government or the NGO’s can take part and use whatever means they know to distribute the cancer messages for people to have knowledge on the problem before it gets worse, I take an example of my certain relative, I went to the Village and I found both of his legs are swollen, I had to touch his legs I did not know what it was and the leg was very dry then as a result I told him that this should be cancer you should go to the hospital no to the traditional healer but the people did not listen to me they rushed to the traditional healer as a result he passed away after 3 months since they were rushing to the traditional healer. So if there can be messages being distributed in all the villages and everywhere in either way, drama’s, songs or even radios, or even different activities in schools or in churches I believe that the patient or the guardian can have knowledge of that thing very quickly then rush to the hospital.

The other message that should go to the world or the government there are a lot of health centers in the areas near where they come from. In some cases when you are sick and has gone to the health center, if you get to the District hospital they tell you to go to your nearby health center, when you get there they will refer you to the District hospital, if it can be possible, if they can send you quickly so that the people who are found with this problem should be getting the medicine in their near by areas it can be helpful so that the person should not be walking a long distance and he/she should also be seeing the doctors time and again because of the problem she has been found wit. We should just take an example of myself from Chilimba to Chilomoni, from Chilomoni to Queens and back is a very long distance and hard if it can be a weak person like some other people they will just say, ‘just stay here and not bother us,” but I take the example of the way my wife was encouraging me, let go to the hospital, she kept on pucshing me for maybe 3 weeks, ‘hey are you not going to the hospital, cant you see the leg?” And I was like, “aaa am busy I will go some other time.” When I went there it is when I was told that, “iii my brother this is the problem,”. Why, it is because its far.It can be very helpful if it can be close by. My plea to the world is that if they can try to open something in the health centers so that the person might maybe get the medicine or he can get the result but especially to get the medicine there. But also the guardian or the relatives should accept that thing whole heartedly they should not regard their relative or guardian or husband or wife as if that is all because some people they do have a mind that this is it if I can touch him/her here I will get it but I hear that cancer its not transmitted you can assist the cancer patient the way you can I don’t know if I got it right so things like those can give courage to the person and take care for the patient that is why when he/she has been taken care of in all these it is when the person goes to work with his/her own hands it is because has received care, being entertained, received medicine, been given food and being given courage by a near by person. Lets take for example if my wife has done something which is disappointing to me while am taking medicine will it work? As a result you will find one is saying, “iii am in trouble and will get medicine and end his/her life. Has destroyed own life and creating trouble for a school child and some other little ones who were supposed to be assisted in the future and also maybe had it been that he/she got care from the person around or the government or being assisted in any different ways would have been an example or would teach some people around him/her something.

I: Mmm

Mmm

08/01: Zikomo kwambiri. Komanso kuonjezera apopo kunoko ndaphunzirako kanthu kena pakati apa mwina tikadabwera ndi chithunzi cha anthu awiri kapena atatu amene ndaona akuvutika mofanana ndi ineyo koma chidwi chopita kuchipatala alibe ndawayendera mmakomo mwawo koma nsinathe kuwatani kuwapeza enawo apita kumudzi enawo kuntchito koma amangodalira kugula ma indocid mkumamwa nde mmafuna kuwayendera nkuwalimbikitsa kuti inu tapitani kuchipatala mukatani mutakaonana ndi dokotala. Zikomo.

Thank you very much. And also in additional to that I have learnt something here, I could have come with a picture of 2 or 3 people whom I saw that they are struggling just like myself but they do not have an interest to go to the hospital. I had to follow them in their homes but I did not manage to get them one has gone to the Village while the other one to work but they rely on buying and taking Indocid so I wanted to follow and encourage them that, “go and meet the doctors at the hospital.” Thanks.

I: Mmm, zikomo. Aliponso ena amene ali ndi choonjezera?

Mmm, thank you. Is there any other with some additions?

04/02: Eee tili mbali yomweyo ya ujeniyo yamatenda akhansa titaona kuti titayenda kupita kuchipatala chakwathu cha ku Ndirande anati pitani ku Queens kupita ku Queens anati tipezane ndiye ku mwayi wathu cholinga kuti akayeze matenda amene akudwala eti ndiye atakayeza kuja anapezeka kuti mai anga ali ndi khansa ndiye titamva chomchija kuti mai ali ndi khansa tinabwerera tinapita kunyumba sanatipatse mankhwala, tinabwereranso kupita kuja ndiye ndili ndi akulu anga tinalimbitsana mtima kuti aa munthu kukupeza ndi matenda a cancer ifeyo tisakhumudwe koma ifeyo tilimbe mtima tidziachengeta mai athuwa chomwe atatipatse kutsogolo kuno Mulungu sitikuchidziwa olo ena apompo pakwathu pompo amatheka kunena kuti iii awo matenda awawa ndizakuti zakutitu apa sachirayi mwakuti mwakuti ndinawauza kuti inuyo mukunama inu si Mulungu mankhwala amapereka ndani mulungu amathandizira pachinachilichonse nde pali anthu ena amene akupezeka ndi matenda a khansa panopa malo moti kuchipatala kuja ena apatsa mankhwala koma akulimbikiranso ach black. Achimzanga inu amene muli muno maguardian chonde tisatenge mankhwala kumpatsa patient wathu chifukwa choti mwina ena atinyengeza kuti iii imeneyoti mwina winatu wangokutchera angokutani, sizoona koma ngati madokotala atipeza kuti awa ndi khansa tiyeni tilimbikiraneni kumwetsana mankhwala akuchipatala ndipo chimene titachite tizichita kuti titatin tiuze mtundu onse wa Malawi kuti munthu akampeza ndi matenda a khansa sikuti adzilimbana mkumwa achi black koma iye akhale pachikhulupiriro kuti tsiku lina lililonse ndidzachira ndipo ifeyo odwalitsa matenda tisamakhumudwe tidzimuonetsa munthu chilimbikitso chotani chokhala, chosangalala nthawi zonse ndiye patient wathu sadzikhala akukhumudwa.

Yes while on the same cancer disease, when we saw that, when we went to our nearby hospital, Ndirande they said that you should go to Queens, when we went to Queens they said that we should meet at Mwayi Wathu so that we should diagnose what disease is she suffering from. So when she got diagnosed it was found that my mother has cancer.And when I heard that my mother have cancer we went back home without being given medication. We went back there so together with my elder sister we encouraged each other that when one has been found with cancer disease then we should not get worried but we should take heart and take care for our mother whatever God will give us in the future but even some around our home they could be saying that, “iii that disease is like this and that, she will not recover,” I told them that you are not God and it is God who gives medication and God does helps in everything so there are some people who are being diagnosed with cancer but they are using **black** medication rather than that given from the hospital. You my friends in here, the guardians lets not take medication and give it to our patient because maybe some will entice us that aa that is maybe someone has just done that to you, that is not true. Lets work on giving the patient the medicine from the hospital if the doctors have told us that this is cancer and what we should do is to tell the people in Malawi when one is diagnosed with cancer should not be taking **black** medicine but trust that one day I will be fine and we the guardians should not be disappointed but show courage to the person by being happy all the time hence our patient will also not be disappointed

I: Mmm, chabwino. Aliponso ena?

Alright, is there any other?

09/01: Komanso ndimafuna ndionjezere pamenepa pamatenda a khansa pali ma neighbors ena amanyoza odwala matenda a cancer monga ngati ineyo ndimaberekedwa kumapita ku stage kuchoka kustage kupita ku Queens kuchokanso ku Queens kuberekedwa kupita kunyumba, amatha kumanyoza kumalenga malovu pondiona ine kuti ii awa nde amwalira ukoko kukhomo ukoko 3 weeks siitha kuoneka zovuta, iii angomwalira basi anthu ena kumakamba pambali zimenezo manieghbors ena ondiona kumatenga malovu kumalavula pansi ndikulephera kuyenda ndiye tikupempha kuti maneighbors oterewo asamalankhule zimenezi.

But I also wanted to add here on cancer disease there are some neighbors who insults the cancer patientfor example myself I was being carried to the bust stage from bust stage to Queens and from Queens also being carried going home, they could insult, and spit when they have seen me that, “ii that one will die and in 3 weeks time will not pass without a funeral in that house, ii he should just die.” some people discussing that, the neighbors. And some spitting saliva upon seeing me while failing to walk so am asking that these neighbors should not speak these things.

I: Chabwino. Aliponso ena amene akufuna kuonjezera?

Alright. Is there anyone who wants to add?

05/02: China chimene ndikufuna kuonjezerapo nchokuti ine ndili munthu oyembekezera ndinapita kuchipatala chakuZomba ndiye mai anga, nditachira mwana wanga uja anamtenga kukamusiya kumagetsi ndiye mwendo wa mai anga uja unayamba kutupa konkuja ndiye adokotala anaona kuti aaa mwinatu ndi cancer imeneyi ndiye amaabaya jackson kumenekujaku ndiye iwo anachivomereza kumapita mwina pafupi, paweek kawiri, pa week ka week, pafupi pafupi ndingotero eti ndiye anachivomereza koma atafika kunyumba anthu ena monga mazineighbor ena kumanena kuti iii awatu mwina akamalira nawo mwendo uja eti kumanena kuti lerotu timverako lero nde iwobeje samakhala munthu okhumudwa amangokhalabe kuti ine mwendo wangaje bolani ukutani ukundipweteka inde koma khaya ndichira kaya sindichira komabe anali ndi chikhulupiriro mpaka panopa alipo lero oti chaka chino ngati chisali cha folo...(overlap)

What I would also want to add is that when I was pregnant I went to Zomba hospital so my mum, when I gave birth my child was taken to kangaroo so my mum’s leg started getting swollen right there and when the doctor saw it they said that maybe its cancer so she was given injection right there and she accepted it and was going there frequently, two times a week, in a week, I should just say frequently right so she accepted it. When she got home some people like the neighbors were saying iii we will her from her today when maybe is crying because of the leg so she wasn’t taking that into her mind and wasn’t worried but was like my leg is in pain it doesn’t matter whether I will get better or now but she still had faith and she is still alive up to date that this is if not a 4^th^ year…(overlaps)

05/01: 8

8

05/02; Cha 8. Eee

8^th^. Yes

I: Mmm, nde inu uthenga wanu mukupereka nde otani?

Mmm, so what message are you giving?

05.02: Ndiye ndikupereka uthenga okuti mwina mazineighborziwo mwina asiye kuchepetsa, kunena azimnzawo akakhala kuti ali pa chiphinjo. Eee Ndithu.

So am giving the message that maybe the neighbors should stop, or reduce talking about their friends when they are facing problems. Yes, sure.

I: Chabwino chabwino.

Alright, alright.

06/01: Inenso ndingolankhulapo komaliza zimathekadi kuti iweyo ukumwa mankhwala a khansawa basi kupezeka kuti wayamba kupemphera kutchalitchi kuja kaya ukumana ndi abusa kaya ndi azitsogoleri kaya wina aliyense amene amatengapo gawo kutchalitchiko akukupempherera ndikuuza kuti iweyo wachira, mmene ndakupephereramu wachira sukuyeneranso kuti usadzapitenso mwina kuchipatala kaya zamankhwala usazamwenso iweyo basi wachira, mwina kumakupangitsa ma fasting miyezi miyezi mwezi kutha uli mu fasting mwina miyezi iwiri uli mu fasting zinthunso ngati zimenezizi zimafunika kuunikira kwa munthu ngati ifeyo tili ndi cancer sizikuyenera ngati mb'usa uja wakupempherera iweyo iyeyo ali ndi chikhulupiriro chake iwenso uli ndi chikhulupiriro chako ndiye sizoona kuti usiye mankhwala kukuuza kuti basi kuyambira lero usazamwe mankhwala wachira, ndiye kupezeka vuto lija lakolerabe lakula iweyo basi kungokhulupirira kunena kuti paja ananena kuti paja ndachira iyayi basi ananena kuti ndsazamwenso mankhwala aa nanga ndi panga bwanji poti mnalumbira mnalonjeza, Zimene zijanso tiyenera kuunikira anthufe, kupemphera sikokanidwa komabe iweyo paumunthu wako ukuyenera kumapanga zimene iweyo unauuzidwa kuchipatala, pokhapokhapo ngati munthu uja wakupempherera achipatalanso ndikutsimikizakuti munthuyu Osat munthu uli chitupire koma iyeyo akakuuza kuti wachira iwe nkusiyadi pamene pajanso zimatha kupangitsa chani mavuto aja kuti adzipita mtsogolo mapeto ake okuthandiza aja akuthandiza koma iwe careless yako kupezeka kuti nthenda ija ikutanibe ikungopitirira pitirira pamenepanso tikuyenera kuzindikirapo.

I should also talk, it happens that you are taking these cancer medication and found that you have started praying there at church and meet either a pastor or church elders who participate at the church and pray for you and they tell you that you are healed and since I have prayed for you and you are healed you should not go to the hospital again or maybe you should not take the medicine again you are healed and maybe making you fast for months or two, these things needs proper scrutiny from us cancer people if the pastor has prayed for you he has his faith and you have your faith so it is not on to stop taking medicine or being told that you should not take medicine from today onwards because you are healed so it is found that your problem has gotten worse and you believe that he said that am fine, no he said that I should not take medication again, what can I do I did swear, and promised. We people should look at that properly it is not a crime to pray but as a human being you are supposed to do what you were told at the hospital unless the person has prayed for you and the hospital personnels has agreed that yes the person has, not while a person is still swollen but you stop just because he has told you that you are healed that brings a continuation of your problems as a result those who assist you will assist you but it will be found that the disease is getting worse because of your carelessness so we are supposed to have that knowledge.

I: Chabwino.

Alright.

09/01: Nde ndiyankhulenso ine, ma neighbors onyoza patient akudwala monga ngati chitsanzo monga ngati pali bambo amenewo tikuwaona akugwira ntchito wo pali ena ndikukhulupirira kuti amatha kuwanena pachithunzichi maneighbors ena panopa amandiona, amati akandiona ndi mmene mnaliri poyambirira amapanga manyazi chifukwa samayembekezera kuti ndingachire matenda amenewawa ndingachire bwinobwino kukhala moyo.

So I should also talk on the neighbors who insult the patient like for example there is that gentleman there we can see him working I believe that there are some who talk about him. On this picture some neighbors do see me and when they have seen me the way I am comparing to the way I was in the beginning they get ashamed because they were not expecting me to get healed of this disease and stay alive.

I: Mmm

Mmm

09/01: Amaganiza kuti ndifa basi, koma mwa mwayi ndi madokotala pondipatsa mankhwala ndi malangizo amangoti aaa basi uzikamwa mankhwala amenewawa nde ma neighbors ena amakhala kuti ndi achipongwe amayankhula chipongwe ndiye pamene paja amati akandiona amathawa, amathawira kumbali akandiona ena kumandisunzumira kodi amene uja wachiradi khansa ija, uja amaberekedwa uja kodi ndi amene uja ndiye ndikupereka chithandizo kwa ziti kwa zimenezizi.

They thought that am going to dies but lucky enough and by been given medicine and conselling by the doctors they say aa take these medicine so the neighbors have insults they insult so when they see me there they run away, they run aside and some do peep on me seeing that, “he really is healed of cancer, the one who was being carried, is he really the same person,”so am giving assistance on this.

I: Ngati palibe ena ali ndi zoonjezera...(Overlaps)

If there is no any other with additions…(overlaps)

02/01: Mmm ndingowalimbikitsa azimnzanga amene tikumwa mankhwala a khansawo ine ndi munthu mmodzi amene ndimati ndikapanga chomchi mwendo winawu kukhala ngati munthu, mwendo winawu munthu wina mwendo wina wa munthu wina koma malangizo akuchipatala mmene amandiuzira pakali pano ndili bwino anandiimitsa koma ananena kuti file sanatani sanaseke iyayi nthai iliyonse zikandigundika nditha kupita kaya ndikufuna kuchipatala ndimatha kupita koma ndina, malingana ndi kumwa mankhwalawo mwadongosolo mmene anenera achainu akewo kuti ooh muzikamwa mwakutimwakuti nthawi yakuti nthawi yakuti. Mukapanga zimenezozo, mukapanga zimenezozo tidzaona ngati kuti mankhwala aja akugwira ntchito koma dongosolo ngati palibe kumalumphitsa mmene anenera achaini akewo kuti oo mudzikamwa mwakutimwakuti ndekuti matenda, mankhwala aja samagwira ntchito ngakhale timwe, tizingomwa ngati madzi nde uthenga wanga ndi owalimbikitsa azimnzanga kuti tizikalimbikira ndi mmene, mmene akunenera achaini akewo chifukwa mukapita kukanena kuti oh ineyo ndikudwala mwakutimwakuti iwo aja amasintha mankhwala, akasintha mankhwala, mupange mwakuti, eeya ndiye ineyo ndi chilimbikitso cha azimnzanga.

I should just encourage my friends who are taking cancer medication. I am one of the people who when I have done like this this other leg was like for some other person while the other one for another but because of the hospitals advise, the way I was given the advise that now they had to stop me but they said that they haven’t closed the file if it starts any time I can go and I happen to go to the hospital if I want but I did, it was because of taking the medicine in proper way as they say that ooh you should be taking them like this at this time. When you do that we will see that the medicine are working bur without proper procedures, and skipping the way the owners have said oo take them like this then disease, the medicine will not work even if we can take them but we will be taking them as water so my message is to encourage my friends to work hard on what the owners have said because when you go and say that am sick, this and that they do change medicine, and you should do this, that is when they have changed the medicine. Sure so that is a courage to my friends.

I: Chabwino.

Alright.

03/01: Pali amayi ena ake amatifotokozera kuti anthu ena amakampatsa chakudya patient amavala ma glovesi.

There is a certain lady who was telling us that some people put on gloves when giving food to the patient.

I: Mmm

Mmm

03/01: Ati kuopa kuti angapatsire ndiyeno sindikudziwa kuti inuyo pamenepopo zitazachitikanso mwina kwa wina wake munthu amene tingazamulangize motani? Imeneyoyo inachitika mwina ngati trial mwina kumene tikumpeza wavala mwina maglovesi kumulangiza kuti mwina aaa aise wavala bwanji ukuvala ma glovesi apatseni chakudya bwinobwino with bare hands sungatani, sungatenge khansa ndiyeno sindikudziwa kuti inuyo kuti mwina mungafotokoze motani titandzampeza wina wake atavala ma glovesi.

Afraid that they can transmit it to her so I don’t know to you on that if it happen again to some one how can we advise that person? Maybe that one happened as a trial but where we have found some one maybe is putting on gloves the advise that maybe aaa my friend why wearing gloves give the food to the patient with bare hands you cannot get cancer so I don’t know how you can explain it if we found one putting on gloves.

I: Mmm

Mmm.

03/01: Mwina mutatifotokozerako tikazampeza wina atavala maglovesi akupereka chakudya tidzamulangize kuti ndimmene awawa akudwalamu simungatenge mwakuti, mwakuti, mwakuti…

Maybe if you can tell us that when we have found one wearing gloves while giving food we should advise him/her that with the condition of the patient you cannot transmit, this and that…

I: Chabwino. Zimenezo mwina atithandiza nthawi inayake atifotokozera awa amatithandizirawa eti?

Alright. Maybe these people who assist us will assist us on that some other time.

03/01: Owo chabwino.

Ok, alright.

I: Tingomaliza kaye za zithunzizi.

Lets just finish with the pictures.

03/01: Owo chabwino.

Ok, alright.

I: Aliponso wina amene ali ndi mau omaliza?

Is there anyone with last words?

09/02: Mau anga oonjezerawo tingonena kuti chidandaulo cha anthu onse pamavuto amene alankhula eti, Mulunguyo kenaka tingowasiira madokotala kuti chimene atatithandize pamatenda amenewa ndi chani, tingowasiira madokotalawa.

My additional words is a plea for all the people on the problems that they have spoken right, God we should just leave it to the doctors to see what they will help us with on this disease, let leave it to the doctors.

I: Chabwino.

Alright.

04/01: (xxx) ndikumva kupweteka

(xxx) Am feeling pain

I: Tathokoza kwambiri, ndiye tathokoza kwambiri chifukwa munatijambulira zithunzi mwafotokoza nkhani yake...(overlaps)

We are very thankful, so we are very thankful because you took the pictures and has explained the story behind them…(Overlaps)

04/01: (xxx)

(xxx)

All: (laughing)

(laughing)

I: Zikomo kwambiri chifukwa cha nthawi yanu ndikutifotokozeranso zithunzizi eti ndiye tidzikupemphanibe kuti mudzikajambulabe musatope. Ndithu.

Thank you very much because of your time, you have explained the pictures to us right so we will still ask you to take pictures you should not get tired. Sure.

09/01: Koma inenso ndionjezere pankhani imeneyi ya khansayi ndikudwala, mntangoyamba kumene kudwala mnapita ku queens, akazi anga ndinaasiya mnyumba anandithawa chifukwa choti ineyo ndimadwala khansa. Ndinakhala ku QUeens kuja kukhalakukhala kukhala mwina mwake kuthamangira zaka ziwiri, akazi anga ndithaweni katundu yense kuola mnyumba, kuwola mnyumba ankaganiza kuti ine ndimwalira. Mpakana panopa amati akandiona ndili bwinobwino ndikugwira ntchito maganyu anga amandizemba amathawa. Mmalo moti andilankhule andipatse moni, amandithawa

But I should also add on the cancer issue, whe I was sick, when I started getting sick I went to Queens, I left my wife at home and she run away just because I was sick from cancer. I was at Queens for maybe 2 years, and my wife had to run away from me and took everything in the house, took everything in the house she was thinking that I will die. Up to date, when she sees me okay and working on my piece works she goes and run away so that I should not see her. Other than to talk and say hie to me she runs away.

09/02: Anathawadi, ati ndukakolora.

She really run away saying am going for harvesting.

09/01: Omwewo, anapita, anandithawa. Panopa amati akandiona amathawa amachita manyazi koma mankhwala...(overlaps)

That one, she went, she run away from me. When she sees me now she runs, she is ashamed but the medicine…(overlaps)

M: koma pano nkuti akuchita manyazitu.

But now she is being ashamed right?

09/01; Eya amachita manyazi.

Yes, she gets ashamed.

09/02: Anathawa kuti ndukakolora.

She run away saying am going for harvesting.

09/01: Anandithawa mkaziyo kuthawa mnyumba basi ine ndili kuchipatala, anandithawa.

She run away from me, she run away from the house while I was at the hospital, running away from me.

I: Chabwino.

Alright.

09/01: Nde panopa amati akandiona ee kodi ndi omwe ajadi awa chifukwa simnali chomchiyi mpakana panopa ndiye ndimakhulupirira kuti kukhala ndi moyo odwala khansa sikuti ndi chiyambi cha kufa iyayi.

So when she sees me now, eee is he really that one because I wasn’t like this up to now, so I believe that having cancer life is not the beginning of death.

I: Mmm

Mmm

09/01: Eeetu

Sure

I: Tathokoza kwambiri.

Thank you very much.

I2: Tidziombere mmanja tokha

We should clap hands for ourselves.

All: (Clapping hands)

FGD4

I: Tikutha kuona tonse chithunzichi eti

We are all able to see this picture right

All: Mmm

Mmm

I: Mayiwo sabwera nawo? Atopa kapenatu, ayi asiyeni. Chabwino ndiye tasanjasanja zithunzi zathu nkhani ndiyomweija tizifotokoza kunena kuti kodi tikufuna kuwauza chani anthu ena kapena tikufuna kuti tingafotokoze bwanji za kukhala ndi matenda akhansa kuuza anthu ena zokhuzana ndi matenda akhansa ndiye pamagulu amene mwaika zithunzizo khalangati mwanena kuti tiziyambira uku eti

Is the lady not coming? Maybe she is tired. (interruptions) No leave her. Alright so we have put our pictures in place and the issue is still the same we will be discussing what we want to tell other people or what we want to, how can we explain about having cancer, telling other people about cancer disease, so you have said that we will be starting this side on the picture categories you have right.

PS: Mmm

Mmm

I: Kapena tiyambire uku (laughs)

Maybe we should start from here (laughs)

PS: Muyambire uku

You should start from here

I: Chabwino tandifotokozeleni za gulu loyambilira lomwe mwaika apo kuti ndi gulu lotani pali zithunzi zokhuzana ndi ndani

Alright can you explain to me about the first category there, what category is it and what pictures are there?

PS: Zonsezo zilipamenepo

All of them

08/02: Awawa ndi ma peshenti sanadzuke bwino akumwa mankhwala

These are the patients, they did not woke up well they are taking medicine.

I: Mmm ndiye ndinkhani yanji imene mungafotokozere anthu zokhuzana ndi gulu limene mwaikalo lokhuzana ndi mapeshenti sakupeza bwino

Mmm, so what can you tell the people about this category there concerning the patients who are not fine?

08/02: Tabwelezani

Can you repeat

I: Ndi nkhani yanji imene mungathe kufotokozara anthu zokhuzana ndi gulu limenelo mwaika pamenepolo

what can you explain to people about the group that you have put there

09/02: Ndikupezeka kuti ngati peshenti wathu aasanadzuke bwino patsiku limenelo tiyenekera kutenga mankhwala ndikumupatsa peshenti wathuyo kuti amwe mankhwalawo ndendikuwafotokozera azinzanga kuti ife monga magadiyani tiyenera kutenga mankhwala kuwapasa odwala wathu kuti amwe mankhwalawo

if it happens that the patient wakes up unwell on that day we are supposed to take medicine and give our patient so that they should drink the medicine so am explaining to my friends that we as guardians we are suppose to get the medicine and give our patient so that they should drink them

I: Ena

another one

07/01: Gulu limeneli ndi odwala nde gulu limeneli likumva ululu mkati mwa thupi lawo nde tikuwauza anthu kunena kuti asamafulumize kuseka munthu akamuona akulira chifukwa akumva ululu nkati mwathupi lake ndeno uthenga umene tikufuna kuwauza kuti pamenepa ali pakati pa ululu ndithu monga ma peshenti

This is the patients category who are feeling pains inside there body so we are telling people that they should not be laughing when they see some one crying because they are feeling pain inside there body now the message that we want to tell is thaat they are feeling alot of pain herre as patients

I: Ena tikuti bwanji

what are others saying

09/02: Mapeshenti amenewo tikhoza kunena kuti tikuwapasa mankhwala ndiye kuti sizikugwirano ntchito pamankhwala paja eti kuwapasa ndiye timatha kuwatengera ku chipatala kuti mankkhwala alipo adokotalaso akatani akawaone

that patient we can say that we are giving them medicine so it is not working on the medicine that we are giving so we get them to the hospital where there are medicine and the doctor to see them

I: Palinso nkhani yina imene mungatheso kufotokoza zokhuzana ndi gulu limeneli

is there any story that you can explain about this group

06/01: Gulu limeneli ndila mapeshenti amene akudwala eti matenda akhansa komano anthuwa akusowekera mankhwala enieni oti achile bwino bwino mankhwala amene timawapeza ndiongozizilisa ma pain killer akatero tikakhala bwino lero mawa ndi nkucha linalo tagundikaso nde gulu limenelo ndi gulu la odwala khansa amene tikusowa chithandizo chenicheni choti khansayo izichililatu nde gulu lake ndilimenelo nde anthu akhoza kuona kuti nthenda ngati zimenezi zikufunikilaso anthu ena atengepo gawo kuti athandize ndithu pa nthenda imeneyi sizikuimira gadiyani yekha sizikuimira dokotala okha komabe zikuimira onse akufuna kwabwino amene angatengeko gawo kuthandizana ndi nthenda imeneyi mudziko lathu

This is the patients category who are suffering from cancer but these people are missing the real medicine that can heal them properly the medicine that we are giving are just pain killers so they get better today but tomorrow and the other day they become sick so this group is a group of people who are suffering from cancer who are missing the real care that the cancer can be totaly healed so thais is the group and people can see that for the disease like this it needs people to get a side in helping for the disease it is not for the guardian only or the doctor but it is also for those that has good wishes to take part in fighting for this disease in malawi

I: Anthu ake ngati ndani

people like who

06/01: Anthu amene akhoza kukhala akufuna kwabwino kaya ndi mabungwe kaya ndi ena onse amene amaona kuti ineyo ndikhoza kuikapo gawo pomuthandiza munthu anthu ngati amenewowo akhoza kuimapo pa nthenda imeneyo ndikuthandizana monga ndimadokotala athu kapena kuchipatala kwathu kumene timapita ndi mpakana pofika kuti apeze chithandizo chokwanira cheni cheni

people who can be well wishers wheather organisations or everyone who hs the feeling that they can help people like these ones can put a hand on this disease and help like our doctors or in the hospitals that we usually go upto they find proper help

I: Mmm nde kaya ndi mabugwe kaya ndi anthu akufuna kwabwino akhoza kutengapo gawo lanji limeneli

Mmm whether organisations or well wishers what role can they play here?

06/01: Gawo limene angatenge ndi lothandizana nawo kusakasaka mankhwala chifukwa timamva kuti kwa anzathuku mankhwala alipo nde mwina anthu akufuna kwabwinowo wina akhoza kupezeka ndithu kupeza chithandizo choti kaya ndi ndalama kukagula mankhwala amene aja kumazapeleka mu zipatala zathu mu tiyanjanemu kuti mankhwala amenewa atifikile ifeyo

the part that they can take is to help in searching for the medicine because we hear that in other countries they have the medicine so maybe the well wishers can have a chance to find them and help in form of money to but medicine and give out in the hospitals like tiyanjane so that we can access them

int-palinso ena amene akufuna kuonjezera gulu loyambililari

is there anyone who wants to add on the first group

08/01: Aaah gulu loyambililari tikuona kuti ndi munthu amene akudwala matenda akhansa ndipo kuganiza kwanga munthuyi ali ku nyumba ndipo amatendawo sakudziwa kuti ndi chani nde uthenga umene uli pamenepapa ndionena kuti titha kuwauza anthu za nthenda ya khansa chiyambi ndi zizindikiro zimene zimaonetsa kuti munthu asachedwe ndikhala kunyumba kapena kuchedwa ndi zina ndi zina koma ayenera kupanga chiganizo mwansanga kupita kuchipatala kuti akauzidwe za nthenda imene alinayo ndicholinga chakuti apeze chithandizo mwansanga

aaah in the first category we can see that a person who is suffering from cancer in my thinking this person is at home and they dont know what this sickness is so the message that is here is that we can tell people about this cancer disease how it starts and the symptoms that are showing so that a person should not hesitate by staying at home or with other things but they are supposed to make a decission early to go to the hospital so that they should know the disease theyb are suffering with the reason that they should find help earlier

I: Nde mwati mutha kuwauza pachiyambi ndizizindikilo zotani zimenezo

So you have said you can tell them early what are those symptoms

08/01: Tiyambe ndi zizindikilo aaah monga kumva kwa ine mmene ndinamvera komanso nditapita kuchipatalako ndinakaona ma poster akufotokoza kunena kuti pali zizindikilo zimene zimaonetsa kunena kuti itha kukhala nthenda ya khansa one ngati kutuluka zotupa ku maso zosapweteka ngakhale pamwendo komanso kufiila kwa nkamwa kapena kuda nkamwa kapenanso kufiila kuphazi kapena kuda kuphaziko zizindikilo ngati zimenezi ndi zimene zimakathandizila kuti nthenda imeneyi itha kukhala khansa poona zimenezo muthamangire mwansanga kuchipatala kuti mukathe kukalandira uphungu kapena kulangizidwa za nthendayi

let us start with the symptoms aaah like how i heard but also when i went to the hospital i saw posters explaining that there are signs that shows that this can be cancer one if the face is swelling but not painfull also having red or black color in the mouth or having red or black color on the feet with these signs it helps to know that this can be cancer if you see this you need to rush to the hospital so that you can recieve counselling on this disease

I: Owo, chabwino. Aliponso ena amene angathe kuonjezera

Ok, alright. Is there anyone who can add on this?

04/02: Khansayi ilipo yamitundu mitundu nde ena imabwera ndikupanga monga ngati imapanga imamanga mmimba monga ngati ine sindimazindikila kuti amayi akudwala chani nthawi yoyamba amakhala akudandaula kuti mmimba mwanga sindikupita ku toilet amakhala mwina one week osapita ku toilet kuja ife kumangolimbana ndichani ndi magnesium tati iii mwina mpweya wanu siukutani siukuyenda bwino basino chonchija kumakhala mwina kumakhala ndithu mwina angopita ku chimbudzi kuja kulephera kuti achite chimbudzi mwina angoti ndingochita chimbudzi cha ujeni cha mbuzi mmene takhalira muja sakuzindikila kuti ndi chani nde panopa tizindikilako kuti khansa imabwera mosiyana munthu mmimba ukamakhala siukupita ku toilet imakhalaso ili kuyamba cha ujeni ili ya khansa yapa ujeni yapakhungu monga ngati ya amayi inawatuluka pakhosi ndipamene inayambano kutupa kumaonesera chani

There is a lot of types of cancer to others it starts like the way it happened to my mother it was giving her problems in the stomach like for me i did not know that what is my mother suffering from at first she was complaining i am not visiting the toilet she could stay maybe one week without going to the toilet so we were jus giving her magnesium we thought that she has a problem with her breathing but now we were just staying like that maybe she could go to the toilet but failing to do feases so it is also the beginning of skin cancer like the one my mother had on the neck its when it started swelling and showing

I: Chabwino titha kupita kugulu linalo

alright we can go to another group

Ps: Eee

Yes

I: Gulu lachiwili tatifotokozeleni nkhani yomwe ikukambidwa pa gulu limeneli

on the second group can you tell us the story that is being said on this group

09/01: Gawo limeneli ndi ma gadiyani pamenepo akudandaula odwala khansa kuti munthu ameneyi kapena mwananga ameneyi kapena agogo anga amenewa achila nde pachithunzipo gawo lachiwiri likukamba choncho

on this section are the guardians that are worried about those who are suffering from cancer that this person or my child or my grandparent are they going to get well so on te second that's what it is saying

I: Nde ndiuthenga wanji umene tingathe kupereka ku dziko okhuzana ndi gawo limeneli

So what is the message that we can give to the country about this section

06/01: Anthu enanu muzilankhulako zinthuzi siza anthu awiri

you others should also be speaking because these pictures are not for these two

MK: Mmm mukuyamba kumenetu tiyeni

Mmm you are just starting so let's go on

All: (laughing)

(laughing)

03/02: Apapa zikutipasa kuti magadiyani asamade nkhawa mwansanga iwowa nde ndiofunika kuti aziyesesa kuti kumamuthandiza peshenti mosalekeza iwowajaso akakhala kuti akuda nkhawa nsanga sizabwinoyi penapake nkhawa tiziichepesa kapena kuichosa

Here it is giving us the guardians that we should not be worried that these needs to be helpfull to the patient continuous because if they are also worried it is not good somehow we need not to be worried

I: Ena ndi uthenga wanji umene mungapereke kuchokera pa gulu limeneli zithunzi zimene taika apazi

The others, what message can you give from this category on the pictures that we have put here?

08/01: Uthenga umenewu ukupita kwa gadiyani ngakhalenso munthu win aliyense amene angathe kukhala ndi peshenti koma nthendayo nikhala ya khansa chifukwa nthenda ya khansayo ndikhulupilira kuti ambiri amaitenga ngati kuti ndi chinthu choophya kwambiri chimene chimatha kuzunza ngakhale omuyang'anira komanso amatha kuona ngati kuti angathe kuitengera koma ngati walandira uthenga umenewu kuti owoo peshenti wako ndi khansa asaonetse kukhumudwa alimbike mtima kumusamalira munthu uja ndichithandizo chimene angathe kumuta chengera chifukwa ameneyu ndi amene ali ndi udindo omutengera peshenti kupita naye kuchipatala olo atadziwa zimene zija za nthenda imeneyo ndikumene akamve malangizo ambiri chifukwa mwini malangizo kulandira malangizo mwini wake ndinameyu amene amayang'anira ndani peshenti ndipo akhale olimba mtima sikuti ndi gadiyani yekha iyayi tikati gadiyani ndekuti ndiyekhayo amene amakhala naye pafupi ngakhale aliyense amene wamuzungulira munthu uja akamamulimbikisa munthu uja zimakapangisa kunena kuti munthu uja akathe kukhala ndi moyo wamphamvu moyo olimbika mtima ngakhale alindi vuto akazionesa kukhumata choncho zimakathandizaso kuti azikhalaso okhumata kumaona ngati iyeyo ndi mathero achani, nde uthenga umenewu ukupita kwa munthu wina aliyense amene alindi pafupi ndi odwala khansa asaone ngati khansayo ndimathero a moyo wa peshenti wake kapena m'bale wake kapena mamuna wake kapena mkazi wake azikhala wachisangalaro mmene amakhalira nthawi zonse

this message is going to the guardian and also people who takes care of the patient but the disease being cancer because with the cancer i beleie alot of people get it as a very dangerous thing that troubles even the guardians but also they think that they can get it from the person suffering but if they get this message that owoo the your patient with cancer should not show to be worried they should continue to taking care because this one is the one who has the authority to take the patient to the hospital even if they know about this disease it's where they will hear alot of advices because it's where you can get the real advices the one who takes care of the patient and they should be strong it's not the guardian only if we say guardian it's not the one who stays close to the patient anyone who is sarounding this person if they are being encourage it makes that a person can leave with a strong life with courage although they have this problem if they show to be worried it helps to make them be worried as if its the end of what, so this message is going to everyone who is near the patient suffering from cancer they should not see that the cancer is the end of the patients life or a relative or a husband or a wife they should be happy the way they stay every time

I: Chabwino ena

Alright others?

07/01: Tikuona kuti tikayang'ana pamenepa gulu lachiwiri tikuona kuti akudandaula amene akukhala nawo akuganiza kuti kodi amenewa akhala ndi moyo chifukwa chakuti akudandaula kwambiri za amuna awo komabe poti matendawa alinawo moyotu umakhozabe kumasintha koma tikayang'ana pamenepa ndithu gulu limeneri ndiye likudandaula kwambiri ndi matenda amene akudwalawa

if we can see on the second category they are worried with who they are staying with they are thinking that, will this one stay alive because they are worried a lot about their husband but still although they have this disease life can change but if we look at that this category the its worried about the disease they are suffering from.

I: Palinso ena amene angathe kuonjezera? Tikupita ku gulu linali eti tifotokozeleni gulu pali zithunzi zambilipo

Is there anyone who can add? We are going to another group right where there are a lot of pictures

03/02: Aaah gulu limeneli likusonyeza kuti mapeshenti…(Overlaps)

Aaah this group is showing that patient…(overlaps)

I: Oh iyayi tili kaye apa eti?

Ooh no we are here first right?

03/02: Kukhalangati ndizimodzi izi

it seems that they are the same.

06/01: Eee

Yes

03/01: Ndi izi ndizimodzi kusonyeza kuti mapeshenti tsiku limenelo adzukako bwino chifukwa akuoneka kuti ali choimilira ena akuchapa ena akuyenda kumene kusonyeza kuti atleast tsiku limeneli adzukako bwino

They are the same with these meaning that on that day the patients woke up well because they seem to be standing others are washing and others walking showing that at least on this day they woke up well

I: Apeshenti

The patient?

03/01: Yaah apeshenti adzukako bwino chifukwa ambiri zithunzizo nzoti ayimilira akuyenda ndithu kapena angoima

Yes the patient woke up well because most of them on the pictures are standing walking or they have just stood up

I: Chabwino nde ndi nkhani yanji imene mungafotokoze kuuza anthu ena zokhuzana ndi zithunzi zili pamenepozo

Alright so what story can you give to people about those pictures?

07/01: Chimene chikufotokozedwa pamenepa ndichonena kuti kuchokera kumayambilira kuja odwala kuja kufika pachiwiri pakudandaula kwambiri kuti amenewa akhala ndi moyo kuzafika pachitatupa kusonyeza kunena kuti mankhwala amene akulandira ku tiyanjane kuja ayamba kugwira ntchito mkati mwa thupi lawo ndi chifukwa chake mukuona kuti ayamaba kuma zithandizo kumachapa ena aimilira kusonyeza kunena kuti mankhwala aja ayamba kugwira ntchito mkati mwa thupi lawo

what is being spoken here is that from the first place the patient to the second place they are very worried will they be alive until the third time where it shows that the medicine they are receiving from tiyanjane have started working in there bodies because you can see that they have started helping them selves with washing others are standing up showing that the medicine have started working in the there bodies

I: Nde ndi uthenga wanji umene tikupeleka kwa anthu mogwilizana ndi zithunzi zimenezi

So what message are giving to people together with these pictures

08/01: Aah uthenga ulipamenepo ndi uthenga ukupita kwa wina aliyense amene alinaye pafupi ngakhale ali patali amene akudziwa za nthendayi kunena kuti ngakhale munthu atapezeka ndi matenda akhansa koma ngati akulandira chisamaliro kapena ulangizi kapena uphungu ndimankhwala ake atha kupeza bwino ndikuchira ndikumayenda ndikumakhala momasuka pachinthu chinachilichonse zimene zingathandizileso awa akudandaulawa kudandaula a peshenti wawo kunena kuti peshenti wawo akutani wachila ndipo akuchira zikhoza kuwachosa kumene aliko ndikufika stage ina nde uthenga uli pamenepopo kwambiri ndiolimbikitsa kwa iwo amene akupeleka mankhwala komanso zithandizo ngati pali zina zoonjezera awonjezere pamenepo kunena kuti mapeshentiwo pamene afika size iyoyo chifukwa atha kufikaso size yothamanga kumene ndikuyamba kugwira ntchito zawo momasuka chifukwa chachilimbikiso cha anthu awa ndi anthu amene angathe kutithandiza pazinthu zosiyana siyana komanso kuthandiza kuthokoza chifukwa imeneyo ndi mbali imodzi yothokoza chithandizo chimene amapeleka a tiyanjane pomulandila peshenti wawo ndikumusamalira bwino ndikumupasa mankhwala mogwilizana ndimmene iye akumvera pa tsiku limenelolo komanso osamunyoza zimapangitsa iwowaja amamulimbikisa peshenti ngakhale gadiyani kupezeka kuti munthu wafika size imene aliyi

aaah the message that is here is that it is going to everybody who is near the patient although they are far to those who knows about the disease saying that if a person can be found with cancer but if they are receivng care or counselling with there medicine they can start getting well and and be healed and start walking freely on everything that can also help these ones who are worried about there patient saying that there patient is now ok and is getting better it can move them from where they are to another stage so the message here is to encourage those that are giving medicine and help if there are other things to add they should add so that the patient reaching that point they can also be running and start doing there works properly because of the encouragement from these people who can help from different things and also help to thank because this is one way of thanking the help that they give from tiyanjane on receiving there patient and taking care of them and giving them medicine with the way they are feeling on that day and also not letting the down that makes the patient to be encouraged and also the guardian to see that the patient has reached at this size

I: Ena nanga ndi uthenga wanji, basi uthenga wake ndiomwewo tipite kwina, zithunzi zili apazi

Others what is the message? Thats the only message we should go to another one, these pictures here

09/01: Zithunzi zimene zili apazi tikutha kuonna kuti ndi magadiyani amene akusangala kuti peshenti kapena amene akusungidwa ndi munthu odwala khansa nde apapa chisamaliro chake akusangalala kwamiri kuti aah peshenti wanga wadzuka bwino akuyenda akumaphika nde pamenepa akusangalala kwambiri

The pictures that are here we can see that they are guardians who are happy that the patients that are being taken care of with the person suffering from cancer here there care, they are very happy that aah my patient has woken up ok they are walking, cooking so here they are very happy

I: Ena tikuti bwa

what are others saying?

05/01: Apapaso zikusonyeza kuti ndimankhwala amene timalandila ku tiyanjane kuja nde ngati tikufika size yoti tikudzuka ndikumayenda chonchi ndimmene tikusangalala aku tikusangalalira pamenepapa kuti zinthu zili chani zili bwino ndeno kutanthauza kunena kuti anthu amene ali apapawa akusangalala ndinene kuti ifeyo tikusangalala kunena kuti mwina mwake tadzuka bwino kapena ma gadiyani athu mapeshenti athu azuka bwino nde apapa ukuku tikusangalala kaya tinali mu chipatala ndeyekuti pamenepapa tikusonyeza kuti tikukatuluka tsopano chifukwa choti chinachilichonse apapa chikuoneka kuti chili bwino ndichisangalalo chokhachokha nnali mu chipatala lero ndazuka bwino kapena lero adokotala akutitani akutitulusa ndi chithandizo chochokera kuti ku tiyanjane nde apapa kusangalala kokha kokha kuti aah panopa peshenti wanga watuluka kapena ine amene gadiyani amene tikutuluka ndi peshenti wanga tikupita kuti tikupita kunyumba ndiye apapa ndichisangalalo chokhachokha chimene chili pamenepapa

Here it's showing that it's the medicine that that we are receiving from tiyanjane so if we are up to this stage that we can wake up and walk like this it's when we happy with people from tiyanjane here so that things are ok now meaning that these people here they are happy let me say we are happy maybe that we have waked up ok maybe our guardian our patients have woke up ok now here we are happy whether we were in the hospital now there it's showing that we are going to be discharged now because anything here is showing that it's ok with the excitement that was in the hospital but now i have woke up well mmaybe today the doctor is discharging us with the help from tiyanjane then here it's all the happiness that aah my patient has been discharged maybe it's me or the guardian we are getting out with my patient going home now here it's all e happiness that is here

I: Nde kaya ndi uthenga wanji umene mungawauze anthu ena zokhuzana ndi gulu limeneli tingawauze chani anthu mdzikoli

So what is the message that you can tell other people about this category what can we tell people from this country

05/01: Apapa tingawauze anthu ngati tili ndi peshenti odwala khansa tisakhale anthu otaya chikhulupiro kwambiri tizipita kuchiptala kukalandila mankhwala tikalandila mankhwala mapeto ake tiona kunena kuti tachila ndithu tilibwino tikubweleraso kuti kubwelelanso kunyumba koma ngati tikudwala osapita ku chipatala kapena ayi ma gadiyani osatenga peshenti wawo ndikupita ku chipatala pamenepopo ndekuti zinthu sizikutani sizikuyenda nde apapa tikuuza anthu kunena kuti ngati peshenti akudwala ayenera kupita ku chipatala mapeto ake achipatala kapena a tiyanjane aja azatithandiza mapeto ake tizasangalala mmene akusangalalira amenewawa kunena kuti aaah apapa zinthu zija zilicha zilibwinono

here we can tell people if we have a patient who is suffering from cancer we should not be people who looses hope we should be going to the hospital and receive medicine when we receive the medicine at the end we will see that we have been healed we are ok and we are going back home but if we are sick and we are not going to the hospital maybe the guardians not taking there patients to the hospital there it's where things are not working properly so here we are telling people that if the patient is sick they are supposed to go to the hospital at the end people from the hospital or from tiyanjane they will help us and we will be happy the way they are celebrating to say that aaah now things are ok

I: Ena ndi uthenga wanji umene tingapeleke ku dziko okhuzana ndi anthu amene akusangalala

Others what message can we give to the World about these people who are happy

08/01: Kutelo apapa tikuona kuti kuchokera mfundo ya nambala two anthu akudandaula kwambiri kuzafika size iyi kusonyeza kuti maganizo alinawo kunena kuti aah odwala wanga uja wadzuka bwino ngati wafika size yoti wayamba kuyenda yekha odwalayo ndichifukwa chake afika size iyi ndiyebe tikuwauza aaah gulu lonse kunena kuti pamene mzathu akudwala asamakhale ndimaganizo okhumudwa kwambiri asamakhumudweso kwambiri ndichonena kuti papikicha ena akudandaula koma mapeto ake omwewonso akusekelera kusonyeza kuti mankhwala akulandilawa akugwira ntchito mkati mwawo

Here we can see that from the second point people were very worried reaching this point it shows that they have ideas that aah my patient has woken up okay if they have reached this size that they have started walking alone that the patient has reached this size but still more we are telling aaah the whole group that if our friend here is sick they should not be having stress worried alot they should not be worried alot with the reason that on the picture others are worried but in the end the same people are smiling showing that the medication they are receiving are working inside them

I: Ena amene ali ndi choonjezera pa nkhani ya anthu amene akusangalala

others that have anything to add on the story of these people who are excited

06/01: Ine ndionjezere kuti kuchokera uko munthu wodwala wayamba kupeza bwino apa anthu akusangalala chimene tingauze anthu ndichokuti anthu awawa kuti apitilize kusangalala kwawo kukuyenera kuti a peshenti aja apitilizeso kutani kukhala bwino zikhoza kutheka kusangalala kwa ma minitsi mommuja ndayankhula kale mapeto ake zikayambilaso anthu ajaso kuyambaso kudandaula nde tikhoza kuwauza anthu apapa kuti aziwe kuti anthuwa kusangalala koma akufunabe kuti chisangalalo chawo chipite msogolo patapezeka mankhwala kuti anthuwa achililetu chifukwa zikhoza kutheka anthu omwewaso kuzakhala gulu lodandaula chifukwa choti peshenti uja wayambilaso zimenezozo zikhoza kuwapangisa kubwelera podandaula koma kuti kusangalala kwawo kupitilire pamozi ndi mapeshenti omwewo kuti kuchila kukhalepo ndikamba chisangalalo chawo chizakhala chosefukira kuposera apapa

Let me add that from where the patient has started getting better it's when people become happy what we can tell people is that these people if they want to continue being happy it needs that the patient should continue to be okay it can happen to be happy for few minutes the way i have said that in the end if these people gets sick again people will start again in getting worried so we can tell people that these people are happy but they want there happiness to continue if there can be medicine for these people to be completely healed because it can happen that the same people can be in the group of worries because the patient is sick again that can make them worried again but if we want there happiness to continue together with the patients so that they should be well i am saying that they will be very happy more than this

I: Ena ali ndi choonjezera

Anyone who has something to add.

08/01: Mmm aaah pamenepo ndipoti kumbali ya kuthokoza kusangalala kumene akusangalala anthu amenewo ndimbali yakuthokoza chifukwacha chisamaliro kapena uphungu umene amatha kupeleka ku tiyanjane chifukwa akanakhala kuti anthu amenewa samapereka chikondi ndi uphungu wabwino sakanafika pa msinkhu umenewu mwinaso akanakhumudwa kuposaso pati posa apa ndiye kusangalala kwa anthuwa kukupeleka uthenga chilimbikiso kwa iwo amene amapeleka chithandizo chawo komanso kwa iwo anapeleka chiganizo chokhazikisa department imeneyi ya tiyanjane komanso kupeleka chiganizo chopereka zina zimenezija kuti titati tione uku kwa odwala kunazabwera kulinganisa kwa gadiyani chifukwa chakuchita bwino ukalumikiza pang'ono gadiyani ndi odwala kuchoka pamenepaja kwa lumikiza anthu onse apabanja kapena anzake onse kuwalumikiza pamozi nkuwayanjanitsa chifukwa cha uphungu ochokera ku tiyanjane nde ndi ubwino pomaliza pake ndingonena kuti zikupeleka uthenga okutino okhuzana ndi department ya tiyanjane kuti imathandiza anthu amene akuvutika ndi matenda a khansa

Mmm aaah there it's about thanking and happy saying that the happiness of these people is interms of thanking them because of the care or the counseling that they give at tiyanjane because if these people were not sharing the love and good counseling they couldn't reach this point maybe they could be worried more than this so the happiness of these people is giving the message, the courage to those who give their help and also to those who gave an idea to start the tiyanjane department and also giving those others that if we can see its a bond between the patient and the guardian because if you have the bonding with the guardian and the patient from there creating a bond to all family members and also all the friends together because of the counseling from Tiyanjane then it is good in the end i will just say that the message about the department of Tiyanjane that it is helping people who are suffering from cancer

I: Nkumasangalala nkuti anthu nkumasangalala

And are happy, the people are happy then.

08/01: Eee nkumasangalala

Yes and are happy

I: Chabwino palinso china tipite pa gulu lapamwambalo ilo uko

Alright is there anything else, we should go to that category at the top that one there

05/01: Gulu ilili ndi lama transipoti ali nkati mosangalala anthu aja anali oti anali nchipatala nde atulusidwa kuzera ku mankhwala amene tikulandila ku tiyanjane atigoneka ndipo atitulusa kaya mapeshenti kaya magadiyanife ndekuti mapeto ake timapezano ma transipoti oti kaya akutali kaya akuchilomoni kaya akuti nde timapeza magalimoto okuti tikwere galimoto tizipita kuti tizipita kunyumba tachilisidwano tikusangalala apano tikukapezano transipoti yopitila kuti yopitila ku nyumba ndiye gulu limene lili apali ndi lamatransipoti

This is a transport category while they are still happy that they were in the hospital and they have been discharged because of the medicine that they were receiving from Tiyanjane after being admitted and have been discharged whether the patients or guardians then we get transports so that those from far whether from chilomoni or somewhere should find cars to ride on going back home. we are healed now we are happy and now we want to get a transport going home so this the transport category..

I: Chabwino nde ndi uthenga wanji umene tingapereke kwa anthu zokhuzana ndi kukhala ndi matenda a khansa

Alright now what message can we tell people about having cancer

08/01: Aah tionjezeleko pamenepa ineyo ndikuona ngati ameneuja ndi mlumikizi kulumukiza pakati pa peshenti ndi ku tiyanjane mogwilizana ndi mtunda umene munthu alili chifukwa ngati walandila vuto ngati ili choyambilira umaganiza za ulendo wakumene ungathe kupeza chithandizo ndipo ukafika bwanji nde masiku amakono ano kugwilitsa kwake njira yake ndi yitino ndi imeneija aah ukagwiritsa ntchito njira imene ijayo mwachangu kukapeza a tiyanjane chifukwa a tiyanjane aja akakupatsa uphungu ukapezaso tanthauzo ndipo ukapezaso ubwino umene ukapezeke kumenekujako uthenga umene uli pamenepajapo wina owonjezera ndiopeleka omwewo a tiyanjane kaya kuli kwina kumene amatenga zimene zijazo kunena kuti atatithandizilako kunena kuti atichepesele maulendowo chifukwa maulendowo amakhala atali atali chifukwa ena akuchoka kutali ndeno akakatipezera kuwapezela chithandizo chawo kumene ali ndikhulupilira kunena kuti umeneujawo utha kuchepelapo atapezeka kuti akupeza chithandizo chapafupi

aaah let me add here i think that that one is the one who creates the bond between the patient and tiyanjane clinic together with the way a person is because if they have a problem like this one the first thing you think of is going where you can get help and how will you go there now with these modern days you use that way aah you uses that way because if you use that way quickly and get help from tiyanjane because people from tiyanjane will give you counseling and help and also there are some benefits found there. The message to add there the people from tiyanjane maybe there is other place where they get those other things saying that if they can help us so that we should not be walking long distances because the distances that we walk are very long since others come from a far so if they will find the help from where they are staying I believe that it will be a very short distance if they can be getting help a very near place.

I: Chabwino pali china choonjezera

Alright is there anything to add

08/01: China choonjezera?

Any thing to add?

I: (laughs) nde tipite ku gulu lili apali kukuoneka nyumba zamakono kumeneku tatitsogoleleni inu mwayamba kugona

(Laughs)So we should go to another category which is here where we can see good planned houses there can you lead us, you are sleeping.

02/01: Owoo tikugona eti

Okay, we are sleeping right.

All: (laughing)

(laughing)

I: Tatifotokozeleni za gulu limeneli.

Tell us about this category.

02/01: Gulu limeneli titadwala dwala uko tikusangalala ngati tingapeze kuti kusangalala kukupitilira kumapeza ntchito monga apa mukuona pali mageni pamenepa ma banki ake ndi awo oti munthu atha kumakasunga ngati munthu ali bwino bwino ngati munthu sakupeza bwino bwino ku banki sangapite kusowa mphamvu zoti ndalama yoti angapitire ngati akukanika kuzithandiza yekha ndekuti ndi ku bankiso sangapite ndiye ndizimenezija

In this category after being sick then being happy , if it can be found that the happiness is still going on and we are getting jobs as you can see here there are businesses and those are the banks where a person can keep money if the person is okay and if a person is not feeling well they can not go to the banks they don't have the energy to go and make money if they are failing to help themselves that means they can not go to the bank so that's the one.

I: Mmm chabwino ndiye ndi uthenga wanji umene tingapeleke kwa anthu okhuzana ndi zithunzi zimenezi ndikukhala ndi matenda a khansa

Mmm alright so what message can we give to people about these pictures and suffering from cancer

02/01: Zithunzi zimenezi uthenga wake woti munthu ngati ukupeza bwino ndekuti uyenera kumapita ku banki kuja koma ngati usakupeza bwino sungathe kumakagwira ntchito inayake sungapite ku banki ayi eya koma uwuwu ndi ubwino oti munthu ngatii akupeza bwino ayenera kupita ku banki

The message from these pictures is that if a person is getting better is supposed to be going to the bank but if you are not feeling well you can not go to work then go to the bank, no but this is good if a person who feeling well is supposed to go to the bank

I: Mumadwala mwapeza bwino

You were sick and now you are okay.

02/01: Eyaa

Yes

I: Muyenera mupite ku banki

you are supposed to go to the bank

02/01: Eyaa koma munthu akudwala sangapite ku banki kukasiya ndalama azipeza bwanji poti ntchito munthu ali ku chipatala

Yes but if a person is sick they can not go to the bank and deposit money how will they find the money when a person is in the hospital

I: Chabwino nanga ena tingawafotokozere chani anthu zokhuzana ndi gulu limeneli ndikukhala ndi matenda akhansa

Alright so what can others explain to people about this category and suffering from cancer

03/02:Apopo ine ndingafotokoze kunena kuti ma banki ndi awina aliyense kaya munthu oti akudwala khansa ngati ndalama zilipo atha kukasungisa ndalama ku banki chifukwa patha kupezeka mapeshenti ena ake mwina atha kumakhala ndi mtima oti aaah poti ineyo ndili ndi ndalama izizi ndazipeza kodi ndikasungitsa ndalama ku banki mwina sinditha chaka nkupezeka kuti ndatani ndamwalira nde apono ndi pempho lonena zoti munthu odwala khansa ngati ndalama alinazo atha kukasungisa ujeni ku banki chifukwa atha kukhala zakazaka anakali ndi moyo akudyelera mwina ndalama zija asazikaikile iyayi kuti ndikasungisa ndalama ku banki azadye ndi anthu ena ake ayi nayeso atha kukhalaso zaka zaka ndalamazo nkuzazidyelera nde asamazikaikile za tsogolo lake

on there i can explain that the banks are for everyone whether a person is suffering from cancer if the money is there they can go and keep there money at the bank because they can be other patients maybe they can be thinking that aaah because i have the money but what if i keep them in the bank maybe i will not finish this year I will be dead so is my plea that when a person is suffering from cancer and they have the money they can keep them in the banks because they can leave for years while they are alive and spending the money they should not underrate themselves that if i can keep the money at the bank other people will be the one to use the money not me, no because they they can also live for years and use the money so they should not be worried about there future

I: Ena tingafotokozere bwanji anthu zokhuzana ndi matenda akhansa ndi zithunzi zili apozo

Others how can we explain to people about cancer and the things that are there

08/01: Aaah pamenepaja mfundo imene ndingaikepo ndiyonena kuti munthu ngati ukudwala matenda akhansa ndipo wafika poti walandira chithandizo kapena wauzidwa kuti iweyo uli ndi matenda ankhansa mphamvu ulinazo usaziyang'anire pansi tikati kuziyang'anira pansi ndikungoyembekezera chithandizo kuchokera kwa anthu ena uyenera kukhala ndi nthawi yothamanga thamanga kugwira tintchito timene umatha kutidziwa ndicholinga choti utukuleso pakhomo pako ukagwira gwira tintchito tako tija ndalamazo ukazipeza uyenelaso kupita kukasunga ku banki ukasunga ku banki potero popita kwa nthawi ndikukhulupilira kuti tikatenga zithunzi zitatatu kumtundako zitatuzo mutha kufika size yokhalaso ndi nyumba ngati zimenezijazo ngati mwiniwakewe sungaziyang'anire pansi chifukwa ambiri timati tikapezeka ndi matenda okhalitsa timazidulira tokha moyo kunena kuti aah ineyo ndili ndi matenda ngati kuli kudya ndalama ndidyeletu chifukwa chakuti ndikafa mwina awa azazitenga adzakhala akukangana ayi ndichilimbikiso choti ndigwire ntchito ndikukhala ndi mtima osunga komanso kusunga kwakoko utha kupezeka kuti uli ndi zinthu zimene sizimayenera kukhala ndi ndani ndi iwe nde uthenga ndiwonena kuti kwa anthu amene angathe kukuona awasiamene alindi matenda ankhansawa sangathe kupanga ichi komataonani akupanga ichi anthu aja azalimbikitsika chifukwa cha iweyo ndipo azatha kubwera kuzafunsa nzeru aaah kodi inuyo mumadwala khansa koma khansayo ilipo kulibe kukhalangati ilipo ndichifukwa chani mukugwira ntchito chonchi ndikugwira ntchito chochi chifukwa choti ndinalandira uphungu kuchokera ku tiyanjane ndipo mankhwala ndimakatenga kutiko ku tiyanjaneko modzichepetsa ineso ozichepesa mulungu amathandiza mpakana ndi chifukwa chake mukundiona ndikufika patipo ndikufika size inoyo

aaah on there the point that i can say is that if a person is suffering from cancer and they have recieved help they have been told that you are having cancer when you are still strong you should not hesitate when i say hesitating i mean just waiting for help from other people you need to have time to do the works that you do with the reason that you should enhance the life of your household if you work and when you find the money you are supposed to keep them at the bank that when time passes by i believe that if we get the three pictures up there , those three you can reach the point that you can have houses like those ones if maybe you can not underrate your self because many people when we have diseases that last long we create ends our life that aah i am having this disease if it is using the money i should use them now because if i die these ones will get the money they will be quarreling no if you can have the courage to work and keep the money and the way you are keeping you can find yourself that you are having a lot of things that were not suppose to be with you now the message is that to the people who can see you they will say is this not the one with cancer they can not do this and those people will have courage because of you and they can come and ask you the ideas aaah you are suffering from cancer but is the cancer there or not why is it that you are working like this and you say because i received the counseling from tiyanjane and the medicine i get from tiyanjane with my loyalty God helps those that are loyal that's why i have reached this point

I: Ena mungafotokoze bwanji zokhuzana ndi nkhani yama bizinesi kaya ku banki ndi kukhala ndi matenda akhansa

Others how can you explain about the story of the businesses or for the banks with having cancer

All: (silence)

I: Tiwauza chani anthu ena zokhuzana ndi matenda akhansawa

what are we going to tell other people about cancer disease?

MM: Chete chete sautsa nyama tiyeni

silence does not contribute anything

03/02: Ineyo ndi ziti pamenepopo pempho langa ndila anthu akumabanki kuti iwowo kaya munthu atadwala khansa wapita kumene kujaku kukapempha kaya ndi loni asakaikile kumpasa loni ija pomuona kuti mwina ndiwakhansa loni ipite kwa wina aliyense kaya munthu oti ndiodwala khansa kaya si odwala khansa atha kumuthandiza loni ngati munthu uja wakwanilisa zofunika kuti atha kulandira loni asayembekeze kuti aaah mwina tikampasa loni mwina akadzatisiya tidzasowa kuti mwina tizampitisa bwanji ku khothi asatengere zimenezozo amuthandizebe loni bola ngati kukwanitsa wakwanitsa

Here i am going to ask the people from the banks that if the person is suffering from cancer has gone to the bank and is asking for a loan they should not fail to give them a loan for just seeing that they are suffering from cancer the loan should be given to everyone whether a person is suffering from cancer or not they can give them a loan if a person has all the capacities to get a loan they should not wait that aaah maybe if we give them a loan maybe if they die we will have no one to get them to court they should not do that they need to help them with a loan if they have managed everything.

I: Chabwino chabwino ngati palibe choonjezera ku gulu limeneli tikupita ku gulu limene lili apali. wina angotifotokozerako zokhuzana ndi gulu limeneli

Alright alright if there is nothing to add on this group we are going to this group here can someone explain about this group

06/01: Gulu limeneli ndi la anthu opemphera anthu amenewa tikhoza kuwaikaso ku gulu loka lolimbikisa chifukwa, galomoto kupanga phokoso lalikulu

This is a category for people who praise the Lord. These people we can also put them in a category of people who encourages…(interruptions)

I: Pepani chasokosa sindinamve

Sorry there was a lot of noise i didn't get you

06/01: Gulu limeneli ndi la anthu opemphera anthu amene timatha kupeza tinene kuti kuchosa nkhawa tikakhala mmagulu amenewa timatha kumalimbikisika pa moyo wa uzimu mmeneso ungakhalire komanso umatha ukakhala mmagulu ngati amenewa umatha kuvomelezeka chimene uli chifukwa choti chimachitika ndi choti ngati siupezeka mmagulu awa iweyo wapezeka ndi khansa upeza zoti ukusaka ndalama movutikila ulendonso kwa asing'anga asing'anga nde zimene atachite iwo akuchekela chekela mabala ajaso ayamba kubwera madzi mapeto ake tikupezaso vuto lalikulu kucha kunthenda imene tili nayo koma ukapeza gulu ngati limenelijalo ku nthenda ya khansa zimakuchitilanso bwino chifukwa anthu ngati amene aja sangakuuze kuti bwera tikuchekere iyayi iwo aja zimene angakupange akulimbikitsa ndikukupemphelera iwenso ndikumamva kukoma mmoyo nde gulu lake ndilimenelo

This is a category for people who praise the Lord, these people we can say we find or let me say that they help to get us not to be worried. When we are in groups we encourage each other with christian life the way you can be living. You can also be in groups like these ones you accepts what you are because what happens is that if you are not in these groups and you have been found with cancer you will find that you are looking for money in a difficult way and you go to the witch doctor and the witch doctor will cut you and have wounds that can make you have big problems on the disease that you are found to have but if you find a group like this one on the cancer disease it happens good for you because people like these ones can not tell you to come and they cut you, no what they can tell you is to encourage and pray for you and you can be feeling good in life with this category of people

I: Kaya ena

Maybe other ones

03/02: Anthu awowo ndi anthu amulungu amene amalimbikitsa ndikukupemphelera munthu eya kuti aah inuyo pitilizani kumapemphera kuti mwina mukhale ndi moyo wautali komanso pempho langa linanso ndilonena zokuti palinso amipingo ena amatha mwina kuti apeshenti tabwerani tikupempheleleni koma tisanakupempheleleni tayani kaye mankhwalawo chifukwa choti inuyo tikakupemphelelani ndekuti muchila mu dzina lamulungu opanda mankhwalawa nde penapake mapemphero ndi ofunika komanso kuchipatalanso mankhwala ndiofunikaso ziziyendera limmodzi tisanamizike mwina ndi magulu ena ake masiku ano amangobwera timamva mu wailesi kuti amangidwa amipingo ena ake amawauza magadiyani kuti iyayi munthu wanuyu kuti mwina timupemphelele koma asamamweso mankhwalawa kuchipatala asamapite zimenezi tisazigwilitse nchito nde tizipitabe kwa amipingo eni eni osati amipingo amasiku ano oti akumawauza anthu odwala zina zake zosokoneza kuti asiye kumapita kuchipatala kukatenga mankhwala

Those people are people of God the ones that encourages and pray for a person, yes that aah you are suppose to continue praying so that maybe you should have a long life. And what other thing that i can ask is that there are also other churches they maybe tell the patient that come here so that we should pray for you but before we pray you should stop taking the drugs because if we pray for you you are going to be healed in the name of Jesus without medicine so somehow the prayers are important but also at the hospital the medicine is important they need to be going together we should not be fooled maybe with these groups of today they just come we hear them from the radios that people from churches have been arrested they tell guardians that if you want us to pray for your person they should stop taking the medicine, they should stop going to the hospital we should not be listening to these so we should be going to the real churches, those of nowadays are telling people things that can ruin them that they should stop going to the hospital.

I: Nanga ndikuonaposo pali maluwa kaya chithunzi chamitambo mungafotokoze bwanji za zithunzi zimenezi

Now i am seeing that there are flowers a picture of the clouds how can you explain about these pictures?

06/01: Izo zofunika munthu amene anajambulayo akhoza kudziwa thandauzo lake

That one needs a person who took the picture so that they can know what it means.

08/01: Aah ndingopanga mwachidule ngakhale ndisanajambule ine ineyo kuona kwanga ndi mbali imodzi yakuuzimu chifukwa pamenepo ndikukhulupilira kunena kuti ndikulemekeza chilengedwe chamulungu analenga kumwamba ndikulenga zomera popeza mutnhu sangathe kujambula zonse nthawi imodzi kungonenapo kuti kulemekeza chilengedwe chandani kodi chamulungu pazinthu zonse zimene amatichitila pa umoyo wathu kuti tikapita ku tchalitchi zimene timauzidwa zokhuza ziti zimenezo ukulu wa mulungu onse kuonekelatu pazinthu ngati zimenezo komanso tipitilizeso funso linalo aah ndimbali imozi yoti ife odwala matenda akhansa tinapezeka ndi vuto limenelo chonde tisadzipatule apapa palivuto lina limene timazipatula tokha posapezeka mmagulu mwa anzathu pofuna kumva ma uthenga osiyana siyana uthenga umodzi ndi uthenga ovuta ukapezeka ku tchalitchi chifukwa palibe mpingo umene ungathe kumuuza kunena kuti mukudwala khansa bwelerani musalowe mu tchalitchimu kulibe ndipo sindinamveko nde tiyenera kukhala pafupi ndi mulungu pofuna kukonza moyo wa uzimu chifukwa kukhala ndi matenda akhansa sizitanthauza kuti anthu amene muli ndi akhansa simubwera ku ufumu wanga ayi tiyenera tikakumane ndi anzathu kumakhala ma uthenga osiyana siyana tikuona anthu amene aja akulalika ndiponso akuphunzisa zitha kutheka amene akuphunzisa ali ndi vuto lomwelo la khansalo koma akuwaphunzitsa anzawo kuwalimbikisa njira chifukwa chilimbikiso chimenecho zimakathandiza kuti tikafike pa stage ngati yosangalala kukafika pa stage iyoyo yosangalasa abale ndi alongo mwinaso kukachita zinthu zopambana kumposa munthu amene alibe vuto litilo vuto ili tikatero tikakaona chilengedwe chake cha mulungu nde tizikangodalitsika ndikuonaso kuti mwa mulungumo mauthenga amene tingamve tikukakhala namba wani kukapulumuka mwinaso kukhala ndi moyo opitilira kuposa atnhu amene alichani kodi alibwino bwino chifukwa chachilimbikiso chopita ku tchalitchi kukhala mumagulu osiyana siyana kukaphunzira zinthu zosiyana siyana

aaah let me just do it though am not the one who took this picture what i am thinking is that it's one thing of the spiritual things because there i believe that it's about respecting God's creation who made the heaven and the vegetation but because a person can not take a picture of everything at once so am just saying that respecting the creation of God on everything that he does for us in our life so that when we go to church what we are being told about these things but the greatness of God is being shown on things like these ones and also let me continue on that question aah it's one thing that we people suffering from cancer we were found with this problem please let us not discriminate ourselves here there is another problem that we discriminate our selves from our friends groups when you want to hear different messages to get one message is difficult to hear from church because there is no church that can tell any person suffering from cancer that you should not enter in this church, there is non and i have never heard so we need to be near God if we want to make our spiritual life because having cancer does not mean that those people who has cancer will not inherit the kingdom of God we are supposed to meet with our friends. There are different messages that are found we can see those people preaching and teaching it can happen that they are teaching while they have the same problem of cancer but they are teaching and encouraging there friends because this courage helps them so that they should reach at this stage like being happy. To reach this stage of making brothers and sisters happy and maybe doing greater things than a person who doesn't have this problem now if we see the creation of God then we will be blessed and see that the messages we can hear in Jesus can be on number one is salvation and also maybe staying alive more than those who are okay because of the courage for going to church and to be in different groups and learn different things

I: kwa ena ndi uthenga wanji umene mukupeleka kwa anthu okhuzana ndi gulu limeneli lakutchalitchi

To others what message are you giving to people about this group from church

02/01: kutchalitchi nkofuna kumapita chifukwa ngati munthu sanga ngati osapemphera ndekuti munthuyo wataika ngakhale matendawo okuti chifukwa mankhwalawo amathandizana ndi mulungu mulungu akaika dzanja akaona kuti munthuyu mwakuti amakupangira chisoni mankhwala aja amagwira ntchito koma ukaziika kuti aaah poti ndikumwa mankhwala ndekuti mulungu ndimutaye ndekuti chilimbikiso chamulungu ndekut tikuchitaya

It is important to be going to church because if a person does not pray that means that person is lost even the disease because the medicine needs to be taken together with praying to God if God puts a hand and see that this person this and that, He has mercy on you, and the medicine works properly but if you make yourself that aah because am taking the medicine and you leave God, it means we are throwing away the courage of God.

I: Chabwino chabwino ena

alright alright another one

07/01: Pamenepopo tikuona kuti ngakhale tikudwala khansa komanso ndikumapita ku tchalitchi ndikumakapemphera kwa mulungu amakupasaso madaliso chifukwa munthu ongokhala osapita ku tchalitchi sungalandile madalitso koma madaliso aakulu timalandila ukamapita ku tchalitchi kukaonana ndi abale ako nde mulungu amakhoza kumakupasa madaliso ambiri

There we can see that even though we are suffering from cancer and also going to church and praising to God he gives you blessings because people who doesn't go to church can not get blessings but we get a lot of blessings because of going to church and seeing your friends so God can give you a lot of blessings

I: Chabwino

Alright

08/01: Ndingoonjezera pang'ono amene tili munofe sikuti ku tiyanjane timabwera tokha iyayi ndikhulupilira kuti anthu amene ali kumeneko ali ndi ma list ambiri mbiri a anthu ambiri mbiri ndichisomo chamulungu kutengapo maina athu ndikutipezeketsa kuno chifukwa chakupemphera kwathu zapangisa kuwanong'oneza iwowaja kuti inu tatiyeni tatiyeni nde tiyenekera kumulemekeza mulungu

let me just add alito bit those of us who re in here it's not only us who comes to tiyanjane i believe there is a list of alot of people it''s only by the grace of God to pick our names and make us found here because of our praying it has made to tell them that let's go so we need to give respect to God

I: Palinso uthenga wina umene tingapereke kwa anthu okhuzana ndi kukhala ndi matenda akhansa ndi zithunzi zimenezi za mulungu zokhuzana ndi kutchalitchi

Is there any message that we can give to people about suffering from cancer and these pictures about God and the church

09/02: Ndithe kuonjezerapo eti ichichi ndi chidandaulo cha anthu amene akudwala matenda a khansa nde chidandaulo chawo chikukhuzana kunena kuti chikufuna mankhwala achithandizo kuti dziko lonse liime pamodzi ndi a tiyanjane nde ndaona kuti nkhani ikutani ukulowa pati pamenepo tingoonjezerapo motero

let me add right this one is my plea that people who are suffering from cancer there worry is about getting medicine help so the whole country should stand together with the tiyanjane clinic so i have seen that the issue is about that so thats my addition.

06/01: Komanso mipingoyo ikhoza kutengaso gawo anthu omwewo opemphera mu mipingomo tikhoza kutengaso gawo powathandizila awawa anthu odwala zikhoza kutheka mpingo kutengaso gawo lothandiza kuti anthu amene akudwalawo nawonso asasalidwe

But also the churches can play a role, the same people who prays in churches we can can play a role in helping these people who are sick it can happen that a church can take part in helping those people who are sick so that they should not be discriminated

I: Pamenepo tafotokozani

Can you explain on that

06/01: Mpingo wina uliwonse kwa munthu amene akudwala umatha kutengapo gawo munthu uja kumakamupemphelera kumakamuyendera zinthu ngati zimenezijaso zimapangisa kuti iweyo ngati uli ndi chikhulupiliro bwino bwino komanso okupemphelera uja akamakupemphelera iyeyo ngati ali zeni zeni umatha muthu kumasulidwa kuchizidwa takhala tikumva mmawailesi mmavidiyo ineyo ndinadwala khansa ndinapita kwakuti anandipemphelera ndinachira anthu ngati amaenewaso atatenga gawo ngati limeneli zikhoza kukhalaso zofunikira ku miyoyo yathu

To a person who is sick any church can play a role of praying for the person visiting them and those things makes you, if you have faith but also the one who is praying for you if they are truthful it happens that you can be healed. We have been hearing from radios, in televisions that i was suffering from cancer i went somewhere they prayed for me and am healed people like these ones should also take part like this it can be very important in our lives.

I: Chabwino

Alright

05/01: Apapaso ineyo ndingowonjezeraso pang'ono kuti chikhulupiliro ndiwekha mwini wake chifukwa munthu wina akhoza kubwera kuzakupemphelera koma ngati iweyo ulibe chikhulupiliro sizingatheke kuti ungachilitsidwe koma iweyo uyikepo chikhulupiliro anthu aja akamabwera azionjezera pamanepaja ndiye mulungu mwini wake ujaso naye akamabwera azangopitiliza madaliso aja ndimachiliso aja kupita pati kupita pamwamba koma ngati iweyo siukukhulupilkira kunena kuti ineyo ndingachire kapena kuzikaikira ndekuti olo anthu onse aja angabwere zonsezija zikhala zopanda ntchito chifukwa iweyo chikhulupiliro ulibe koma iweyo mwini wako ukhale ota ukhale okhulupilira kuti aah zingavute zingatani ine ndiochilisika inde nthendayi ilipo koma ndine otani ndine ochilisidwa ndekuti mapemphelo aja akamabwera azingosanjikiza naye ambuye yesu uja akamabwera azangopitiliza kutani kukumasulano iwe kukhala kuti uli kuti uli pa levulo yambwino nde apapa chachikulu anthufe tizingokhala anthu okhulupilira olo tikukumana ndi ululu olo tikukumana ndi matenda akhansa inde a tiyanjane akutithandiza akutiunikira njira izi ndi izi chifukwa a tiyanjane nde amatithandizadi komanso ifeyo ayini wathu ujeni mtima wathu tiusegule timasuke ndithu chifukwa tikapanda ifeyo kumasuka kusegula moyo wathu zonsezi olo tingapange zikhala zopanda pake koma ifeyo tikasegula mtima wathu kulumikizana nawo komanso ifeyo kumupempha mulungu ndi mtima onse china chilichonse ndichotani ndichotheka

here let me add a little bit that faith is in yourself because any person can come to pray for you but if you don't have faith healing can not happen but if you have faith those people if they come they will just add from there now God himself when coming He will just continue with the blessings and the healing going up but if you don't believe saying that i can be healed or doubting for yourself it means that if those people can come all the things will be useless because you don't have faith but you should have faith for yourself that aah whatever happens i am to be healed yes the disease is there but am healed then the prayers when coming they will be filled with Jesus Christ and will continue to help you and you will be on a good level now here the most thing is that we should be people with faith even if we are having a lot of pain or suffering from cancer. Yes people from tiyanjane are helping us they are showing us the right ways because these tiyanjane people they really help us but also we should open our heart because if we doesn't open our heart everything here even if we can do they will be useless but if we open our heart and ask God with all our heart everything is possible.

I: Chabwino

Alright

05/01: Eee

Yes

I: Titha kupita ku gulu lina kapena pali ali ndi choonjezera

can we go to another group or is there anyone with something to add

(silence)

I: Gulu lomalizali tatifotokozeleni zomwe zikuchitika pamenepo ndi gulu lotani

on the last category can you explain on what is happening there and what category is this?

09/01:gulu lomalizalo munthu odwala khansa tiyenera kumuchapira mkumuphikira ndiponso chithunzi ichocho cha nambala folocho ndikumuyalira pa bedi ndi bulangete kuti azikhala bwino azigona bwino

on the last category, we are supposed to wash and cook for the person who is suffering from cancer and also on that picture number four making on the bed with the blanket so that they should be sitting down and sleeping properly

I: Nde ndi uthenga wanji umene tingapeleke pa tsikuli okhuzana ndi kukhala ndi matenda akhansa pa zithunzi zimenezi

Now what message can we give on this day about cancer on these pictures

04/02: kwa amene tikudwala matenda akhansa tiyenera kuwasamalira kuwachapira kuoneka kuti azioneka amoyo wabwino osintha ndithu usakhale opanda ukhondo chifukwa ukhondoso matenda ukakhala osachapa umakhalaso munthu matenda amakulira kulira chifukwaso chaumve chachani cha munthu amene mukusamalira matenda koma ngati mukumuchapira mukumupangira china chilichonse munthu uja amaoneka bwino moyo wake umaoneka opitilira kuti awatu olo akudwala koma apapa china chili chonse akuchitilidwa kuthandauza kuti kwa mtundu wa amalawi ndi ife tonse amene tikupanga odwala tikudwalisa amatenda akhansa tiyenera kuti tiwasamalire powachapira powasambisa ndipowapanga china chilichonse kuwaonesera ukilini osakhala kuti akhale a umveyi

To those of us suffering from cancer we need to take care of them, washing for them so that they should be looking smart with a change they should not be unclean because cleanliness and the disease when you have not washed clothes the sickness go on because of uncleanliness of the person that you taking care off but if you are washing and do everything this person looks smart and there life looks brighter that this one even that they ae sick but here they are doing everything for them meaning that to all malawians and we all who are caring the patients suffering from cancer we need to take cae of them by washing for them bathing them and on doing everything for them and showing them clean so that they shuold not be unclean

05/02: komanso china ndichakuti ngati munthu odwala khansa wapeza akupezako bwino ayenera kuzichapira yekha olo kuphika olo kuyeselera kutunga madzi zonsezoso zitha kukhala zabwino

And the other thing is that if a person suffering from cancer is getting well they are supposes to be washing for themselves or cooking or trying to draw water all of that, that can be alright

I: Ena

Any other

09/01: Tionjezelepo pamenepo munthu odwala khansa pachithunzi chanamba fayificho ndithokoze ndinaonda kwambiri nde ndimagona pa mkeka nde atiyanjane ndikudwala kwanga kwa khansa anaganiza kuti munthu ameneyu timpase chani nde anandipasa matress amene mukuona kumenekowo ndi blangete amene mukuona pamenepo chithunzi chake ndi ichi nde zinthu zimenezi ndinalandira ku tiyanjane

let me add on that a person suffering from cancer on the fifth picture let me thank a lot, i became very thin and was sleeping on a mat then people from tiyanjane saw my suffering from cancer and they thought that what can we give him they gave me a mattress those that you can see there and the blanket that you can see there this is the picture and those things are the ones that i received from tiyanjane.

I: Nde ndiuthenga wanji umene mungawapatse anthu wakumva zokhuzana ndi gulu limenelili, apa mwathokoza ku tiyanjane komano ndi uthenga wanji mungapeleke kwa anthu.

So what message can you give to people who are listening about this category, here you are thanking tiyanjane clinic but what message can you give to people?

06/01: Gulu limenelo likuonesa kuti pali ena ophika kuchapa mbale chani zovala kutunga madzi zikhoza kuthekanso kuti ngati zinthu zimenezi zingasowe kwa munthu odwala zikhoza kukhalaso vuto ku thupi lake ngakhale atamalandila mankhwala kutani koma ngati akusowa chakudya nthupi chabwino chikhoza kukhala kuti palibe nde ngati kwa anthu amene ayandikana ndi anthu ngati amenewa akhoza kumayesesa kumawathandiza anthu ngati amenewa choti adye apeze chani mphamvu amwe mankhwala ndizotheka kuti munthu akadya amakhala ndi mphamvu mankhwala aja kumwa samakupangisaso vuto lina lililonse chifukwa kulipotu munthu kumwa mankhwala osadya siumaonaso bwino zimachitikaso zinaso mthupi mwinaso matenda enaso mthupimu nde zikuyenera kuti chokudya chizipezeka kuwathandiza ndithu anthu amenewa kuti chakudya chizitani chizipezeka uthenga wanga ukhoza kukhala umenewu

This group is showing others cooking , cleaning plates, washing clothes drawing water it can also happen that if these things will not be available to this person who is sick they can be a problem in there body even if they will be receiving medicine but if they are missing food in there body okay maybe it can not be there so to those people who are close to these people they can be trying to help these people with what to eat so that they have power to drink the medicine it happens that when a person eat's they become strong when you drink the medicine they don't have effects because it can happen that a person can drink medicine without eating you can't feel okay. Some other things happens in the body they can cause other complications in the body so the food should be available to be helping these people so the food should be there this is my message

04/02: Aponso pa ujeni popasidwa a tiyanjane kupasa odwala matilesi blangete lofunda tinganene ku mtundu wa amalawi kuuza a tiyanjane kuti zimenezozo azipitilize asasiile panjira enawonso aziwapasa kuti azitha kufunda chabwino kugonera pabwino chifukwa tikatero ndekuti munthu odwalayo amuteteza mmalo moti mwina anakadwala matenda ena koma apa amupasa chabwino chofunda ndipogonera pooneka pabwino

there also about tiyanjane giving a patient mattress, blanket we can tell Malawians and telling the people from tiyanjane that they should continue to give others so that they should have good bedding's and sleep on a nice place because if they do that it mean s that the sick person has been protected instead maybe they could be sick from other diseases but they have been given good bedding's and a nice place to sleep

I: Choonjezera china

Any other thing to add?

I: kapena pomaliza ndiuthenga wanji umene munganene ku mtundu wa amalawi zokhuzana ndi kukhala ndi matenda akhansa ndi zithunzi zilli apazi

Maybe lastly what message can you say to malawians about suffering from cancer and the pictures that are here?

06/01: Chimene ndinganene pachithunzipa ku mtundu wa amalawi ndichokuti mmene ifeyo tajambulira apo ndimmene anthu angaonele akuyenelaso anthu kutengapo chani kutengapo gawo mabungwewa pankhani ya mankhwala ndimmene zilili pamenepa kuti athuwa apitilize kusangalala

What i can say to malawians on this picture is that the way we have taken those picture it's when people can see so the people, the organisations are also supposed to take part on the issue of medicine with the way things are here so that these people should continue to be happy.

I: Ena mawu anu ndiotani tiwauza chani anthu zokhuzana ndikukhala ndi matenda akhansa

others what can we say to the people about suffering from cancer?

08/01: Aah chimene tingawauze anthu uthenga kupezeka ndi matenda akhansa wani simathero a moyo iyayi sikuti munthu wawola iyayi tintenge munthu ngati mmene amakhalira munthu wina liyense ndipo munthu akapezeka ndi matenda amenewa akalandira chisamaliro kumene akulandira mankhwala ndikukondedwa ndi wina aliyense zizapangisa kunena kuti munthu akathe kupeza bwino ngakhaleso kuchira kumene zimatheka kunena kuti ngakhale dziko limatha kunena kuti matendawa alibe mankhwala koma chifukwa chachisamaliro ndikuyendesa bwino kwa odwalayo pakamwedwe ka mankhwala ndichilimbikiso zitha kuthandiza kuti munthu uja akathe kuchira chifukwatu pali ena amene akuchira kupezeka kuti nthawi zina kuchipatalaku nthawi yina amawauza kuti yambani mwaima kaye mankhwala chimene chikuwapangisa kuti imani kaye mankhwala ndi chani munthu uja wayendesa dongosolo podziwa kuti iyeyo ndi munthu komanso amene akumuyang'anira akudziwa kuti uyuyu ndi munthu ndikumuthandizira kumusamalira osamusala chifukwa chazimenezijazi akalandira mankhwala aja atha kukachilitsidwa tikapita ku nkhani ya achibale onse achibale akhale naye pamodzi osasiilanso munthu mmozi popeza ndi mkazi wake kapena mamuna wake kapena uyo ndindani ayi kuti timusiila yemweyo ndi amene azitha kuyang'anira matenda aja ayi tipite tikamuchezere ngati chithandizo tikamuthandize ngati alindi tilindi ntchito yoti timupase atigwilire kuti timpase kenakake tiyenera kumpatsa komaso ngakhale mma ofesimu amene amatha kulemba ntchito asayang'ane za nthendayi koma ayang'ane za ntchito imene iye amaitani kodi amaidziwa ampatse ntchito kuti akagwira ntchito ija akathe kupeza chithandizo kunyumba komanso ku mpingo ndi uthenga oti ampingo nawonso ayenera kumusamalira ndipo kumuona ngati munthu timaudindo tonse timene timafunika ku tchalitchi kapena ntchito zonse zimene zimafunika mu mpingo athe kugwira nawo chifukwa aliyense ali ndi mphatso imene mulungu anamupatsa tikapita kumbali ya kugwira gwira ntchito pali ena amangonena kuti popeza munthuyu ndi odwala akati agwile ichi iyayi usagwire ntchito zimenezijazo zisatero apatsidwe ntchito ndipo agwire ntchito chifukwa mukugwira gwira ntchito mwake thupi limatakasuka zonsezo zikamangilira pamodzi zikamangilira pamodzi zikawakweza anthu aku tiyanjane ndi anthu amene anapanga chinthu chimenechijacho ndi amene anapanga mankhwalawo ndi amene amapeleka chithandizo cha mankhwalawo ku tiyanjaneko kuti azitipasila ife anthu zikawapasa chilimbikiso ngakhaleso kuonjezera zina mwaizo kudzera mu nkhani imene taika apayi

Aaah the message that we can give to people is that having cancer is not the end of life no it's not that the person is rotten, no we should them just as any other person and if a person is found with this disease if care is received from where they are receiving medicine and being loved with anybody it will make a person to become better even to be healed it happens that even the country says that this disease has no cure but because of the care and following the procedures for taking medicine and the courage it helps a person to be cured because there are others who are healed and it happens that sometimes at the hospital they are told to stop taking the medicine what is making them say stop taking the medicine? That person has followed the procedures. Taking in mind that this is a human being and also the one taking care of them and not discriminating them and because of that and also after they receive medicine they can be cured. If we go to the issue of relatives every relative should be together with them they should not be leaving that to one person because it's the wife or husband and you are someone else, so leaving that one to be taking care of the patient no. We should be chatting with them we should help them and if we have something to give them like to work for us so that they can earn money let's give them even in the offices those who employs should not say this person is suffering from cancer but they should see what job does this person knows and give them to work so that they can get something to help the family even also in churches this is a message that they should also take care of them and look at them as people so that they can also give them the church positions or the jobs that are at church they can also work because everyone has a different gift given from God if we go to the side of working others just say that because this person is sick if they want to work they will say don't do this don't do this job it should not be like that they should be given jobs and they should work because the time that they are working their bodies become strong and that makes people from tiyanjane to be proud of and other people who started that thing and those who made the medicine and those that give help for the medicine at tiyanjane so that they should be giving us if it gives them courage even adding other things from what we have been saying here

I: Chabwino palinso ena amene ali ndi mau oonjezera?

Alright is there anyone who has anything to add?

(silence)

I: Ngati palibe tithokoze kwambiri chifukwa cha zithunzi zimene munatenga ndikufotokoza mmene mwatifotokozera zikomo kwambiri ayi tithele pamenepa pa zokambirana zathuzi tathokoza kwambiri tiyeni tiombe mmanja

if there is nothing else then we should thank you very much because of the pictures you took and explained the way you have explained. Thank you very much, so we should finish our discussions here we are very thankful, let's clap hands.

All: (clapping hands)

(clapping hands)

FGD5

zoyankhula kupatul mwina mayi anaga ali apowo chifukwa chokuti samatha kuona bwinobwino koma enafe...Ndiye tiyambepo eti. Ndiye lero zokambirana zathu kwambiri tufuna tidutse monse mmene takhala tikudutsa muja chimene tikutanthauza nchoti tufuna tione mafunso onse atatuwo ngati pali pena pamene mwina sitinathe kunena lerolinso tapatsidwa mpata oti tikhonza kufotokoza eti ndiye zithunzi ndi mmene mwaziikiramu mmagulu osiyanasiyana zimene zili ndi nkhani yosiyana siyana koma tionenso mafunso atatu aja. Zithunzi zimene zili patsogolo zathunzi tikufuna kuona kuti kodi ndi ziti zimene tikufuna kuwauza anthu ena kuti adziwe zokhuzana ndi chisamaliro cha ku Tiyanjane ndi matenda a Khansa kapena apo mukufuna mutawafotokozera zotani anthu kuti kodi kukhala ndi matenda a Khansa zimatanthauza chani kapena apo ndi ziti zimene tikufuna kuwauza anthu ena kuti adziwe zokhuzana ndi kukhala ndi matenda a Khansa. Tanena mafunso onse atatu pamenepopo kuti titsakhale ngati ta, tapatsidwa mbali imodzi yokha. Koma leroli pokhala zokambirana zomaliza tikudziwa kuti mwina tikudziwa kuti ndi zokambirana zomaliza eti tikufuna kuti ti. ti ti, pasakhale mbali imene munganene kuti aaa koma mbali ijayi mnaiwala chinachake choti ndiyankhule nde nthawi yake ndiimeneyi. Tipempha kuti wina aliyense ndithu akhale omasuka tikambirane bwinobwino timve kuti kodi nkhani imene leroli tabwertsa kudutsanso mmafunso atatu muja ndinkhani yotani. Aliyense ali omasuka kuyamba, kuyamba kufotokoza gulu loyambiriralo kuti limenelolo likutanthauza chani. Ndinkhani ya chisamaliro cha ku Tiyanjane ndi matenda a Khansa mwina zikugwirizana bwanji eti?

We should have all gather around here so that we should be able to see these pictures so that we should all take part on the discussion except maybe that my mum because she is not able to see clearly but the rest of us…So we should start right. So mostly on our discussions today we want to pass through where we have already passed what I mean is that we want to see all the 3 questions if maybe there is somewhere where we did not talk about so we have been given that chance today that we can explain right so the pictures, like the way you have placed them in different groups have different stories but we should also tackle those 3 questions. The pictures which are in front of us, on the pictures we would want to see what are the things that we would want to tell other people so that they should know about the care from Tiyanjane and the cancer disease or what would you want to tell people about what it means to have cancer disease or what are the things that we want to tell other people to know about having cancer disease. We have talked about the 3 questions there so that it should be like, we have been given one side only. But today being the last discussion we know that maybe, we know that this is the last discussions right we want to, to, to, there should not be any side where you can say that aa but I forgot to say something that side so this is the time. So we ask that everyone should be free so that we can discuss well and we should hear what stories have we brought today tackling the 3 questions, what story is it. Everyone is free to start, starting with explaining what the first group means. What is the connection to the issue of Tiyanjane palliative care and the cancer disease right?

P: Mmm

Mmm

I: Chomcho mpaka tifika kumapeto. Amene ayambe, tiyeni tiyambe aliyense akhoza kuyamba. Musavutike kwambiri kuona mafunso, mafunsowo muli nawo kale mmutumo mnangofuna kuti tiunikire kuti tufuna tione mbali zonse, timalizitse mbali zonse.

Like that and we will reach to the end. The one who will start, let’s start anyone can start. Don’t bother a lot on looking at the questions, you already have the questions in your mind I just wanted to show that we want to look at both sides, we should finish with all the sides.

I2: Mutha kufotokoza mbali imene inuyo mukuona kuti mungafotokoze bwanji.

You can talk about the side that which you feel that you can explain in what way.

I: Ehe kaya ndi mbali imodzi.

Yes, either one side only.

08/01: Ine ndiyambapo, ndiyankha funso layambiriralo ndiziti zimene tikufuna kuwauza anthu ena kuti adziwe zokhuzana ndi chisamaliro cha Tiyanjane ndi matenda a khansa. Ndiyamba ndi matenda a khansa. Matenda a khansa ndi nthenda imene imatha kufika pathupi lamunthu monga nthenda inailiyonse ndipo nthendayi imasonyeza kuti ilibe mankhwala koma sichinthu chodetsa nkhawa chokuti kunena kuti ukathe kuda nacho nkhawayi chifukwa chani chokuti ukade nacho nkhawa? Pali amene a Tiyanjane amene adachikonza kuti iwow azitha kuthandiza anthu akhansa chifukwa cha chithandizo cha khansacho apa zikufotokoza kunena kuti munthu amene wapezeka ndi matenda a khansawo asakhale okhumudwa ndikudandaula. Chifukwa chani, pali awa aTiyanjane palliative care amene amasamalira zamatenda a khansa pamene paja tikulandira chithandizo, chisamaliro chonse chamatenda a khansa ndi uphungu omwe oti tikathe kulumikizana pakati pamatenda ndi chasamaliro cha Tiyanjane ndipo munthu amene tikumufotokozera tikamufotokozere molimba mtima kumutsimikizira kunena kuti asachite mantha atapezeka ndi matenda a Khansa chifukwa ndi nthenda inailiyonse imene monga munthu wamoyo angathe kukumana nayo.

I will start, I will answer the first question, what are the things that we want to tell other people to know about the Tiyanjane Palliative care and the cancer disease. I will start with the cancer disease. The cancer disease is the disease that gets to the humans body like any other disease and the disease shows that it does not have medication but this isn’t a thing to give you stress, a thing to stress you up, why should you be worried? There are people, the Tiyanjane’s who designed it that they should be assisting the people with cancer because of the cancer assistance, here it explains saying that the person who have been diagnosed with cancer should not be worried and disappointed. Why, there are the Tiyanjane Palliative care who care for the cancer disease, when we are receiving the care, all the cancer care and counseling for us to be able to connect between the disease and the Tiyanjane care and the person whom we are explaining to we should explain to him/her with confidence that if found with cancer she/he should not be afraid because its any other disease that a human living being can encounter.

I: Chabwino, ena amene akufuna kuonjezera.

Alright, any other who wants to add.

07/01: Kunena kuti pamenepopo nthenda yakhansayi ndi thenda yo, yaikulu kwambiri. Poyamba munthu akadwala khansa…

Saying that the cancer disease is a kind of disease, a very huge one. At first once one has cancer…

I: Mmm

Mmm

07/01: Amakhonza kumu, kuchotsa ngati mwendo kapena nkono...(Overlaps)

They happen to, to cut off his leg or arm…(Overlaps)

I: Tipempha kuti mukwezekobe mau eti.

We ask that you should raise your right.

07/01: Eee

Yes.

I: Mmm, pitirizani bambo.

Mmm, continue dad.

07/01: Poyamba munthu akadwala khansa kaya watupa mwendo.

At first when a person is sick from cancer either the leg is swollen.

I: Mmm

Mmm

07/01: Amakhonza kuudula mwendo uja kuti mwina mwake apeze thandizo labwino. Koma chifukwa cha ku Tiyanjaneku kuli mankhwala amene amatipatsa kuti munthu uja asachotsedwe nkono kapena mwendo ndiye pamenepapa tikuona kunena kuti ku Tiyanjane kuja tima, timayamikirako chifukwa cha, mankhwala amene amapatsa munthu wodwala kuti matenda aja afike kunena kuti akupezako bwino mkati mwa thupi lake. ndiye timathokoza kwambiri a Tiyanjane chifukwa cha matenda a khansa.

They would cut off the leg so that he/she might be assisted. But because of Tiyanjane, they have medicine which they give us so that the person’s leg or arm should not be taken off so here we can see that we are grateful to Tiyanjane because of the medicines which they give the patient so that the disease should reach the point of, feeling better inside his/her body.

I: Chabwino. Enanso amene akufuna kuonjezera. Azimayitu mwangokhala pheee.

Alright. Any other who wants to add also. You ladies are quite.

06/01: Tikudikira uku.

We are waiting for this side.

I: Mukudikira uku...(laughs). Uku kulibe pamene mukufuna muonjezere? Koma mwina nkutheka alipo amene akufuna aonjezere mbali ya uku?

You are waiting for here…(laughs). There is no where here where you want to add? But maybe it can happen that there is someone who wants to add on this side?

02/01: Aa koma ineyo ndikuyamikara koma a Tiyanjane poganiza zo, amene anaganiza zobweretsa ujeni, anthu a Tiyanjanewo kuti abweretse chipatala munomo Mmalawi muno chifukwa poyamba anthu amavutika kwambiri enawa monga mmene amanenera amdalawa kudulidwa mwendo, kapena nkono kapena kumwalira kumene koma tsopano a, a Tiyanjane ndi mmene apangiramu tikuona ngati kuti imfa yambiri kapena adulidwanso sakupezeka kawirikawiri ndithu. Ndiye tikuyamikiranso ndithu kwambiri kuti zimenezo apitirize zikanapitanso ngakhale mmidzi mmene, eya.

Aaa, but myself am grateful for Tiyanjane for having the idea of, the ones who thought of bringing this, the Tiyanjane for bringing the hospital here in Malawi because at first people were struggling a lot, some like this man has said having their leg being cut or the arm or even dying but now with the way Tiyanjane people has done it we see that a lot of deaths or even those who are cut are not found frequently.

I: Chabwino.

Alright.

02/01: Ndithu.

Sure

I: Chabwino. Mwati, Tiyanjane, ikanakhala kuti yafikanso mpaka mmidzi...?

Alright. You said that had it been that Tiyanjane has reached even in the villages…?

02/01: Eee yafalikira mpaka mmidzinso chifukwa mmidzimu ndi mmene anthu akuvutikanso chifukwa chakuti ena sakudziwa kuti Tiyanjane, ujeni mankhwala ake tingaapeze bwanji kapena chiyambi chimakhala bwanji.

Yes, it has spread to the villages because it is in the villages where people are struggling because some people do not know about Tiyanjane, this, “how can we find its medication or how is its beginning.”

I: Chabwino.

Alright.

02/01: Eee

Yes

I: Tathokoza. palinso mwina ena amene akufuna aonjezere?

We are thankful. Is there any other who wants to add also?

06/01: Mmm ndizoonadi a Tiyanjane akutithandiza.

Mmm, its tru the Tiyanjane people are assisting us.

I: Mmm

Mmm

06/01: Kutereko ifeyo popanda gulu ngati limeneli sitikudziwa kuti zikadafika pati chifukwa a Tiyanjane anayesetsa ndithu kuunikira winaaliyense mpakana kupeza chani chithandizo komanso mwina kumakuchepetsa nkhawa, mmm chifukwa munthu umakhala ndi nkhawa malingaliro anzako akamati iii iyitu ndi nthenda yoopsya auje aja anamwalira nayo. Koma ukapita kumene kuja samakuuza zimenezo amakulimbikitsa komanso ndikukupatsa chilimbikitso chokwanira choti iwe ukamachoka mmenemo umakhala ukudziwa kuti ineyo sikuti kupezeka ndi khansa kuti ndifa lero ayi ndikhala ndithu mdimoyo bola nditsatire zimene a Tiyanjane akunena. Komabe paokha a Tiyanjanewa nkuthekadi akhonza kumakhala opelewera muzambiri monga tiyerekeze panopa a Tiyanjane amango, amatipatsa makhwala ongoziziritsa osati othesetseratu ayi. Akanakhalaponso ena owathandizaa Tiyanjanewa kuti apeze mankhwala othesetseratu kuti azichiriratu anthu osatinso kumangoziziritsa ukatere ubwererenso ayi. Akanapezeka anthu ngati amenewo othandiza kuti apezeke mankhwala zidakachitabe bwino kwa anthu amene akubwera kutsogolowa sadakazayenda moyo umene tayenda ifewu. Chifukwa ndiye nthendayi ndiye ndiyowawa ndipo ndi yozunza tikuyenera ndithu kutani, kupeza thandizo ngati limenelo.

It is that without this group we do not know where we could have reached because the Tiyanjane people tried their best to screen averyone up to finding the assistance and also maybe reducing your anxieties, mmm because you do have anxieties, the thoughts when your friends are like, “iii this is a killer disease that person died of it.” But they do not tell you that when you go there they encourage you and gives you enough courage that when you are leaving there you know that although I have been diagnosed with cancer it doesnt mean that I will die today, no I will be alive as long as I follow what the Tiyanjane people are saying. But still the Tiyanjane people on their own it might happen that they lack in so much like for example right now the people from Tiyanjane gives us medication just to ease the pain and not end it. Had it been that there are some others assisting these Titanjane people to find medication that will end or cure people not only easing the pain then it should come back, no. Had it been that people like these were found , assisting to find the medication this would have been good to the generation coming, they wouldn’t have passed where we have passed. Because this disease is very painful and tormenting we are really supposed to what, get that assistance.

I: Chabwino. Palinso ena? Tipite kugulu lina?

Alright. Is there anyother? We should go to another category?

06/01: Mmm

Mmm

I: Chabwino. Tiyeni tsopano pagulu lina lachiwiriri tikambapo nkhani zoti bwanji?

Alright. Lets go to the second category, what stories are we going to talk about?

09/01: Apa…

Here..

I: Mm mmh

Mm mmh

09/01: Ndi chithandizo chimene amapereka a Tiyanjane kwa munthu odwala khansa.

Its the assistance which is given to the patient by Tiyanjane people.

I: Mmm

Mmm

09/01: Choyamba amapereka mankhwala munthu odwala khansayo amampatsa matiresi pulasi soya kuti adzikadya kunyumba pulasi neti oti udzudzu usamamulume.

Firstly, they provide medication, the cancer patient is given mattress plus Soya to eat at home plus net so that should not be beaten by mosquitoes.

I: Mm mmh

Mm mmh

09/01: Mm mmmh, basi zomwezo.

Mm mmh, that is all.

I: Chabwino. Ndiye pankhani imeneyoyo mwina nkutheka mufunanso mutapereka uthenga kapena kwa anthu kuti adziwe pankhani yachisamaliro chimenechocho komanso ndi nkhani yamatenda a khansa.

Alright. So on that issue maybe you would want to say or tell people to know on the issue of that care and the cancer disease.

08/01: Mmm, pankhani imeneyo ndikuwamema anthu. Tikati kuwamema kuwapempha anthu, kuwauza anthu kunena kuti ngati munthu ukhala ndi vuto la khansa nuthamangira ku Tiyanjane nakuuza kunena kuti uli ndi vuto la khansa iwo omwewo ndiamene amatha kukhala ndi zoyenereza zoti akuthandizire ndi uphungu wawo monga mankhwala, zakudya ngakhale pogona ngakhalenso kudziteteza kuudzuzu ndi uphungu oyenera kuti ungathe kukhala ndi moyo nthawi yaitali pokulimbikitsa kamwedwe kamankhwala posa, pokulimbikitsa posakupatsa mantha.

Mmm, on that issue am urging people, when I say urging, asking people telling them that if a person has a cancer problem and run to Tiyanjane and they tell you that you have the cancer problem they are the same people who have the necessities to assist you and appropriate counsel for you to have a long life by encouraging you on how you should be taking the drugs and by not, by encouraging you without frightening you.

I: Mm mmh

Mm mmh

08/01: Ndiye anthu onse kaya amene akudwala kapena sakudwala kapena ndinene kuti amene ali ndi odwala amene akuonetsa zizindikiro zoti iwow ndi matenda akhansa kapena akukaikirapo ayenera kunyamuka kupita ku Tiyanjane kuti akathe kulandira chisamaliro chimenechi.

So all the people either they are sick or are not sick, or I should say that those who have patience showing the signs of cancer disease or they are not sure they are supposed to go to Tiyanjane so that they might be able to receive this care

I: Chabwino. Ena? Pagulu lachiwiriri tingalankhulepo kuti bwanji? Tikumbukire kuti tikulankhula zambiri, tikulankhula nkhani ya ku Tiyanjane ndi matenda akhansa kapena iyayi zimene mufuna kuti anthu adziwe, kuti kodi kukhala ndi matenda amenewa a khansa zikutanthauza chani pamoyo wanu.

Alright. Any other? What can we say on the second category? We should remember that we are talking on a lot, we are talking on the issue of Tiyanjane and cancer disease or else what you would want people to know about what it means to have this disease in your life.

07/01: Mmm, tikuona kuti munthu ukadwala khansa umakhala ndi maganizo ambiri monga tinena kuti ena amatha kumakuuza kuti panali abale awo ena amadwala nthenda imeneyi koma anamwalira ndiye ukamakhala ukumaganiza maganizo ngati amene aja iwenso umakhala ndi nkhawa komanso chifukwa tima, tikunena kuti a Tiyanjanewa, anati, amatipatsa mankhwala ngakhale utadwala bwanji koma ukalandira mankhwala kuchokera ku Tiyanjane kuja umadziwa kuti, thupi lako umalimva, umaona kusintha kuona kuti wayamba kupezako bwino kusiya ndi mmene unaliri, chifukwa chake tikuwathokoza kwambiri a Tiyanjanewa kuti apitirize khalidwe lawo limeneli.

Mmm, we can see that a person has a lot of thoughts when is sick from cancer like we will say that some happen to be telling you that there was their certain relative of them who was sick from this kind of disease and they are dead, so you happen to have anxieties if you are staying while thinking about those thoughts and also because we do, we are saying that these Tiyanjane people did, gives us medicine even if you are seriously ill but when you have received the medicine from Tiyanjane you know that, you feel your body, you see the change, you see that you are better than the way you were that is why we are thanking these Tiyanjane people to continue their behavior.

I: Mm mmh, chabwino. Ena? Azimayi, apatu mwafatsa mwangokhala phe.

Mm mmh, alright. Anyother? Ladies, you have been so quiet here.

05/02: Chifuwa.

Cough

I: Eee?

What?

05/02: Chifuwa.

Cough

I: Ooh chifuwa?

Ooh, cough?

05/02: Mmm

Mmm

I: ooh pepani. Ena amene angaonjezere a (name)

Ooh, am sorry. Any other who can add Mr (name)

09/02: Ine ndingoonjezerapo kumbali yamatenda a khansawo.

I should just add on the issue of cancer disease.

I: Mmm

Mmm.

09/02: Kuti tingowathokoza madokotala amakhala ngati kuti akamabwera amatipatsa mau otani, oti, kutilimbikitsa. Chifukwa pali patient amadandaula kunena kuti inetu matenda amenewa nditani nawo nditha kufa nawo koma madokotala akafika amanena kuti ayi simungathe kupanga zimenezoyi kuti mungafe nawoyi inuyo mudzingopemphera kwa Mulungu ndiyeno tingakhale ifenso timatha kutani kumulimbikitsanso munthu uja monga ngati kwa ifeyo tingowathokoza aku Tiyanjane zimene amachita potiyendera ifeyo kapena kuchipatala zimene amatipanga tingowathokoza poti zimene zimachitika.

We are just thanking the doctors, they happen to visit and give us encouraging words, because where there is, a patient gets worried that I can die of this disease but when the doctors come they tell you that you cannot do that, you cannot die with it you should just be praying to God so even us we happen to encourage the person so to us we are thanking the people from Tiyanjane for what they do by visiting us or what they do to us at the hospital, we are thankful because of what happens.

I: Chabwino. Palinso ena amene akufuna kuonjezera. Pagulu limeneli lachiwiri aliponso ena amene akufuna ayankhule zina kuti aonjezere? Tikhonza kupita gulu lachitatu?

Alright. Is there any other who want to add? Is there any other who wants to talk or add on the second category? We can proceed to the third category?

09/01: Moti ineyo…

For example myself…

I: Mm mmh

Mm mmh.

09/01: Ineyo kupanda a Tiyanjane ndikudwala kwa ine kwa khansa ndimmene mukundionera ndimmene ndakhalira panomu mdakakhala kuti zinthu zimenezi sanandipatse iyayi bwenzi ine palibe komano andipatsa zinthu zake ndi zakudyazo mukuziona pamenepozo. Amadziwa kuti munthuyu akudwala afunikira chani, kudya zinthu zimene zina, zimakhala zosowa kwa munthu. Nde anandipatsa izozo kuti ndizidya ndikazifuna. Umu mmabondomu mnaonda kwambiri ndiye ndimati ndikagona pamkeka mmandipweteka umu mmabondomu anaganiza kuti timpatse chani matiresi. Ndimati kugona pamatiresi ndiye umumu sindimamva kupweteka iyayi ndinaonda kwambiri mankhwala amatipatsa oyenerera oti ndizimwa nde ndimamwadi mwachilungamo.

Myself, without the Tiyanjane, with the way I was sick from cancer and the way you are seeing me sitting here I wouldnt have been here had it been that they did not give me these things but they gave me those things, the food that you can see there. They knew that this person is sick needs what, food and those things which are not available to a person. So they gave me that so that I should be eating whenever I want to. I was very thin in the elbow joint that I was feeling pains in here the elbow joints when I sleep on the mat so they thought that we should give him what, mattress. So when I sleep on the mattress I wasn’t feeling pains here, I got so thin, they were giving me proper medication for me to take and I was really taking them honestly.

I: Chabwino.

Alright.

09/01: Iyo ndi wailesi ili apoyo.

That which is there is a radio.

I: Mmm

Mmm

09/01: Ndimati nkakhalakhala ndimamvera kawailesi kanyimbo, eya poti ndikucheza mwina mwake ndili ndekha mwina mwake a gadiyaniwa ondiyang'anira ineyowo ali panja basi ineyo ndizimvera kanyimbo kangako.

It was that after sometime I would listen to the radio, a song, yes since am chatting or maybe am alone maybe the guardian is outside I should be listening to my song.

I: Mm mmh.

Mm mmh

09/01: Eya

Yes.

I: Ndiye pamene mumamvera nyimbo mumapeza, mwina mumapezapo thandizo lina lotani pomvera nyimbo?

So what assistance were you getting when listening to the song?

09/01: Aaa thandizo limene ndimalipeza ine ndilokuti ndimamva, ndimamva bwino ndimangomvera nyimbo basi.

Aaa, the assistance that I was getting was that I was feeling, feeling good, I was just listening to the music

I: Mmm

Mmm.

09/01: Eee ndimamva bwino, ndimamva kukoma.

Yes, I was feeling good, was feeling sweet.

I: Mm mmh

Mm mmh.

09/01: Eya ndi matiresiwo. Eee ndimamva kukoma kwabasi moti anandipatsanso thumba la Soya ilo lili apolo, likuni phala kuti ineyo ndidzidya ndiye ndimadyadi oti kupanda a Tiyanjane ndi matenda akhansa kundisamamalira kufiks pamenepopo bwenzi aaa ndili kudothi mtamwalira.

Yes, with the matress. Yes, I was feeling very sweet and they also gave me a bag of Soya that one over there, likuni porridge for me to eat so I was eating, its that without the Tiyanjane taking care of me to that point with the cancer disease aaa I would have been in the soil, dead.

I: Chabwino.

Alright.

09/01: Eee

Yes.

I: Chabwino. Ayi nkhani yabwinotu eti?

Alright. Well thats a good story right?

09/02: Mmm

Mmm.

I: Tipite gulu lina, kapena...(Overlaps)

Should we go to another category, or…(overlaps)

09/02: Koma kuchokera pamenepopo pankhani imene akambayo oti mavuto nthawi imeneyo analipodi oti akafuna kudzuka pamphasa kukhalira kumbuyo kumachita kudzutsa mnyamata wamkulu, kumudzutsa kupita kutoileti kukhazika kuvekedwa kabudula.

But from there on the issue that he has explained that there really was problems that time that whenever he wants to stand from the mat, he was being carried at the back always, we would wake him up such a big boy but waking him up, holding him at the toilet, dressing him in his shorts.

I: Mmm

Mmm.

09/02: Kuchokeranso pamenepopo ukali unalipo osakhala masewera kumasoku konseku kunatupa, miyendo yonseyi kutupa koma zimene anachita a Tiyanjane kuchokera pazakudya ndi mankhwala tidaona kunena kuti zinthu zidasintha kobasi. Oti akafika chomchi akaona adokotala kuti tabwerani muone kuwauza kuti ayi zimenezi zitani ziphwa mmisempha. Ndiye tinaonapo kuti chisamaliro cha, chimasamalidwa kwambiri panthawi imeneyoyo koma sindimkazindikira kunena kuti angakhale mmene alili munthuyu ayi ndimati ndikayang'ana ndimangoti aa Mulungu alipo. Chifukwa zonsezo zimachokera kwa anthu a Tiyanjane pondiyendera komanso kuchipatala timakhala nawo limodzi. Potuluka ndithu anatithandiza kutinyamula kubwera kunyumba kudzatisiya ndiye a Tiyanjane zimene anandichitira ine pankhani ya Khansa kudwala kwa iyeyu.

From there also there was much roughness, the whole face was swollen, the legs were swollen also but we saw that things changed a lot because of what Tiyanjane did starting with the food and medicine. That when the doctor is here we have called him saying that, “come and see,” was saying that, “no the swollen muscles will be fine.” Sp we saw that the care, the care was a lot that time but I wouldn’t know that this person can be the way he is now, whenever I look at him I say aaa, “God is there.” Because all that was coming from the Tiyanjane, by visiting me and we were also together at the hospital. They also did help us by bringing us home when we were discharged, so that is what Tiyanjane did to me on the issue of cancer when he was sick.

I: Chabwino mayi.

Alright.

09/02: Eee

Yes

I: Tipitirize, tipite gulu lina eti, Kapena alipo amene ali ndi choonjezera?

Should we continue, we shouold go to the other category right, or there is someone who has something to add?

09/02: Mm mm

Mm mm

I: Chemwali mungoona, pali choonjezera?

My sister you are just watching, is there anything to add?

05/02: Mm mm, ayi.

Mm mm, no.

I: Tipite gulu lina?

Should we go to another category?

05/02: Eee

Yes.

I: Tinali apa ndiye tili apa eti? Nanga ili ndi ili ndi gulu limodzi?

We were here and now we are here right. Is this and this the same group.

Ps: Mmm

Mmm

I: Ndi gulu limodzi, ooh ndiye kuti tili pa, tili apa?

Its one group, ooh so it means we are, we are here?

08/01: Ili ndi lina ili.

This is different.

I: Ili ndilinatu?

Its a different one?

09/01: Ndilina

Its a different one.

03/01: Ili ndi lina.

This is a different one

I: Ndiye...(overlaps)

So…(Overlaps)

08/01: Mmm

Mmm

I: Ndilina eti a (name)

Mr (name), this is a different one right?

08/01: Eee

Yes

I: Chabwino ndiye tiyeni tikambetu tsopano zagulu limene lili pamenepa mukupereka uthenga otani? Kuchokera kugulu limeneli.

Alright, so lets talk about this category here, what message are you giving? From this group.

08/01: Uthenga omwe ukuchokera apa, aaa ndi zotsatira zachisamaliro cha a Tiyanjane. Tikuwafotokozera anthu ndikuwatsimikiziranso anthu uphungu umene umabwera ku Tiyanjane umakhala ukutiphunzitsa chinthu choyamba ngati chithunzi ichi.(1) Tikuona kuti amayi apa akupereka chithandizo akupereka madzi kwa odwala kukhala uku nkuona kwa ineyo ndikuona kuti ngati odwala timkhalira chomchi timaona, tikuonetsa kunena kuti munthu tikumtalikira ndipo tikumutsala tikuopa kuti mwina atipatsira matenda aja koma chifukwa chauphungu waku Tiyanjane wabwera wadzatilimbikitsa kukhala chomchi kumusamalira chomchi , zikupangitsa chomcho kuti akasamalidwa zimene zijazo ndi chithandizo cha uku zukagwirizana, uphungu ochokera ku Tiyanjane chisamaliro chochokera ku Tiyanjane kubwera mwa gadiyani omuthandizira zukapangitsa kunena kuti odwalayo akathe kuoneka ngati mmene alili apa, ndipo odwalayo akathe kukhala olimba mtima nkukapeza kunena kuti, kuperekanso chitsanzo ngati ku ana awa.(7) Ananso kukawayandikira odwala uja osamutha, osamutalikira odwala chifukwa cha uphungu ochokera ku Tiyanjane. Papa, papanda a Tiyanjane bwenzi pano ma gadiyani amene amatiyang'anira ife odwala akumatitalikira koma chifukwa cha uphungu ndi chisamalaliro kupezeka kunena kuti tinadwalika nkudzalandira, kupita kuchipatala nkulandira mankhwala thupi likusintha gadiyaninso atalandira uphungu oti asamalireni odwalawa zikupangitsa kunena kuti wina aliyense adzitikonda.

The message from here aaa are the results from Tiyanjane palliative care. We are truly telling people that the counsel from Tiyanjane does teach us like this picture (1) as a first thing. We can see that this ready is giving assistance, she is giving water to the patient to me I see that if we sit with the patient like this we are showing that we are at a distance and we are afraid that maybe she might infect us with the disease but the counsel from Tiyanjane encourage us to sit like this, if we assist her like this it comes into agreement with the assistance from here, the counsel from Tiyanjane, the assistance from Tiyanjane through the guardian assisting the patient, it makes the patient to be like the way she is here and makes the patient to have trust to find that… and giving also an example like these children (7). The children also getting closer to the patient without, not being far away because of the counsel from Tiyanjane. Here, without Tiyanjane our guardians taking care for us patients would have been far from us but because of the counsel and the care it is found that we got very sick and received…we went to the hospital and received medicine that the body has changed, and while the guardian also received counsel that take care for the patient and this makes everyone to love us.

I: Chabwino. Achimwene anga mwakhala kumpando mwina muli ndi zoonjezera lerolitu mwakhala phe?

Alright. My brother there on the chair maybe you have something to add, you are quiet today?

03/02: Aaa kutsogoloku, titulukira.

Aaa, I will chip in as we go along.

I: owo, kutsogoloku, apapa palibe chimene mufuna muonjezere? Nanga mayi (name) mwina poti bambo ayankhula kale?

Ok, as we go along, it means you don’t have anything to add here? What about mrs (name) maybe because the husband has spoken already?

08/02: Ndizomwezo.

Thats all.

I: Alipo amene akufuna tionjezere pagulu limeneli, zimene mufuna mukambe zoonjezera zokhuzana ndi gulu limeneli?

Is there anyone who want to add on this category, what you would want to say in relation to this category?

09/01: Apo tikutha kuona kuti odwala ena pamene paja akugwira ntchito monga pachithunzithunzi ichi komanso tikuwauza anthu kuti khansa, pachi, pachithunzithunzi icho (PICTURE) ine ndimadwala khansa, panopa ndima, panopa ndimagwira ntchito bwinobwino. Mwendo wanga sunali okuti mpakana kuti ndingavale nsapato iyayi panopa nsapato ndimavala kunena kuti oo ndimapezeka kuti mwendowu watupa, phazi patupa osavala nsapato iyayi.

We can see there that some patients are working there like on this picture, and we are telling people that cancer, on that picture (36) I was sick from cancer but I now, I now work without any problem. It was that I would not wear shoes with on my leg but now I do wear shoes and saying that ooo, it was that the foot would get swolen and the leg too that I would not wear shoes.

I: Chabwino. palinso zina zoonjezera?

Alright, is there any other thing to add also?

07/01: Kuonjezera kwapamenepo nkonena kutino petient wayamba kupezako bwino.

The addition on that its that the patient has now started getting better.

I: Mm mmh

Mm mmh.

07/01: Kuonjezera kwa pamenepo nkonena kuti patient wayamba kupezako bwino, monga titati tionetsetse kuchokera ku koyambirira kuja mwendo utatupa uja sizokuti munthu ungavale nsapato monga akuneneramu amakuuzakuti usamavale nsapato udzivala silipasi komangati munthu wafika saizi yoti unali kumenekujaku ndiye wayamba kuvala nsapato ngakhale okuyang'anira amene amaona kuti ee bambo kapena mayiwa ayamba kuchira. Ndigulu losangalala kunena kuti patient uja wayamba kupezako bwino ndithu, chithandizo cha ku Tiyanjane kuja ndiye tima, apitirizebe kutisamalira ndithu.

The addition on that is that the patient has now started getting better, because if we can see from the beginning when the leg was swollen we it was that you cannot wear shoes like he is saying and they tell you that you should not be wearing shoes but put on slippers but if it happens that you were there and you are now wearing shoes even the guardian is able to see that the husband, or the lay has strted getting better. This is a happy category saying that the patient has started getting better, the care from Tiyanjane, so we do, they should surely continue to take care for us.

I: Chabwino. Pakonapo...azimayitu mwakhala chetetu kapena tipite gulu lina eti, paja mwati zakutsogolozi ndizimene…

Alright, there at the corner…the ladies, you are very quiet or maybe we shouold go to another category you said the ones to come is what…

All: (laughing)

(laughing)

I: Ndiye tupita gulu lina.

So we are going to another category.

09/01: Ine ndionjezereko pang'ono eti.

I should just add a little bit right.

I: Emhe.

Yes

09/01: Ndikhansa imene ndinkadwala ine ndi chisamaliro chimene anandipatsa ku Tiyanjane sindimkadziwa kuti ine ndifika saizi imeneyi pamene ndafikapa. Ndinkaganiza kuti mwinabe miyendo yonse iwiri andidula ndikhala opanda miyendo koma mwa mwayi Mulungu sanafune panopa miyendo sanaidule ndimayenda bwinobwino.

I did not know that I can reach the **size** I am now with the type of cancer that I had and the care that the Tiyanjane gave to me. I was thinking that they will cut off all my legs and I will be without legs but lucky enough God did not want that that they did not cut the legs I now walk without any problem.

I: Mm mmh, chabwino.

Mm mmh, alright.

09/01: Ndi mankhwala amene amandipatsa ku Tiyanjanewo ndi zakudya.

With the medicine and the food that Tiyanjane was giving me.

I: Chabwino. Tipitirize eti? Ndiye tikachoka apa tikupita gulu liti, ili eti?

Alright. So we should continue right? So where are we going to from this category?

08/02: Mmm

Mmm

I: Gulu limeneli tiyeni tilifotokoze, pamenepa pakukambidwa mukamba nkhani yoti bwanji? Tafikatu pagulu lija munati aaa ife tifotokoza kwambiri apa. Ndiye tiyambire painu chemwali.

Lets explain this category, what stories are you telling from this category? We have reached the category where you said that, “aaa, we will explain this category a lot.” So we should start with you my sister.

06/01: Gulu limeneli ndi lanthu ati osangalala.

This is a category for happy people.

I: Mm mmh.

Mm mmh.

06/01: Anthuwa zinthuzi zachokera komweko uku atatha kukhumudwa ndi patient mmene alili kulandira uphungu kuchipatala ndiye ndikufikatu posangalalano tsopano. Anthuwa kutereku alibenso nkhawayi mmaganizo awo basi akuona kuti iii patient wanga akudya, sankayenda akuyenda chinachilichonse akutani akupanga ndiye anthuwa ndi amenewa amene akutani, akusangalala.

These people, these things are coming from here, after being worried with the condition of the patient, receiving counseling from the hospital then reaching the point of being happy now. These people are not anxious anymore there thoughts now are that, they can see that iii, “my patient is able to eat, wasn’t walking but is walking, was not able to do anything but is what, is doing ,” so these are the people who are what, happy.

I: Uthenga wanu umene mukunena ndi onena kuti patient akalandira bwinobwino amapezeka kuti akusangalala, anthu amene akusangalala anthu komanso patient akusangalala eti?

So the meassage that you are giving is that when the patient has received properly he/she happens to be happy, the people who are happy…the people but also the patient is happy right?

06/01: Mmm patient ndi omuyang'anira.

Yes, both the patient and the guardian.

I: Ndiomuyang'anira.

And the guardian.

06/01: Ndi ana chani, monga mukuoneramu eya kusangalala kuti apapa zinthu zikuoneka kuti zili bwino.

And the children etc as you can see, yes happy that now the things here are fine.

I: Chabwino.

Alright.

06/01: ndithu.

Sure

I: Chabwino. Ena amene akufuna aonjezerenso pa pagulu limenelili kuti tukamba nkhani yoti bwanji. Amene tufuna titaonjezera pamenepa?

Alright. Any other who would want to add on this category about what stories are we saying? Those who wants to add here?

07/01: Pamenepatu tikuona kunena kuti anthu amene ali pamenepawa akusangalaladi chifukwa choti ayi tiyang'anitsitse pamwambapo pali ndithu akudya tikati tiyang'anitsitsenso achikulire ena awa (PICTURE) atenga container yawo akudya kusonyeza kunena kuti patakhala kunena kuti patient wawo sali bwino sangatenge container kumadya nawonso amakhumudwa kuti kodi pamenepa zikhala bwanji koma ngati watenga ujeni kumadya kusonyeza kutidi anthu amenewa ali pakati pachisangalalo kunena kuti patient wawo ija wayamba kupezako bwino ndithu.

Here we can see that these people here are really happy, if we can look properly up there they are eating, and if we can see also there is this gentleman is carrying his container eating also meaning that he cannot carry the container and eat if there patient is not fine they also get worried that what will happen here but if they have carried a container and eat it really means that these people are in a midst of happiness saying that there patient has started getting better.

I: Mm mmh. Chabwino. Ena, tafikatu apa achimwene munati kutsogolo.

Mm mmh. Alright. Any other, my brother we have reached here you said as we go along.

All: (laughing)

(laughing)

03/02: Pazithunzi zimenezi zikusonyeza kuti magadiyani akusangalala kuti patient uja amene amadandaula koyambirira monga kuchokera uku, atleast akupezako bwino. ya monga anenera pa point number 3 tikufuna kuwauza anthu ena onse kuti munthu odwala khansa ngati mukumuyang'anira bwino atha nayenso kukhalanso munthu osangalala koma ngati mukumusala kumamtalikira izi ndi izi nde kuti munthu uja sangakhale osangalala chifukwa ndekuti adzikhala ndi nkhawa chifukwa choti mukumusala. ya koma khala nayeni pafupi kumuyang'anira ndekuti magadiyani plus patientiyo nonse mudzakhala osangalala monga zithunzi zikuonekeramu.

On these pictures it shows that the guardians are happy that the patient who got them worried like from here is **at-least** getting better. Like they have said on **point number 3** we would like to tell other people that if you are taking care for the cancer patient he/she can as well be a happy person but if you are discriminating him/her being far away from him/her etc then the person cannot be happy because he will have anxieties because you are discriminating him/her. Yes but be close to him/her, look after him/her it means the **guardian plus** the patient will both be happy like the way it is showing on the pictures.

I: Chabwino. palinso ena amene akufuna kuonjezera. Kodi izizi ndi izi ndi zimodzi?

Alright. Is there any other who wants to add? Are these and these the same?

06/01: Mmm

Mmm

02/01: Eee

Yes

I: zonse zachokera uku?

Both are coming from here?

06/01: malire ndi apa.

This is the boundary.

I: Chabwino. Aliponso ena amene akufuna aonjezere? Mwina bwerani konkuno mukaziona ndi mmatha kulankhula bwino osazitalikira. Mayi (name) tikunenatu za zithunzi zimene zili pakati apa. Mmm mukuti bwanji mau oonjezera. (interruptions) Olo mukhale pampandopo. A (name) muthanso kutenga mpando uwo penanso kuimaku kumatopetsa. Tili pamenepa apa, zinanso zimene tingakambe zokhuzana ndi zithunzi zimenezi ndi zoti bwanji?

Alright. Is there anyone who wants to add? Maybe you should come here you will be able to talk when you are closer don’t be far from them. Mrs (name) we are talking of the pictures in the middle here. Mmm what are you saying, additional words. (interruptions) or you can sit on the chair. Mr (name) you can also get that chair, standing up is somehow tiresome. We are here, what are the other things that we can say about these pictures?

All: (quite)

(quite)

I: Tinkhonza kupitirira mwina gulu lina?

We can go ahead maybe to another category?

FP: Mmm

Mmm

I: Chabwino ndiye tipite, tiyambire liti ili, ili?

Alright so we should go, which one should we start with, this one or this one?

06/01: Mmm, yambirani ili.

Mmm, start with this one.

I: Tiyambire apa eti.

02/01: (laughs)

(laughs)

I2: Awa akuloza kuti apa.

This one is pointing here.

02/01: Iyayi nanga apa si kutchalichi...(overlaps)

No, since this is at the church…(Overlaps)

06/01: Apa mpomalizira apa.

This is the last one.

I2: Eee

Yes

02/01: Mpomalizira.

Its the last.

I: Tiyenino pamenepapa tifotokoze kuti pamenepa pali nkhani yoti bwanji.

Lets go here, we should explain what stories are here.

06/01: Gulu ili ndi la ndi gulu lantchito titero, zantchito chomchi.

This is the work category, working etc

I: Mm mmh.

Mm mmh

06/01: Eya apatient atadwala uko uko atalandira mankhwala, uko atalimbikitsidwa chilimbikitso ku Tiyanjane, atapezako bwino akuyenda, akusangalala aona kuti zonse amasukano bwinobwino ndiye gadiyani naye amamasuka kaya ndi gadiyani amamasuka naye ndikuyamba kugwira chani, ntchito.

Yes, when the patient was sick there, received medication there, has been encouraged with courage from Tiyanjane, has gotten better is walking, is happy and has seen that is free in everything then the guradian becomes free also, either the guardian becomes free and start what, work.

I: Ok

**Ok**

06/01: Kutereku gulu limeneli ndi gulu limene likugwira ntchitono kuona kuti aa apapano ndi ndipangeno njira yoti ndithandizire chani pakhomo panga chifukwa ndamasulidwa muzina ndi zina. Ndiye gulu lake ndilimeneli.

This category, its a category which is working seing that now I should find a way to assist my home because I have been set free in some other things. So this is the category.

I: Chabwino. Ena amene akufuna kuonjezera, pagulu limenelili.

Alright. Any other who would want to add on this category.

02/01: Gulu ilili likuonetsa kuti munthu ali bwinobwino ndithu akufuna kuzithandizira osati kuthandizidwa iyayi ndiye ayenera kugwira ntchito malingana ndikuti tsopano ali bwinobwino.

This category shows that a person is ok and wants to assist him/her self not being assisted so is supposed to work since is now fine.

I: Mmm

Mmm

02/01: Eya

Yes

I: Apapa, safu, sa, sakuthandizidwanso ndi munthu?

Here, he doesnt, he doesnt want to be assisted by a person?

02/01: Mm mm, akuzi...(overlaps)

Mm mm, is…(overlaps)

I: Apa akuzithandiza yekha.

Is assisting him/herself here.

02/01: Akuzithandiza yekha chifukwa chokuti ali bwino.

Is assisting him/herself because is fine.

I: Mm mmh.

Mm mmh.

02/01: Eya.

Yes

I: Mungatambasule pamenepopo kuti apeze bwino mwina chinachitika ndi chani?

You should explain clearly there, what wappened for him/her to get better?

02/01: Eee kuti apeze bwino munthuyo atamva ululu anapita kuchipatala ndiye kuchipatala kuja mpakana amtumiza ku Tiyanjane.

Yes, when he/ she was in pain he/she went to the hospital and was sent to Tiyanjane from the hospital.

I: Mmm

Mmm

02/01: Kumene, kumene anapeza thandizo lotere chomchi kuti zifike mpaka pamenepa.

Where, where he/she got this kind of assistance to get to this point

I: Mm mmh.

Mm mmh.

02/01: Eya

Yes

I: Chabwino.

Alright.

02/01: Ndithu.

Sure.

I: Aliponso ena amene akufuna aonjezere gulu limeneli. Pamenepapa tingaonjezerepo kuti bwanji?

Is there anyone who wants to add on this category? What can we add on this?

08/01: Choonjezera changa ndifunso lakumapeto lija.

My addition is coming from the last question.

I: Mmm mmh.

Mmm mmh

08/01: Ndiziti zimene ndikufuna kuwauza anthu ena kuti adziwe zokhuzana ndi kukhala ndi matenda a khansa. Mwazinthu zimene tingafotokozere munthu ndi zonena kuti munthu ngati apezeka ndi matenda a khansa iyeyo asazilembere malire kuti ndiye apa ndathera pamenepa ayenera kuti apite kuchipatala akapita kuchipatala akalandira chithandizo ndi chisamaliro mwina kutumizidwa ku Tiyanjane, ku Tiyanjane kuja akalandira uphungu oyenerera ndi matenda a Khansa plus kupatsidwa chithandizo, atapatsidwa chithandizo thupi lake lidzabwerera ndikukhala ndi mphamvu atakhala ndi mphamvu adzayamba kugwira ntchito zake momasuka kaya ndi driveradzapezeka kuti akuyendetsa galimoto yake penapaliponse pamene angathe kugwira kaya ndi conductor adzayenera kukhala, kulandira, ku kulandira ndalama ndikuwapatsa anthu ma tikiti awo kaya ndi biyudala adzayenera kumanga nyumba adzagwira ntchito ina iliyonse koma asadziderere kuona kuti ndili ndi matenda akhansa ndisapezeke pagulu pamnzanga ndikugwira ntchito mapeto ake adzakhala ovutika, ovutikitsitsa ndipo adzakhala opempha pachifukwa cha icho tikulimbikitsa anthu ena kuwafotokozera zamatenda a khansa kunena kuti sayenera kutisala ayenera kutipatsa ntchito zimene angathe kutipatsa poyerekeza kuti ifenso ntchitozo ngati tikudzidziwa titha kuzigwira ndikuposanso munthu walungalunga.

What are the things that you would want other people to know about having cancer disease. On the things that we would tell other people is that if a person has been found with cancer he should not limit himself that am done here, he is supposed to go to the hospital and he will receive assistance and care when he goes to the hospital and maybe also being sent to Tiyanjane, It is at Tiyanjane where he will receive the proper counsel on cancer plus because of being provided with assistance his body will come back to having strength, after having strength he will be able to perform his duties freely either is a driver he will be found driving his car everywhere where he can be able to work either is a conductor he will be getting, getting the money and giving people their tickets or either is a builder he will have to build a house and will work on any job but should not look down on himself seeing that I have cancer disease so I should not be found with a group of friends and work as a result he will be found poor, very poor and will be a beggar that is why we are encouraging other people telling them about cancer disease that they are not supposed to discriminate us they are supposed to give us the works that we can be able give to us considering that if we know the work we can perform them even better than a health person.

I: Chabwino. Aliponso ena mwina amene akufuna awonjezere.

Alright. Is there anyone who wants to add?

07/01: Kuonjezera pamenepobe ndikunena kuti ngakhalke enafebe tikudwala nthenda imeneyi koma tili pantchito chifukwa cha mankhwala amene tikulandira ku Tiyanjane amatipatsa mankhwala koma kuona kuti tikuyenda bwino tili pantchito kumagwira podziwa kuti banja lathu iziyenda bwino chifukwa chamankhwala amatipatsa.

Just to add on that also I am saying that even though some of us are sick from this disease we are working because of the medicine that we are receiving from Tiyanjane, they give us medicine but seeing that we are able to walk and we are working knowing that our family should be ok because of the medicine that we are given.

I: Chabwino. Tikupitatu ku gulu lomaliza, pali china chimene tikufuna tionjezere. Tinkhonza kupitagulu lomaliza, kapena gulu limene lili pakonali ndikuona ngati ndilomaliza palinso lina tikachoka apa.

Alright. We are going to the last category, is there anything that we would want to add. Can we go to the last category, or the category at the corner I think its the last category or is there any other from here?

08/02: China ichi.

Here is another one.

I: Chabwino. Ndiye tikambirane chimenecho kenaka tipita apo.

Alright. So we should discuss about that then we will go there.

06/01: Iyayi tiyambire ichi.

No we should start with this.

I: Tiyambire icho?

We should start with that?

06/01: Mmm

Mmm

I: Chabwino. ayi chiikeni pomwepo

Alright. No put it right there.

08/02: Chikhala pa line ichi?

Should this be on the line?

06/01: Iyayi ichi ndi cha ntchitotu.

No this is for work.

I: Simwati gulu la paokha eti?

So you said that has its own category right?

08/02: Mmm

Mmm

I: Eee chimeneco tingakambe nkhani yoti bwanji? Nkhani imene ili pa chithunzi chimenecho ndo yoti bwanji?

Yes, that one what story should we discuss? Whats the story on that picture?

06/02: Mwajambulanu tiyeni.

Go on the one who have taken it.

08/02: isi ajambula ndi awa.

No its her who has taken it.

I: Eee

Yes.

06/02: Chimenecho katundu wanu ameneyo.

There you go, your things there.

08/01: Aaa nkhani imene ili pamenepoyo...(Overlaps)

Aaa the story there…(Overlaps)

05/02: Ine mnajambulidwatu, ine ndiwojambulidwa.

I was taken that picture, It was just taken for me.

08/01: nkhani imene ili pamenepo ndi mb'ale kapena neighbour kapena munthu aliyense amene wapita kukamucheleza, kapena kukamucheleza payekhayekha gadiyani amene akuyang'anira matenda a khansa kuchokera pachiyambi pamene anayamba kudwala munthu afika povuta ndikunyamuka kupita kuchipatala. Panthawi imene ijayi gadiyani amafuna chilimbikitso ndipo amafuna abwenzi okhala naye pafupi kumulimbikitsa kumuuza zinthu zomulimbikitsa pamene paja magadiyani tiyenera kusamala kwambiri chifukwa pali ena amatha kubwera ndi kukhala ngati thandizo koma akukusokoneza chifukwa payenera kukhala pali nkhani ziwiri kapena zitatu. 1 inutu aga, aujeni aa matenda anuwa zitha kutheka kuti ndizopondetsedwa mupite kwa Sing'anga, inutu odwala anuwa musangowayang'anira pita nawoni kutiko kuchipatala mukapita kuchipatala pali abale anga akuti akuti anakumana ndi zakutizakuti zokhuzana ngati, atapita kuchipatala anakalandira uphungu ndikutumizidwa kuchipatala cha Tiyanjane atalandira uthenga kumene kuja kunapezeka kuti munthu anapezano bwino komanso sikuti inu mudzikhala okhumudwa iyayi alimbikitseni odwala anu, asamalireni odwala anu musawathawe muwayang'anire pena paliponse mudziwayang'anira kufikirana adzapeza bwino. Nkhani ili pamenepaja ndi uphungu osiyana siyana ochokeranso kwa azibale ena osiyanasiyana umene ukubwera pamenepo kuonjezera pauphungu umene umachokera ku Tiyanjane.

The story that is there is about a relative or a neighbor or any person who has gone to chill, or chill the guardian, the guardian for a cancer patient from the beginning when he started getting sick and things went bad and leave for the hospital. Its at that moment when the guardian needs courage and needs friends to be close to her and encouraging her telling her encouraging things and it is that time that us the guardians needs to be very careful because there are some who happens to come as if with assistance but they are there to confuse you its because its supposed to be 2 issues. 1 its hey you this your disease can be that you were bewitched you should go to the witch doctor or you should not be just looking at you patient take her to the hospital when you go there at the hospital there was this my relative who experienced this and that and when she went to the hospital they were counseled and sent to Tiyanjane and she got fine after receiving a message there and you should not be worried but encourage your patient, take care of your patient and don’t run away from her but take look after her everywhere until when she will get better. The issue here is different counsel from different relatives coming there in addition to the counsel from Tiyanjane.

I: Mm mmh, ndiye apapa mufuna kutanthauza kuti achibale amatha kubweretsa mauphungu osiyanasiyana

Mm mmh, so you here you mean that some relatives happen to bring different counsels?

08/01: Mauphungu osiyansiyana.

Different counsels.

I: Ndiye odwala afunika akhale osamala.

So the patient needs to be careful.

08/01: Osamalira.

Careful.

I: Chabwino. palinso ena amene afuna aonjezere pa, pankhani ili pamenepayi?

Alright. Is there any one who would want to add on this, the issue at hand?

06/01: Pang'ono chabe.

Just a little bit.

I: Mmm

Mmm.

06/01: Zikhoza kutheka kuti achibalewo abweretsa mfundo yoti tipite kwa Sing'anga, kumene kujanso ndiye timakatenga mavuto ena chifukwa msing'anga zimene amachita iye amakuchekacheka ndiye munthu odwala khansa mmene ndimadziwira ukachekedwa pamatuluka madzi mapeto ache bala, bala lija likukula kupezekanso kuti tili ndi bala mmalo moti tinakati tisapite kumenekuja kukachekeresa tipite kuchipatala anakatithandiza moyenera ndi mmene iwo anaphunzirira koma iye msing'anga amangodziwa kucheka ndikupaka zimankhwala zakezo ndiye tikupangitsanso vuto kuti lipite patsogolo tikuchedwa kulandira chithandizo choyenera mapeto ake iii akundilodza akundilodzamapeto ake osakhala bwino kupezeka kuti munthu vuto likungopitirira pitirira kulepheranso kumasuka pabanja pako kaya abambo amagwira ntchito kaya ndani amene akutiyang'anira amagwira ntchito zonsezo zikhonza kuima chifukwa cha iwe mmene waganizira chisankho cholakwika tikuyenera tikapeza vuto tipite kuchipatala ndipo kuchipatala sangakuuze kuti iyayi iwe poti ukudwala khansa pita kwa sing'anga mm mm. Izizi zikutiunikira kuti tiyenera tikapeza vuto tizipita kuchipatala.

It can happen that the relatives have brought the issue that we should go to the traditional doctor so it is there where we get other problems because what the traditional doctor does is to cut you so the way I know a cancer patient you produce some water when you have been cut off as a result there is a wound and that wound would grow and finding out that we have a wound, had it been that we didn’t go there to get the cuts instead go to the hospital we would have been assisted properly regarding to what they were taught but the traditional doctor just knows to cut and pour in some medicine making the problem to continue we are being late to receive proper medication as a result, “iii they are bewitching me.” As a result not becoming better the problem is persisting failing to be free on your family if the husband works or who ever is our guardian was working it can all stop because of the wrong thought and decision you made. We are supposed to go to the hospital and the hospital cannot tell you to go to the traditional doctor just because you are sick from cancer mm mm. This is just telling us that we should go to the hospital when we have a problem.

I: Chabwino. Munafotokozapo zonena kuti mwina kupita kwa sing'anga kupangitsa kuti pakhomo zinthu zina zisokonekere ngati mwina okuyang'anira amagwira ntchito zisokonekere, mungangofotokoza bwinobwino?

Alright, You explained that maybe going to the traditional doctor can make some things at home to not be alright if the guardian maybe works it will not work, can you explain clearly?

06/01: Ee

Yes

I: Kusokonekera kumeneku muti mutanthauza kwanji?

What do you mean by not being alright?

06/01: Tiyerekeze ineyo ndapita kwa Sing'anga ndi amene akundiyang'anira kumenekuja akagula lezala lawo apala mankhwala awo mmene amachitira muja ii khalani apa miyendoyo tapangani chomchi ndiyambe kukuchekerani akuchekera chekera sikhalatu mphini imodzi azungulira mwendo mmene angafunire aini akewo nde iwe ukachoka kumenekuja ukapita kunyumba muja achekamu mwayamba mabala ndiye kuti uja sangathe kumasuka kupita ku ntchito chifukwa aziti akaona ii ayi munthuyi wayambanso mabala vuto ili latani labweranso kupezeka kuti iye uja kumatanganidwa ndikukusamala mpamene iweyo utati wapita kuchipatala uzidzikako bwino udzigwira wekha zintchito vuto limene likhoza kubwera lokuti gadiyani sangathenso kuchoka yi pamenepo adzikhala ndiiwe kukusamala pamene iwe wapeza vuto, vuto loliputa dala, ukubweretsa vuto pavuto limzake. Ndiye zimene zitha kupangitsa kuti banja likhale lotanganidwa.

Lets imagine that I have gone to the traditional doctor with my guardian and they have bought a lazer brade and has made their medicine like the way they do, “iii sit down there and let your legs like this I should start cutting you, and he cuts and put in the medicine and it wont be only one place they will surround the whole leg the way he wants and from thee when you get home you will see that there are wounds in the cuts made which means that person cannot be free to go to work because will see that ii this person has started having wounds this problem has developed also finding out that the person is bust with taking care while if you can go to the hospital you will be getting better and you will be working yourself. The problem that can come is that the guardian cannot leave has to be with you to take care for you while you have got a problem, a problem that you have brought your self, bringing a problem on another problem. So that can make your family to be busy.

I: Mm mmh

Mm mmh

06/01: mmm

Mmm.

I: Nde mwati mukubweretsa vuto pa vuto limzake vuto lina limene likubwera pamenepo ndi vuto lanji?

So you said you are bringing a problem on another problem, whats the other problem adding on that?

06/01: Lamabala iweyo unali ndi khansa, iwe mwina suma, sunali ndi balayi koma mwendo unangotupa chabe moti khansa imeneyo koma chifukwa choti wachekera pabwerenso vuto, pamatenda amane aja waonjezera mabala ndiye likhonza kupangitsa kuti mabala amenewowo likhalenso vuto lina limenelo.

The wound, you had cancer so maybe you were not, you did not have wounds but the leg was just swollen and that was cancer but there has come another problem because of the (cut from the traditional doctor) you have added the wounds on the disease so it can make those wounds to be another problem.

I: Chabwino.

Alright.

06/01: Eee

Yes.

03/02: ndingoonjezeranso pa awa kuti vuto linanso limene lingakhalepo ndi lokuti munthu ukapita kwa sing'anga mmm, sing'anga choyambirira amayamba kugwira munthu aaa iyayi mwendo umenewu kuti utupe ndi akuti akuti akulodzani. ndiye zotsatira zake mukachoka kwa sing'anga kuja mukangofika kunyumba basi mapokoso pakhomo paja mmmalo moti inu chidwi chanu chonse chikhale pomuyang'anira patient uja kupezeka kuti basi mwayambanso kutani kulozana zala ndiye zotsatira zolozana zala zija kupezeka kuti mwina pakhomopo ena anyanyala and chifukwa mwina sing'anga akuwanamiza kuti aaa mwina ndi agogo abu aja akuwatani akuwalodzawo zotsatira zake mukangofika kumene kuja ndewu kapena kutukwanizana zotsatira zake mwina ndi agogo aja akunyanyala akupita kukatani kukakhala kwina kapena inuyo mwina mwaatani mwathamangitsa ndiye kupezeka kuti mukuthamangitsa munthu amene mwina amamuyang'anira patient uja chifukwa choti mwamvera zokamba za sing'anga chifukwa msing'anga paliponse salephera kugwira munthu ufiti aaa apatu ndikuuzeni, aaa taonani pagalasipa ndi akuti akuti ndi akuti akuti pamene zili zabodza angofuna akudanitseni.

I should also just add on her that the other problem that can be also there is that when a person has gone to the traditional doctor mmm, firstly the traditional doctor finds a person to blame aa it is this person who has made your leg to get swollen, as a result when you come home from the traditional doctor your start quarrels at home other than concentrating on taking care for the patient you start pointing at each other so the result for pointing at each other its found that maybe some gets angry and its because the traditional doctor have lied to them that aa its the granny (abu) who is bewitching him as a result soon after arriving home you start fighting or insulting each other as a result maybe the granny gets angry and leave to stay somewhere else or maybe you have chased her out so it is found that you have chased a person who was maybe supposed to be taking care of the patient because you have taken head of what the traditional doctor was saying because a traditional always finds some one as a witch all the time, “aaa I should tell you here aaa look on the glass its that one, its that one,” while its all a lie he just want to make you people hurt each other.

I: Chabwino. Tipitirize kapena palinso amene ali ndi choonjezera pagulu limeneli?

Alright. So we should continue, is there anyone with additions on this category?

06/01: Mm mm.

Mm mm.

I: Timalize eti. Ndiye tikupita pakona apo. Tiyeni tifotokoze pamenepo kuti pamapeto penipenipo gulu limenelolo tikutantha, pali nkhani yoti bwanji?

We should finish off right. So we are going to that corner. Lets explain what the last category means, what story is there?

06/01: Gulu limeneli ndi lamapemphero.

This is a prayers category

I: Mmm

Mmm

06/01: Komanso pali tchalitchi.

There is also a church.

I: Mm mmh.

Mmm

06/01: Eya mmene tafotokozera poyamba uku kuti munthu wadwala walandira, wapita kuchipatala walandira mankhwala wamwa wayamba kupezako bwino apo akusangalala, akulimbikitsidwa, akugwira ntchito zotsatira zake pazonsezi wayamba kumasuka mpakana kumapezeka kugulu lotani, lopemphera chifukwa sizingatheke munthu amene ali uku akudwalayo kukapezeka malo awa, sizingatheke limatenganso step gulu ili kumapita kwa iyeyo mapeto ake munthu wachira ndiye iye akutenganso step kupitanso kuti, kumapemphero.

Ok, the way we explained at first that a person is sick and has received, has gone to the hospital and received medication, has taken them and is now feeling better and is happy there, Is being encouraged and is working the results from all these is that has started being free to the point of being found at with what group, the praying group, because it cannot be possible for a sick person to be found at that place, it cannot be possible…this group also takes a step visiting him as a result the person is fine and he is taking the step going there also, to the prayer group.

I: Mm mmh.

Mm mmh

06/01: Zikutitanthauzira kuti ngakhale tili mmavuto ngakhale tili mmaululungakhale tili kakasi muzina ndi zina tikuyenerabe ifeyo kumamutchula Mulungu chifukwa mankhwala nthaiw zina paokha sizingathekeyi koma ngati ukukhulupirira wekha nde mpamene iweyo umatani umapezeka uli mgulu limenelo ukutani ukumasulidwa nawo. Amzanga muzipitiriza pamenepo.

It means that even if we are in troubles even as we are in pain even we are stranded in some other things we still need to mention God because the medicine cant work on their own but if you yourself believe its when you are found in that category being set free. You my friends should continue from there.

I: Chabwino. akuti enafe tipitirize iwo alankhula mbali yawo. Ena tionjezera kuti bwanji?

Alright. She is saying we should continue she has spoken on her behalf. What are we adding?

08/01: Ine kumeneko, pamene tapezeka tili ndi vuto la, tili ndi matenda a khansa pamoyo wathu wa tsiku ndi tsiku timayenera kumulemekeza Mulungu kubadwa mpakana kukula kufika tsiku limene Mulungu adatiikira malirepo. Pachifukwa cha ichi chinachilichonse cha moyo wa tsiku ndi tsiku tiyenera kumpembedza ndi kumutumikira Mulungu. Ndichimodzimodzi ngati apa kukhala nawo mmagulu nkumagwira ntchito chifukwa walandira chithandizo uku chimene chili choonjezera pakuthupi ichi. muuzimu Mulungu ndi amene amabwera nkukwaniritsa. Paichonso tidziikizanso mmanja mwa Mulungu anthu achipatala madokotala ngakhale ma nurse ngakhale amene amagwira zakafukufuku amenenso amatha kupeza nzeru zotha kupezera mankhwala oti ifeyo tikathandizikire nawo. Nidikukhulupirira kunena kuti nkhani imene ndikunenayi ndi nkhani iyi. Ndikhulupirira kuti munthu uyuyu uyu apapa akupemphera pamene akupemphera uyuyu pali nkhani zingapo utha kuikiza chifukwa cha vuto lako la kudwala kapena cha vuto la odwala wako, kapenanso ma dokotala amene amapereka chithandizo chimenechichi kuti Mulungu akathe kupereka mzeru mwa anthu amenewa komanso kwa azakafukufuku amene amatha kupeza mankhwala Mulungu akathe kupereka nzeru chifukwa palibe chimene chili chosatheka ndi Mulungu. Mulungu amadzera mwa munthu ndikumpatsa nzeru nthenda imene tikuitenga ngati kuti ndiyopanda mankhwalayi tsiku lina kudzera mmene tililimu pogwiritsa ntchito pemphero adzapereka mnzeru mwa anthu awiri kapena atatu tidzapezeka kuti tonsefe tachira, zikomo.

Myself on there, whenever we are found with the problem of, although we have cancer disease on our everyday life we are supposed to thank God for being born up to growing up to the day that God put boundary. For this reason we are supposed to glorify and serve God. Its the same with being in these groups and work because you have received treatment from here which is an additional to the fresh and God comes in in spirit and satisfies. It is for this reason also that we should be putting in Gods hands the medical personnels, the doctors, even the nurses and even the research people who also find wisdom to find the medicines for us to be assisted. I believe that this story am talking about is a story. I believe that this person here is praying so while praying there are a lot of stories that you can pray about because of your sick problem or your patients even the doctors who provide this treatment so that God can give wisdom to these people and also to the research people who are able to find medication, God should give them wisdom because there is nothing impossible with God. God uses a person and gives him wisdom that the disease which we are thinking that it doesnt have medication one day through the way we are through prayers He will give wisdom to 2 or 3 people and we will find out that we are all healed.

I: Mm mmh. Palinso ena amene akufuna aonjezere?

Mm mmh. Is there any other who wants to add?

07/01: Kuonjezera pamenepopo tingonena kuti Mulunguyo ndiamene amapereka nzeru nde amaperekanso nzeru kwa ma dokotala kuti madokotala tengani mankhwala akuti mumpatse munthuyu achire ndiye Mulungu yemweyonso amaperekanso madalisto kwa odwalayo kuti uchire nde tingopemphabe Mulungu kuti a pereke nzeru kwa madokotala kuti atithandize ifenso odwalafe kuti tipeze machiritso.

In additional to that we should just say that God is the one who gives wisdom, so He also gives wisdom to the doctors that doctors take these medicine and give it to this person for his/her cure so its the same God who also blessed the patient to get healed so we are just asking God to give the doctors the wisdom to help us the patients to find healing.

I: Chabwino. Tafika pamapeto pa zokambirana zathu ndiye tisanamalize kwenikweni chemwali anga ali ndi zoonjezera.

Alright, we have reached the end of our discussion so before we complete everything my sister has something to add.

I2: Ndiye timangofunanso kudziwa kuti mwakamba zambiri zokhuzana ndi ku Tiyanjane zambiri zabwino kwambiri, funso lina ndi lonena kuti mwina poyambirira pamene inuyo mmadwala kupita lkuchipatala kupita ku, kukupititsani ku Tiyanjane munkaganiza kkuti, munkafuna nthenda yanu ya khansa itachira eti?

So we also wanted to know you have spoken a lot about Tiyanjane, a lot of good things, the other question is that maybe at first when you were sick and went to the hospital and they sent you to Tiyanjane were you thinking, you wanted your cancer disease to get cured right?

All: Eee

Yes

I2: Nde mwapezeka zoti mwapita ku Tiyanjane ogwira ntchito ku Tiyanjane akuuzani kuti nthenda imeneyi ya khansa simachira, pazinthu zimenezizi muganiza kuti zinakuthandizani kapena sizinakuthandizeni?

So it has been found that you went to Tiyanjane and the Tiyanjane personnels have told you that this cancer disease is not curable, do you thunk these things assisted or did not assist you?

07/01: Zinthu zimenezizi tinene kuti zinatithandiza chifukwa monga ineyo poyambapo mwendo utatupa ananena kuti munayenera utadulidwa umenewuwu koma chifukwa cha mankhwala amene anandipatsawo ndikuona kuti simmavala nsapato pano ndikuvala nsapato ndiye ndikuona kuti zinandithandiza chifukwa choti anandipatsa mankhwala panopa ndikuyenda ndiye kuti zinatithandiza.

We should say that these things assisted us because like myself when my leg got swollen they said that you were supposed to get it cut off but because of the medicine that they gave me I can see that I wasn’t wearing shoes but now I can wear shoes so I think that it really assisted me because they gave me medication and I can walk now so it assisted me.

I2: Mmm, chabwino. Ena?

Mmm, alright. Any other?

08/01: Aaa ineyo ndimaona kuti ndi zothandiza, amati ku, uli pankhondo ndiye ukumenyana ndi mdani ngati sukumuona nkhondo yake imakhala yovuta koma ngati wamdziwa mdani iwe umakhala ochenjera komanso amatiuza kunena kuti chifukwa choti muli ndi vuto ili ifeyo tiyesetsa pazimene tili nazo tizikupatsani kuti inu muzikhalabe ndi moyo komanso ndi mphamvu ndikuziwa kuti, zikutanthauza kunena kuti ii aa monga achipatala nzeru za ku thupi zikuthera patipo pamenepo koma kwinako adamusiyira Mulungu angachite ndi ndani ndi Mulungu chifukwa Mulungu ndinamvanso tsiku limenelolo ntakumana ndi adokotala anandifunsapo kuti mwaaona awa ndati eee, amene aja inunso muyenera kupita kumene iwo anapita chifukwa mmene anabwerera week yathayi ndi mmene abwerera lerolo zikusiyana kumene ananenako ndi ku mapemphero kusonyezakuti nzeru za dziko zathera pamenepo sitikunyoza iyayi nzeru za dziko zathera pamenepo nde pothera pamene pajapo adapereka chilimbikitso kuti Mulungu yekha ndi amene ali wamkulu palibe chosatheka ndi Mulungu, chimene, aa chosatheka ndi munthu chimene Mulungu chingankanike ndi chifukwa chake kukubwera pemphero lija. Kwa ineyo zinandipatsa chilimbikitso chifukwa chani, ndikhulupirira kunena kuti kuchokera panthawi imene ndinapezeka ndi mwendowu ndikukhulupirira kunena kuti kusamalira ana anga kukanakhala kovuta, kusamalira banja langa kukanakhala kovuta chifukwa nthawi imeneyo pafupipafupi ndimapezeka kuti ndagona pansi, pafupifupi ndagona pansi koamanso ndimafotokoza kunena kuti chifukwa cha vuto limeneli linapangitsa kunena kuti ngakhale chitonzo chimene amachilandira akazi wanga chinali chopangitsa kunena kuti kupanda kulimba akanatha kungonyamuka bwenzi ndili ndekha ndiyeno pachifukwa chimenecho chondilimbikitsa chomcho nkulandira mankhwala nkuona kuti ndikutha kugwira ntchito kumunda ndikupita, ndipo ndimakumbukira nditapita kumunda apongozi anga samadziwa kuti mwendo wanga ulu bwanji iyayi koma ndikuumba zidina ndipamene adaona mwendo ati inu akudwala mwendo aja ndi awawa akuumba zidinawa? Eti kusonyeza kuti chilimbikitso chimene ndinachilandira kuchokera ku Tiyanjane ndi chisamaliro chidapangitsa kunena kuti ena ake ngati apongozi anga adachiona zidapangitsa kuti ngati anthu ena manena zachipongwe ndiumboni umene adauona apongozi anga a ineyo zidapangitsa zoti agwetsa chifukwa ndinagwira ntchito ndiye ndichilimbikitso kwa ineyo ndipo ndili ndi moyo ndikukhala ndi mphamvu kusiyana ndi mmene ndinaliri pachifukwa cha icho ndimathokoza chifukwa cha uphungu wawo chifukwa adatiuza mchoona chake kunena kuti mdani muli naye mdani akunena kuti atha kukhala ndi malire koma ifeyo tiyesesa kukupatsani chithandizo kuti mukhale ndi chani kodi, ndi moyo kufikana pamene Mulungu adakutanini, adakuikirani malire. kwa ineyo ndidachilandira bwino kwambiri.

Aaa I see that its helpful, they say that if you are at war fighting the enemy if you cannot see him the war becomes difficult but when you know the enemy you become very clever and they also tell us that since you have this problem we will do our best we will be giving you whatever we have for you to be alive and strong. Here it also means that he hospitals wisdom being for fresh ends there but they left it all in God because God is the one who can do it and I also heard that day the doctor asked me that, “have you seen this one?” and I said, “yes.” and he said you should go where this one went because there is a different with the way he came here last week and this week and he was talking about prayer places meaning that the earth’s wisdom ends there, we are not insulting but that is the end of the earth’s wisdom they gave courage that it is only God who is high above and there is nothing impossible with God, aaa nothing impossible with a human being which can fail God that is why the prayer comes. It gave me courage you know why, I believe that from the time I was found with this leg it could have been hard to take care of my children it could have been hard to take care for my family because I was found on sick bed frequently and I also explain that it is because of this problem that even the humiliation that my wife got it was to the extent that if not strong she could have left I could have been alone so for that reason, for giving me that courage and received medicine and seeing that am able to work, I can go to the field, and I remember when I went to the field my in-law did not know how my leg is but when I was molding bricks its when she saw the leg and was like, “you, the one molding the bricks is the one who is sick from the leg?” You see meaning that the courage and the care that I got from Tiyanjane made it that some others like my in-law saw it, it made other who were talking lots of bad things with the testimony that my in-law got aa I think that we destroyed them because I worked, so its a courage to me and I am alive and strong comparing to the way I was so it is for that reason that am grateful because of their counsel they told us the truth that you have the enemy and the enemy is saying that you can have the end but we will do our best to provide you assistant to what, to be alive up to the point where God said that this is your end. To me I accepted It very well.

I2: Ndiye mutatimasulirako mukumati nkhondo, mdani muli naye?

So if you can explain clearly, you are saying wars, you have an enemy?

08/01: Nkhondo mthenda ilipo, ili umu ndiye adafotokoza kunena kuti mankhwala amene tili nawo amene akupezeka panopo ndioti sangathe kuthetsa vutolo koma ndiochepetsa ululu umene inu mungakhalenawo. Kusonyeza kuti ndi nkhondo imene, oti penapake ukagona ukakhala mmaganizo umatha kumaziona kuti zoona ndili ndi mwendo, ukamayerekeza mwendo uwu ndi uwu, mwendo uwu unali ngati uwu ndi nkhawa inayake imene munthu umatha kukhala nayo mumtima ndiponso kupanda kuganiza bwino bwino pali ena oganiza udyo atha kupezeka mwina kuti adzikweza kudzidulira moyo nkhondo yamumtima imene ija yoti ngakhale akazi ako umagona nawo chomchi sangathe kudziwa kuti nkhondoyi mumtima mwake ndimotani. Koma chifukwa cha chilimbikitso cha a Tiyanjane zimapangitsa kuti nkhondo imene ija izitani kodi idzichepa.

The war is that the disease is there, its here so they explained that the medicine that we have which are available now thay cannot end the problem but help reduce the pain that you can have. Which means it is the war which, its that sometimes when you are sleeping and you are thinking you see yourself that is it true that I have a leg, when you comparing this and this leg, was this leg like this one? Its a certain anxiety that you have as a person in your mind and if not thinking properly, there are some who thinks improperly they can end their lives, that heart war which not even your wife whom you sleep with right here can know what kind of war is in his heart. But it is because of the courage from Tiyanjane that makes this war to be reduced.

I2: Mm mm, chabwino. Kaya ena?

Mm mm, alright. Some others?

06/01: Ineyo kumbali yanga ndinakhumudwa. Atandiuza kuti nthendayi ilibe mankhwala. Mankhwala, chimene tingakuthandizeni kuno ndichongoti mudzipezako bwino mudzigwira ntchito zanu pakhomo. Chimene chinandikhumudwitsa ndi chokuti mmene ndimanyamuka kuchoka kwathu ndi mtima onse ndinati ndikupita kuchipatala ndukalandira mankhwala oti ndichile monga mmene munthu ukatsehula mmimba ukapita kuchipatala umalandira mankhwala ndikutani ndikuchira ndiye inenso ndimayembekeza kuti mmene andiuza kuti nthendayi ndikuyenera kupita ku Tiyanjane kumeneko ndikalandira mankhwala ndichila ndinapita ndi maganizo amenewo. Nditafika kumeneko mpamene amadzandifotokozera kuti aaa apapatu nthendayi tingokupatsanimo jackson chanichani, mankhwala ma pain killer koma nthendayi mankhwala eni eni ochiritsa palibe ine ndidakhumudwa moyo mwanga chifukwa mnaganizira kuti aa ndiye ngati achipatala akuti nthendayi ilibe mankhwala nthenda imeneyi yayenera kuti ndiyoopsya kwambiri zinandipatsanso chani maganizo mmoyo mwanga komano mkati mokhumudwa momwemo pachifukwa chokuti ndimapita pita kundirembera ma date mubwere tsiku lakuti mubwere tsiku lakuti ndiye ndi mmenenso amaperekera uphungu mnayamba kumasukano mmachani, kakhumuditsidwe, kukhumudwa kuja kunayambano kundichepera kuti aa izizo ndikuyenera kuvomereza chithandizo chimene ndikueyenera kulandira ndi chiti chimenechi sindidakayeneranso kupitirira kukhumudwa mpakana kufikana lerli ayi ndimangokhalabe olimba mtima basi ndi kumapemphera komano pachiyambipo ine ndinakhumudwa chifukwa choti ndinamva zoti nthendayi ilibe mankhwala.

To my side I was very disappointed when they told me that the disease doesn’t have a cure, the medicine, what we can help you with here is that you should be better and work on your household chores. What disappointed me was that when I was leaving home with all my heart I told myself that I am going to the hospital and I will receive medication and be healed just like the person who has diarrhea goes to the hospital and receives medicine and get healed. So I was expecting that since they have told me that I should go to Tiyanjane with the disease I will also receive medication and I will be healed so I went with those thoughts. So when they told me that here we will only give you the injection and the like, the medicine, the pain killers but the disease doesn’t have the medicine to completely cure you my life got very disappointed because I thought that aaa if the medical people are saying that the disease does not have cure then it means that this is a killer disease, it also gave my life some other thoughts but still while disappointed because I was frequently going there and writing the dates for me that you should come on this day and this day and the way they give their counsel I started freeing my mind, the disappointment, I started being relived with my disappointment that aaa I should just accept this, and this is the assistance that am supposed to receive I could not continue being disappointed up to date am strong and praying also but I was disappointed at first because I heard that the disease doe not have a cure.

I2: Mmm

Mmm

06/01: Eetu

Sure

I2: kaya ena ife tikuti bwanji, titauzidwa kuti nthendayi ndiyopanda, ma, siingachire?

What are the others saying, when we were told that this disease doesn’t have, it cannot be cured?

02/01: Aaa ineyo nditapita kuchipatala atandiuza kuti khansa mumaidziwa ndiye mnawauza kuti khansa ine ndimangoona kwa anthu koma sindinadwalepo kapena abale anga. ndiye anati imeneyi ndi khansa ndiye atandipatsa uphungu kumene kuja mnayesesa kusatira uphungu uja ndinakhala ngati kuti mpaka kufika moti mmene mulilimu chifukwa ndimati ndikapanga chomchi mwendowu umakhala ngati kuti uwu wamuthu wina, uwu wamunthu wina nsapato ndimavala oversize, sindimavala size yanga 8 iyai ndimagula 10 yoti mwendo winawu osakwana winawu ndikumati sha koma mwendo nsapatoyo ndi yanga koma pamene amafika pondipanga kuti aa mmene mulirimutu inu muyenera kuima kaye sitinaseke ma file koma ndi mmene mulilimu ndi mmene zilili nanenso nkuona kuti aa apapo ndimmene zilili ndimangoyendera tsopano panado ndiye pakali panopa ndikuyamikira ndimmne anandiuziramo ndimmene ndililimu ndikuona kuti chipatala ndi chothandiza ukamveranso malamulonso akumeneko akuchipatalako. Eetu

Aaa when I went to the hospital when they told me that do you know cancer so I told them that I just hear of cancer but I have never got sick of it not even my relatives. So they said that this is cancer so when they counseled me there I tried to use the counsel up to the way I am now because it was that if I do this it was like this leg is for someone else while the other one is someone’s else too. I was wearing **over-sized** shoe, I wasn’t wearing my **size** 8 but I was buying size 10 which was not fitting the other leg while the other and was saying that wow is the shoe on the leg mine but when it reached the point of telling me that aa you are supposed to stop with the way you are now, we haven’t closed the files but because of your condition now and I also did see that aaa my condition now I depend on panado so I am grateful now with what they told me and my condition I can see that the hospital is very helpful when you have also followed the procedures from the hospital. Sure

I2: Mm mm ndiye atakuuzani kuti nthenda imeneyi simachira inuyo, kwa inuyo ndi maganizo anu amene munali nawo...(Overlaps)

Mm mm, so when they told you that this disease doesn’t get cured, the thoughts that you had…(Overlaps)

02/01: Poyamba ndinakhumudwa nawo nanga si akundiuza kuti chinthuchi angakhale mugwire ntchito, kukuuza kuti aa angakhaletu mugwire ntchito inu palibe chomwe mungapeze munthu umakhumudwa koma wina akakulimbikitsa mwakuti koma anati aaa muone enawa mmene mukuonera awowo mmene analiri ndiponso kuchipatala kujanso chimene timapanga ndi choti tikafika penapake timatha kuwapatsa ena ake kaya iwe unatsogola nkuwauza kuti iyayi akuluakulu ayambe awa malingana ndi kadwalidwe koma kupezeka kuti amene ukuwapatsa mpata wo kupezeka kuti ndimmene analili zimakhala zolimbikitsa chifukwa chake kukhumudwa kuja kunatha tsopano nkuyamba tsopana ngati iweyo ochira tsopano kuona kuti aa nanga sikukhumudwa kumaphatikizana ndi matenda tsopano kukaphatikizana ndi matenda sumachira kwambiri chifukwa choti ukukhumudwanso mbali ina yake chifukwa mtima ukatsimikiza kuti aa apapa nthawi zachitikazachitika basi ndimmene tinapangira kuti aa bola mmene zilirimu ndi dongosolo tiapange ndichifukwa chache tinakhala ngati kuti ngati akundiuza kuti mwachira ngati zitakugundikani mutha kubwera ndiye mmene mukundioneramu ndikugwira ntchito , eetu.

I was disappointed at first since there are telling me that although you can work on this things, they are telling you that, “ even if you can work there is nothing you can gain,” so a person does get disappointed but when someone has encouraged you that this and that but they said that aaa see the others the way you see that one the way he was and what we do at the hospital is that sometimes when you get there you happen to give anchance to some other person to go ahead of you telling them that gentlemen let this one be first with their sickness condition but finding out that the way that person you were giving a chance was it gives you courage that is why the disappointment went and you start seeing yourself as a person who is fine and seeing that aaa since the disappointment mixes with the disease so when its mixed with the disease then you do not get better because you are disappointed at some point because once the heart accept that aaa this is the time, things have happened and are the way they are that is the way we did that aaa its better the way they are now we should do the procedures that is hwy we did like when they were telling me that you are fine but if it can start again you can come, so they way you can see me now am able to work, sure.

I2: Chabwino. aliponso ena?

Alright. Is there any other?

03/01: Ifenso koyambirira mmene anatiuza kuti maknkhwala palibe tinali ukhumudwa kwambiri chifukwa tinkaganiza kuti ndiye kuti basi after mwina miyezi kapena mwina chaka ndekuti mayiwa mwina akhala atatisiya koma chifukwa chomapita ku Tiyanjane kumakalandira mankhwala ake omwe ena ngati mofin (xxx) awa ali ndi moyowa. Komadi panthawi imeneyo tinakhumudwa kwambiri. Ya moti panopa tikuona kuti iyayi akumazisitira okha moti kusita samatiuza zoti tiasitire ayi kuchapa okha, kusita okha, kuphikanso okha. Eya koma panthawi imeneyoyo titauzidwa zoti palibe mankhwala oti angachiliretu eee nkhawadi nde inalipo kwambiri chifukwa tinkaganiza ndiye kuti miyezi kapena chaka ndiye kuti basi zikhala zitatani, zitavuta koma panopa 3 years yatha moti timangopita pena akakhala kuti iwowo ali ndi mphanvu amatha kupita okha ndithu. ya kukwera minbus ulendo ku Tiyanjane kukalandira mankhwala miyezi nkumatha, zaka nkumatha penanso ine ndikaatengere moffin ndi ena ndi ena zaka nkumatha. Ndiye kutaya kumene tinali nako koyambirira panopa kunatha ya, or else iwonso mwina ndikukhulupirira zoti anataninso atleast panopa confidence inabwera.

We also were very disappointed when we were first told that there is no medication because we thought that it means the mother will leave us after some months or maybe a year but because of going to Tiyanjane frequently and get the medicine like **mofin** (xxx) here she is alive. But that time we were very worried. Yes that we can see her ironing her clothes she does not tell us to iron her clothes now, she wash her clothes, iron and even cooking for herself. Yes but that time when we were told that there is no medication to cure her eee there was much anxiety and we were thinking that maybe it will what, will be hard on us after months or a year but its 3 years now that we just go and sometimes when she is strong she goes herself. Yes she boards a min-bus to Tiyanjane to get the medicine and months are passing, and years passing or is should go and get **mofin** for her and the other medicine and years are passing. So the lost mind that we had at first its gone now yes **or else** I also believe that even herself she also did what, she **at-least** gained **confidence**.

I2: Mmm

Mmm.

03/01: Ee ndithu

Yes sure.

I2: ndiye kutaya mtima kumene munali nakoko kapena kukhumudwa kumene munali nako kunabwertsa mabvuto ena alionse nthawi imeneyoyo?

So did the lost of your mind or the worries that you had bring any problems to you that time?

03/01: Eee tinganene zokuti tonse tinakhala ngati tinataya mtima.

Yes, we can say that we all lost our minds.

I2: Mmm

Mmm.

03/01: Eee titangouzidwa pachiyambi kuti apatu palibe mankhwala okuti angachiliretu eee nkhawa ndiye inalipodi ndithu.

Yes, when we were first told that there is no medication here that would cure her completely eee there was much anxiety.

I2: Mmm

Mmm

03/01: Eee komano pang'ono pang'ono tinaona kuti iyayi ku Tiyanjanedi kwatithandiza ndithu chifukwa zintchito ndithu akugwira okha ndithu. Ee chifukwadi panthawi imeneyi ndiyotino memory inathawa eee pachiyambi mwina akapanga chinthu kuiwala chani chani koma panopo aa chilichonse amakumbukira ndithu. Eee ndithu bola mankhwala tiapatse moyenerera. 2 nso tsiku loti lakuchipatala monga apapa apita mmawa ndiye kuti tiyesese kutidi akatipatsa date ku Tiyanjane date imeneyoyo tisaiphonye tipitedi tikatengere mankhwala aa mankhwala ndi awa kapena pena apite okha aa mankhwala ndi awa kamwedwe kake ndi kaya 5 days per day akwaniritsedi ndithu. Ndiye kuti iyayi tikuona kuti zaka zikutha akadali ndi mphamvu chomchi.

Yes but we still saw that Tiyanjane has assisted us little by little because she can work on her roles. Yes because at that time her **memory** was lost, yes at first she would forget when she has done something like that, but she does remember everything now. Yes as long as we are giving her the medicine the way it supposed to be. Secondly the hospital dates for example she is supposed to go tomorrow so we are supposed to make sure that she does not miss the date given at Tiyanjane but we should go and get the medicine for her, “aaa these are the medicine,” or sometimes she go herself aa these are the medicine and she should follow the medication proper way of taking them either **5 days per day**. So we can see that the years are passing while she is still strong like this.

I2: Chabwino. ndiye nangano ndizinthu ziti mwina zimene tingafune kuti mwina kapena timaona kuti mwina a Tiyanjane samakwanitsabe penapake zimene tingafune kuti mwina ata…

Alright. So what about the things that maybe we would want maybe or what we see that the Tiyanjane does not fulfill that which we would want them to maybe…

I: Atafikira ngati pamenepo.

If they reach that point.

I2: Eee atafikira?

Yes, if they could reach that point?

03/01: Ine apopo sikuti ndulamulayi komano at least patakhala kuti mwina ngati a Tiyanjane aja mwina apeza ndalama adziyesesa kuti mwina kaya mkatikati mmawa, anthu amene mwina anaalembera date ngati yammawa mwina makapita muja aliyense mwina ndi kathumba ka soya kuti atleast mwina patient'yo ngati ndalama mwina amazipeza movutikira ka soya kaya ndi ka 5kg kakamuthandiza ndithu week imeneyo kuti adzikadya adzikhala ndi mphamvu ndiye tingowalimbikitsa kuti funding itakhala kuti ilipo, eee funding itakhala kuti ilipo atha okha iwowo mwina kuona kuti aaa apo patient uyo atleast ka 5kg eni aka ka soya, ka soya. Ndithu zingakhalireko bwino kuti patient uja pofikira akapapeze chifukwa mwina patient mwina popita mwina ku Tiyanjaneko mwina wangonyamuka mwina mmimba mwina mulibe chinachilichonse ee malingana ndi momwe ndalama yavutiramu koma amakachoka kumenekoko ndi ka 5kg kaya ndi ka soya kaya ndi ka nyemba tingoti chakudya chinachilichonse chomwe chingamuthandize zingathandizire kwambiri.

Am I am not being autocratic but **atleast** if the Tiyanjane might fins money maybe like in the midst, tomorrow, for those people who maybe they were given tomorrows date maybe a bag of soya for each when coming back so that **atleast** if it is hard for the patient to find money that soya either a 5kg can assist that week for the patient to be eating and be strong so we are just encouraging them if funding is there, yes if there can be funding they can be able to see themselves that aaa this patient, here is **atleast** a 5kg of soya. It can be good the patient will find somewhere to start with because maybe the patient did went to Tiyanjane on an empty stomach yes because of the problems of money but if is leaving there with a 5kg of either soya or beans we should just say any food that can assist him/her that can be of a great assistance.

07/01: Komanso poyamba timalandira jackson ndiye panopa jacksoniyo kulibe akanakhalabe kunena kuti akupezeka kumakalandira zikanachitanso bwino.

And at first we would also be receiving injection but there is no injection now had it been that it is available and we would b e receiving that would be very good,

I2: Mm mm.

Mm mm.

I: Chabwino.

Alright

09/01: Apo ndinenepo pang'ono eti ineyo pafunso limene atifunsa adokotala pamatenda anga akhansa ine ndinachira ndipo simmene ndinaliri iyayi mwachitsanzo apa mmh mukuona apa apapa ndikuoneka bwino ndipo ndikuyenda ndiye ndikupempha kuti ngati mankhwalawa alipo kumenekoko kunjako atithandizireko madokotala athuwa kuti madokotalawonso athe kumatithandiziranso ife tomwe.

I should talk on that a little bit on the question by the doctor, my cancer desease got healed and am not the way I was like for example mmh you can see here I am looking good and am able to walk so am asking that if there is really medication out side Malawi they should assist our doctors so that the doctors they should also assist us.

I2: Mmm mankhwala aaaa?

Mmm, what medicine?

09/01: Aa jackson asasiyeyi, adzipezeka.

Aaa the should not stop the injection, it should be available.

I2: kaya ena...(overlaps)

What about the others…(overlaps)

09/01: Chifukwa ndi jackson ndikutha mmm ndikutha kuona kuti ndikuyenda bwinobwino.

Because I can see that…mmm I can see that am able t walk properly because of the injection.

I2: Mmm

Mmm

I: Mmm kwambiri.

Mmm, very much

09/01: Ndimatha kumayenda nkumagwa, kumayenda nkumagwa, kumayenda kumagwa masiku ano ndimatha kumayenda bwinobwino.

I would be walking and falling down like that, walking and falling down but I do walk properly these days.

I2: Ena:

Some other.

08/01: Mmm nkhani ndingoikira ndemanga zimene anena akuluwa zokhuzapo kunena kuti pamalangizo okhuza mankhwala amafuna kuti uyambe wadya pambuyo pake umwe mankhwala ndiye sionse amene ali ndi zokwanira pali ena atha kupezeka ndi vuto limenelo zakudya alibe kumakhala kuti munthu uja alephera kuti ndimwe mankhwala kupezeka kuti alibe chakudya kuti amwe mankhwala koma kutakhala kuti kwapezeka thandizo ngati mmene ananenera akufuna kwabwino nathandiza a Tiyanjane program imeneyo itakhala ndikukhulupirira kuti zitha kuchita bwino. China nchonena kuti aaa mmene amapereka thandizo a Tiyanjane poti iwo ali ndi luso ndi nzeru athe kuonjezera kupemphanso anthu ena mabungwe ena kuti pathe kuonjezereka, kuti jackson asamakhale kuti akusowa pali zambirinso zimene ifeyo sitingathe kudziwa kuti pali njira zanji zimene zingathe kupezeka popeza nthenda imeneyi imakhala kuti, nthenda iliyonse imakhala ndi zifukwa zake zimene zimafunikira kuti munthu athe kukhala bwino nde iwowo ndi amene amatha kudziwa kuti tikatere pafunika zakuti pafunika zakuti, chifukwa chochitenga chimenechocho nkuchipereka paiwo okha sangathe kukwanitsa a Tiyanjane koma alinso nkolowera monga ngati kholo kapenanso mwina ndi mwina angatitengere uthenga umenewu ndikutitumizira kwa anthu amene angathe kutero kuti awathandizire chifukwa chakuchepekedwa kwawo mwina kusapereka zimenezozo sikufuna kwawo zimenezo amakhala mmaganizo amakhala nazo koma chifukwa chakuchepekedwa kwao koma mwa ifeyo kudzera mwa ifeyo tikupereka pempho limenelili kuti lithe kufika dziko lonse lapansi kwa atsogoleri a maiko ngakhale a mabungwe kuti atumize zimenezo kunonso kuli anthu amene akuvutika akumwa mankhwala pafunika zakudya akumwa mankhwala afuna zosiyanasiyana ngakhale mankhwala amene mwinanso tikamamva kunena kuti kuli mankhwala ena amene amatha kupezeka kunena kuti atha kulandira munthu nkupezeka kuti nkuthandizika amenewo atawaonjezera a Tiyanjane kuti chikondi chawo uphungu wawo, luso lawo likawonekere lopambana kwa ife Malawi akakhale wabwino nthawi zonse.

Mmm, the issue is, let me just add on what this gentleman has said about the medicine counsel, they want you to eat first, then take medicine afterwards. Its not all who have enough some can be found with the same problem but they don’t have food, they would want to take medicine but, “I should take medicine,” but finding out that the person doesnt have food to take the medicine but if assistance can be found and the well wishers assist the Tiyanjane I feel that if that program can be there it can be good. The other things is that aaa the way Tiyanjane gives assistance, since they have the skills and wisdom they should add and ask the people from other organizations for the additional…so that the injection should be always available because we cannot know what ways can be there since each disease has its own, each disease has its own reasons necessary for a person to be fine so they are the ones who knows that, “if we do this and that these necessities will be needed.”By taking that responsibility and giving it, the Tiyanjane cannot manage on their own but they have somewhere where they go as a parent and also maybe they can take the message to other people who can assist because of their lack it might be possible that they do have that in their mind but its not there wish to not provide that but its because of their lack so through us we are giving this plea to reach out to everyone in the whole world, the national leaders even the organizations to send those that there are people who are in trouble they are taking medicine but they are is a need for food, they are taking medicine but they need different things and even the medicine itself maybe when we are hearing that there are some medicine of which a person does take and is assisted the Tiyanjane should add that kind of medicine so that there love, their counsel, their skills should be seen as being great to us and Malawi should be good all the time.

I2: Mm mm. Aliponso wina amene akufuna kuonjezera.

Mm mm. Is there anyone else who wants to add?

06/01: Ine ndilankhulepo pang'ono, ndimamva anthu ena akadwala khansa amati akupita kunja kumeneko kukalandira thandizo ndiye kaya sindimadziwa kuti kaya amachiliratu chifukwa sindinamuone ndimangomva kuti kunja nkumene kuli thandizo amachira anthu akuti nde iwowonso akanakhala kutenga thandizo limene anthu amachira, amachoka kuno kupita kumeneko kukawapatsa ndikuchira anakatenganso step yokathandizira a ku Tiyanjane kuti thandizo limeneli lizifika mma Tiyanjanemu kuti nafenso adzitithandiza chimodzimodzi kuti nafe tisa, nanga siife sitingafikire kupita kunjako kaya kuti kukasaka thandizolo kulibwino anthu ngati amenewo atatengako gawo kubweretsa mankhwala kuti ku Tiyanjane kuti tonsefe titani, atifikire.

I should talk a little bit I here that some people goes outside Malawi when they are sick and they receive assistance there so I do not know whether they become cured since I have never seen one but I just hear that there is assistance outside and people gets cured so if they could also get that assistance which makes people get cured, they leave here going outside and are given and get healed had it been that they also took that step to assist the Tiyanjane so that this assistance should be in the Tiyanjane’s so that we should also be assisted in the same way so that we should not, since we cannot manage to go outside to seek for that assistance its better that people like those should take part and bring the medication to Tiyanjane so that it reaches all of us.

I2: Mwina pongoonjezera pazimene mwakamba kalezi timakhulupirira kuti chinachilichonse chili ndi ubwino ndi kuipa kwake eti mmm, kaya mwina ndizinthu ziti zina zimene a Tiyanjane kaya amachita kaya zimachitika inuyo sizikusangalatsani kaya ndingafunse motero.

So maybe just to add on what you have already said, we believe that everything has its advantages and disadvantages right, mmm so maybe what are the things that the Tiyanjane does or happens but it does not make you happy, maybe I should ask like that.

07/01: Mmm tingonena kuti nthawi imeneyoyo timabayidwa jacksoni ngati asemphanitsa jackson munthu sizimakhala bwino eee ngati sapita mmisempha muja monga tiyerekeze ndekuti dzalazi zimazachita zazi mpakana iziz zitatuzi (xxx)

Mmm we should just say that that time we were given injection so it wasn’t good if they have given you another injection instead of another yes if it will not pass through the muscles for example then these 3 fingers were becoming numb (xxx)

I2: Mmm, mumalankhulatu.

Mmm, you were talking.

08/01: Mmm ndimalankhula chomchi, tidziwabe ndilankhula kumbali popeza kuti chibadidwe kusiyana zimatheka kufika ku Tiyanjane kukumana ndi dokotala mwina chifukwa chokuti monga odwala wachita zosemphana ndi maganizo a dokotala zimatheka madokotala amatha kumukalipira patient monga ngati zinthu ngati zimenezozo monga a Tiyanjane chonde mukakumana muma meeting anu langizanani kunena kuti patient amatha kunena kuti akumva kuwawa mwina tsiku limenelo sanadzuke bwino mwinanso ndi chibadwidwe chake athe kuchitapo kanthu, patient ndithudi nthawi zina zimapezeka kuti ndikhulupirira kuti tsiku lina lake koyamba kwanga kupita kukalandira mankhwala ku kodi kaya ku 4b kaya 4 a kunapezeka kunena kuti ndinapatsidwa mankhwala amtundu umodzi koma anandilembera mankhwala awiri ndiye mnangoti aaa ndalandira basi koma ntazabwereranso wina wake ntakampeza anandiuza kuti aa mwalandira amodzi okha chifukwa chani anatenga mankhwala ena aja nkutani, nkundipatsa pempho limene lingafike ku, kopereka mankhwala adzitha kuonetsetsa kuti alemba chani chifukwa kumapezeka kunena kuti munthu ukuyamba kumene sukudziwa kunena kuti ndi mankhwala anji ndi mankhwala anji ndipo nanga dokotala akulemba bwanji komanso ku Malawi kwathu kuno sionse amene angathe kuwerenga athe kumuthandiza patient moyenerera kunena kuti athe kulandira mankhwala ake moyenerera.

Mmm, I was saying like this, I know I will talk here aside since we we have different characters it happens to go and meet the doctor at Tiyanjane and maybe the patient has acted in a different way to the doctor’s thoughts happens to shout at the **patient** things like those please you Tiyanjane people advise each other when you have your meetings that the patient happen to say that he did not work up well that day or maybe that’s his character they should do something, it really happens with a **patient** that, I believe that this other day, my first time to receive medicine from is it 4b it was found that I was only given one type of medication but I was supposed to get 2 as prescribed for me so injust said that aa I have got is thats all but when I came back a certain person asked me why I have only received one and took the medicine and gave it to me, my plea is that the pharmacy people should be making sure to see what it has been written its found that maybe its your first time and you don’t know the types of medicines and what the doctor has wrote and also its not all who can read here in Malawi they should assist the patient properly so that he can receive his medication properly.

I2: Mm mmh. Kaya ena tikuti bwanji, azimayi?

Mm mmh. What are the others saying, ladies?

06/01: Nkhani ndi yomweyo kumvetsetsana ndiye kumafunika chifukwa nthawi zinanso munthu ukadwala umapanga zinthu zosokonekera umakhala ndi ukali mwina munthu akuchitira zabwino iwe koma ukapanga za, ndinaonapo patient wina wake iii munthu akumu, akumuyendetsa panjinga paja kuti akamufikitse pamalo poti aklandire mankhwala koma iyeyo zomwe akulankhula zambirimbiri eee ukundigunyuza ee mwakuti nde dokotala pena akakhala kuti watopa nayo ntchito amatha kukuthandiza maganizo ako kuyamba kukukalipira ee iwe munthu siiwe wekhatu oti ndikuthandize iyayi alipo amnzako akundidikiranso ine mwakutimwakuti zimene zijanso zimakhala zotani zosemphana awirinu, dokotala ndi patient. Mapetient amakhala a ukali mukuyenera kuvala mtima opirira madokotala munapirira kale munaphunzitsidwa kale komabe mtima umenewo musausiye mupirirebe chifukwa madokotalawo okalipa sanathe alipo ena amangotukwana kutukwanatu zosadziwika zija ine ndakumana nawo zinthungati zimenezizi muku, mukuyenera kuwamvetsetsakungopewa kuti ii sitikudziwa kuti uyuyu akukumana ndi zotani munkhondo yake yamatendawa.

That is the issue and listening to each other is what is most needed because sometimes when a person is sick you do some wrong things, you are like very rough while a person is doing good things to you but when you do, I saw this other patient iii you are, is being pushed on the wheel chair to get him where he is suposed to receive medicine but what he is saying, he is talking a lot, ee you are arousing me etc so sometimes the doctor helps your thoughts because of being tired with the work and starts shouting at you, ‘ee you are not the only person am suppose to help there are your friend also who are waiting for me and so on and so on,”those are also disagreements between you two, the doctor and the patient. You are supposed to wear an enduring heart because the patients are rough, you doctors have already endured you were taught already but you should not stop but keep the enduring heart still because there are still doctors who shouts and some just insults without reasons I have met those things, you are supposed to understand and avoid that, “iii we do not know what this one is facing in the his diseases battle.

I2: Mmm, otukwanawo ku Tiyanjane komweko?

Mmm, the insulting ones are also at Tiyanjane?

06/01: Iii mnaapeza ine ku phamarcy ali panjinga koma munthu akutukwana zimene zijanso zimatha kupezekanso kuti mwina zapita ku Tiyanjaneko sitikuti tizichita dala iyayi komano pali, pali ena sindikudziwa kuti, kuti kaya zimakhala kuti matenda atani kaya zakhala bwanji amapezeka kuti ali ndi ukali dokotala akukuuza zabwino iweyo mmalo moti uyankhe bwinobwino umapezeka kuti dokotala uja ukumuyankha ngati kuti simunthu, ndiye dokotala amakumana ndi zinthu zambiri amathandiza anthu ambiri amakhala kuti watopa ndiye nayenso ndikutopa kwake amangoonjezeranso zake pompo ndiye zimenezijazo zimakhalanso zinthu zotani, zosemphana ndithu.

Iii I found them at the pharmacy while on a wheel chair but the person was insulting those things might happen to get to Tiyanjane also we are not saying that we will be doing that intentionally but there is, there are some I do not know what, I don’t know what happens to the disease or what has happened but they are found of being rough, the doctor is telling you good things other than you responding politely you are found responding to thedoctor as if he/she is not a human being so the doctor meets a lot of things, assist a lot of people, happen to be tired also so with his tiredness he just add on that, so those things happen to be what, different things.

I2: Mmm, kaya alipo amene akufunanso kulankhula chinachake pomalizitsa kukambirana kwathuku, zonse zimene takamba kaya kumalizitsa.

Mmm, Is there anyone who wants to talk also in concluding our discussions, all that we have discussed or finishing off.

I: Khala ngati palibe eti?

It seems that there isn’t right?

All: Mmm

Mmm

I: Chabwino ifeyo tathokoza chifukwa cha zokambirana zathu zimene tinali nazo koma sitinamilize kwenikweni....(interruptions)

Alright, we are very thankful because of the discussions that we had but before we completely finish…(interruptions)
