# Supplementary material for: Household concepts of wellbeing and the contribution of palliative care in the context of advanced cancer: A Photovoice study from Blantyre, Malawi
Source: PLoS One. 2018 Aug 22;13(8):e0202490. doi: 10.1371/journal.pone.0202490 (PMC6104988; doi:10.1371/journal.pone.0202490)
Supplement: S2 File — (DOCX) [file pone.0202490.s002.docx]

Appendices

Appendix A

**University of Liverpool School of Tropical Medicine Information Sheet**

**Participant Information Sheet : English version 1.0 (14/03/2016)**

**An exploration of household wellbeing and the contribution of palliative care following a diagnosis of advanced cancer : a Photovoice study**

1. **Introduction**

Palliative care has expanded in Malawi since it began in 2001, though many patients are still unable to get the help they need once they have an serious disease like cancer. Little is known about the impact on household wellbeing (including costs) of a diagnosis of advanced cancer in Malawi, though research from other low resource settings suggests that the experience is often associated with catastrophic expenditure. We are doing this study to explore experiences of living with/as a patient who has cancer to try to understand more about the challenges that you and your family members face.

1. **Why have you been chosen?**

You are known to be a members of a household with a patient who has a diagnosis of advanced cancer under the care of Tiyanjane Palliative care in Blantyre.

1. **Do I have to take part in the study?**

Your agreement to help with this research study is completely optional and if you would prefer not to participate, this will be without penalty or loss of care/benefits to which you would be otherwise entitled. You can choose to leave the study at any time, without providing a reason.

1. **What will be involved if I agree to take part in this study?**

We want to ask you to explore your experience of living with/as a patient who has advanced cancer. This will be done through the use of photographs. The study is composed of seven separate group sessions in Ndirande. As you attend these sessions you will be taught how to use cameras and then asked to take pictures in and around your home environment. This will take place over a period of three weeks. You will be asked to take photographs of aspects of your life which you think best tell the story of your experiences, both before and since having the diagnosis, what things matter most to you, what challenges you face day to day. Later on you will be asked to select photographs and then to discuss them with one of the team. We will record these discussions.

1. **Will there be any risks involved in the study?**

You will be provided with a working camera, SD card and batteries. As part of the training we will discuss with you how to use and handle the camera and how to keep it safe. If you don’t feel safe to keep the camera in your household overnight we will make arrangements for it to be collected and returned the same day.

1. **Will there be any benefits involved in being in the study?**

Return transport cost to the sessions will be provided, and you will receive refreshments during the group session. You will be provided with 20 printed copies of photographs of your choice at the end of the study. You will be able to display your photographs for exhibition to make key stakeholders at national level more aware of your situation.

1. **Who is organizing the study?**

The research is being done by researchers at the College of Medicine linked to the University of Liverpool, Liverpool School of Tropical Medicine

1. **Who will know what we find out?**

We will use digital voice recording machines to record group sessions and individual interviews and store this information on a computer. This information will be transferred to a computer database but without using your name or address so that you could not be identified from this information. This database will be analyzed by researchers at the College of Medicine and Liverpool School of Tropical Medicine. We will share the results of this study with you and your community, at local charity or research meetings and will present the findings at an international conference and in journals.

1. **What happens if you change your mind, move away or become too unwell to continue in the study?**

If you agree to join the study you can change your mind and withdraw your consent at any time. If you move away or become too unwell to continue, we will ask permission from you (or from your household member) if they are happy for us to include the photographs and any interview material with which you were involved.

If you have any questions about this study, please contact our Field work coordinator (details withheld)**.**

For any questions regarding participant rights in the scope of this study, please contact the Chairman of the local ethics committee (COMREC). This committee has reviewed and approved all of these studies. The contact details are: COMREC Secretariat, College of Medicine, P/bag 360, Blantyre 3. Tel no: **[+265 111 989 766].**
