# Supplementary figures and images for: Household concepts of wellbeing and the contribution of palliative care in the context of advanced cancer: A Photovoice study from Blantyre, Malawi
Source: PLoS One. 2018 Aug 22;13(8):e0202490. doi: 10.1371/journal.pone.0202490 (PMC6104988; doi:10.1371/journal.pone.0202490)

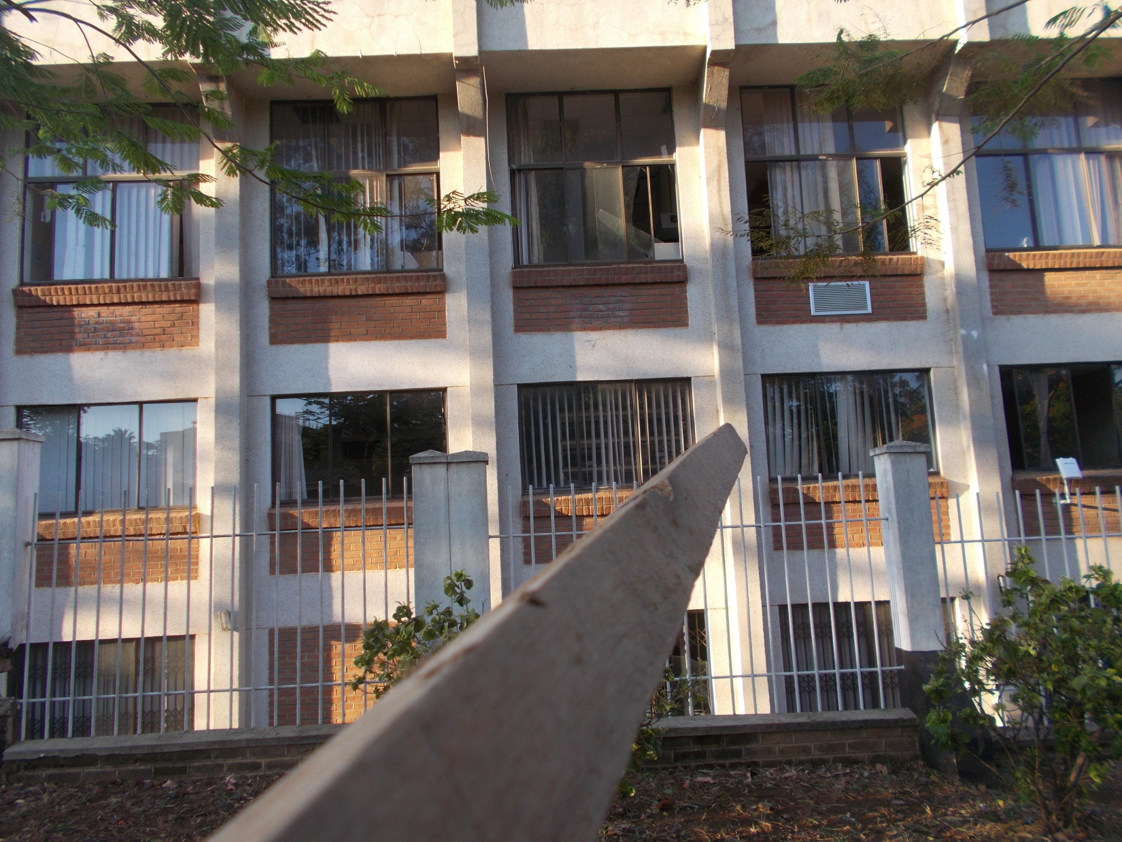

Supplement: S4 File — (ZIP) [file pone.0202490.s004.zip › example images/example1.tif]

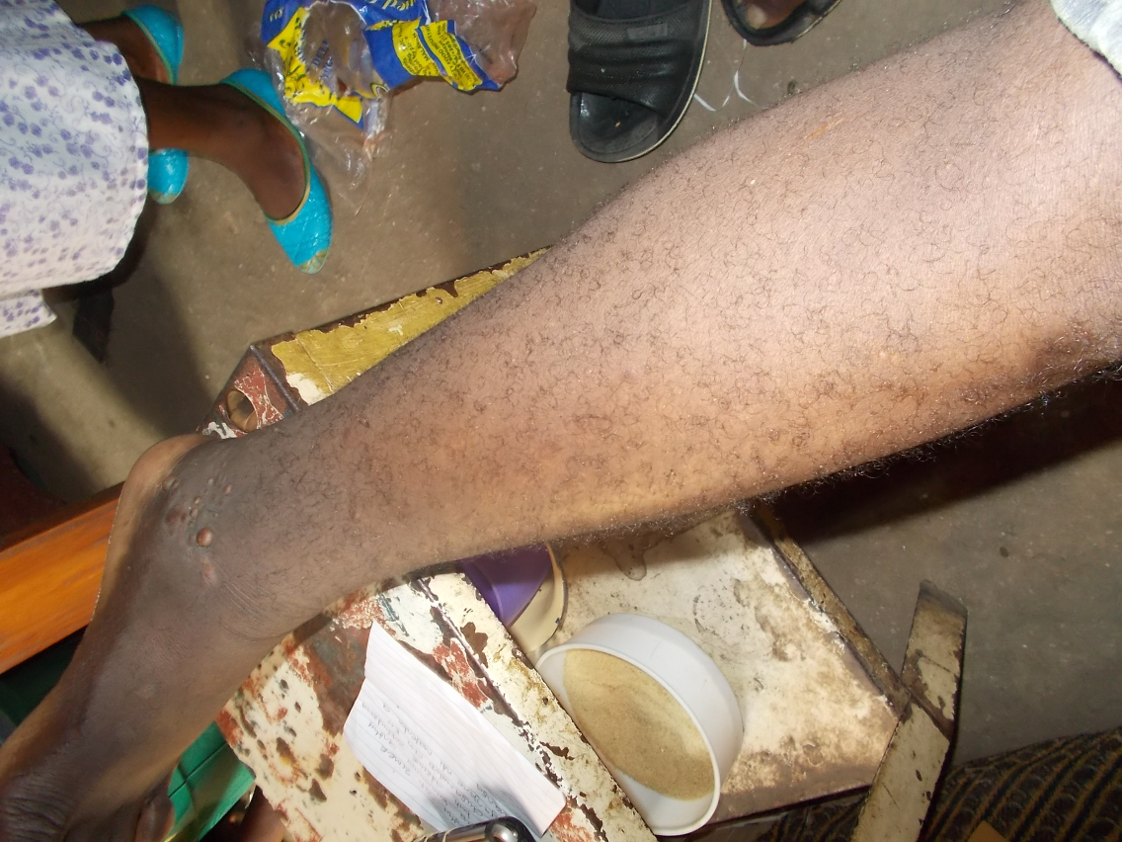

Supplement: S4 File — (ZIP) [file pone.0202490.s004.zip › example images/example10.tif]

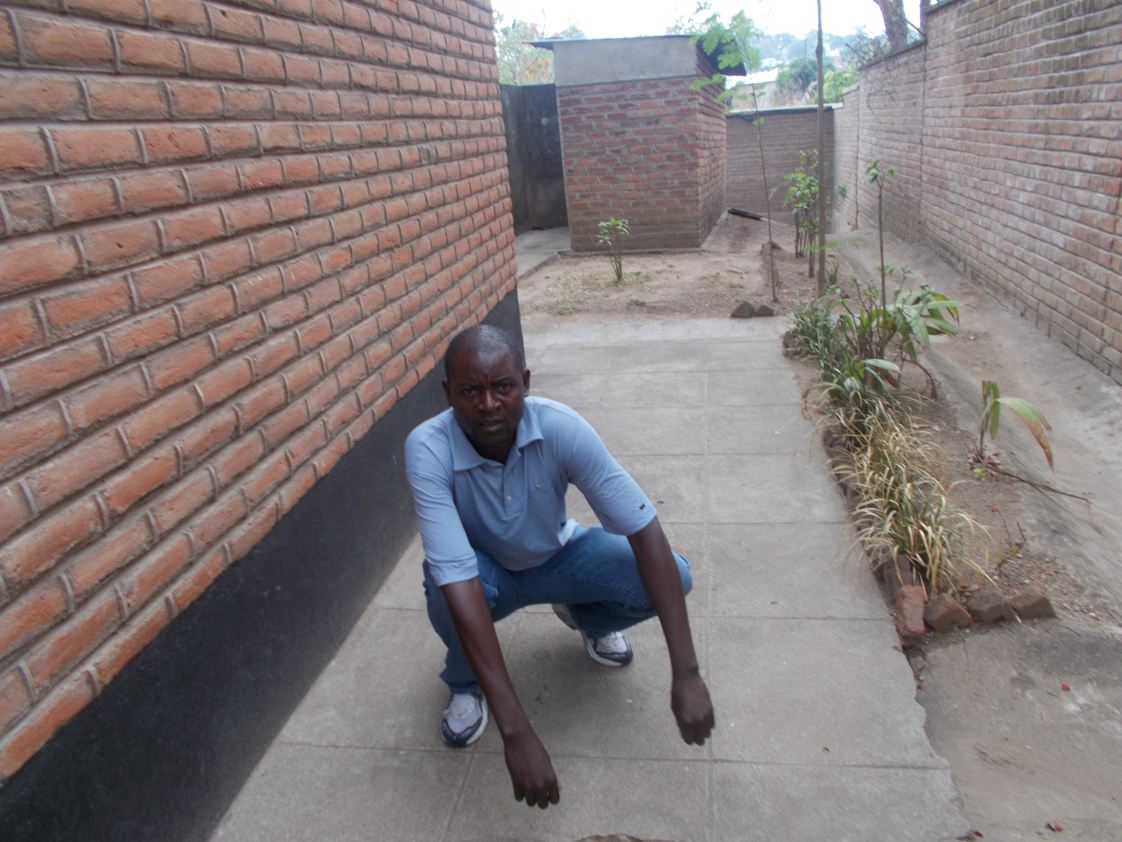

Supplement: S4 File — (ZIP) [file pone.0202490.s004.zip › example images/example11.tif]

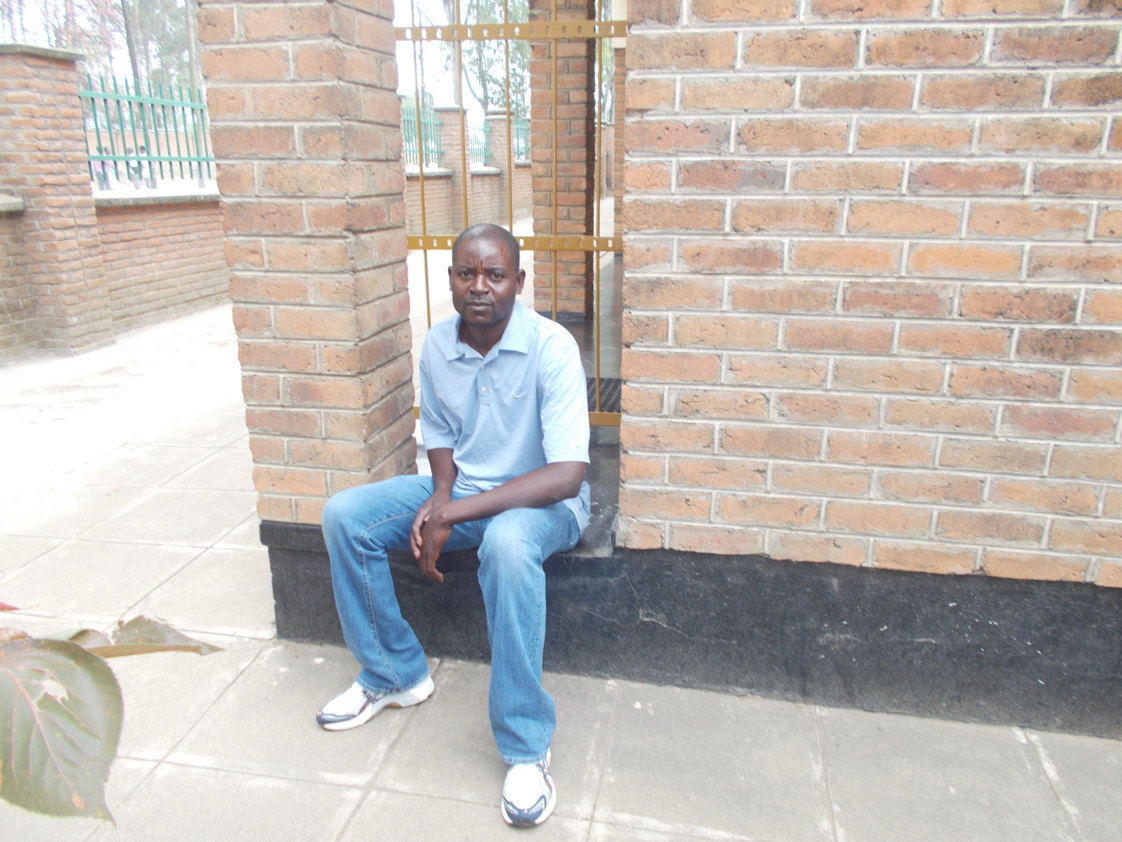

Supplement: S4 File — (ZIP) [file pone.0202490.s004.zip › example images/example12.tif]

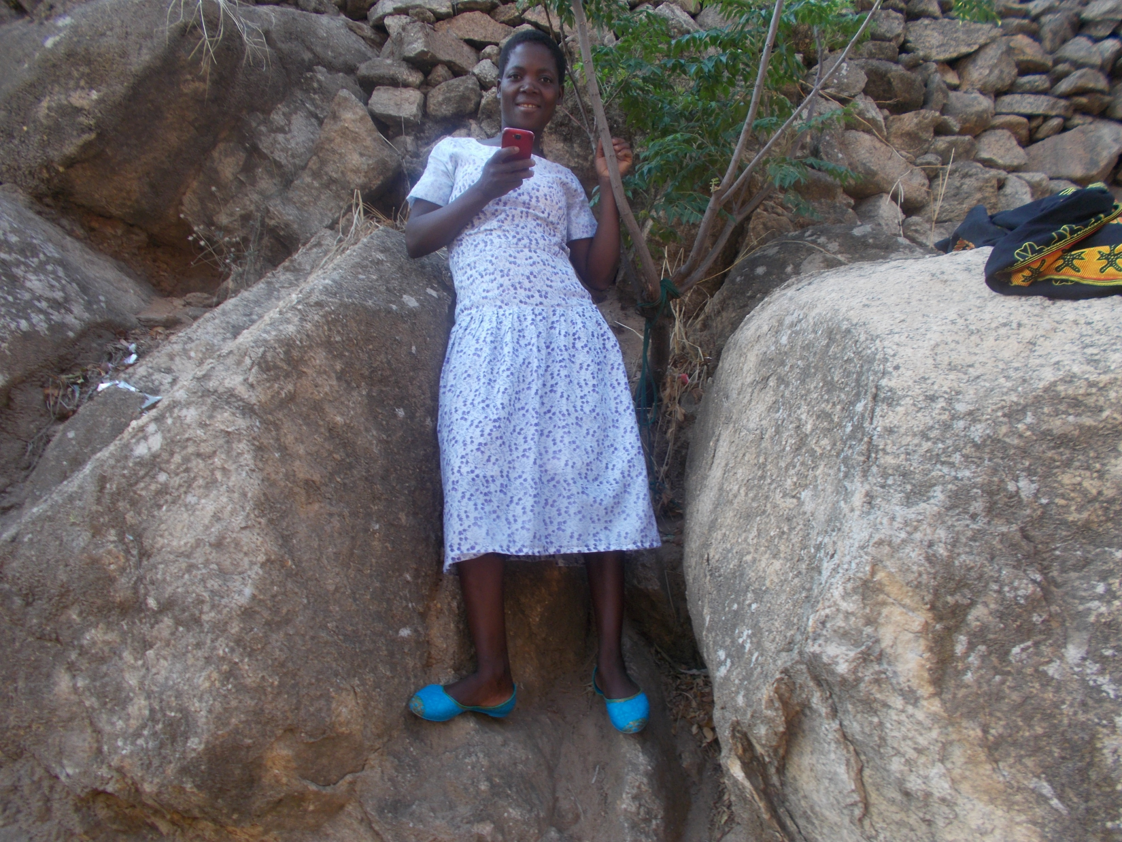

Supplement: S4 File — (ZIP) [file pone.0202490.s004.zip › example images/example13.tif]

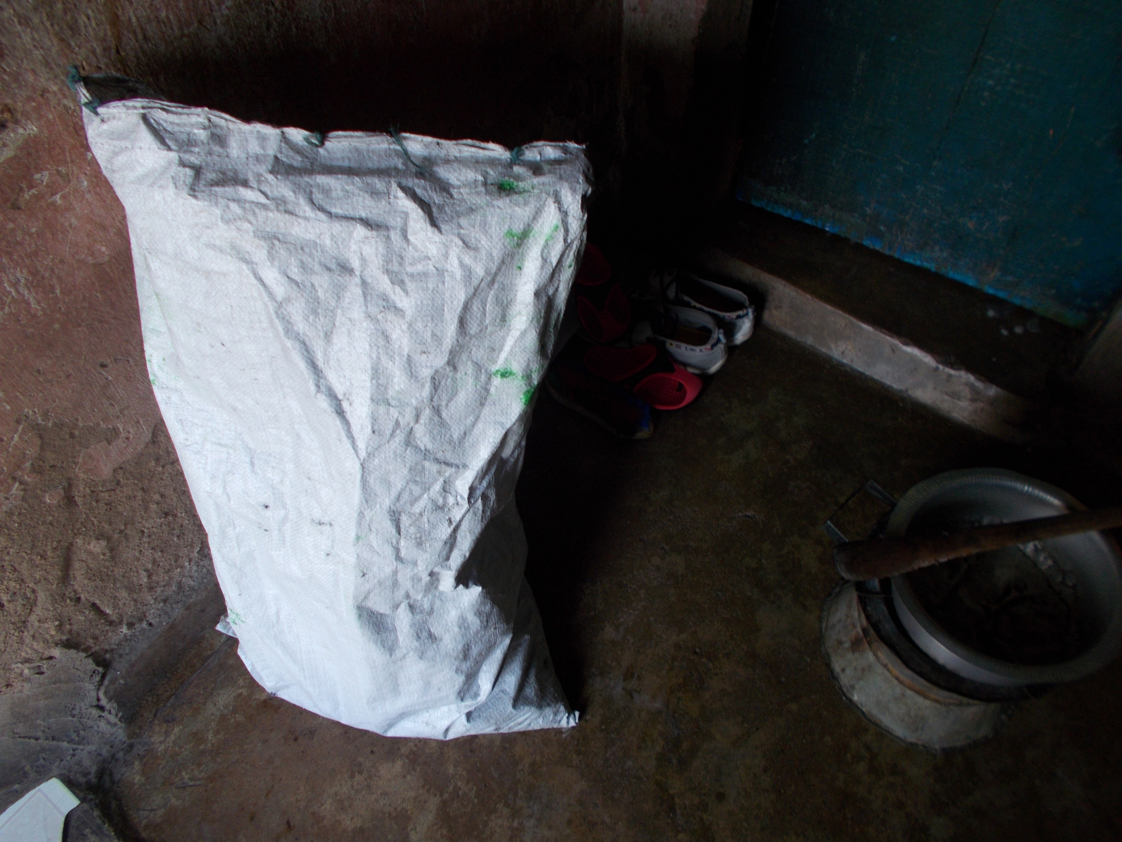

Supplement: S4 File — (ZIP) [file pone.0202490.s004.zip › example images/example14.tif]

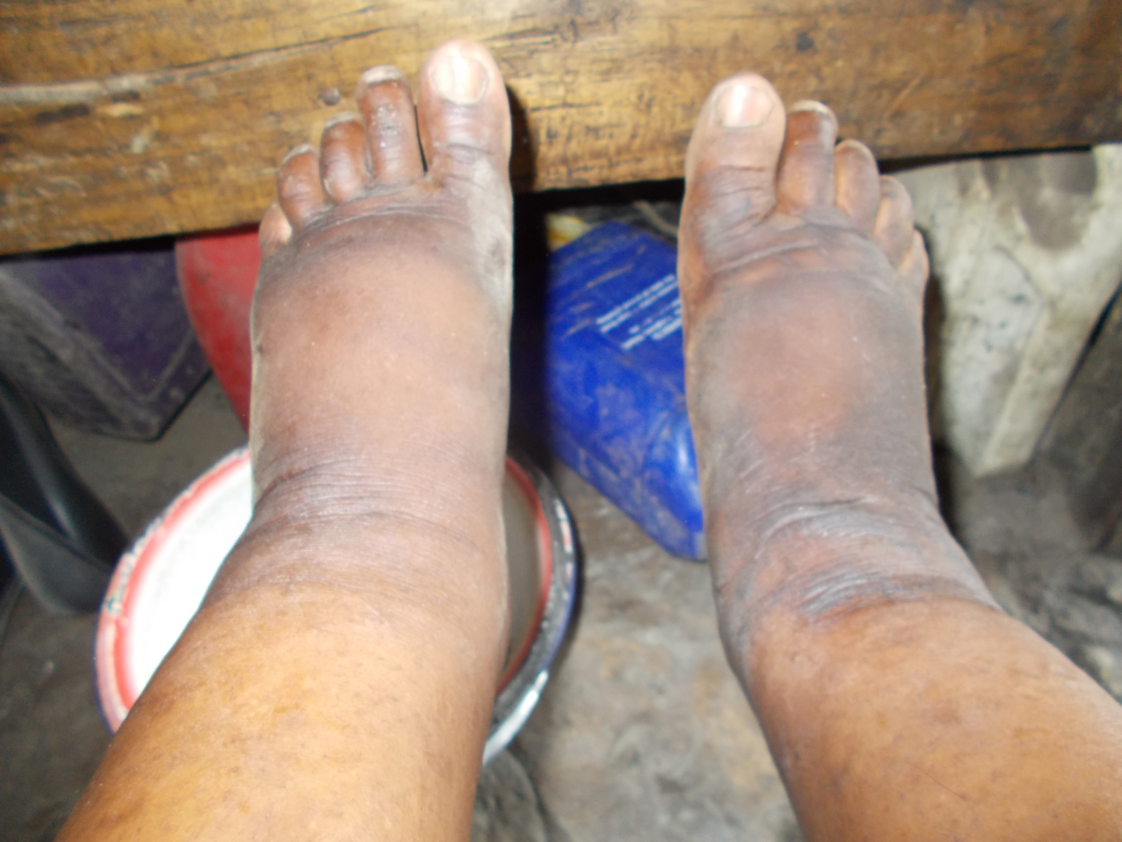

Supplement: S4 File — (ZIP) [file pone.0202490.s004.zip › example images/example15.tif]

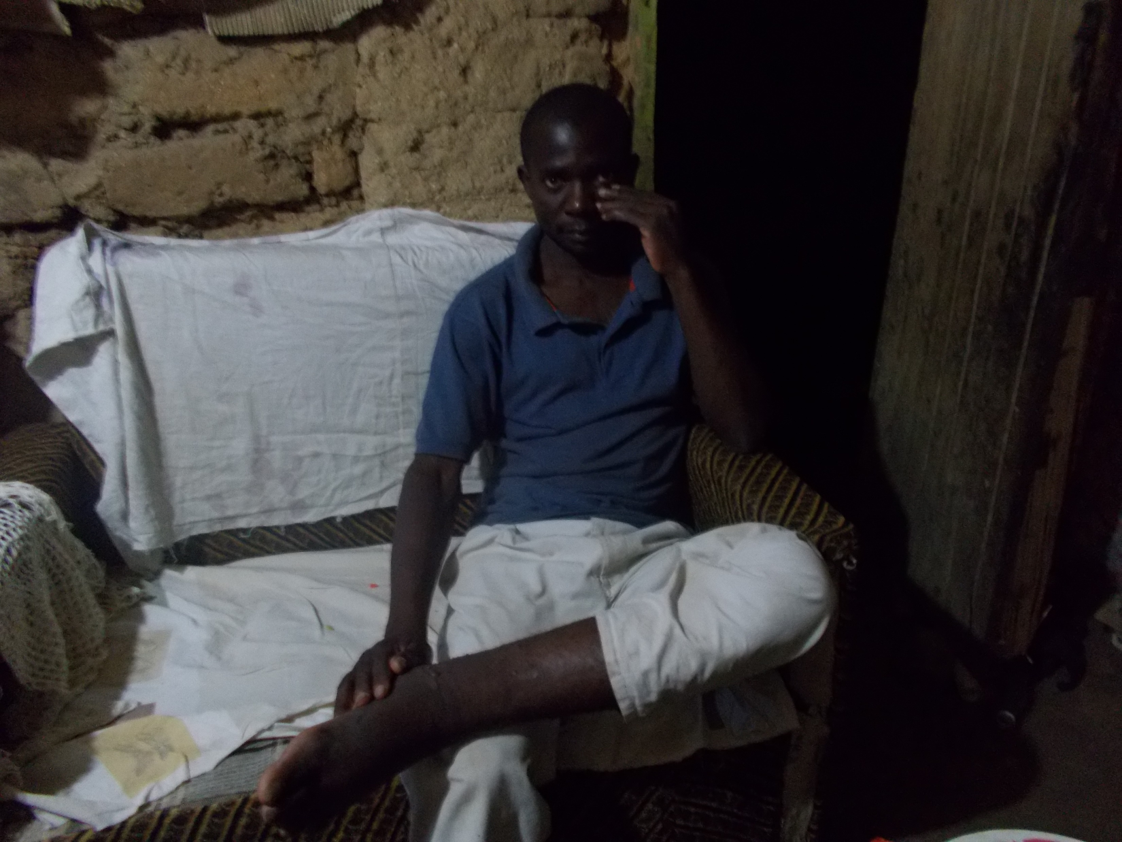

Supplement: S4 File — (ZIP) [file pone.0202490.s004.zip › example images/example16.tif]

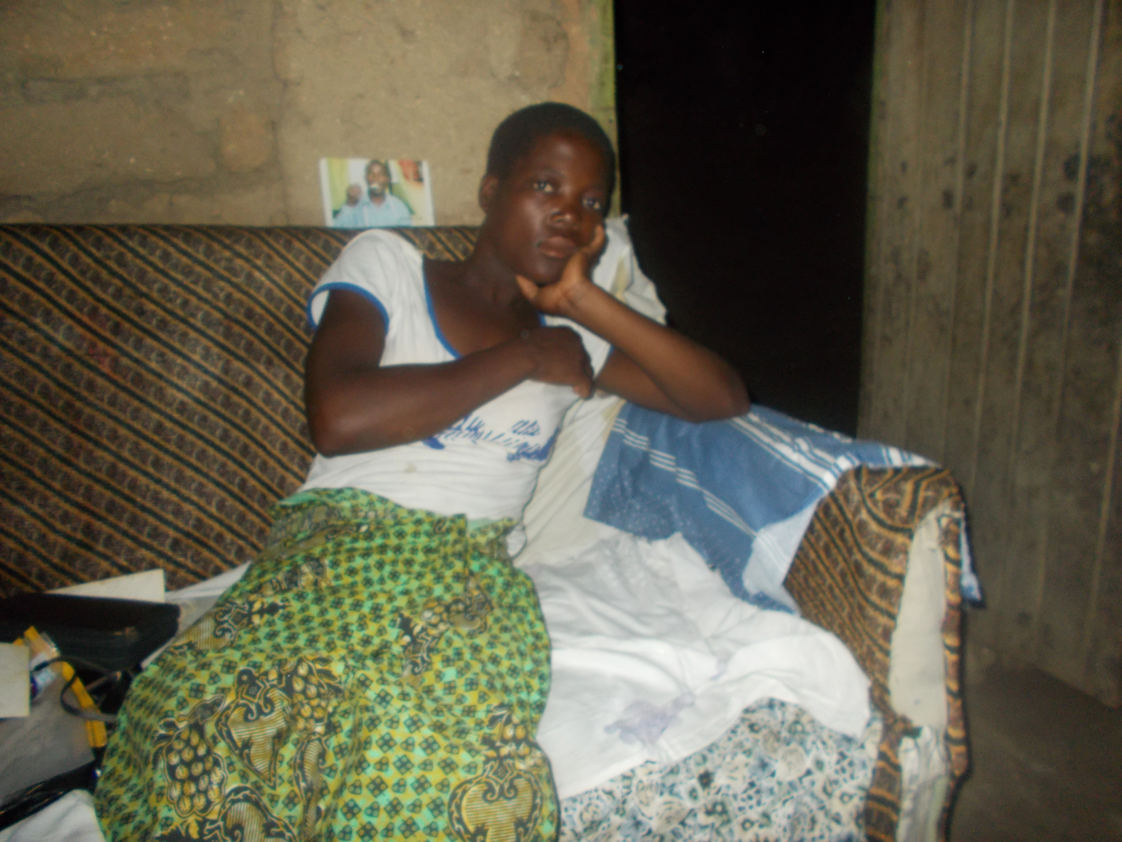

Supplement: S4 File — (ZIP) [file pone.0202490.s004.zip › example images/example18.tif]

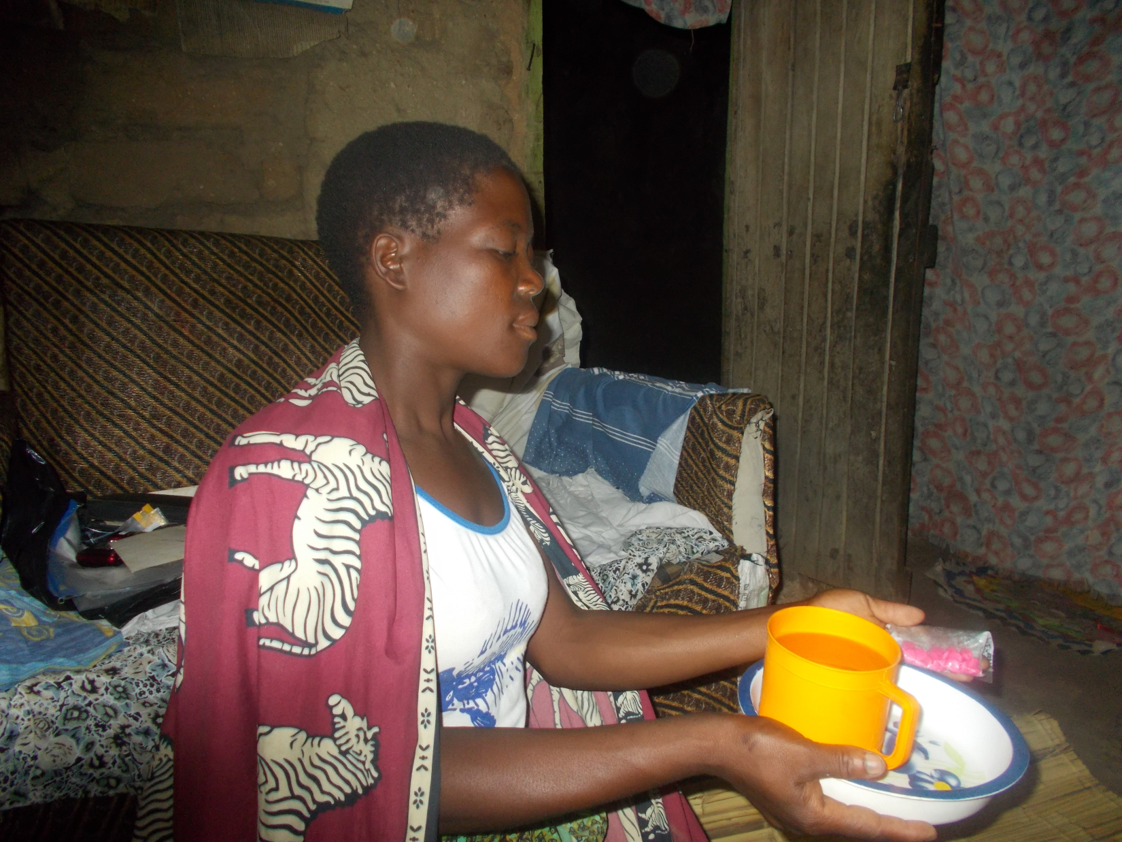

Supplement: S4 File — (ZIP) [file pone.0202490.s004.zip › example images/example19.tif]

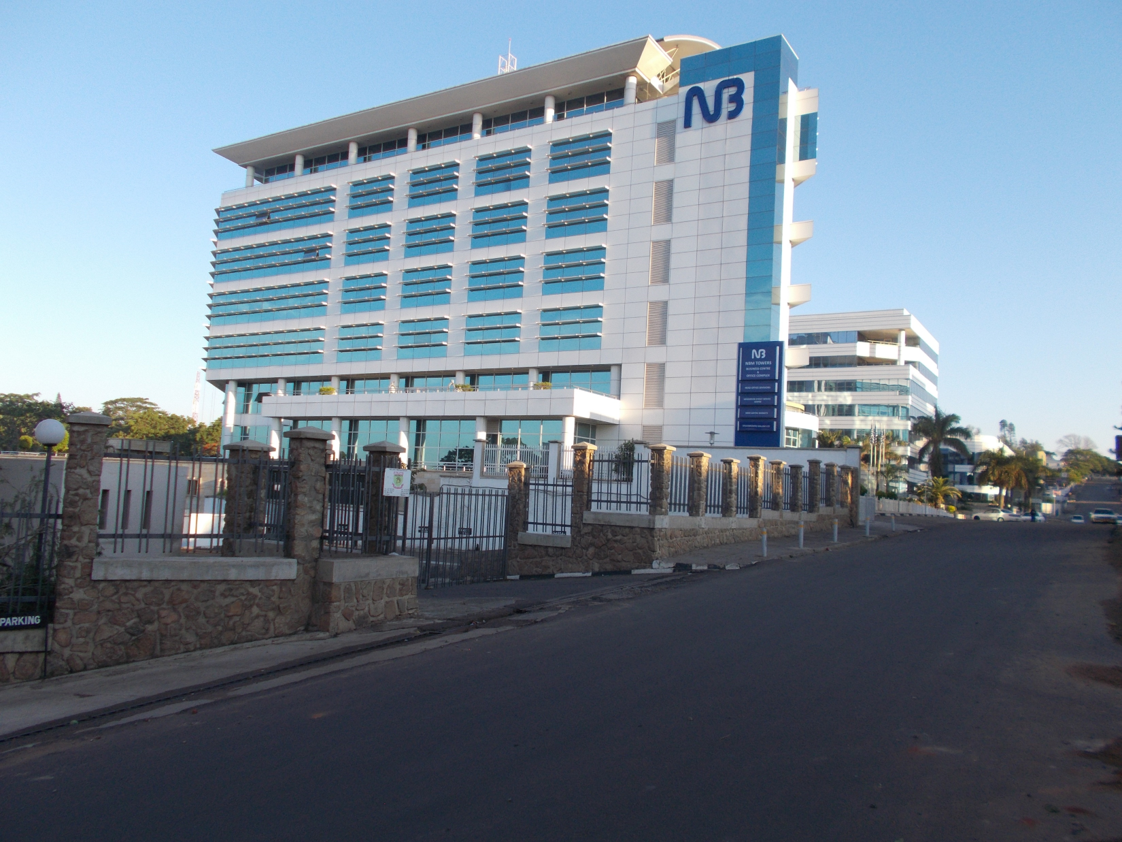

Supplement: S4 File — (ZIP) [file pone.0202490.s004.zip › example images/example2.tif]

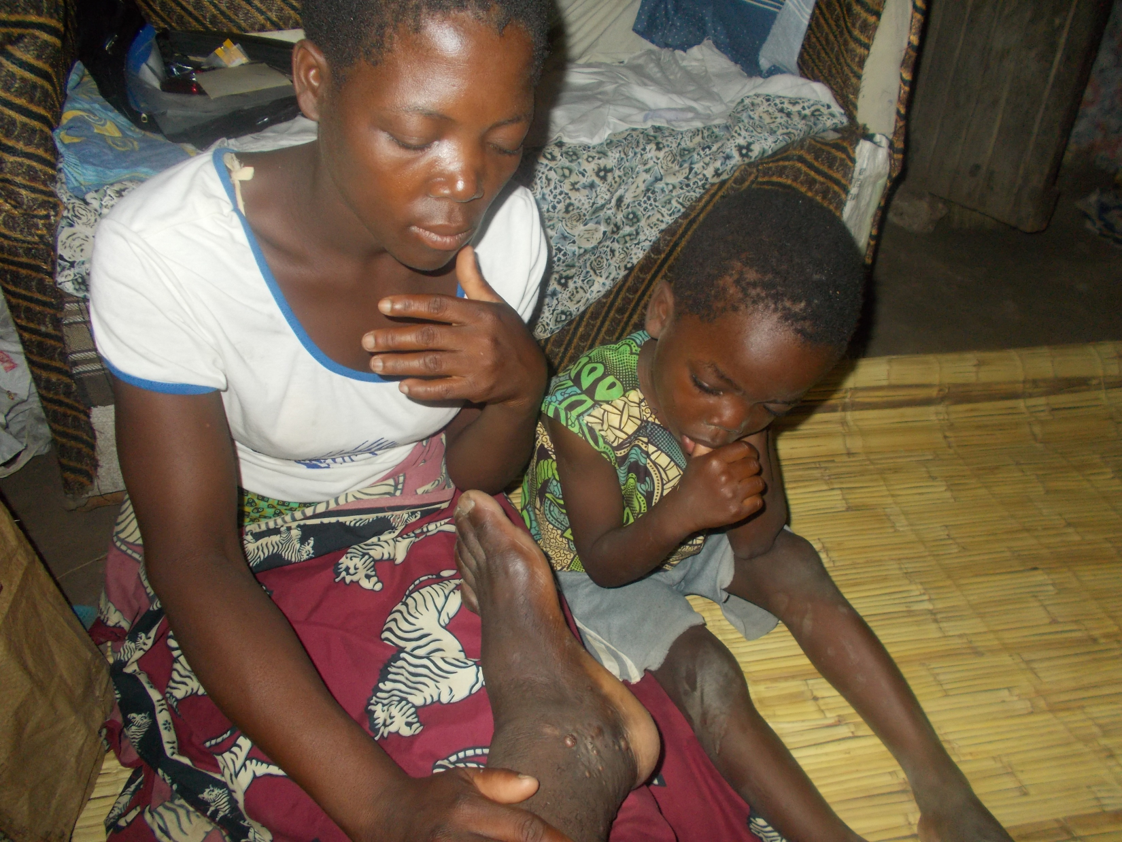

Supplement: S4 File — (ZIP) [file pone.0202490.s004.zip › example images/example20.tif]

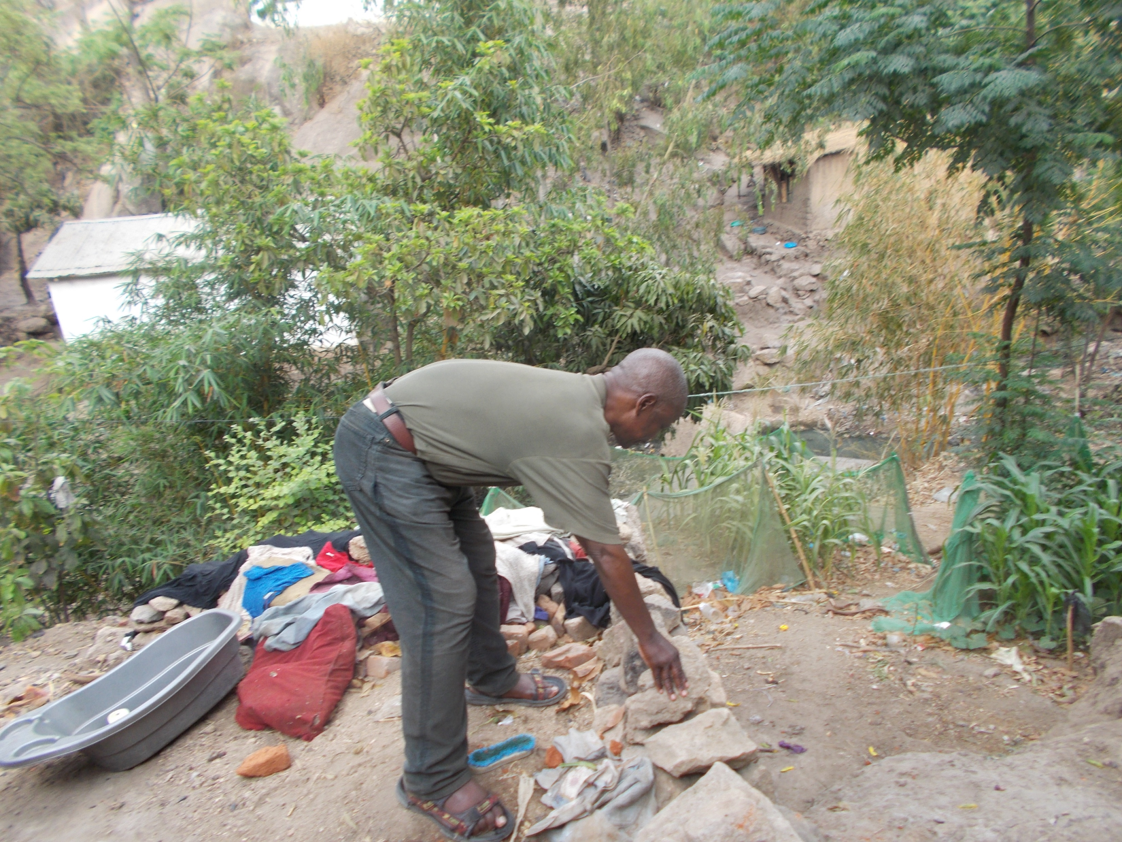

Supplement: S4 File — (ZIP) [file pone.0202490.s004.zip › example images/example21.tif]

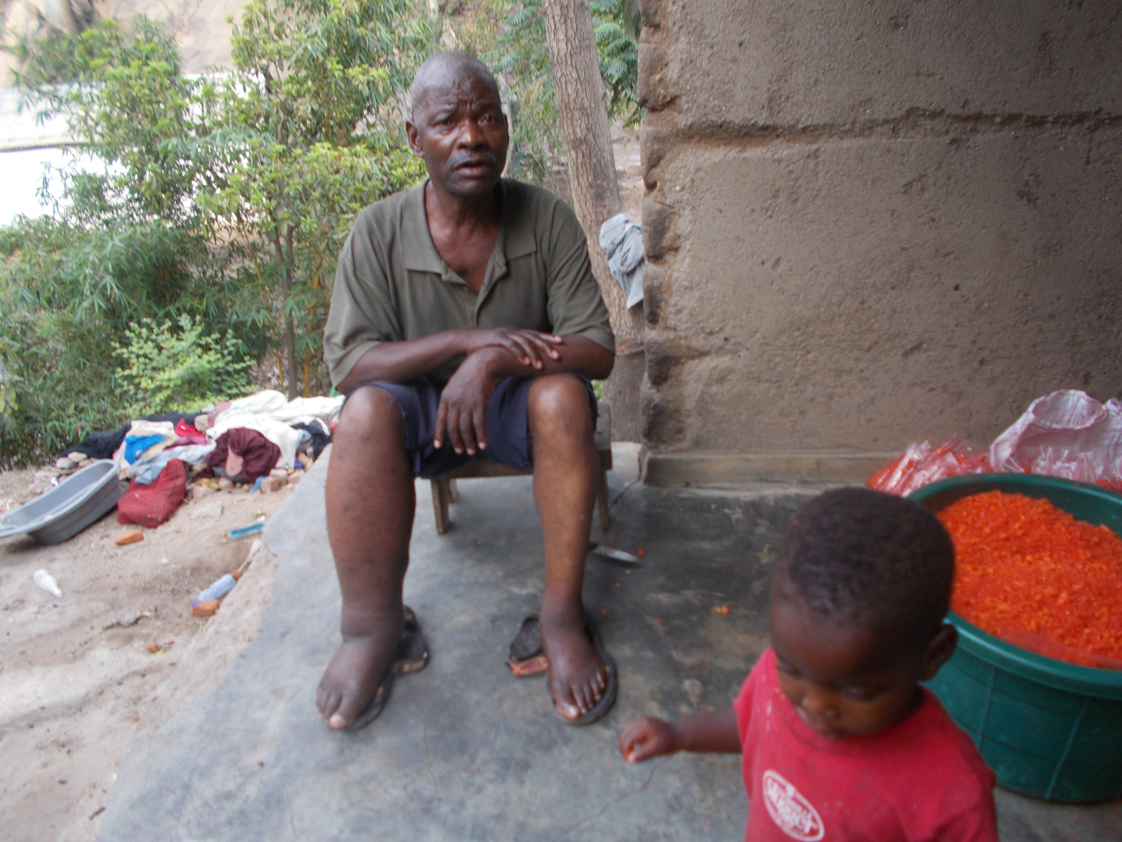

Supplement: S4 File — (ZIP) [file pone.0202490.s004.zip › example images/example22.tif]

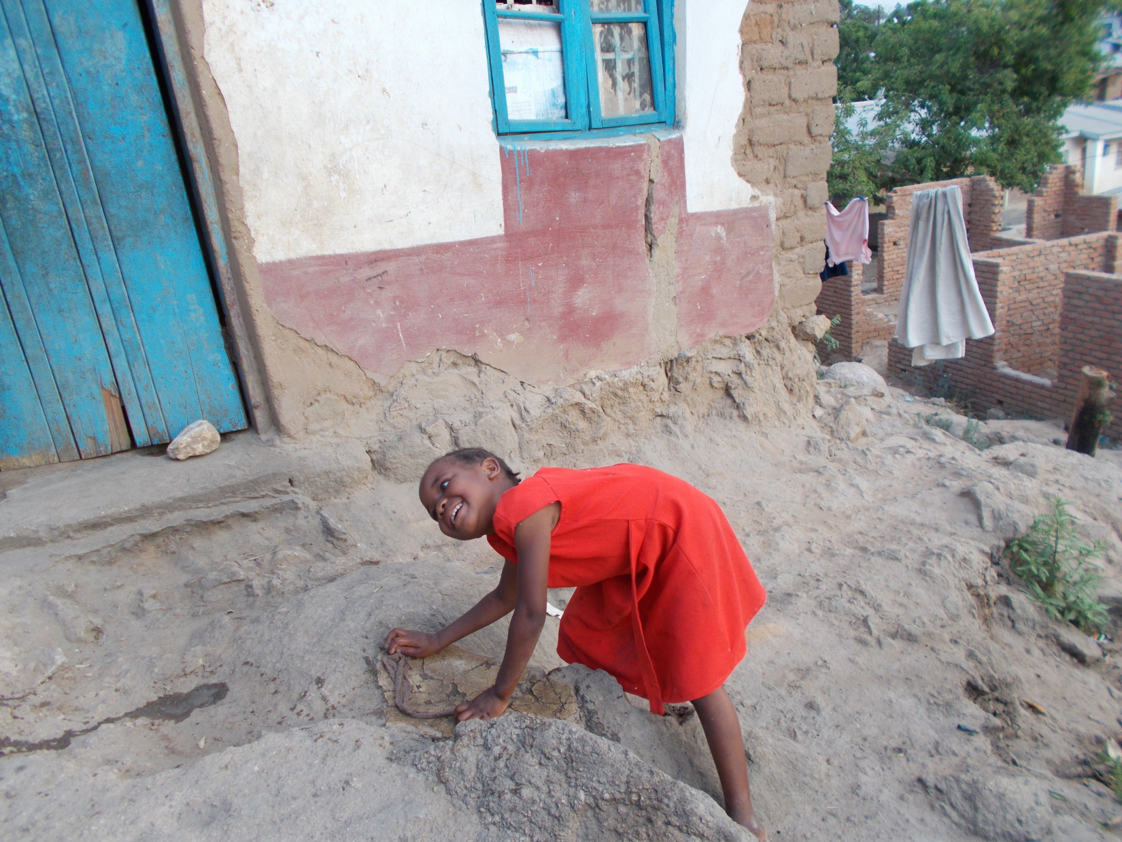

Supplement: S4 File — (ZIP) [file pone.0202490.s004.zip › example images/example23.tif]

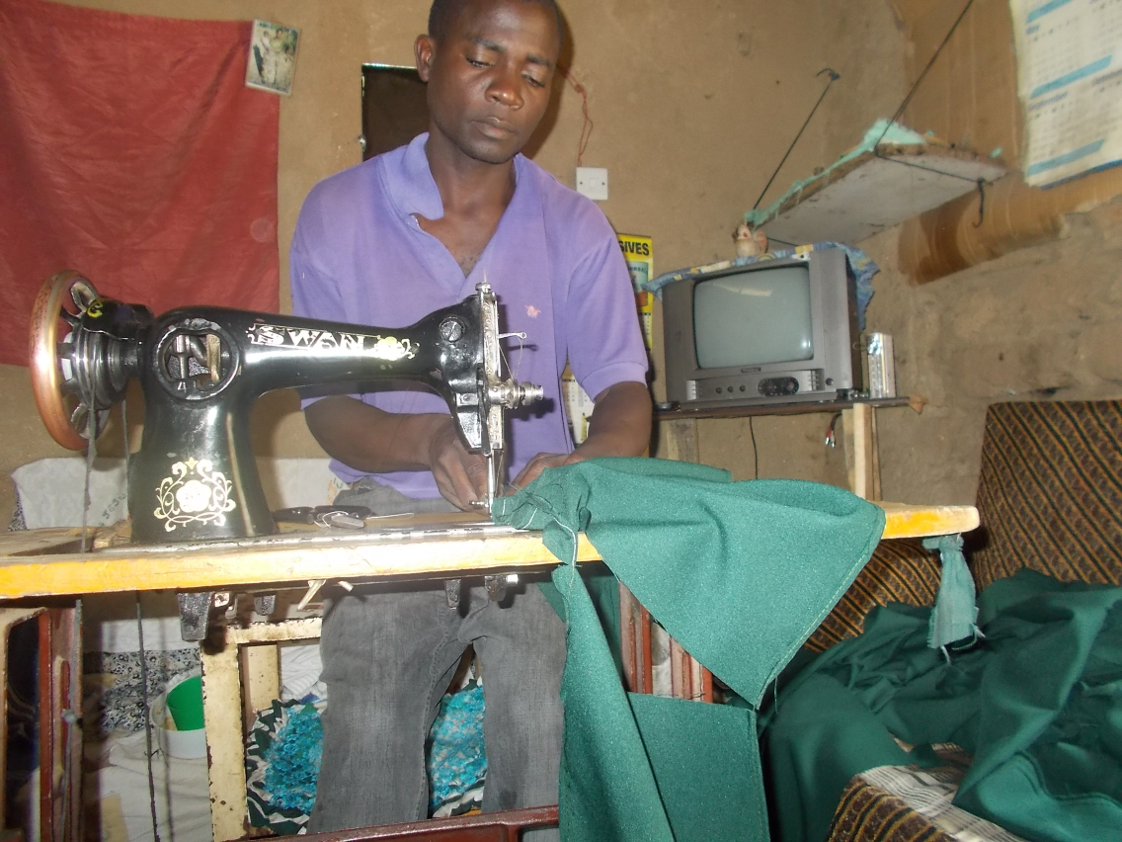

Supplement: S4 File — (ZIP) [file pone.0202490.s004.zip › example images/example24.tif]

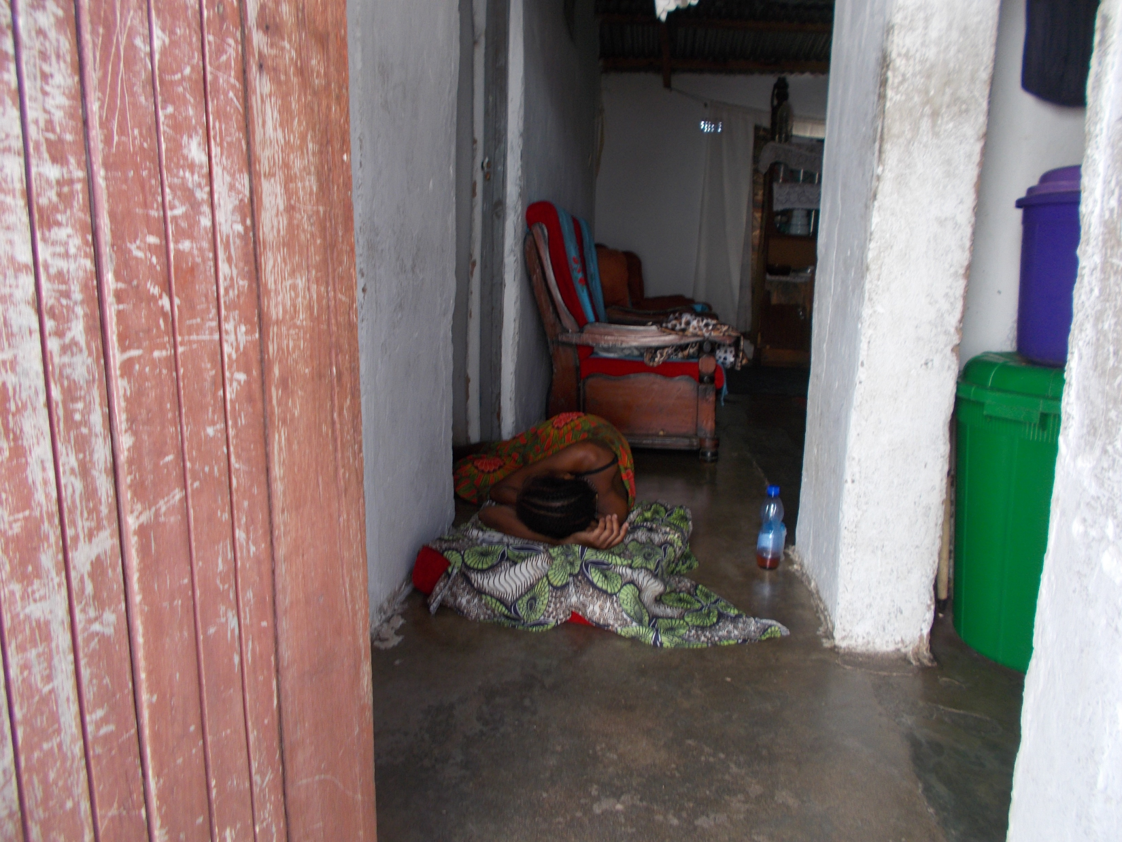

Supplement: S4 File — (ZIP) [file pone.0202490.s004.zip › example images/example25.tif]

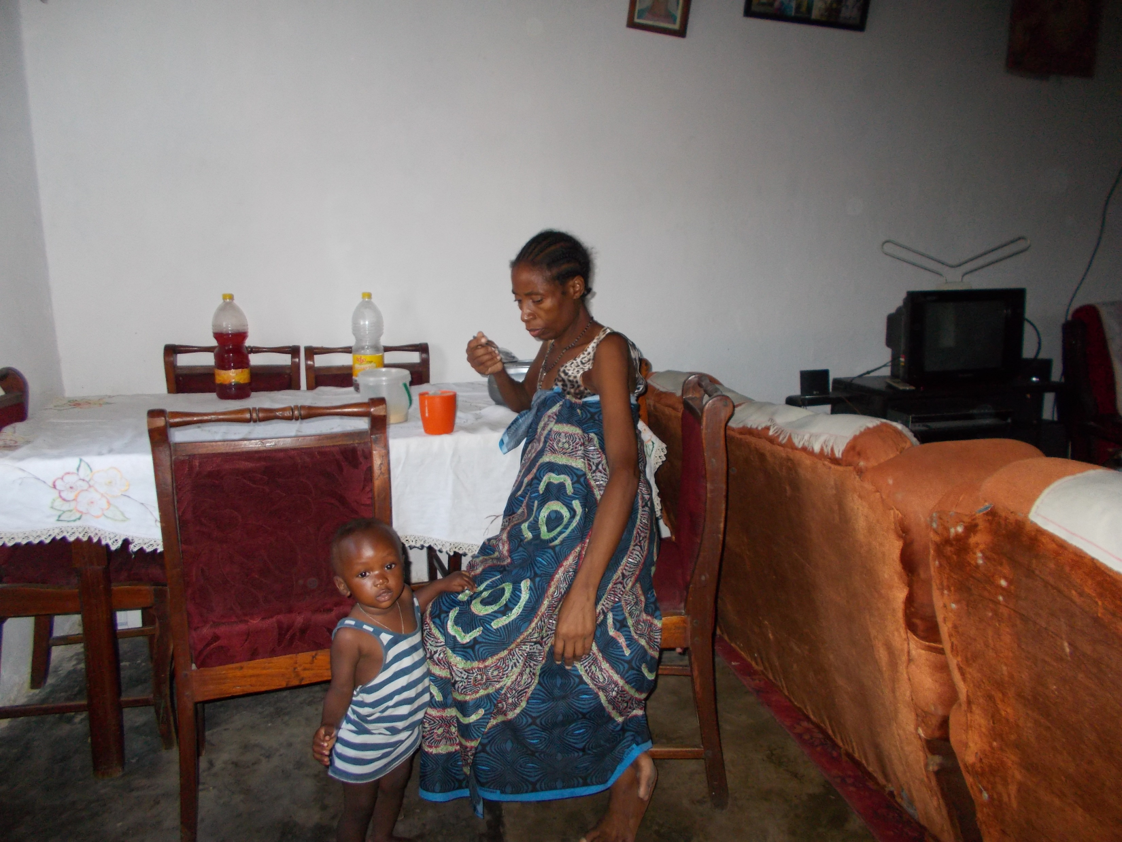

Supplement: S4 File — (ZIP) [file pone.0202490.s004.zip › example images/example26.tif]

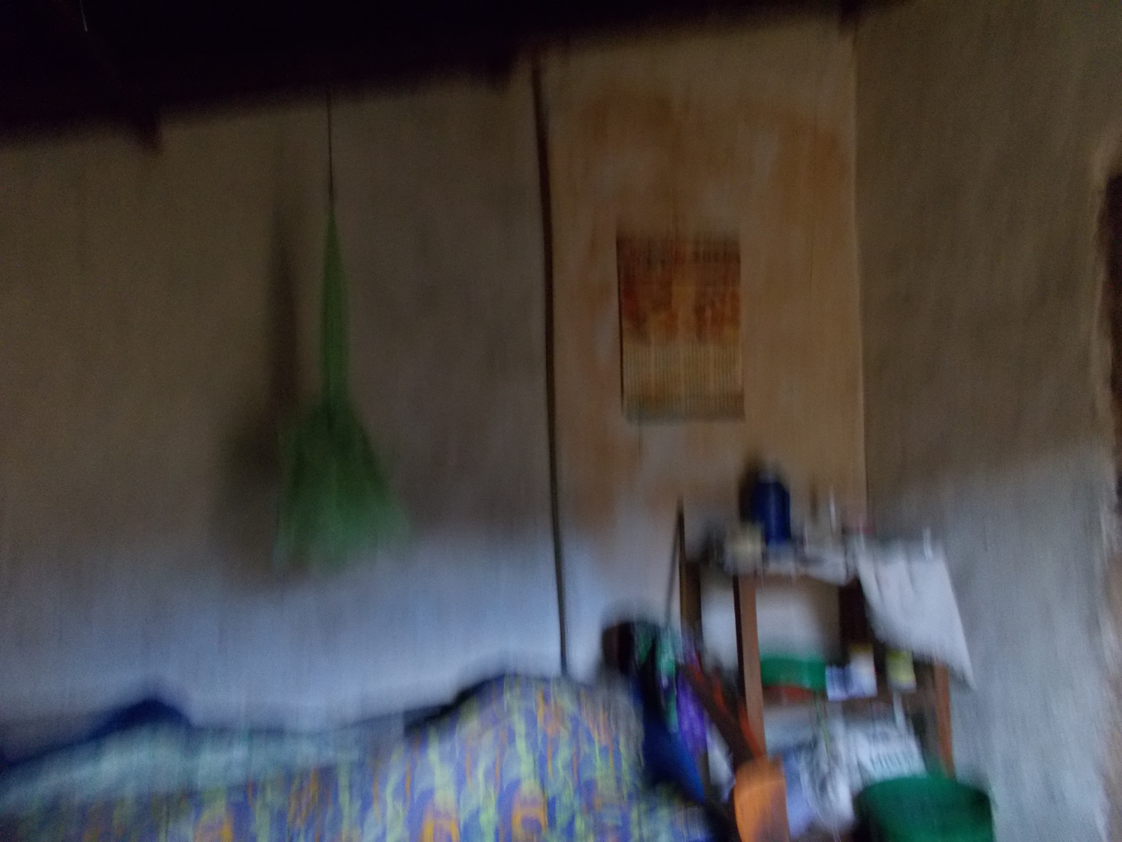

Supplement: S4 File — (ZIP) [file pone.0202490.s004.zip › example images/example3.tif]

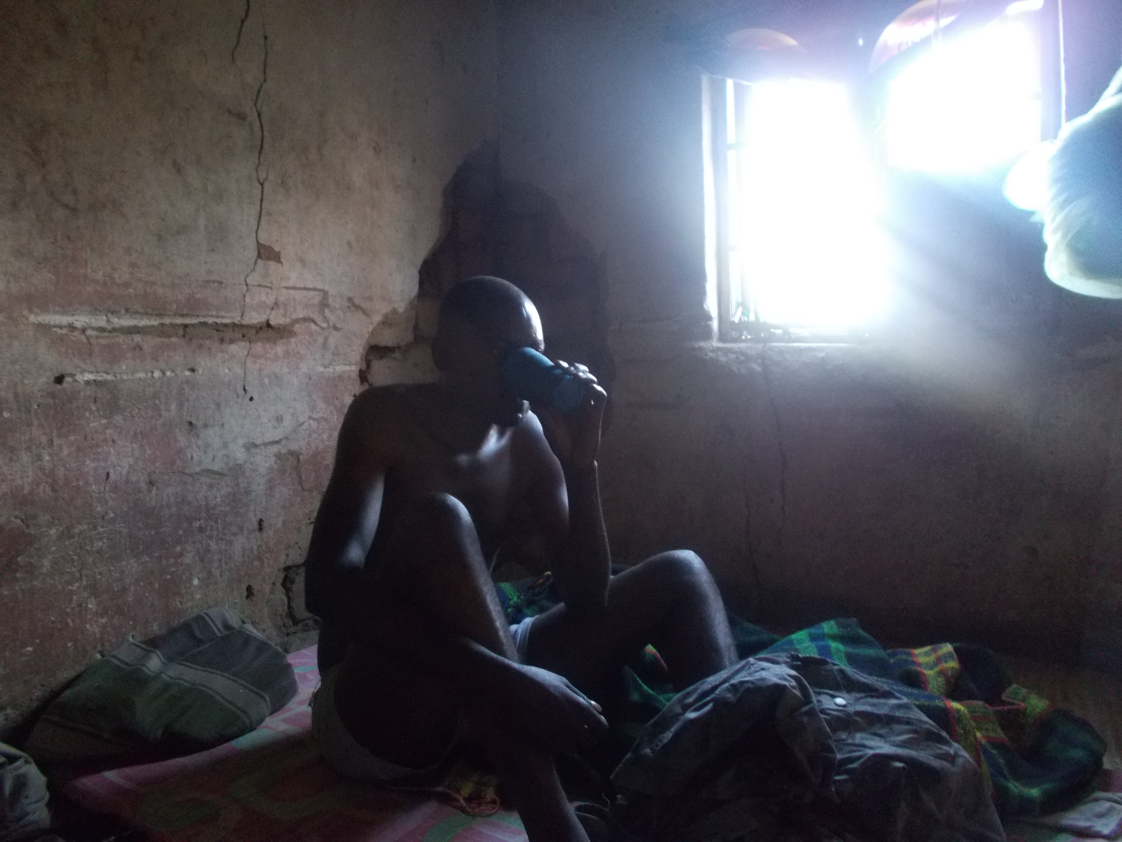

Supplement: S4 File — (ZIP) [file pone.0202490.s004.zip › example images/example4.tif]

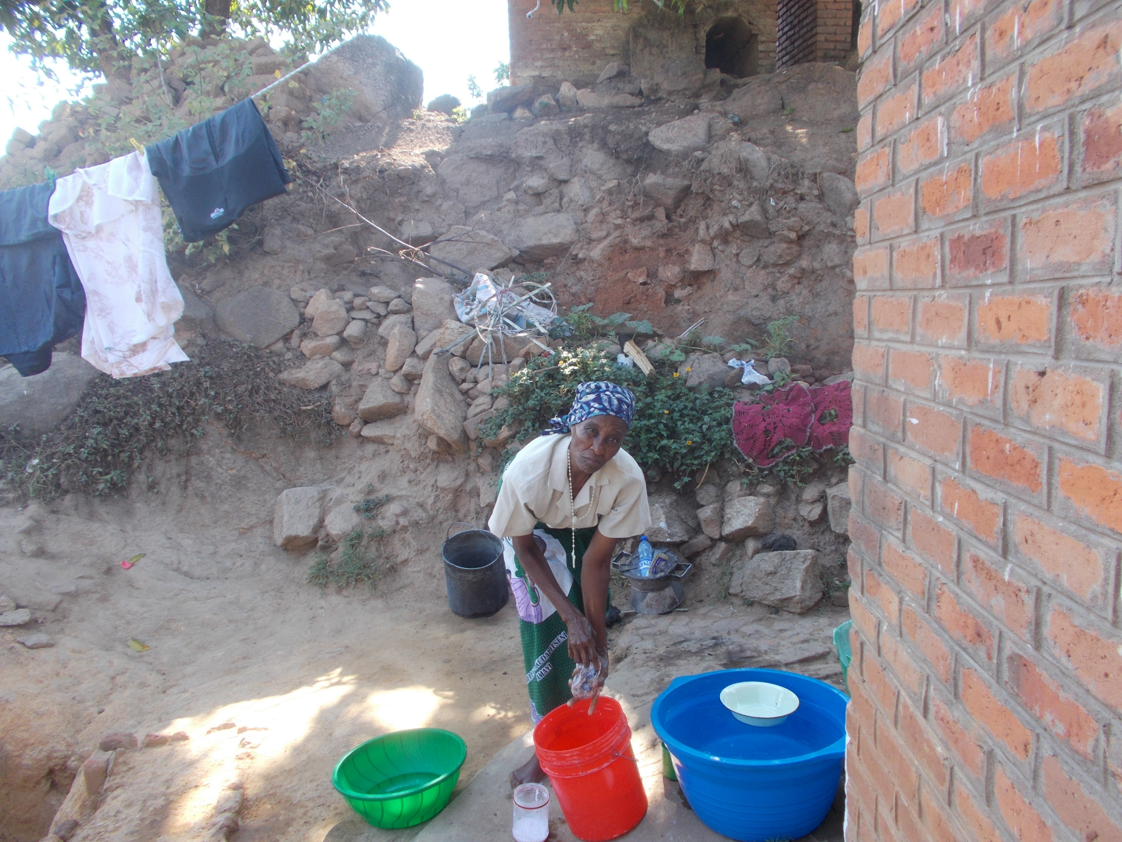

Supplement: S4 File — (ZIP) [file pone.0202490.s004.zip › example images/example5.tif]

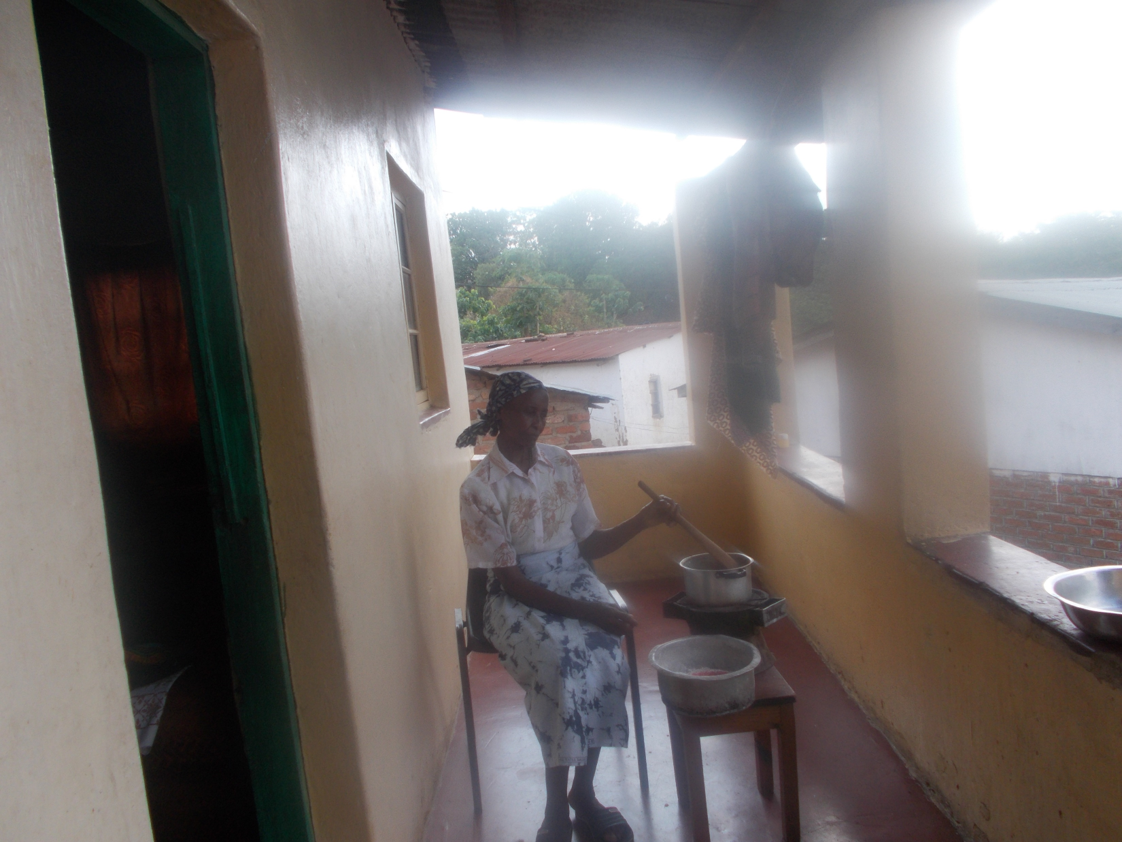

Supplement: S4 File — (ZIP) [file pone.0202490.s004.zip › example images/example6.tif]

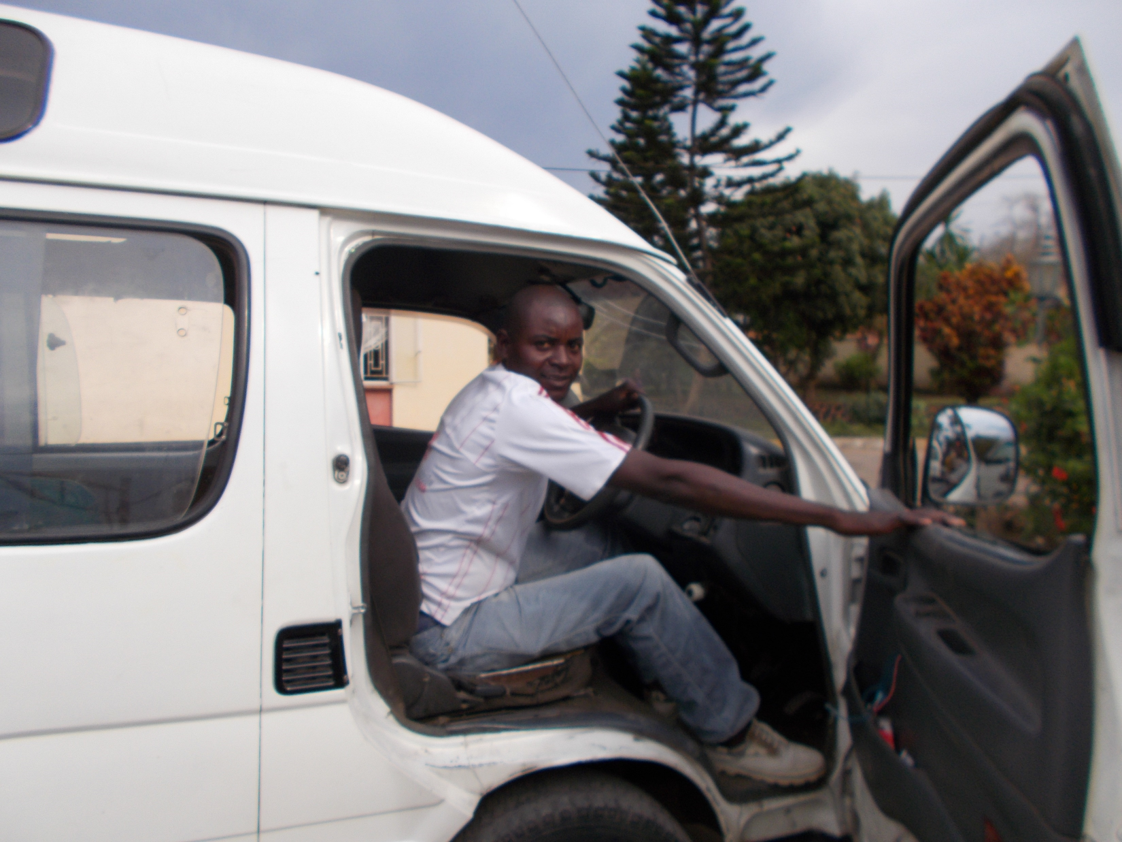

Supplement: S4 File — (ZIP) [file pone.0202490.s004.zip › example images/example7.tif]

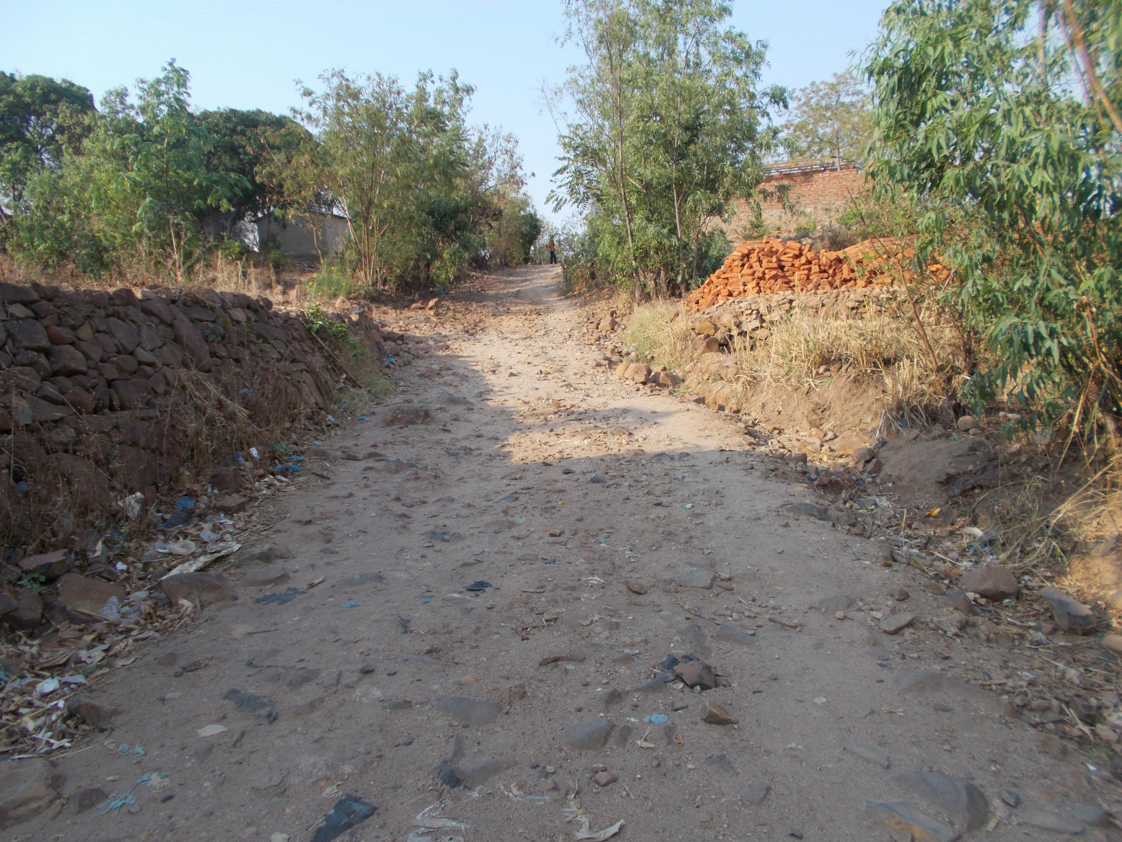

Supplement: S4 File — (ZIP) [file pone.0202490.s004.zip › example images/example8.tif]

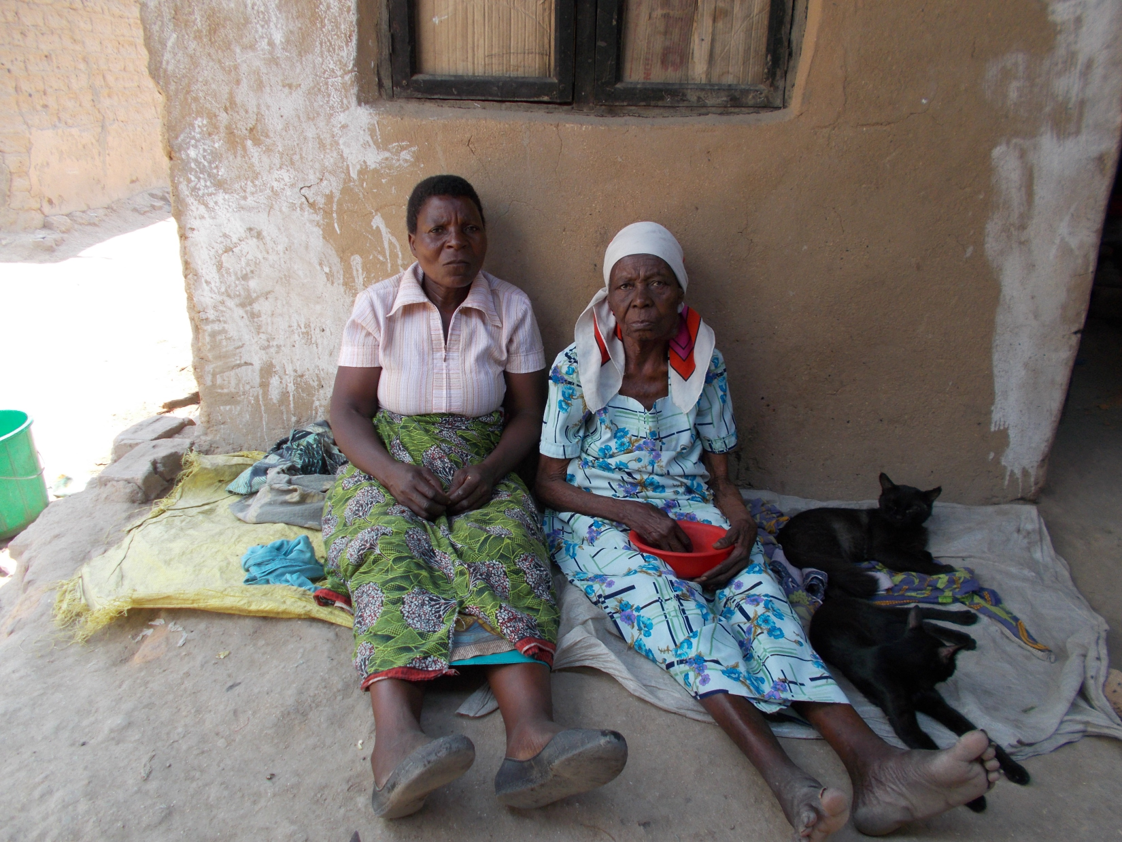

Supplement: S4 File — (ZIP) [file pone.0202490.s004.zip › example images/example9.tif]
